# Supplementary material for: The C-terminus of the prototypical M2 muscarinic receptor localizes to the mitochondria and regulates cell respiration under stress conditions
Source: PLoS Biol. 2024 Apr 29;22(4):e3002582. doi: 10.1371/journal.pbio.3002582 (PMC11093360; doi:10.1371/journal.pbio.3002582)
Supplement: S1 Raw Images — (PDF) [file pbio.3002582.s015.pdf]

Fig 3C

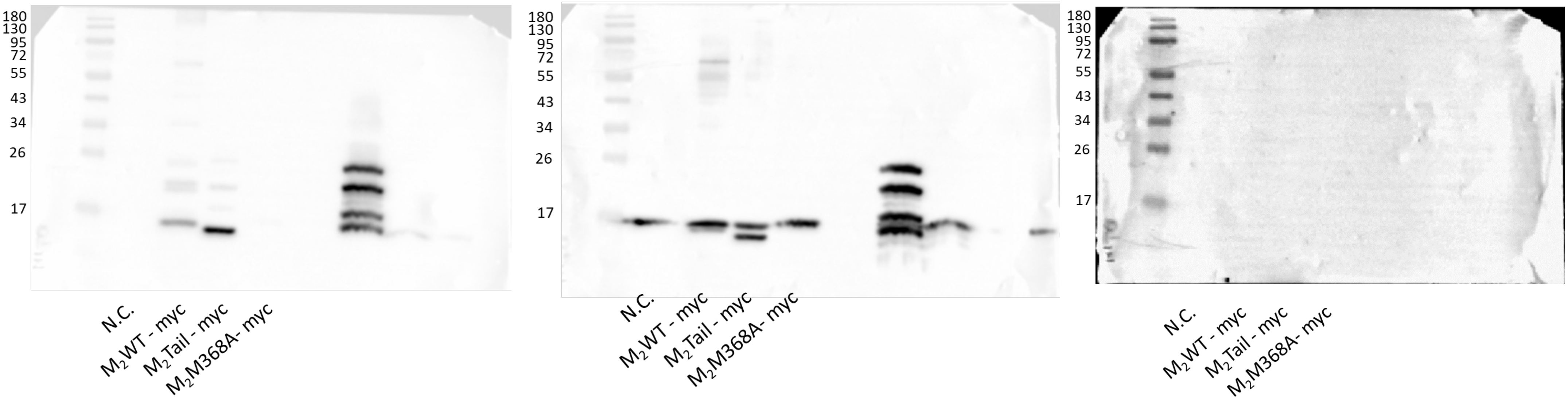

COX IV 15 KDa

β – actin 45 KDa

Fig 4C

autoradiograph

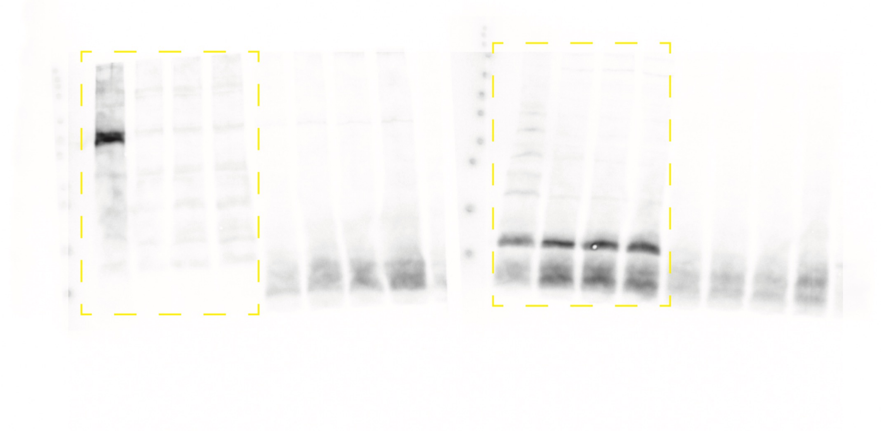

Porin loading control

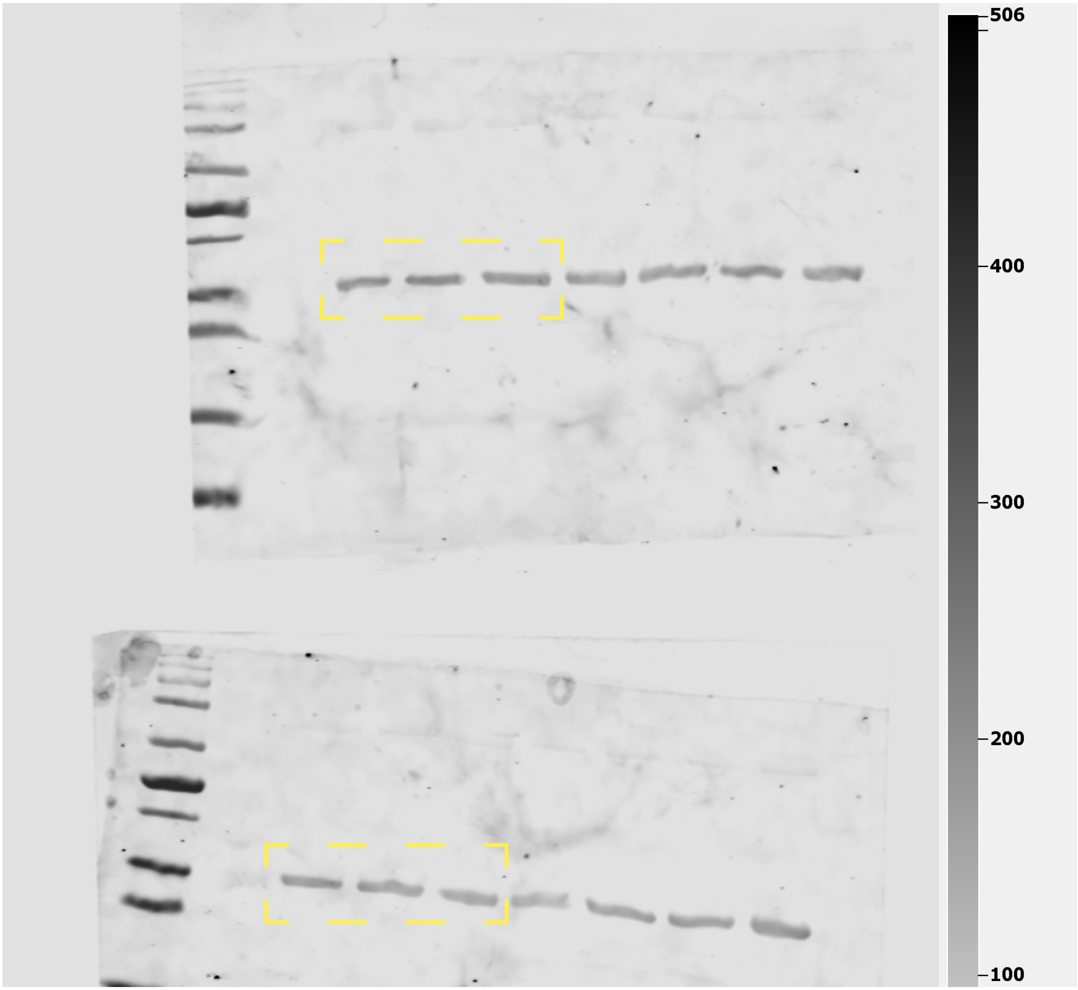

Fig 7A

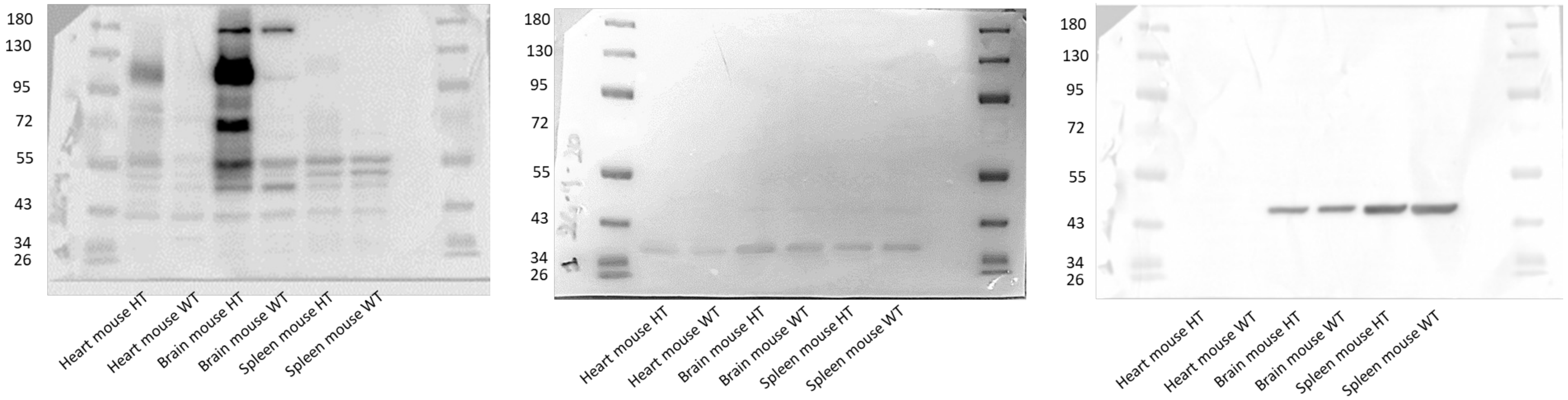

GAPDH 37 KDa

β-actin 45 KDa

Fig 7B

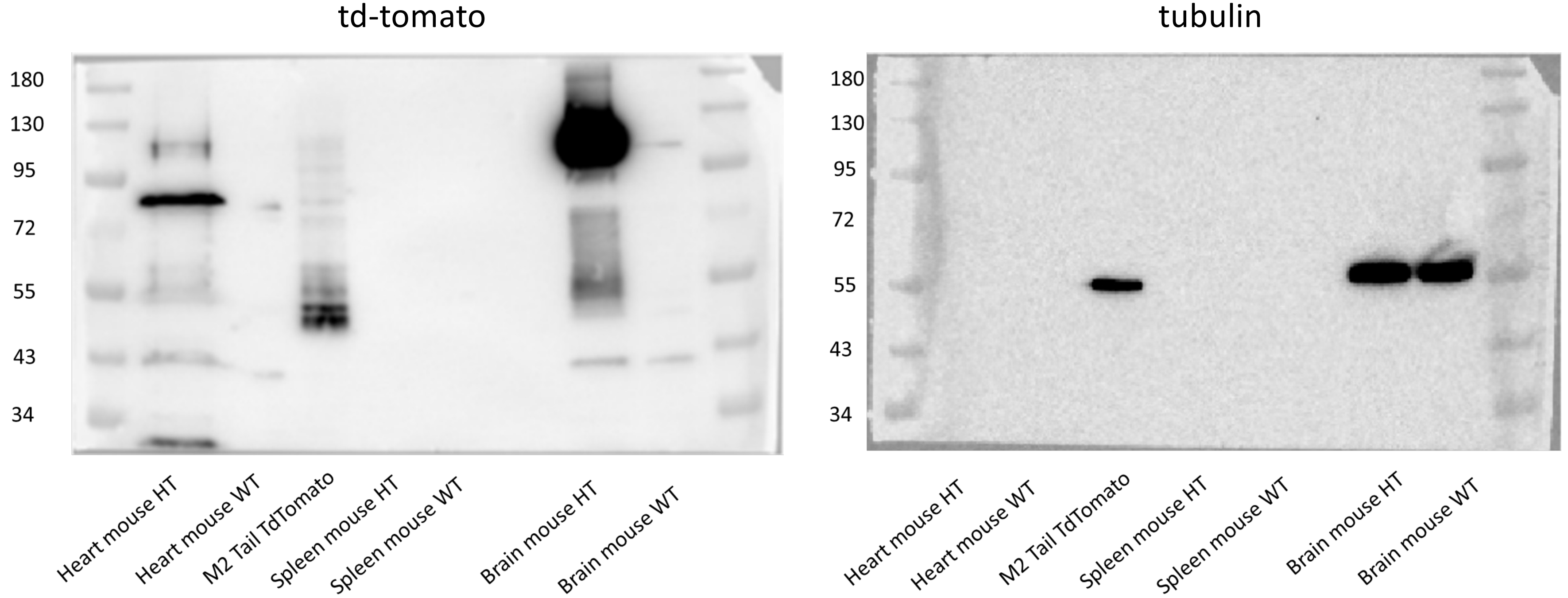
td-tomato
tubulin
180
130
95
72
55
43
34
Heart mouse HT
Heart mouse WT
M2 Tail TdTomato
Spleen mouse HT
Spleen mouse WT
Brain mouse HT
Brain mouse WT

Fig S2A

ERK

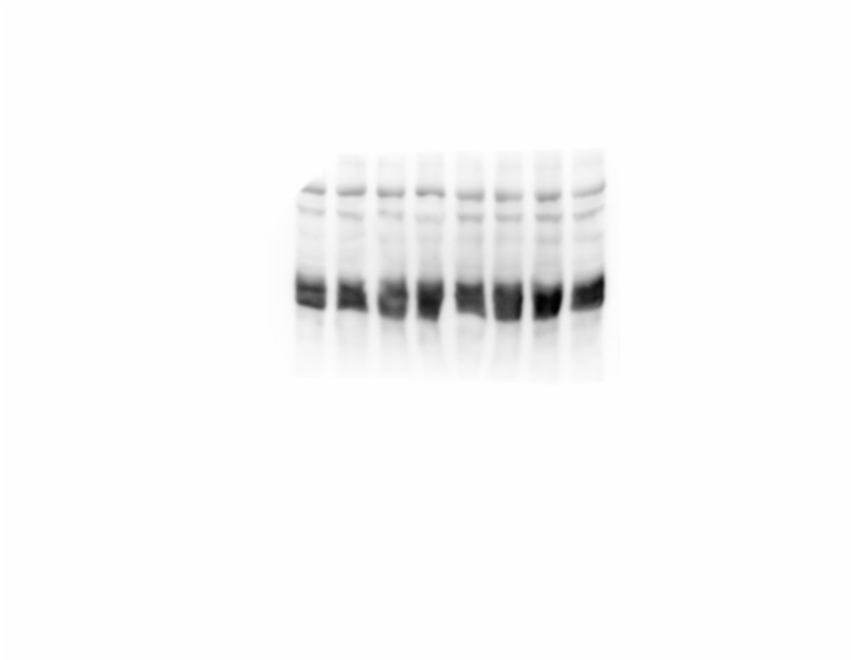

pERK

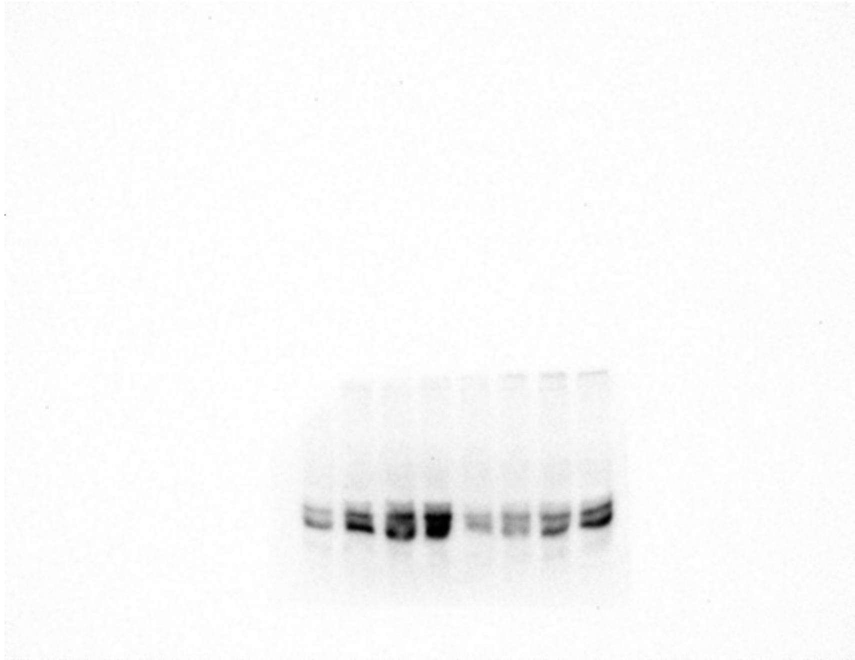

Fig S2B

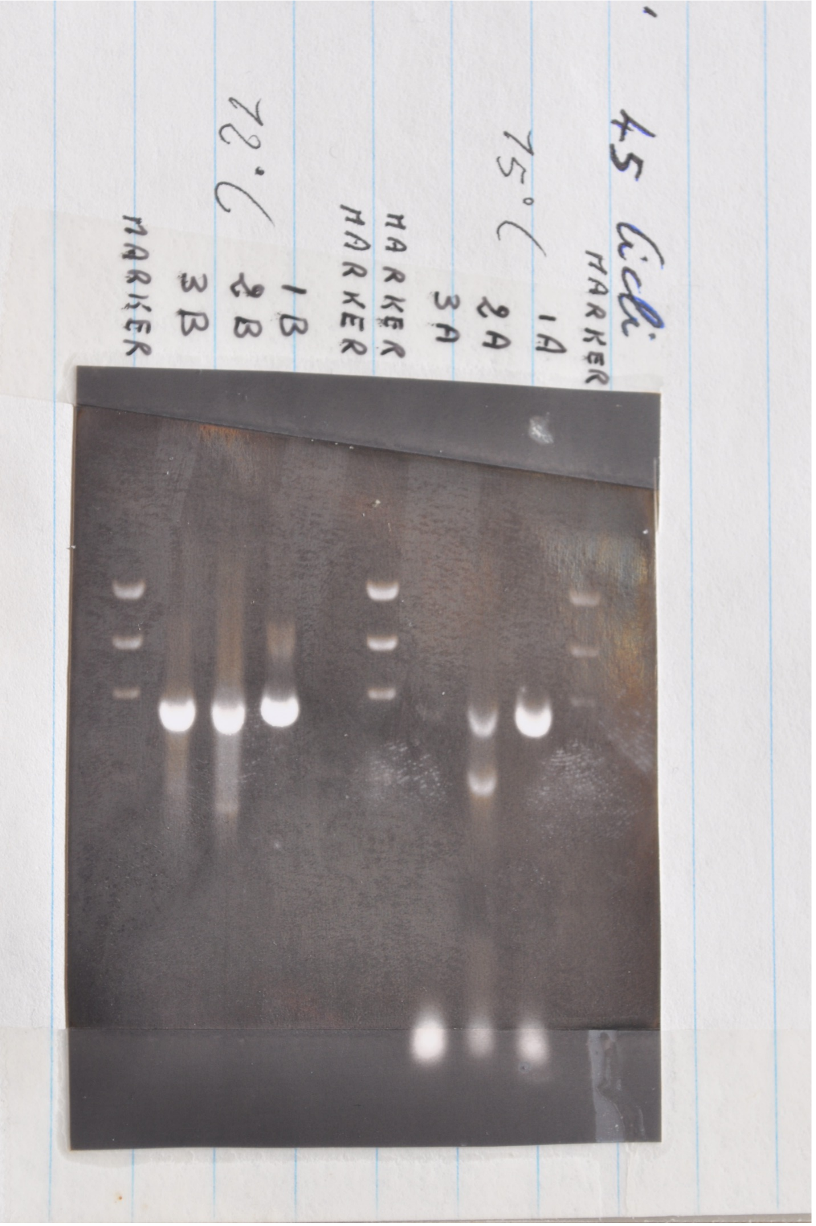

Fig S2C

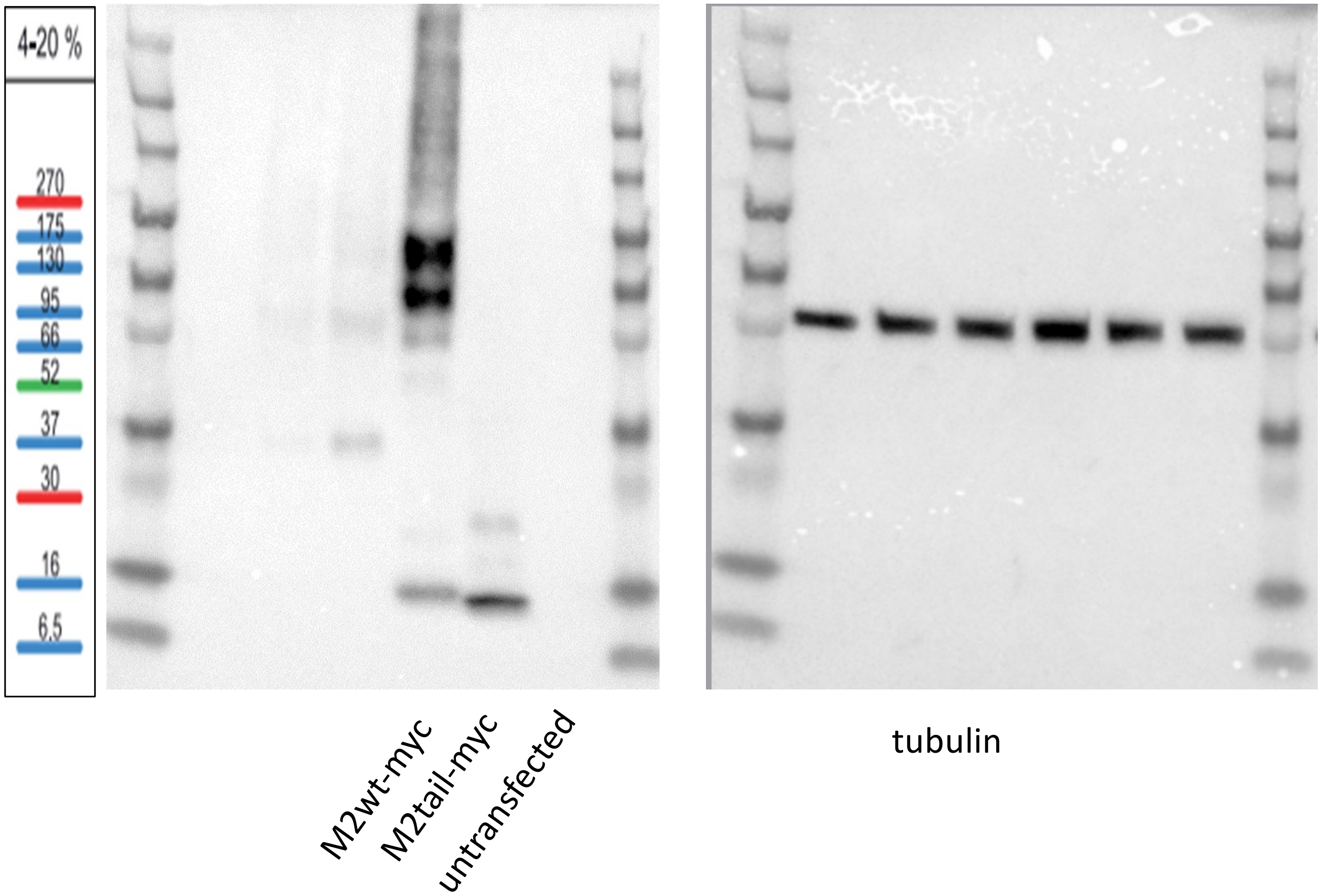
4-20 %
270
175
130
95
66
52
37
30
16
6.5
M2wt-myc
M2tail-myc
untransfected
tubulin

Fig S2D

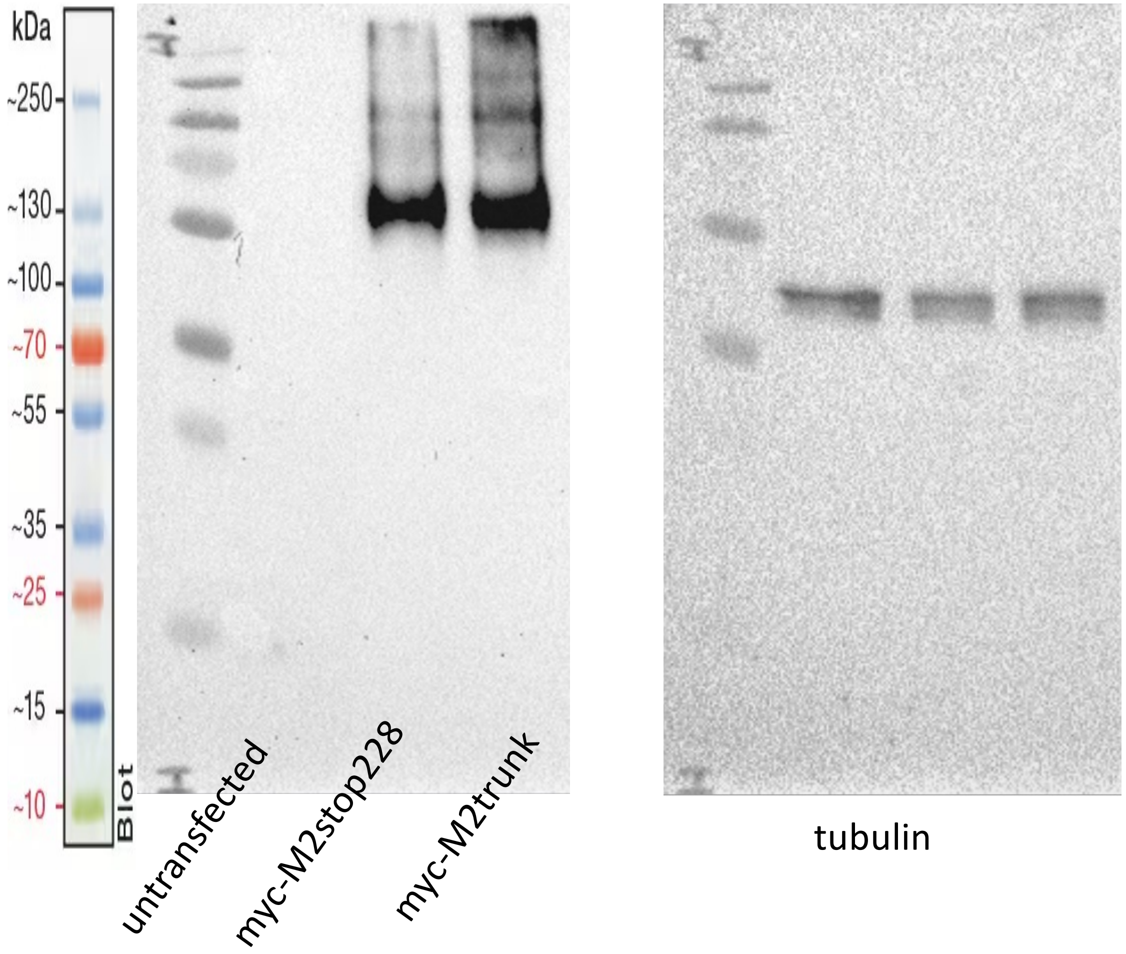
kDa
~250
~130
~100
~70
~55
~35
~25
~15
~10
Blot
untransfected
myc-M2stop228
myc-M2trunk
tubulin

Fig S2E

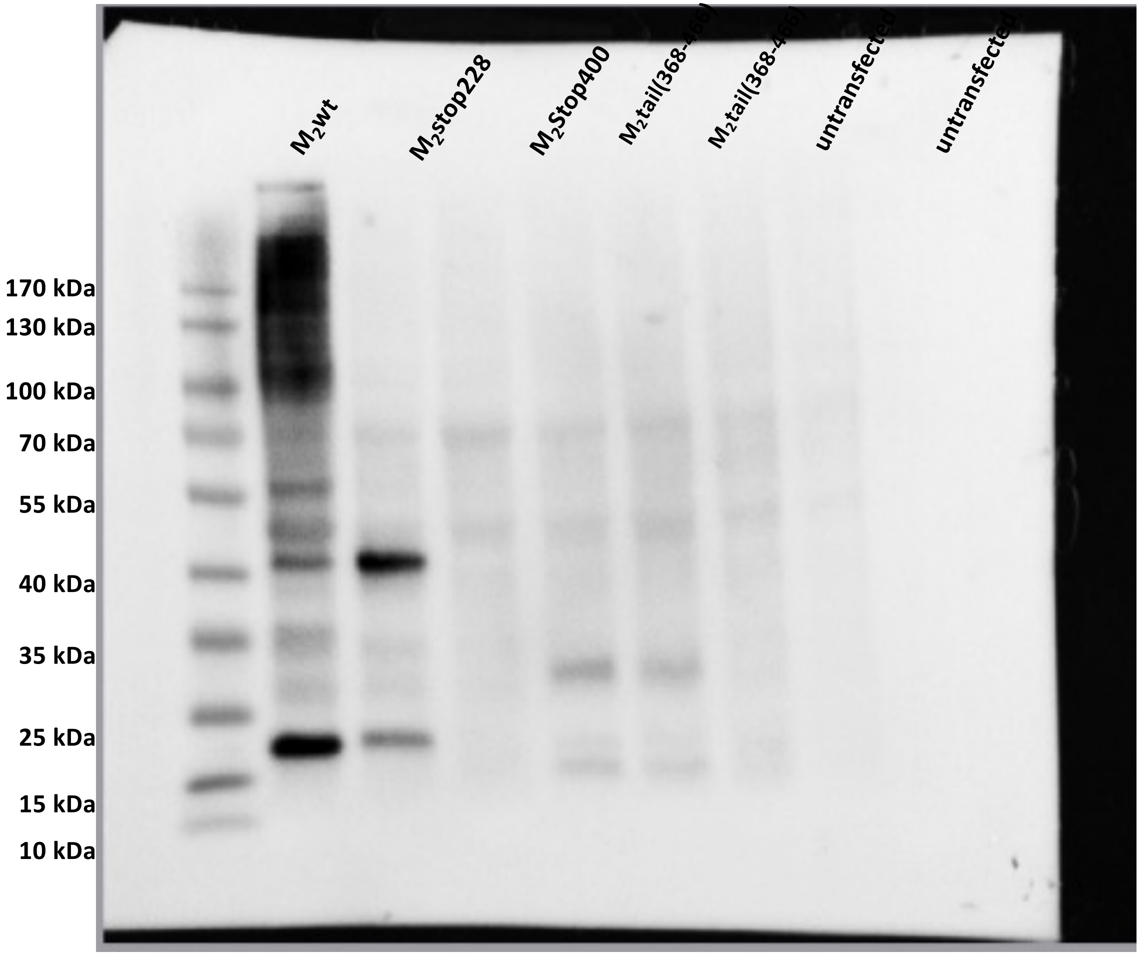

M2wt
M2stop228
M2Stop400
M2tail(368-466)
M2tail(368-466)
untransfected
untransfected
170 kDa
130 kDa
100 kDa
70 kDa
55 kDa
40 kDa
35 kDa
25 kDa
15 kDa
10 kDa

Fig S6K

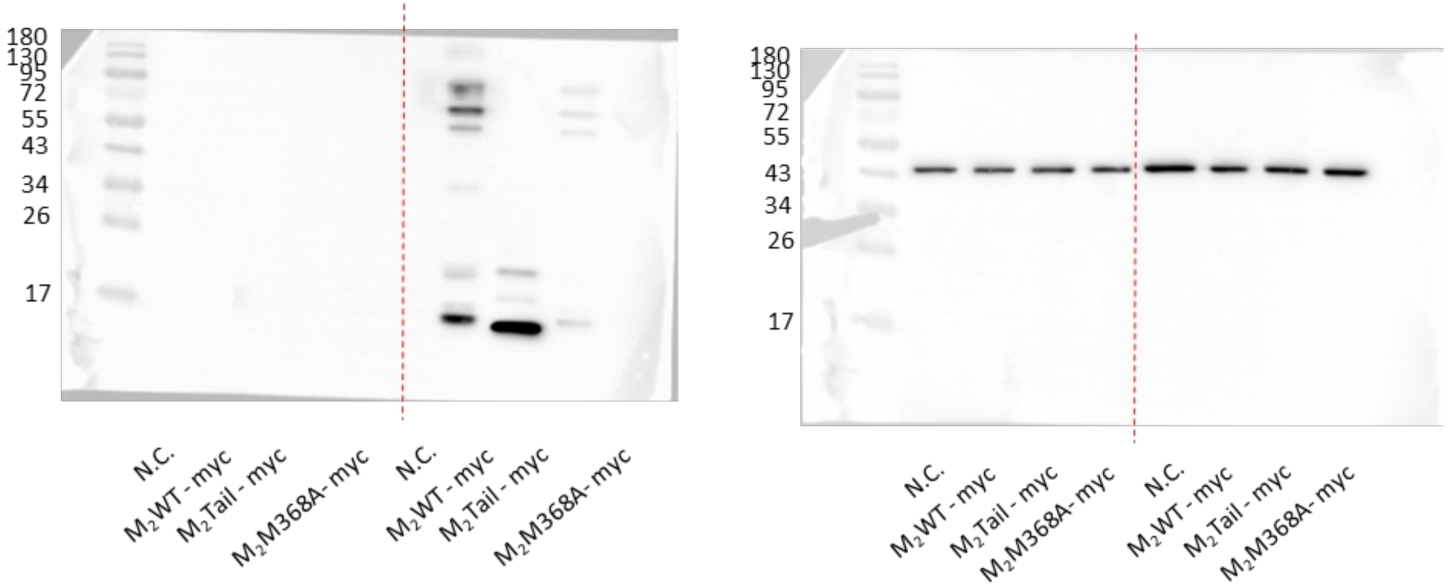

β -actin 45 KDa

Fig S7 (1/2)

Figure S7D BN-PAGE Autoradiograph (Porin) highlighted

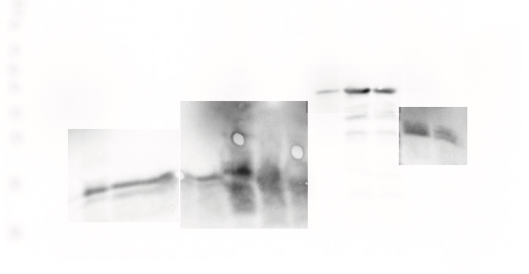

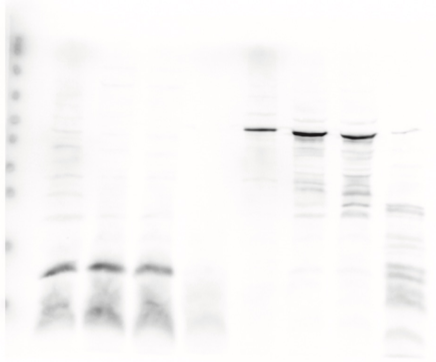

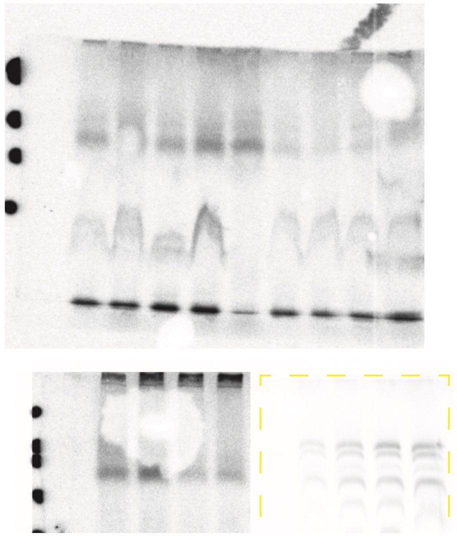

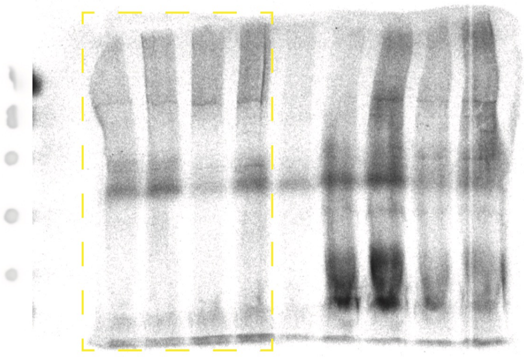

Fig S7 (2/2)

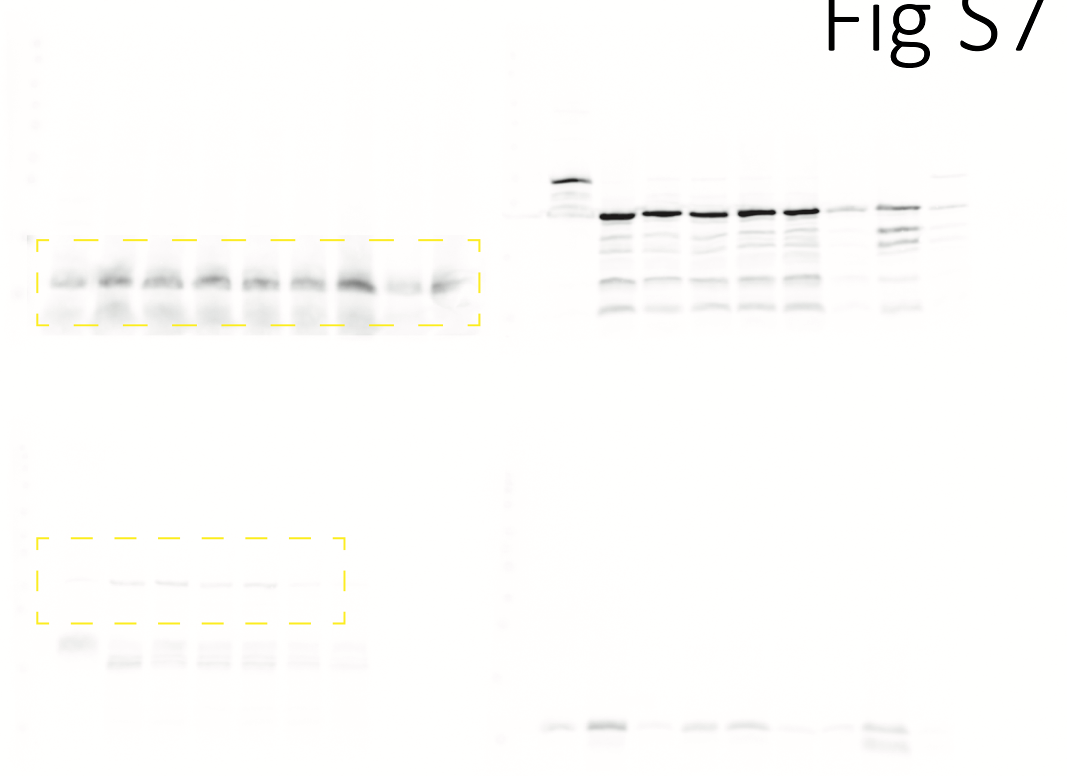

Figure S7D SDS-PAGE Autoradiograph highlighted

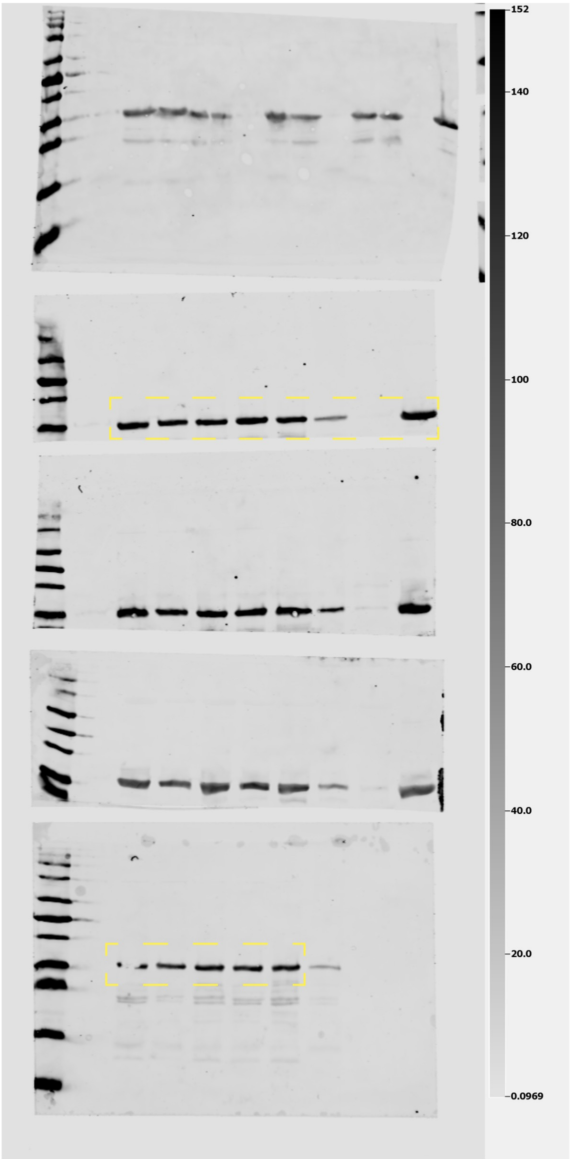

Figure S7D SDS-PAGE Loading Control Porin highlighted

Fig S10D 1/2

Caspase 3 normal exposure

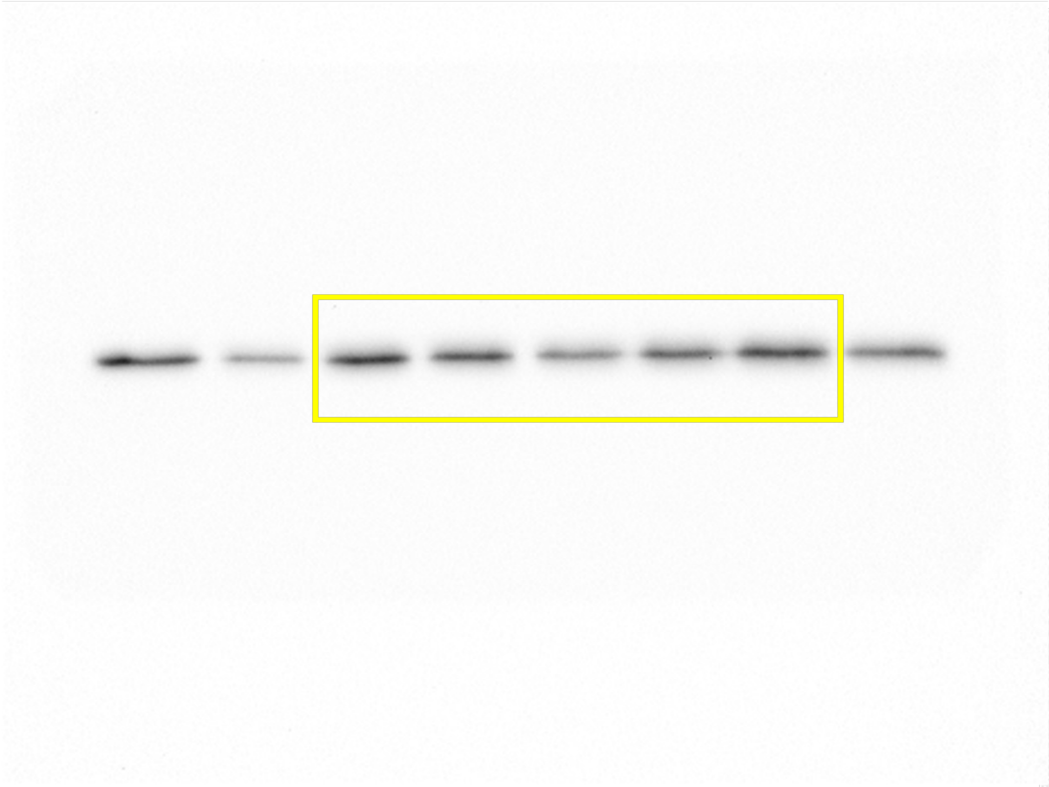

Caspase 3 overexposed

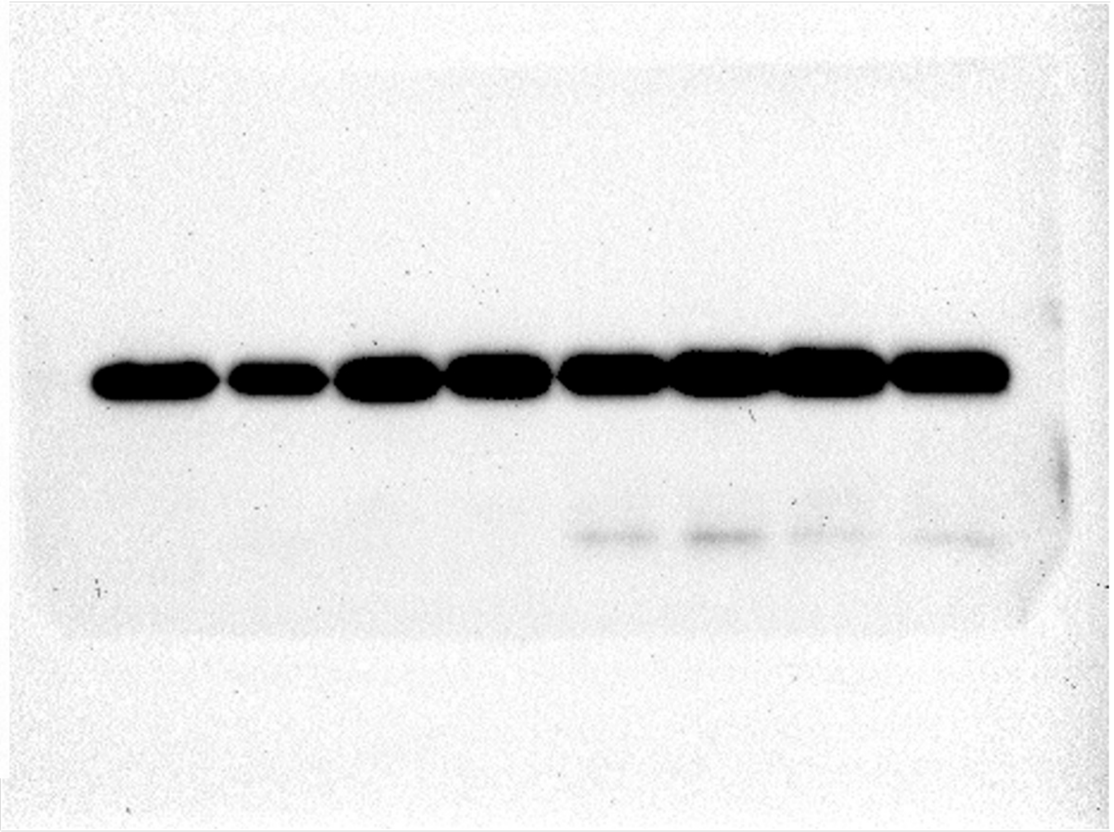

Fig S10D 2/2

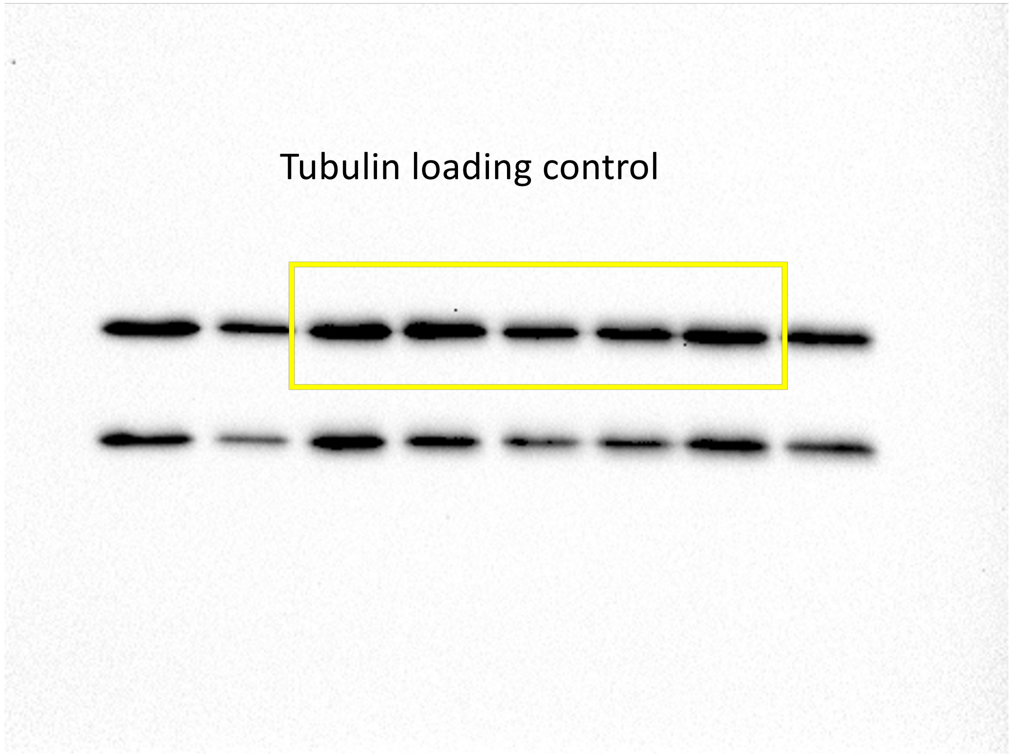
Tubulin loading control

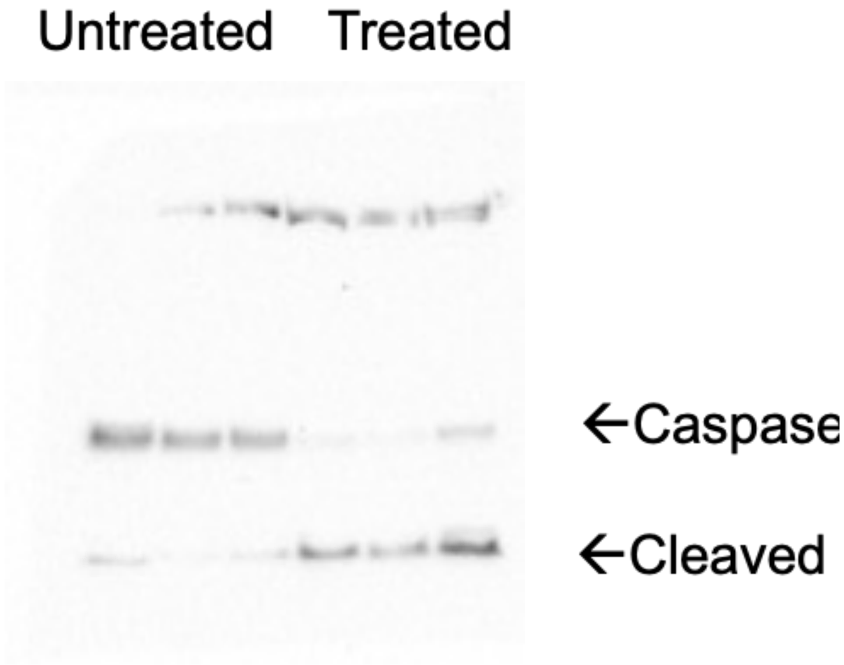
Untreated
Treated
←Caspase
←Cleaved
