# Supplementary material for: The C-terminus of the prototypical M2 muscarinic receptor localizes to the mitochondria and regulates cell respiration under stress conditions
Source: PLoS Biol. 2024 Apr 29;22(4):e3002582. doi: 10.1371/journal.pbio.3002582 (PMC11093360; doi:10.1371/journal.pbio.3002582)
Supplement: S1 Text — (DOCX) [file pbio.3002582.s016.docx]

Supplementary Information for

**The C-terminus of the prototypical M2 muscarinic receptor localizes to the mitochondria and regulates cell respiration under stress conditions**

Irene Fasciani^1†^, Francesco Petragnano^1†^, Ziming Wang^2^, Ruairidh Edwards^3^, Narasimha Telugu^2^, Ilaria Pietrantoni^1^, Ulrike Zabel^4^, Henrik Zauber^2^, Marlies Grieben^2^, Maria E. Terzenidou^3^, Jacopo Di Gregorio^1^, Cristina Pellegrini^1^, Silvano Santini Jr^5^, Anna R. Taddei^6^, Bärbel Pohl^2^, Stefano Aringhieri^7^, Marco Carli^7^, Gabriella Aloisi^1^, Francesco Marampon^8^, Eve Charlesworth^9^, Alexandra Roman^2^, Sebastian Diecke^2^, Vincenzo Flati^1^, Franco Giorgi^7^, Fernanda Amicarelli^5^, Andrew B. Tobin^3^, Marco Scarselli^7^, Kostas Tokatlidis^3^, Mario Rossi^1^, Martin J. Lohse^2,4,10^*, Paolo Annibale^2,4,9^*, Roberto Maggio^1^*,

**This PDF file includes:**

Supplementary Text

Figs. S1 to S10

Tables S1 to S3

References (1 to 14)

**Supplementary results related to the muscarinic M_2_ receptor**

**Ligand binding properties of** M_2_ **receptor mutants with single and double stop codons**

To understand the significance of the retained binding activity of the M_2_stop228, verify whether the sequence downstream of the receptor is indeed translated and characterize the role played by the position of the stop codon in conferring this capacity to the receptor, we analyzed a number of receptor constructs with a stop codon placed either upstream or downstream of the i3 loop (**Fig. 1A** and **Fig. S1 and S3**).

None of the two M_2_ receptor mutants bearing the stop codon in TM V and VI, such as M_2_stop196 and M_2_stop400, proves capable of binding [^3^H]NMS (**Table S1** ). However, binding could still be rescued by co-transfecting M_2_stop196 and M_2_trunk(1-283) together. Similarly, binding of M_2_stop400 could be restored by co-transfecting it with M_2_tail(M-281-466) (**Table S1**). These observations are in line with previous data showing that receptor fragments interact with defective mutants [1]. Furthermore, they also corroborate the hypothesis that the carboxyl terminal both of M_2_stop228 and M_2_stop196 are indeed translated, resulting in an interaction with the truncated fragments.

To understand why mutants bearing a stop codon in regions TMV, TMVI and i3 loop could be rescued by co-transfection, and gain new insight into the mechanism underlying their interaction, new receptor mutants containing two additional stop codons, namely M_2_stop196/stop400 and M_2_stop228/stop400, were created (**Fig. S3**). When transfected alone in COS-7 cells (**Table S1**), none of the above receptor mutants proved capable of binding [^3^H]NMS. Nevertheless, co-transfection of M_2_stop228/stop400 and M_2_tail(M-281-466) rescued the [^3^H]NMS binding activity up to a value of Bmaxs comparable to that already observed by co-transfecting M_2_trunk(1-283) with M_2_tail(M-281-466) (**Table S1, and Table 1**). Conversely, co-transfection of M_2_stop196/stop400 with any of the M_2_ fragments, M_2_trunk(1-283) and M_2_tail(M-281-466), did not result in [^3^H]NMS binding (**Table S1**). These data are indicative of the possibility that rescue of these receptor mutants depends entirely on the position of the stop codons. This is clearly demonstrated by the finding that the receptor cannot be rescued if the stop codons are present both in TMV and TMVI, as in M_2_stop196/stop400. Based on these results, it also appears not possible that during transfection plasmid sequences could undergo homologous recombination[2] .

**Excluding stop codon read-through, termination-reinitiation and alternative splicing in the translation of M_2_tail**

To rule out the possibility that the stop codon could be interpreted as a sense codon thus encoding an amino acid, a mechanism known as stop codon read-through[3, 4] , a frame shift was induced by inserting a sequence of four bases (AATT), fifteen nucleotides downstream of the stop codon 228: the mutant is hereafter named M_2_stop228/fr.sh (**Fig. S3**). Our data show that the occurrence of a frame shift following insertion of this artificial stop codon does not alter [^3^H]NMS binding activity of the mutant that essentially retains the Bmax values of 47 pmol/mg of protein (**Table S1**).

To check whether termination-reinitiation, a process known to occur in ribosomes translating the downstream Open Reading Frame (ORF) upon termination[5], was the mechanism responsible for the functional properties of the mutant M_2_stop228, a 42 bases long palindromic sequence was inserted 15 nucleotides downstream of the stop codon (**Fig. S3**). When transcribed into mRNA, this palindromic sequence is expected to form a hairpin structure with a ΔG of −64 kcal/mol, a value sufficient to block ribosome scanning[6]. Our data show that insertion of this hairpin loop after the stop codon reduced slightly the [^3^H]NMS binding activity of the receptor, attaining a Bmax value of 37 pmol/mg protein (**Table S1**). This evidence implies that termination-reinitiation is not the mechanism that accounts for [^3^H]NMS binding of M_2_stop228. These results were replicated by using similar mutants of the muscarinic M_3_ receptor (**Table S2 and S3, see below**).

To verify whether a correct reading frame could be restored by the removal of the stop codon, through a mechanism of alternative splicing, mRNA was extracted from cells expressing M_2_ and M_2_stop228 and subjected to reverse transcriptase (see Materials and Methods). As shown in **Fig. S2B**, PCR amplification of the reverse transcript was resolved and detected with gel electrophoresis and showed a single band comparable in size to both M_2_ and M_2_stop228 receptor mRNAs, as it was for the control M_2_ cDNA plasmid.

**Ligand binding properties of M_2_stop228/stop248, M_2_stop228/stop296, and M_2_stop228/stop368**

To find out if any of the in-frame start codons was responsible for the translation of the carboxyl terminal fragment of the M_2_stop228 receptor, the three ATG codons in frame after the stop codon 228 (**Fig. S1**), were each mutated to an additional stop codon. This yielded three new mutants, hereafter referred to as M_2_stop228/stop248, M_2_stop228/stop296, and M_2_stop228/stop368 (**Fig. S3**). The first two mutants proved still capable of binding [^3^H]NMS with M_2_stop228/stop248 and M_2_stop228/stop296, having Bmaxs of 49 fmol/mg and 38 fmol/mg of protein, respectively (**Table S1**). On the contrary, suppression of the third ATG in M_2_stop228/stop368 abolished [^3^H]NMS binding completely (**Table S1**), even though this capacity could be rescued by co-transfecting it together with the M_2_tail(M-281-466) construct (**Table S1**).

**Preliminary analysis of the bicistronic Sirius-H-M_2_i3(15n/30n)-EGFP construct to individuate the nucleotides required for internal ribosome entry**

The sequence alignment of the 33 nucleotides, from 1072 to 1104 of the M_2_ i3 loop, with regions adjacent to the second (M_1_), fourth (M_3_ and M_4_) and first (M_5_) in frame ATGs of the other muscarinic receptors, revealed some conserved nucleotides, specifically an A in position 1078 and the three nucleotides AAG in position1090-1092 (four nucleotide AAGA 1090-1093 if we consider the two most related muscarinic M_2_ and M4 receptors); furthermore the position of the ATG was conserved in four out of five muscarinic receptors (**Fig. S4C**).

Mutation of the conserved nucleotide A1078 with T, as in the Sirius-H-M_2_i3(15n/30n)Mut#1-EGFP construct, reduced by half the EGFP fluorescence intensity compared to Sirius-H-M_2_i3(15n/30n)-EGFP (**Fig. 2C**). Mutation of the four nucleotides AAGA1090-1093 with TTTT, as in the Sirius-H-M_2_i3(15n/30n)Mut#2-EGFP construct, sharply decreased EGFP expression, retaining only a residual fluorescence of 24 ± 14% as respect to Sirius-H-M_2_i3(15n/30n)-EGFP (**Fig. 2C**). Control experiments with mutants where non-conserved nucleotides were replaced, specifically Sirius-H-M_2_i3(15n/30n)Mut#3-EGFP and Sirius-H-M_2_i3(15n/30n)Mut#4-EGFP did not alter significantly EGFP expression (**Fig. 2C**).

Eventually we decided to alter the sequence that contains the residues that affected more dramatically EGFP expression. To this end, we mutated in T the sequence comprised between nucleotides 1083 and 1101, as in the Sirius-H-M_2_i3(15n/30n)Mut#5-EGFP mutant. The nucleotide AAGA (1090-1093) was then reinserted in this mutant as in the Sirius-H-M_2_i3(15n/30n)Mut#6-EGFP construct. As shown in **Fig. 2C**, only 12.3 ± 10.3% of the green fluorescence originally present in Sirius-H-M_2_i3(15n/30n)-EGFP was retained in the Sirius-H-M_2_i3(15n/30n)Mut#5-EGFP mutant. This loss, however, could be recovered up to 118 ± 7% of the Sirius-H-M_2_i3(15n/30n)-EGFP level by re-inserting the four AAGA nucleotides into its sequence as in the Sirius-H-M_2_i3(15n/30n)Mut#6-EGFP construct (**Fig. 2C**).

**Localization of the M_2_-Muscarinic Receptor C-terminal upon cell stress**

We studied the expression of M_2_tail(368-466)-mRuby2 – originated as a segment of the M_2_-mRuby2 construct – and of M_2_tail(368-466)-EGFP – originated as a segment of the M_2_-i3-tail-EGFP part – both derived from the M_2_-mRuby2-STOP-M_2_-i3-tail-EGFP mega construct, after serum starvation. **Fig. S9A-E** shows representative images of the mitochondrial localization of M_2_tail(368-466)-EGFP and M_2_tail(368-466)-mRuby2, in HEK293 cells stained with Mitotracker following two hours of starvation in PBS. As it can be clearly seen, expression of both M_2_tail(368-466)-EGFP and M_2_tail(368-466)-mRuby2 is visible in mitochondria, whereas at the plasma membrane only a M_2_-mRuby2 signal is visible but none of the EGFP signal.

To further validate this observation, we repeated the exact same experiment where the mega construct was mutated in the third in frame methionine, resulting in M_2_(M368A)-mRuby2-STOP-M_2_-i3-tail-EGFP (**Fig. S3**). This time we did not observe expression of M_2_tail(368-466)-mRuby2, and therefore no mRuby2 signal was visible in mitochondria **Fig. 9F-L**. On the other hand, M_2_tail(368-466)-EGFP was still observed within mitochondria.

**Influence of the M_2_-muscarinic receptor C-terminal fragment on mitochondrial oxygen consumption measured with the Clarke electrode**

The extent of oxygen consumption in COS-7 cells expressing the wild-type M_2_, M_2_stop228 and M_2_tail(368-466) was also measured with the Clarke electrode. Our data show that no difference could be revealed, in term of total oxygen consumption, between cells transfected with the wild-type M_2_ receptor or mock transfected cells (**Fig. S10B,C**). However, a sizable decrease of oxygen consumption rate was instead revealed, as compared to controls, in cells co-transfected with M_2_stop228 and M_2_tail(368-466) (**Fig. S10B,C**).

By comparison, cells transfected with the M_2_trunk(1-228) fragment, did not change significantly their oxygen consumption rate. In order to evaluate whether O_2_ depletion is coupled to oxidative phosphorylation, the ATP synthase inhibitor oligomycin, at the concentration of 17 nM, was added to the culture medium. The observation that oligomycin addition caused O2 consumption rate to decrease consistently in control samples, proved that the O2 consumption rate was coupled to oxidative phosphorylation (**Fig. S10B**), with an average value of 45 ± 6%. Oligomycin addition reduced also the O2 consumption rate of COS-7 cells transfected with M_2_ and M_2_trunk(1-228), while it only slightly reduced oxygen consumption rate in cells transfected with M_2_stop228 and M_2_tail(368-466) (**Fig. S10B,C**), thus suggesting that C-terminal-M_2_ peptide suppresses only that portion of the O2 consumption rate that is coupled to the oxidative phosphorylation. It is worth nothing that in this assay the wild type M_2_ receptor did not inhibit O2 consumption at variance with the Seahorse assay. The most parsimonious hypothesis to explain this discrepancy could be that in the assay with the Clarke electrode cells were not starved then the cap dependent translation is prevailing.

**Supplementary Figures and Tables**


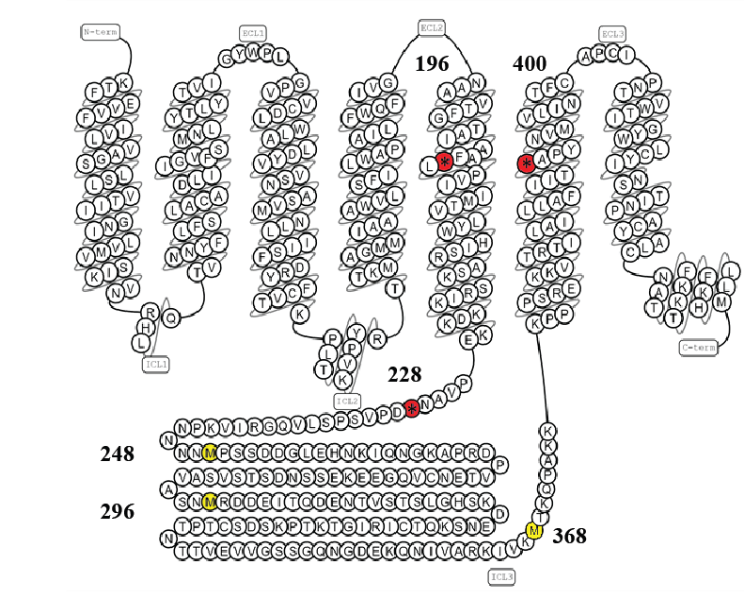


**Fig. S1.** **Snake diagram of the M_2_ receptor.**

Key residues are highlighted[13]. Asterisks in the red circles denote the insertion of stop codons within transmembrane regions V (196) and VI (400), as well as the i3 loop (228). The in-frame methionine highlighted in yellow are the ones within the third loop that have been substituted with stop codons to investigate the initiation start site of M_2_ Cterminal fragment.


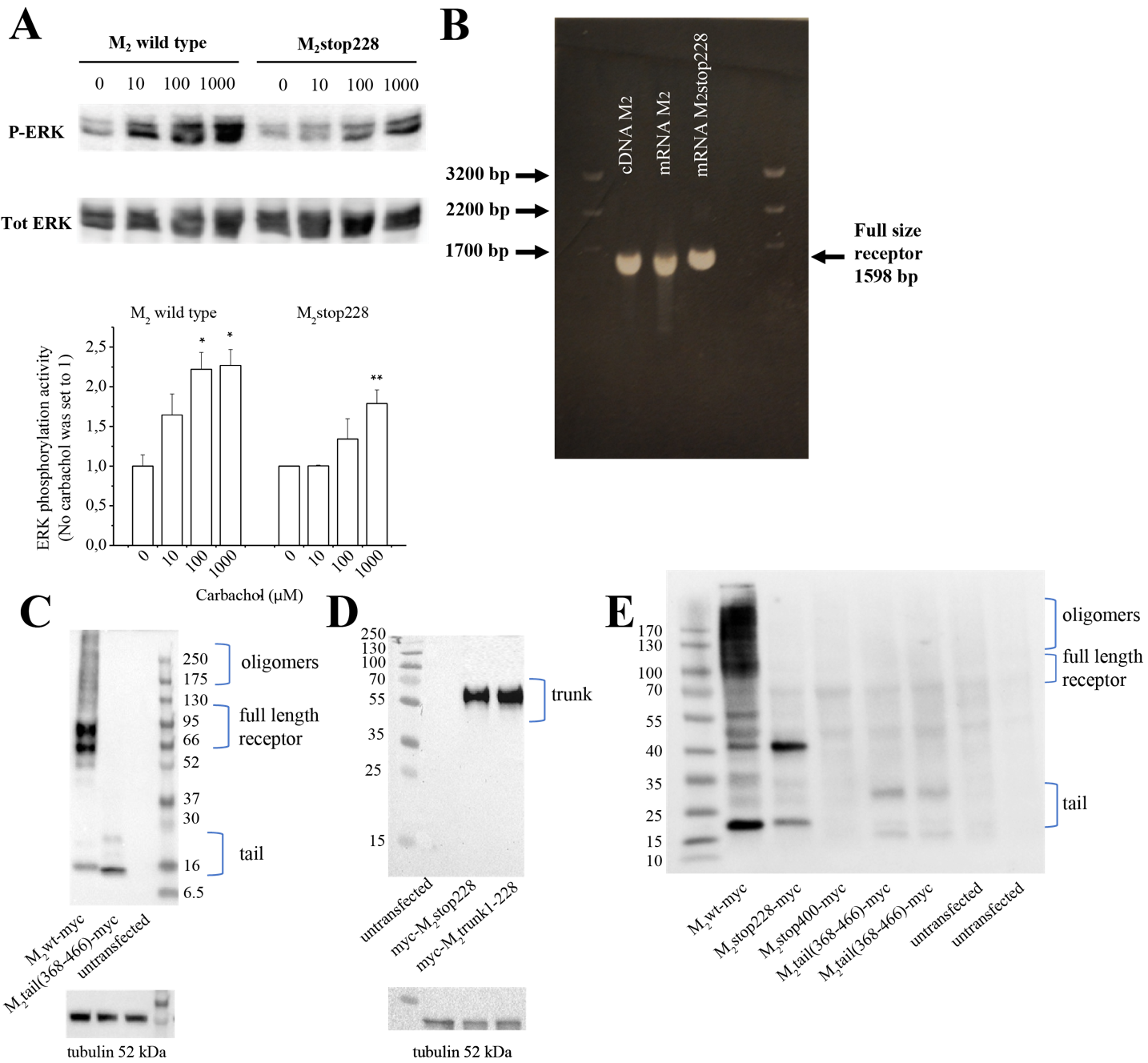


**Fig. S2.** **M_2_ receptor mutants: signalling, alternative splicing and Western blotting**.

**A,** Dose response curve of carbachol induced ERK phosphorylation in HeLa cells transiently transfected with M_2_ and M_2_stop228 muscarinic receptors. Cells were stimulated for 5 minutes with the indicated concentration of carbachol. Phosphorylated ERK (P-ERK) was normalized vs total ERK (Tot ERK). Significance values reported in the graphs were determined by a one-tailed Student t-test, p-values: ** 0.001<p<0.01; * 0.01<p<0.05 was calculated against carbachol 0 µM. **B,** mRNAs were extracted by COS-7 cells transfected with M_2_ wild type and M_2_stop228, subjected to reverse transcriptase and the resulting cDNA amplified by PCR with two oligos directed to the 5’ and 3’ end of the receptors. The gel shows, in both M_2_ wild type and M_2_stop228, a single band of 1401 bp corresponding to the full-size receptor, running at the same level of the band amplified directly from the M_2_ pcD plasmid. **C,** Western blot of whole cell lysates of HEK293 cells. M_2_-Myc and M_2_tail(368-466)-Myc were transiently transfected in HEK293 cells and immunodetected via Western blot together with an untransfected control. Loading control was verified by immunoblotting tubulin. **D,** Western blot of whole-cell lysates untransfected HEK293 (lane 1) and then HEK293 cells transfected with myc-M_2_Stop228 (Lane 2) and myc-M_2_Trunk(1-228) (Lane 3). Loading control was verified by detecting tubulin. **E,** Western blot of whole cell lysates of COS-7 cells. M_2_-Myc, M_2_stop228-Myc, M_2_tail(368-466)-Myc, M_2_stop400-Myc were transiently transfected in COS-7 cells and immunodetected via Western blot together with an untransfected control. Source data for panel A can be found in S1 Data.


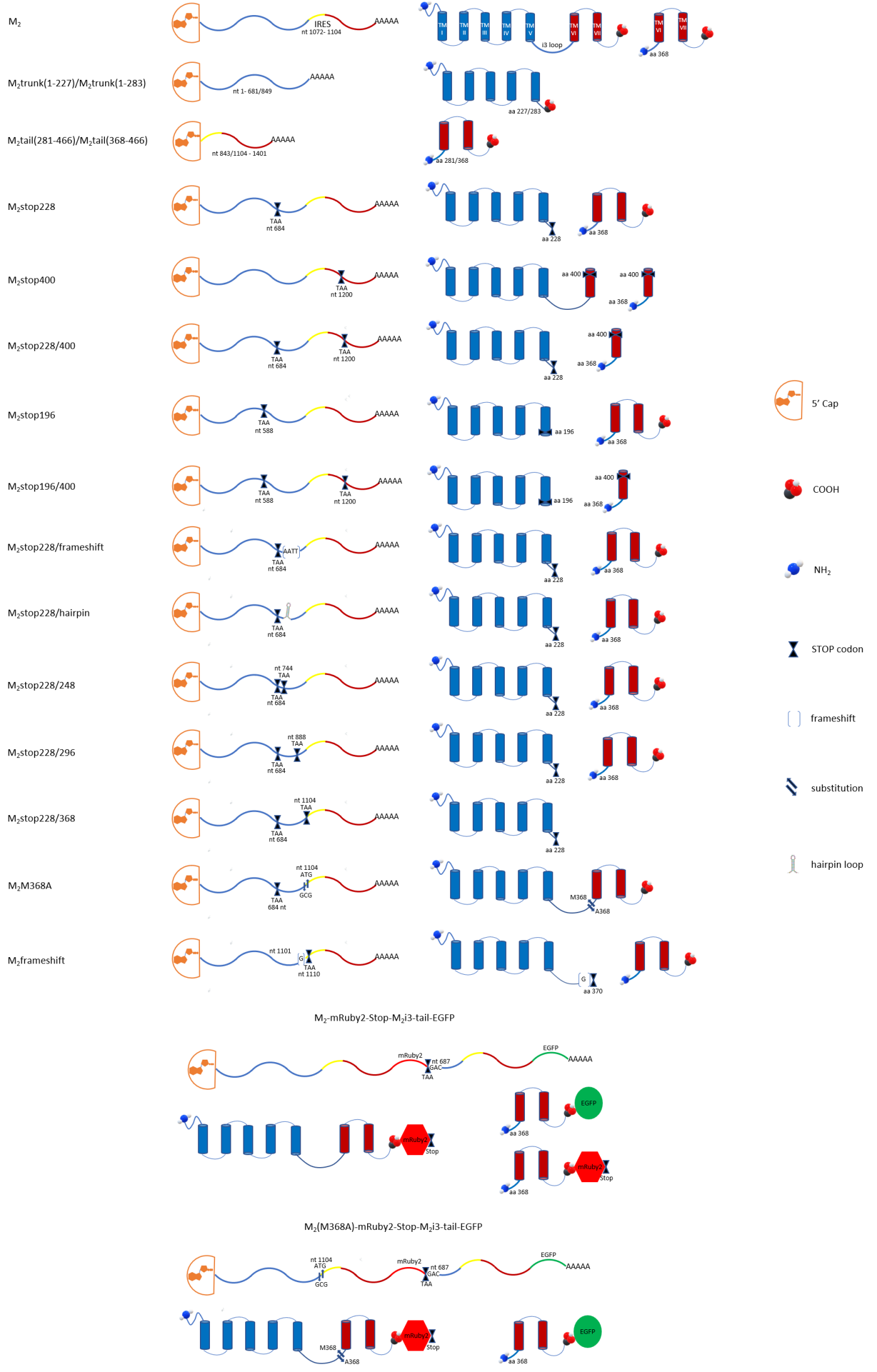


**Fig. S3.** **Schematic representation of wild type muscarinic M_2_ (human) receptors and derived mutants.**

Each construct was obtained as described under **Materials and Methods**. The left column indicates the mRNA product from each construct, the right column the expected protein product. For the last two constructs, above mRNA product, and below the expected protein product.


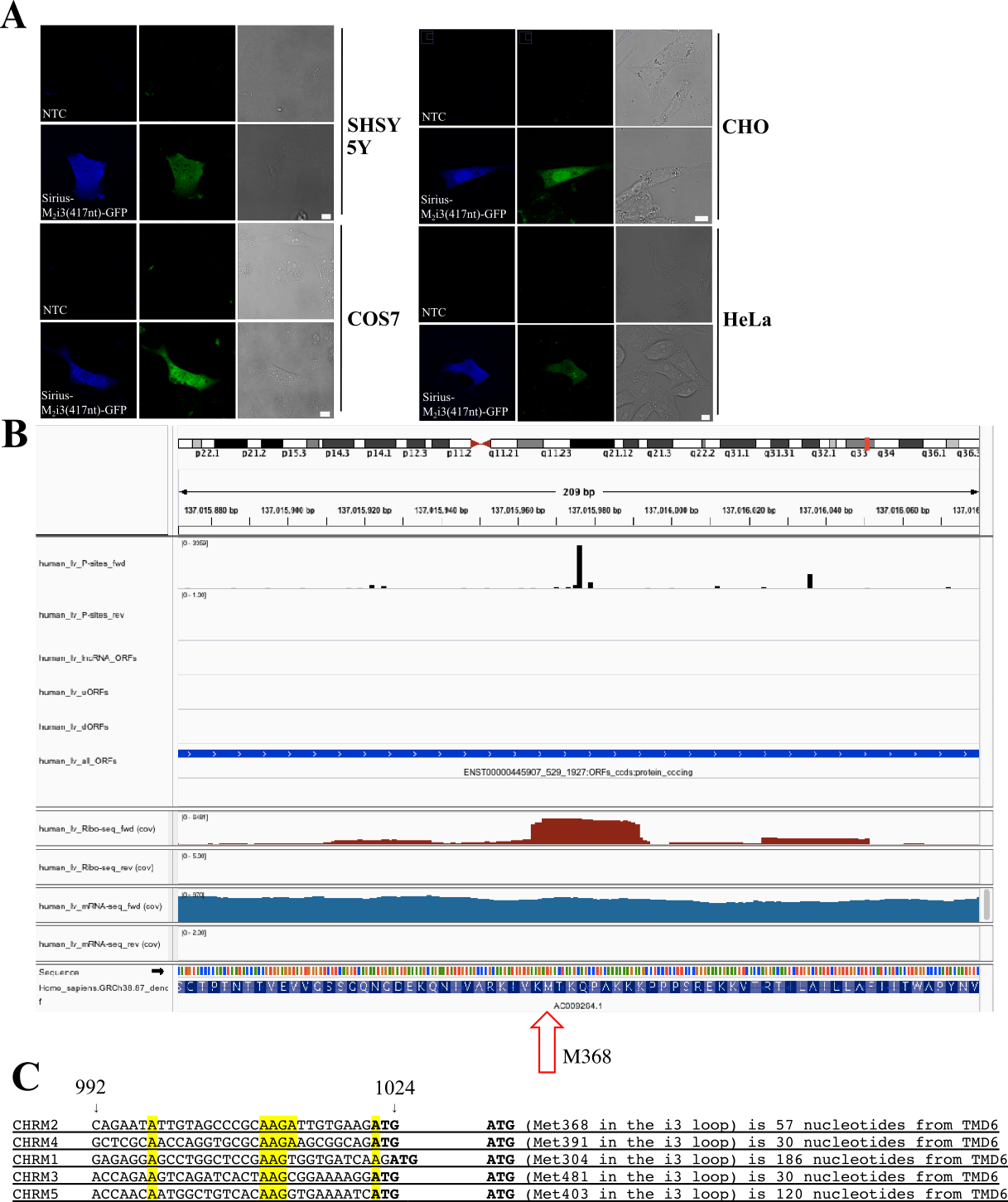


**Fig. S4.** **Expression of the bicistronic plasmid Sirius-M_2_i3(417n)-EGFP in four different cell lines, ribosome profiling data and sequence alignment**

**A,** Expression of Sirius was driven by the canonical scanning mechanism of translation initiation, while EGFP expression was driven by an IRES dependent mechanism. All Sirius-M_2_i3(417n)-EGFP transfected cells expressed both the Sirius and EGFP proteins. On average, 25 ± 4% of the COS-7 cells transfected with Sirius-M_2_i3(417n)-EGFP plasmid were blue fluorescence positive, while 17 ± 2% were green fluorescent positive, with only a few cells being only green. Excitation conducted at 405 nm (410-450 nm detection) for Sirius, and at 488 nm (500-550 nm detection) for GFP. Scale bars are 10 µm throughout. **B,** Ribosome profiling data from heart-specific transcriptomic databases[14], where the M_2_ receptor is highly expressed. When looking at left ventricle data, the ribosome coverage data is prominent in correspondence of the third i3loop in frame-methionine M368 (highlighted by the red arrow). In this case, also p-site hits are observed. **C,** Alignment of the nucleotide sequence 1072-1104 of the M_2_ i3 loop with analogous sequences of the other four muscarinic receptors. The criteria for the alignment are described in the supplementary data. In yellow are highlighted the conserved nucleotides. In bold are the in frame ATG codons. Next to each sequence is indicated the codon number of the in-frame ATG and its distance in nucleotides from the beginning of TMDVI.


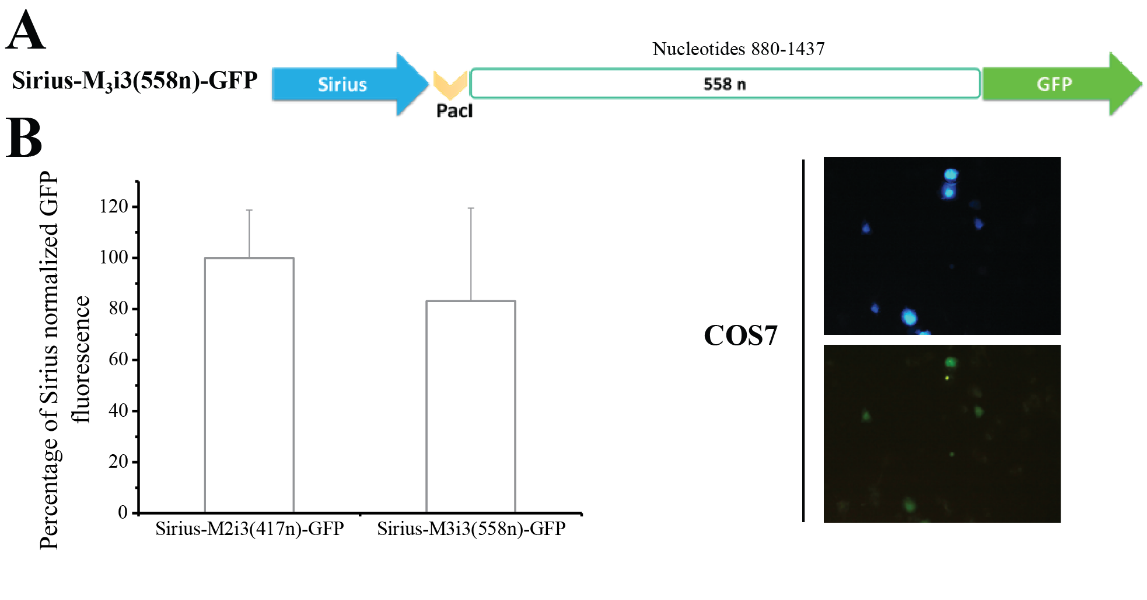


**Fig. S5. Expression of the bicistronic plasmid Sirius-M3i3(558n)-EGFP.**

**A,** Schematic representation of the bicistronic plasmid bearing the i3 loop of the rat muscarinic M_3_ receptor (558 nucleotides from nucleotide 880 to nucleotide 1437) between the coding regions of the Sirius and EGFP fluorescent proteins. **B,** EGFP expression in COS-7 cells transfected with the bicistronic plasmid Sirius-M_3_i3(558n)-EGFP. Source data for panel B can be found in S1 Data.


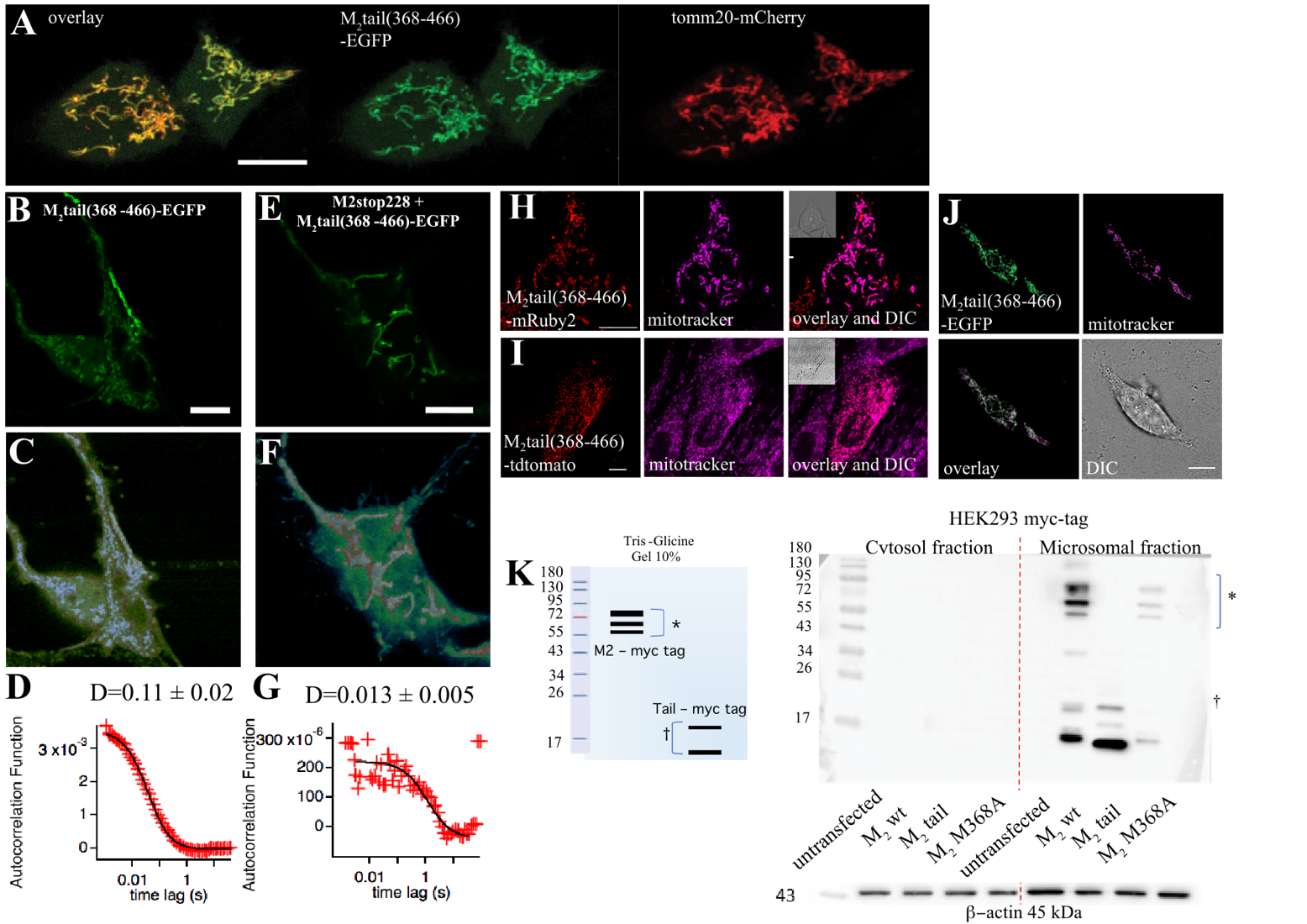


**Fig. S6.** **Subcellular localization of the M_2_tail(368-466)**

**A,** Confocal image displaying the colocalization (left of M_2_tail(368-466)-EGFP (green, middle) in HEK293 cells, co-expressed with the outer mitochondrial membrane marker Tomm20-mCherry (red, right). **B,** expression of M_2_tail-EGFP displaying marked mitochondria and cytosolic localization. **C,** Glasbey colorscale representation of panel (B) highlighting the low-intensity pixels. **D,** autocorrelation function of the M_2_tail(368-466)-EGFP diffusion taken at or in proximity of the basal membrane, yielding a mean diffusion coefficient of 0.11 µM_2_/s, incompatible with membrane diffusion. **E,** co-expression of M_2_stop228 and M_2_tail(368-466)-EGFP. M_2_stop228 is unlabeled. M_2_tail(368-466)-EGFP maintains the localization to the mitochondria observed in cells expressing M_2_tail(368-466)-EGFP alone, but it is also localized to the plasma membrane, visible in panel **F,** Glasbey colorscale representation of panel E highlighting the low-intensity pixels. **G,** autocorrelation function of the M_2_tail(368-466)-EGFP diffusion taken at or in proximity of the basal membrane, yielding a mean diffusion coefficient of 0.013 µM_2_/s, compatible with membrane diffusion. **H,** Cellular localization of M_2_tail-mRuby2, together with Mitotracker deep red staining of the mitochondrial network and corresponding DIC image. **I,** Cellular localization of M_2_tail-tdtomato and corresponding DIC image. **J,** Confocal sequential images displaying the localization of fluorescently labeled M_2_tail(368-466)-EGFP (green), together with the mitochondrial network (magenta), colocalization (white) and DIC image in COS-7 cells. All imaging panels originate from Laser Scanning Confocal Microscope sequential acquisitions, with laser lines 488 nm (EGFP) 561 nm (mRuby2, mCherry and tdtomato) and 633 nm (Mitotracker deep red), and corresponding emission filters in the ranges 520-600 nm, 570-620 nm and 640-750 nm. Scale bars 10 µm. **K**, Western blot (10% TRIS-Glycine PAA gel) of the cytosolic and microsomal fractions resulting from the mitochondria purification of lysates of HEK293 transfected with myc-tagged constructs, as displayed in **Fig. 3C**. Loading control involved immunoblotting for 𝛽-actin. Source data for panels D and G can be found in S1 Data.


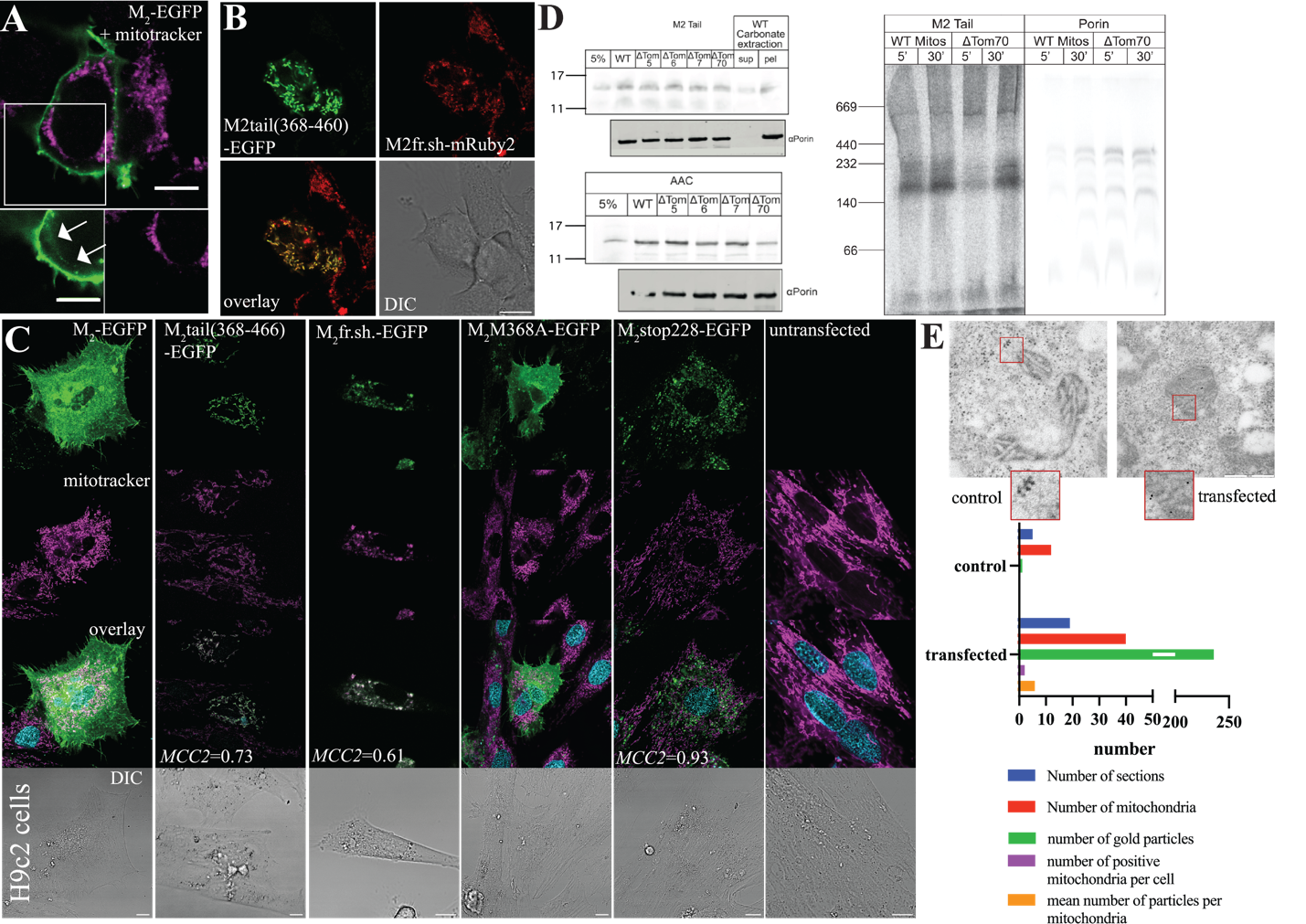


**Fig. S7.** **Mitochondrial localization of the M_2_tail fragment under endogenous IRES production and in vitro mitochondrial import of M_2_tail(368-466)**

**A,** Confocal image of M_2_-EGFP (green) expressed in conjunction with Mitotracker (magenta) in HEK293 cells. Separate panels below. **B,** Confocal image of HEK293 cell co-expressing M_2_tail(368-460) and M_2_fr.sh-mRuby2, together with the merged fluorescence image and DIC. **C,** Confocal micrographs of representative H9c2 cells expressing M_2_wt-EGFP, M_2_tail(368-466)-EGFP, M_2_fr.sh.M368A-EGFP, M_2_M368A-EGFP, M_2_stop228-EGFP and an untransfected control. Panels display, from top to bottom EGFP (green), Mitotracker (Magenta), overlay (EGFP, Mitotracker and, where present, Hoechst 33342 (Cyan)) and DIC (grays). Scale bars are 10 µm. Confocal sequential acquisitions were performed with 405 nm excitation and 420-460 nm detection (Hoechst), 488 nm excitation and 520-600 nm detection (EGFP), and 633 nm excitation and 650-750 nm detection (Mitotracker) using HyD detectors in Photon Counting Mode. Manders Correlation Coefficient M_2_ (fraction of green/M_2_tail features within magenta/mitochondria features) is indicated on the overlay images. **D,** 35S-labelled M_2_tail(368-466) or AAC (an integral internal mitochondrial membrane protein) were imported into isolated yeast mitochondria originating from different knock-out lines for outer membrane transporters (ΔTom). ΔTom5=68% of WT, ΔTom6=87% of WT, ΔTom7=87% of WT, ΔTom70=59% of WT . Samples were incubated for 30 minutes, and then washed in breaking buffer. After import, 100 µg of mitochondria were subject to Carbonate extraction to determine if M_2_tail(368-466) is integrated into a lipid bilayer (Pellet, 68%) or loosely associated/soluble (Supernatant, 32%). Samples were then loaded onto a 12% Tris-Tricine gel followed by semi-dry transfer and visualized via a phosphorimager (n=1). To test for equal mitochondrial loading anti-𝛼Porin was used via western blot. (right)35S-labelled M_2_tail(368-466) and 𝛼Porin were imported into isolated wild-type or ΔTom70 mitochondria for the indicated timepoints. Samples were then washed in breaking buffer and loaded on blue-native page followed by gel drying and visualized via a phosphorimager (n=1). **E,** Representative IEM micrographs of a control (left) and transfected (right) COS-7 cells. Scale bar is 1 µm. Statistics of IEM staining, both in transfected as well as control cells. The discernible 'dark spots' observed in the control, likely attributed to varied exposure settings during imaging, exhibit a noticeably larger size and less distinct shapes compared to the transfected samples, where circular, sharp, and intense gold nanoparticles are evident. Source data for panel E can be found in S1 Data.


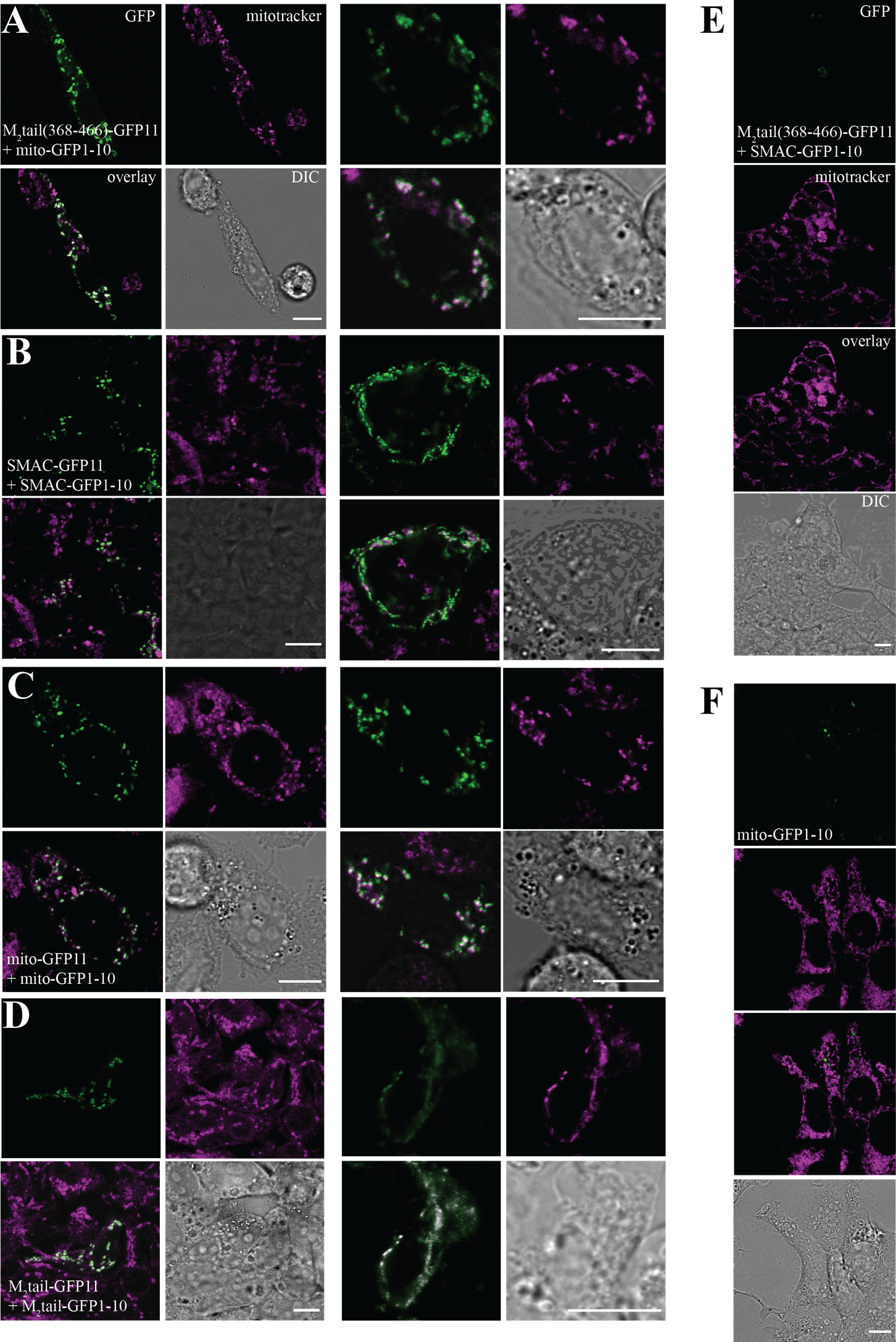


**Fig. S8.** **Cellular localization of split GFP constructs in HEK293 cells.**

**A,** Confocal images displaying the mitochondrial localization of M_2_tail(368-466)-GFP11 and mito-GFP1-10. **B,** co-expression of SMAC-GFP11 and SMAC-GFP1-10 (positive control) and corresponding Mitotracker deep red image. **C,** mito-GFP11 and mito-GFP1-10 (positive control). **D,** M_2_tail-GFP11 + M_2_tail-GFP1-10, with corresponding DIC image. Two representative experiments are displayed in panels A-D out of n=5 transfections for each condition. **E,** M_2_tail(368-466)-GFP11 and SMAC-GFP1-10. **F,** mito-GFP1-10 alone (negative control). One representative experiment is shown in panels E-F out of n04 transfections for each condition. EGFP (green) was excited at 488 nm and fluorescence collected between 500-600 nm. Mitotracker deep red (magenta) was excited at 633 nm and fluorescence collected between 650-750 nm. Scale bars are 10 µm.


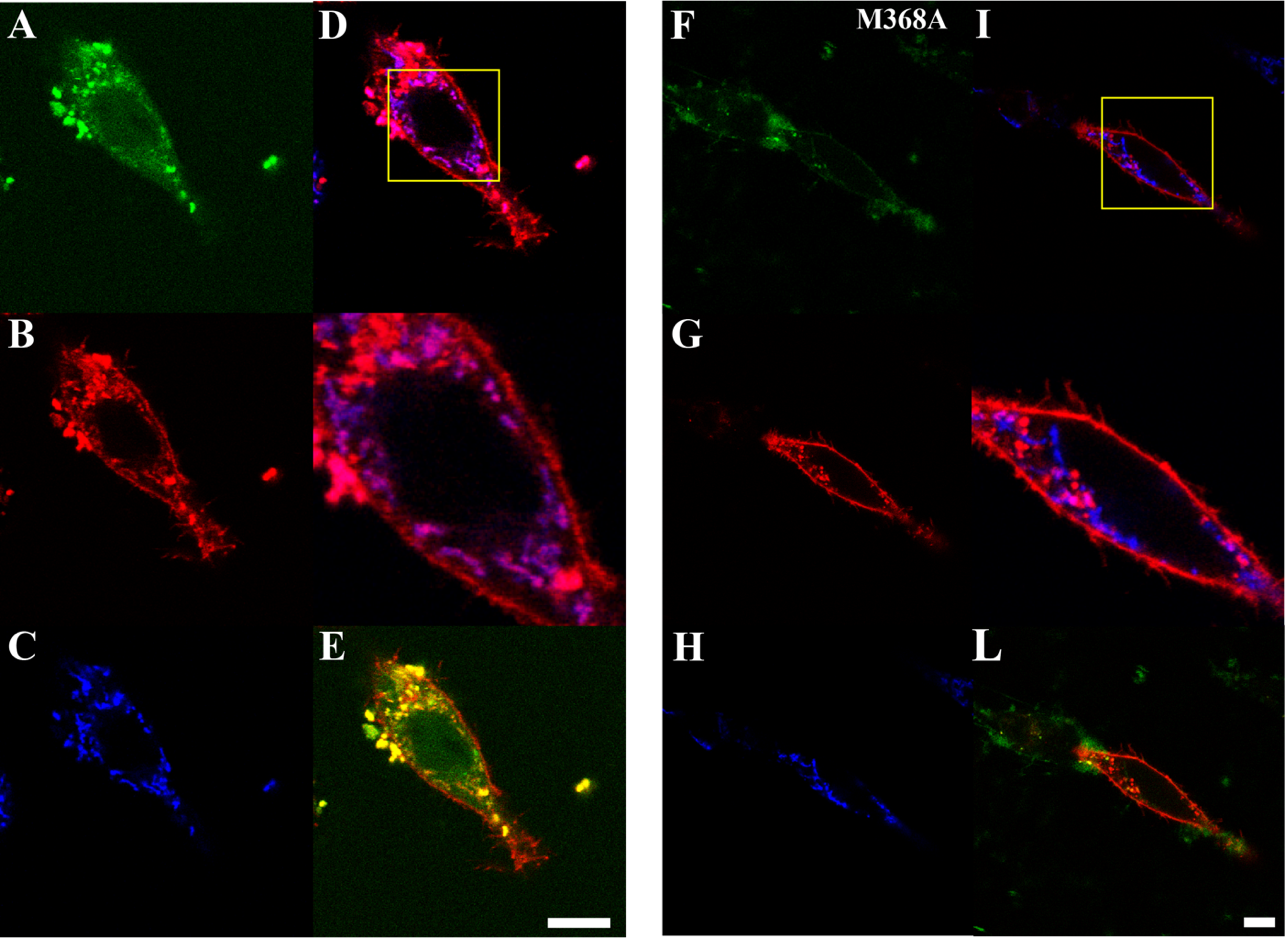


**Fig. S9.** **Mitochondrial localization of the M_2_tail(368-466) fragment upon IRES production.**

**A-E**, Mitochondrial localization of the IRES mediated C-terminal fragment is enhanced upon cellular stress. HEK293 cells transfected with the super construct M_2_-mRuby2-STOP-M_2_-i3-tail-EGFP display increased localization of the EGFP labelled portion to the mitochondria, upon serum starvation for 2 hours and incubation of the cells in HBSS buffer, in presence of Mitotracker: A, is IRES-driven M_2_tail(368-466)-EGFP. **B**, Cap dependent M_2_-mRuby2 + IRES-driven M_2_tail(368-466)-mRuby2, both derived from the M_2_-mRuby2 gene. **C**, Mitotracker Deep Red **D**, Overlay of Mitotracker Deep Red and mRuby channel (colocalization in magenta). Zoom-in of yellow square immediately below the panel. **E**, Overlay between GFP and mRuby2 (colocalization in yellow). **F-L**, Localization of the mutant construct M_2_(M368A)-mRuby2-STOP-M_2_-i3-tail-EGFP in HEK293 cells after 2 hours incubation in HBSS buffer. The same legend as in **A-E** applies. Bottom: Schematic structure of the mega constructs M_2_(M368A)-mRuby2-STOP-M_2_-i3-tail-EGFP. This construct is similar to the M_2_-mRuby2-STOP-M_2_-i3-tail-EGFP construct but the methionine 368 in the M_2_ sequence has been replaced with alanine. The asterisks represent the stop codons at the end of mRuby2 and EGFP. EGFP was excited at 488 nm and fluorescence collected between 500-600 nm; mRuby2 was excited at 561 nm and fluorescence collected between 580-620 nm. Mitotracker deep red was excited at 633 nm and fluorescence collected between 650-750 nm. Scale bars are 10 µm.


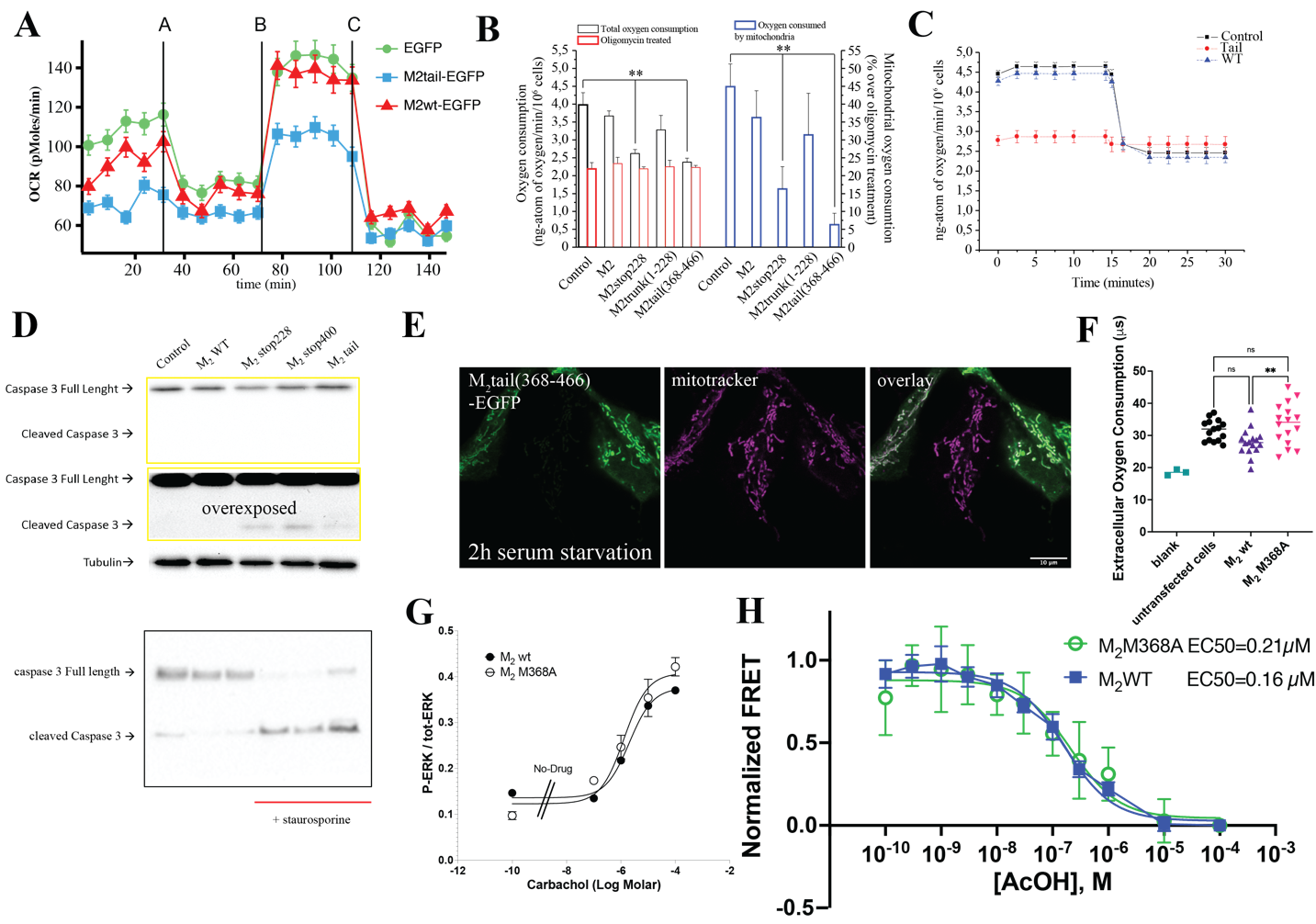


**Fig. S10.** **Supplemental data on the effect of the M_2_tail(368-466) fragment on mitochondrial function and cell metabolism.**

**A,** Representative experiment of Seahorse assay showing the time course of oxygen consumption inhibition by M_2_tail(368-466)-EGFP and wild type M_2_-EGFP, compared to the same experiments performed in control cells transfected with EGFP. The rate of ATP-linked respiration measured after oligomycin administration is decreased in M_2_tail and slightly decreased in M_2_ cells as well as compared to the controls. FCCP, an ionophore, was added to each cell type to investigate the cellular bioenergetic reserves that seem to be slightly reduced only in the M_2_ tail cells. **B,** Oxygen consumption measured by a Clark type electrode-based-polarographic method at a constant temperature of 37 °C. After 15 minutes of basal respiration, the ATP synthase inhibitor oligomycin was added at a concentration of 17 nM. Oxygen consumption in the absence and presence of oligomycin is represented by black and red bars, respectively. The blue bars represent the percentage of mitochondrial oxygen consumption (total minus oligomycin treated) in cells transfected with each construct. Significance values reported in the graphs were determined by a one-tailed Student t-test, p-values: ** 0.001<p<0.01. **C,** is a representative experiment showing the time course of inhibition of oxygen consumption by M_2_tail(368-466), compared to the same experiments performed in control cells and cells transfected with the M_2_ wild type construct, upon addition of oligomycin. Results are given as ng-atom of oxygen/min/106 cells. **D,** Western blot of caspase-3 cleavage in COS-7 cells transfected with the indicated M_2_ receptor mutants 48h after transfection (top of panel, normal contrast; bottom of panel, increased contrast). Below, as a positive cleavage control, the blot for caspase-3 with and without Staurosporine incubation. **E,** Confocal sequential images of M_2_tail(368-466)-EGFP (green), Mitotracker (magenta) and overlay upon 2h of serum starvation. Scale Bars are 10 μm. **F,** Extracellular oxygen consumption assay of HEK293 cells transfected with the indicated M_2_ receptor mutants using a fluorogenic dye quenched by Oxygen. Blank indicates the dye lifetime measured in wells containing the dye, but no cells. **G,** Normalized dose response to Carbachol stimulation of HeLa cells, comparing M_2_ wild type to the M_2_ M368A mutant. **H,** Normalized dose response curves based on FRET Gi biosensor[10], reflecting Galphai3 activation by two M_2_ receptor mutants (M_2_ wild type n=7 transfections, M_2_ M368A n=3 transfections) stimulated by acethylcholine (AcOH) in HEK293 cells. Source data for panels A,B, C, F, G and H can be found in S1 Data.

| Receptor | Bmax (fmol/mg) | Receptor | Bmax (fmol/mg) |
| --- | --- | --- | --- |
| M_2_stop228 | 45.3 ± 2.4 | M_2_stop228/stop368 | N.B. |
| M_2_stop196 | N.B. | M_2_stop228/stop368 + M_2_tail(281-466) | 370± 20 |
| M_2_stop400 | N.B. | M_2_stop228/stop400 | N.B. |
| M_2_stop196+ M_2_trunk(1-283) | 47 ± 3 | M_2_stop228/stop400+ M_2_tail(281-466) | 380 ± 20 |
| M_2_stop400+ M_2_tail(281-466) | 400 ± 30 | M_2_stop196/stop400+ M_2_trunk(1-283) | N.B. |
| M_2_stop196/stop400 | N.B. | M_2_stop196/stop400+ M_2_tail(281-466) | N.B. |
| M_2_stop228/stop248 | 49 ± 5 | M_2_stop228/fr.sh. | 47 ± 3 |
| M_2_stop228/stop296 | 38 ± 3 | M_2_stop228/Hairpin | 37 ± 2 |

**Table S1.** Ligand binding and functional properties of M_2_ receptor mutants with single and double stop codons. Number of [^3^H]NMS binding sites for the indicated mutants. Untransfected COS-7 cells did not show any [^3^H]NMS specific binding. N.B. = no specific [^3^H]NMS binding.

**Table S2.** Radioligand binding and activation properties of M_3_ receptor mutants. Binding characteristics and stimulation of phosphatidylinositol accumulation by M_3_, M_3_stop273 and the co-transfected M_3_trunk(1-272) and M_3_tail(M-388-586). Untransfected COS-7 cells did not show any [^3^H]NMS specific binding. N.B. = no specific [^3^H]NMS binding.

| Binding data | | | | Phosphatidylinositol accumulation assay | |
| --- | --- | --- | --- | --- | --- |
| Receptor | Bmax (fmol/mg) | [^3^H]NMS KD (pM) | Carbachol IC50 (µM) | Carbachol EC50 (µM) | Maximum increase in IP1 level above baseline (%) |
| M_3_ | 1120 ± 71 | 29 ± 2 | 59 ± 4 | 4.2 ± 0.5 | 183 ± 15 |
| M_3_trunk(1-272) + M_3_tail(388-589) | 114 ± 17 | 23 ± 2 | 48 ± 5 | 1.4 ± 0.1 | 126 ± 12 |
| M_3_stop 273 | 52.3 ± 0.5 | 31.3 ± 2.8 | 56 ± 2 | 1.1 ± 0.1 | 89 ± 13 |

**Table S3**. Ligand binding of M_3_ receptor mutants with single and double stop codons. Number of [^3^H]NMS binding sites in COS-7 cells transiently transfected with the indicated M_3_ receptor mutants with single and double stop codons.

| Receptor | Bmax (fmol/mg) | Receptor | Bmax (fmol/mg) |
| --- | --- | --- | --- |
| M_3_stop273 | 54 ± 1 | M_3_stop273/stop503 | N.B. |
| M_3_stop240 | N.B. | M_3_stop273/stop503 + M_3_tail(388-589) | 120± 15 |
| M_3_stop503 | N.B. | M_3_stop240/stop503+ M_3_trunk(1-272) | N.B. |
| M_3_stop240+ M_3_trunk(1-272) | 44 ± 3 | M_3_stop240/stop503+ M_3_tail(388-589) | N.B. |
| M_3_stop503+ M_3_tail(388-589) | 135 ± 12 | M_3_stop273/fr.sh. | 120 ± 4 |
| M_3_stop240/stop503 | N.B. | M_3_stop273/Hairpin | 30 ± 2 |

**References**

1. Maggio R, Barbier P, Colelli A, Salvadori F, Demontis G, Corsini GU. G protein-linked receptors: pharmacological evidence for the formation of heterodimers. The Journal of pharmacology and experimental therapeutics. 1999;291(1):251-7.

2. Rauth S, Song KY, Ayares D, Wallace L, Moore PD, Kucherlapati R. Transfection and homologous recombination involving single-stranded DNA substrates in mammalian cells and nuclear extracts. Proceedings of the National Academy of Sciences. 1986;83(15):5587-91. doi: 10.1073/pnas.83.15.5587.

3. Schueren F, Lingner T, George R, Hofhuis J, Dickel C, Gärtner J, et al. Peroxisomal lactate dehydrogenase is generated by translational readthrough in mammals. eLife. 2014;3. doi: 10.7554/eLife.03640.

4. Loughran G, Chou M-Y, Ivanov IP, Jungreis I, Kellis M, Kiran AM, et al. Evidence of efficient stop codon readthrough in four mammalian genes. Nucleic Acids Research. 2014;42(14):8928-38. doi: 10.1093/nar/gku608.

5. Powell ML, Napthine S, Jackson RJ, Brierley I, Brown TDK. Characterization of the termination–reinitiation strategy employed in the expression of influenza B virus BM2 protein. RNA. 2008;14(11):2394-406. doi: 10.1261/rna.1231008.

6. Kozak M. Constraints on reinitiation of translation in mammals. Nucleic Acids Research. 2001;29(24):5226-32. doi: 10.1093/nar/29.24.5226.

7. Maggio R, Vogel Z, Wess J. Reconstitution of functional muscarinic receptors by co-expression of amino- and carboxyl-terminal receptor fragments. FEBS Letters. 1993;319(1-2):195-200. doi: 10.1016/0014-5793(93)80066-4.

8. Kozak M. Circumstances and mechanisms of inhibition of translation by secondary structure in eucaryotic mRNAs. Molecular and Cellular Biology. 1989;9(11):5134-42. doi: 10.1128/mcb.9.11.5134-5142.1989.

9. Zhang J, Nuebel E, Wisidagama DRR, Setoguchi K, Hong JS, Van Horn CM, et al. Measuring energy metabolism in cultured cells, including human pluripotent stem cells and differentiated cells. Nature Protocols. 2012;7(6):1068-85. doi: 10.1038/nprot.2012.048.

10. Van Unen J, Stumpf AD, Schmid B, Reinhard NR, Hordijk PL, Hoffmann C, et al. A new generation of FRET sensors for robust measurement of Gαi1, Gαi2 and Gαi3 activation kinetics in single cells. PLoS ONE. 2016;11(1). doi: 10.1371/journal.pone.0146789.

11. Kritsiligkou P, Chatzi A, Charalampous G, Mironov A, Grant CM, Tokatlidis K. Unconventional Targeting of a Thiol Peroxidase to the Mitochondrial Intermembrane Space Facilitates Oxidative Protein Folding. Cell Reports. 2017;18(11):2729-41. doi: 10.1016/j.celrep.2017.02.053.

12. Bonner TI, Buckley NJ, Young AC, Brann MR. Identification of a family of muscarinic acetylcholine receptor genes. Science. 1987;237(4814):527-32. Epub 1987/07/31. doi: 10.1126/science.3037705. PubMed PMID: 3037705.

13. Pándy-Szekeres G, Munk C, Tsonkov TM, Mordalski S, Harpsøe K, Hauser AS, et al. GPCRdb in 2018: Adding GPCR structure models and ligands. Nucleic Acids Research. 2018;46(D1). doi: 10.1093/nar/gkx1109.

14. van Heesch S, Witte F, Schneider-Lunitz V, Schulz JF, Adami E, Faber AB, et al. The Translational Landscape of the Human Heart. Cell. 2019;178(1):242-60.e29. doi: 10.1016/j.cell.2019.05.010.
