# Supplementary material for: The C-terminus of the prototypical M2 muscarinic receptor localizes to the mitochondria and regulates cell respiration under stress conditions
Source: PLoS Biol. 2024 Apr 29;22(4):e3002582. doi: 10.1371/journal.pbio.3002582 (PMC11093360; doi:10.1371/journal.pbio.3002582)
Supplement: S1 Methods — (DOCX) [file pbio.3002582.s017.docx]

Supplementary Methods for

**The C-terminus of the prototypical M2 muscarinic receptor localizes to the mitochondria and regulates cell respiration under stress conditions**

Irene Fasciani^1†^, Francesco Petragnano^1†^, Ziming Wang^2^, Ruairidh Edwards^3^, Narasimha Telugu^2^, Ilaria Pietrantoni^1^, Ulrike Zabel^4^, Henrik Zauber^2^, Marlies Grieben^2^, Maria E. Terzenidou^3^, Jacopo Di Gregorio^1^, Cristina Pellegrini^1^, Silvano Santini Jr^5^, Anna R. Taddei^6^, Bärbel Pohl^2^, Stefano Aringhieri^7^, Marco Carli^7^, Gabriella Aloisi^1^, Francesco Marampon^8^, Eve Charlesworth^9^, Alexandra Roman^2^, Sebastian Diecke^2^, Vincenzo Flati^1^, Franco Giorgi^7^, Fernanda Amicarelli^5^, Andrew B. Tobin^3^, Marco Scarselli^7^, Kostas Tokatlidis^3^, Mario Rossi^1^, Martin J. Lohse^2,4,10^*, Paolo Annibale^2,4,9^*, Roberto Maggio^1^*,

**This PDF file includes:**

Supplementary Methods

Cloning and molecular biology

The two expression plasmids referred as M_2_trunk(1-283) (containing transmembrane domains I-V and the N-terminal portion of the third cytoplasmic loop) and M_2_tail(281-466) (containing transmembrane domains VI and VII, and the C-terminal portion of the third cytoplasmic loop) were described previously[7].

**Constructs for radioligand binding and immunoblotting**

M_2_stop228 – This plasmid was created by substituting codon 228 (CAA) with a stop codon (TAA) at the N-terminal of the i3 loop of the wild type M_2_ receptor.

M_2_stop228/stop400 – This plasmid was created by inserting an additional stop codon – TGG was substituted with TAA – at the position codon 400 of the TM region VI of the M_2_stop228 mutant.

M_2_stop400 – This plasmid was created by substituting codon 400 (TGG) with a stop codon (TAA) in the TM region VI of the wild type M_2_ receptor.

M_2_stop196 – This plasmid was created by substituting codon 196 (TAT) with a stop codon (TAA) in the TM region V of the wild type M_2_ receptor.

M_2_stop196/stop400 – This plasmid was created by inserting an additional stop codon in the TM region VI of the M_2_stop196 mutant, where TGG was substituted with TAA at position of the codon 400.

M_2_stop228/fr.sh. – This plasmid was created by inserting four bases (AATT) fifteen nucleotides downstream of the stop codon to create a shift in the correct reading frame of the M_2_stop228 mutant.

M_2_stop228/hairpin – This construct was created by inserting a 42 bases long sequence (AGGGGCGCGTGGTGGCGGCTGCAGCCGCCACCACGCGCCCC), fifteen nucleotides downstream of the stop codon of the M_2_stop228 mutant. Upon transcription, this palindromic sequence has been shown to form a hairpin structure with a ΔG value of −64 kcal/mol that blocks effectively ribosome scanning[6, 8].

M_2_stop228/stop248, M_2_stop228/stop296 and M_2_stop228/stop368 – These three constructs were obtained by substituting the three in-frame ATG codons (1. ATG 248, 2. ATG 296 and 3. ATG 368) with the stop codon TAA downstream of the stop228 of the M_2_stop228 mutant.

M_2_trunk(1-228) – This plasmid was created by substituting codon 228 (CAA) with a stop codon (TAA) at the N-terminal of the i3 loop of the wild type M_2_ receptor followed by removal of the downstream sequence.

M_2_tail(368-466) – This construct was created by removing all bases of the M_2_ receptor sequence up to codon 368 (ATG) of the C-terminal of the i3 loop. The resulting plasmid encodes for a polypeptide fragment that contains the trans-membrane domains VI and VII along with the C-terminal portion of the third cytoplasmic loop.

M_2_(M368A) – In this construct the third in-frame methionine of the i3-loop of the M_2_ muscarinic receptor (ATG) was mutated to alanine (GCG).

M_2_-Myc, M_2_stop228-Myc, M_2_stopM368A-Myc, M_2_tail(368-466)-Myc and M_2_stop40-Myc – M_2_-Myc was purchased from OriGene. This plasmid encodes the human M_2_ receptor with a Myc (tag at the C-terminus of the protein. M_2_stop228-Myc was obtained by replacing the 0.9 Kb BmtI-PspOMI fragment of the M_2_stop228 mutant with the corresponding fragment of M_2_-Myc. M_2_stop400-Myc was obtained by substituting the codon 400 (TGG) with a stop codon (TAA) of the M_2_-Myc. All the constructs have an additional DDK tag after the C-Myc. Subsequently, all C-Myc constructs were extracted by PCR and subcloned into a bicistrionic pVitro2-MCS plasmid (Invivogen), allowing for the expression of a reporter gene (in our case the red fluorescent protein mRuby2) after an IRES sequence, in order to check for transfection efficiency.

**Generation of bicistronic constructs expressing Sirius and EGFP proteins** (**Fig. 2A**)

Sirius[PacI-M_2_i3(685-1101)]EGFP – This bicistronic construct was created by inserting the i3 loop of the muscarinic M_2_ receptor, from nucleotide 685 to nucleotide 1101, between the ultramarine fluorescent protein (Sirius) and the green fluorescent protein (EGFP). Eight nucleotides corresponding to the recognition site of the PacI enzyme were inserted between Sirius and the i3 loop of the wild type M_2_ receptor to alter the reading frame downstream of the Sirius stop codon. Hereafter this plasmid is referred to as Sirius-M_2_i3(417n)-EGFP.

Sirius[PacI-M_2_i3(685-699)-Hairpin-G]EGFP – This bicistronic construct was created by inserting a 42 nucleotides hairpin loop (see above) between Sirius and the EGFP fluorescent protein. The hairpin loop was spaced from the Sirius stop codon by inserting a PacI recognition sequence and 15 nucleotides of the M_2_ i3 loop sequence comprised between nucleotide 685 and nucleotide 699. A G nucleotide was also inserted following the 42 nucleotides of the hairpin loop and upstream of the initial ATG triplet of the EGFP to restore a correct reading frame. Throughout the text we referred to this plasmid as Sirius-H-EGFP.

Sirius[PacI-M_2_i3(685-699)-Hairpin-G-PacI-M_2_i3(685-1101)]EGFP – This bicistronic construct was created by adding 417 nucleotides of the i3 loop of M_2_ from nucleotide 685 to nucleotide 1101 to the plasmid Sirius[PacI-M_2_i3(685-699)-Hairpin-G]EGFP. An additional PacI restriction site was also inserted upstream of this segment of the i3 loop. Throughout the text we referred to this plasmid as Sirius-H-M_2_i3(417n)-EGFP.

Sirius[PacI-M_2_i3(685-699)-Hairpin-G-PacI-M_2_i3(685-699/1072-1101)]EGFP – This bicistronic construct was created by deleting from the plasmid Sirius[PacI-M_2_i3(685-699)-Hirpin-G-PacI-M_2_i3(685-1101)]EGFP 372 nucleotides of the M_2_ i3 loop, from nucleotide 700 to nucleotide 1071. Throughout the text we referred to this plasmid as Sirius-H-M_2_i3(15n/30n)-EGFP.

Sirius[PacI-M_2_i3(685-699)-Hirpin-G-PacI-M_2_i3(685-699)]EGFP – This bicistronic construct was created by adding 15 nucleotides of the i3 loop of M_2_ from nucleotide 685 to nucleotide 699 to the plasmid Sirius[PacI-M_2_i3(685-699)-Hirpin-G]EGFP. An additional PacI restriction site was also inserted upstream of this short segment of the i3 loop. Throughout the text we will refer to this plasmid as Sirius-H-M_2_i3(15n)-EGFP.

Sirius[PacI-M_2_i3(685-699)-Hirpin-G-PacI-M_2_i3(685-1071)]EGFP – This bicistronic construct was created by adding 387 nucleotides of the i3 loop of M_2_ form nucleotide 685 to nucleotide 1071 to the plasmid Sirius[PacI-M_2_i3(685-699)-Hirpin-G]EGFP. An additional PacI restriction site was also inserted upstream this long segment of the i3 loop. Throughout the text we will refer to this plasmid as Sirius-H-M_2_i3(387n)-EGFP.

Mutants of the Sirius-H-M_2_i3(15n/30n)-EGFP plasmid – Based on the sequence alignment of amine and adenosine GPCRs, several conserved nucleotides were mutated in order to define the putative IRES sequence. These mutants are reported in **Fig. 2A**.

The fluorescence intensities of the two spectral bands were quantified by the use of a fluorometer and the EGFP intensity values normalized to those of Sirius.

**Constructs for fluorescence microscopy**

M_2_-EGFP – The wild type M_2_ muscarinic receptor was cloned into the pEGFP-N1 expression cassette, between the restriction sites HindIII and XbaI.

M_2_-mRuby2 – The wild type M_2_ muscarinic receptor was cloned into the mRuby2 vector (Addgene plasmid #40260).

M_2_tail(368-466)-EGFP – The M_2_tail(368-466)-EGFP was constructed so that the receptor protein would start from the third in-frame methionine of the M_2_ i3 loop (M368). The EGFP gene was fused C-terminally following a short restriction site sequence (TCTAGA) of the XbaI enzyme.

M_2_tail(368-466)-mRuby2 – The M_2_tail(368-466)-mRuby2 was constructed so that the receptor protein would start from the third in-frame methionine of the M_2_ i3 loop (M368). The mRuby2 gene was fused C-terminally following a short restriction site sequence (TCTAGA) of the XbaI enzyme.

M_2_-mRuby2-STOP-M_2_-i3-tail-EGFP – This mega construct was obtained by fusing two preceding constructs, M_2_-mRuby2 and M_2_tail(368-466)-EGFP, in a unique plasmid, but the M_2_tail started at codon 229 (M_2_tail(229-466)-EGFP). After the stop codon of mRuby2 and before the codon 229 of M_2_tail was inserted a short restriction site sequence (TCCGGA) of the BspEI enzyme (**Fig. 3D**).

M_2_(M368A)-mRuby2-STOP-M_2_-i3-tail-EGFP – This mega construct is analogous to M_2_-mRuby2-STOP-M_2_-i3-tail-EGFP, but the third in-frame methionine of the i3-loop of the M_2_ muscarinic receptor was mutated to alanine (M368A) (**Fig. S3**).

M_2_fr.sh-EGFP – A single base insertion (G) upstream of nucleotide 1102 of the wild type M_2_ receptor, just before the third in-frame methionine of the i3-loop (M368), induces a frame shift that, following 2 aminoacids, generates a stop codon (TAA) in the amino acid position 370 of the new reading frame. The construct is then fused to EGFP.

M_2_fr.sh-mRuby2 – As above, but the EGFP is replaced by a mRuby2

M_2_-hairpin-EGFP – The 3^rd^ i3 loop of the M_2_ receptor (between resides 228 and 338) was replaced by the sequence aggggcgcgtggtggcggctgcagccgccaccacgcgcccct, generating a mRNA hairpin, as discussed above before the 30 nt sequence upstream of M368.

M_2_-GFP11 – A short peptide 16 aminoacids long from the GFP protein, GFP11, was fused to the C-terminus of the muscarinic M_2_ receptor. GFP11 was custom syntethised and inserted as a linker between the XbaI and XhoI restriction sites in the plasmid backbone of M_2_-EGFP.

Mito-GFP1-10 – The mitochondrial targeting sequence of the cytochrome c oxidase subunit 8A (COX8A) was fused to the N-terminal of GFP1-10 fragment of GFP. GFP1-10 was purchased from Addgene as Addgene plasmid 70219.

Mito-GFP11 – The mitochondrial targeting sequence of COX8A was fused to GFP11 fragment. Mito sequence was obtained from Addgene plasmid 23348.

SMAC-GFP1-10 – The mitochondrial targeting sequence of the SMAC protein was fused to the N-terminal of GFP1-10 fragment. SMAC was obtained from Addgene plasmid 40881.

SMAC-GFP11 – The mitochondrial targeting sequence of the SMAC protein was fused to GFP11 fragment.

M_2_tail(368-466)-GFP1-10 – The GFP1-10 fragment was fused to the C-terminus of the muscarinic M_2_tail(368-466) receptor fragment.

M_2_tail(368-466)-GFP11 – The GFP11 fragment was fused to the C-terminus of the muscarinic M_2_tail(368-466) receptor fragment.

SMAC-mCitrine – mCitrine was fused to the C-terminus of the mitochondrial targeting sequence of SMAC using restriction enzymes: BamHI and NotI. SMAC sequence was obtained from Addgene plasmid 40881. mCitrine originates from plasmid FLAGmMOR-mCitirine, in pcDNA3.1.

Mito-mCitrine - mCitrine was fused to the C-terminus of the mitochondrial targeting sequence of COX8A using restriction enzymes: BamHI and NotI. Cox8A (Mito) sequence was obtained from Addgene plasmid 23348.

CV-mCitrine - mCitrine was fused to the C-terminus of the mitochondrial targeting sequence of Complex V (CV) using restriction enzymes: EcoRI and NotI. CV sequence was obtained from Addgene plasmid 213884.

M_2_tail-mTurquoise2 - mTurquoise2 was fused to the C-terminus of M_2_tail using the restriction enzymes: XbaI and NotI. mTuquoise2 originates from the plasmid pc-FLAG-mTq2-b2AR, in pcDNA3.1.

**Constructs for in-vitro mitochondrial import assays**

PSP64-M_2_tail(368-466)-5Met - The vector backbone PSP64 (courtesy of Ruaridh Edwards). The following primers: FWD: ATGATGTAAtctagaggaggcggacgc, REV: CATCATCATccttgtagcgcctatgttcttataatg

were used to add five methionines to the C-terminal domain of the M_2_tail(368-466)-GFP11 template (see above) using Q5® Site-Directed Mutagenesis Kit (New England Biolabs® Inc.). The construct was then extracted with appropriate restriction enzymes (HindIII and XbaI) and ligated into the vector backbone PSP64 (courtesy of Ruaridh Edwards).

PSP64-M_2_R-5Met – The following primers: FWD: ATGATGTAAtctagaggaggcggacgc REV: CATCATCATccttgtagcgcctatgttcttataatg were used to add five methionines to the C-terminal domain of to the M_2_-GFP11 template (see above) using Q5® Site-Directed Mutagenesis Kit (New England Biolabs® Inc.). The construct was then extracted with appropriate restriction enzymes (HindIII and XbaI) and ligated into the vector backbone PSP64.

All constructs were sequenced either with a Genetic Analyzer 3500 (Applied Biosystem®) or by a professional sequencing service (LGC Genomics, Berlin).

**Immunoblot of muscarinic M_2_ receptor mutants transfected in HEK293 cells**

Cells were seeded in 10 cm plates and transfected after 24h according to manufacturer protocols (Effectene, Qiagen) using 2 µg of plasmid DNA. 48h after transfection cells were washed (2x) in ice cold PBS. 200 µL per plate of ice-cold lysis buffer were added. Lysis buffer was a RIPA buffer: 50 mM Tris-HCL pH 8, 150 mM NaCl, 1% TRITON, 0.5% sodium deoxycholate and 0.1% SDS. The following compounds were added to the buffer to a final 1x dilution: 100x Halt Protease Inhibitor Cocktail (Thermo Fisher), 100x 0.5 M EDTA and (10x) 10 mM PMSF. Cells were then scraped and the cell lysate transferred to pre-cooled 1.5 mL Eppendorf tubes. The tubes were placed in a Thermomixer at 4 °C, 300 rpm for 30 minutes. Then the Eppendorf tubes were centrifuged for 30 minutes at 14000 rpm at 4 °C. The supernatant was then transferred to new Eppendorf tubes. Protein concentration in the cell lysates was quantified by a BCA assay (Pierce BCA Protein Assay Kit, from Thermo Fisher) according to manufacturer instructions.

Cell lysates were loaded into 10% polyacrylamide gels. Cell lysates were loaded using Laemmli Buffer 2x (Sigma Aldrich) in equal mass. As a reference marker we loaded 5 uL of PageRuler Prestained Protein Ladder (Thermo Fisher). Gels were mounted into a Mini Protean Tetra Cell kit (BioRad) using 1x SDS running buffer. Gels were ran using a constant voltage of 90V for approximately 15 minutes, and afterwards at 130V for 45 minutes. Gels were transferred to PVDF membranes. Wet transfer was achieved by using the Mini Trans Blot Module (BioRad) in Wet Transfer Buffer (25 mM Tris pH 8.3, 192 mM Glycine and 20% MetOH) at 350 mA for 90 minutes. The immunoblotted membranes were then blocked in 5% Milk in TBS-T for 1 hour at room temperature. The membrane was then incubated overnight at 4 °C with primary antibody solution using the following concentrations depending on the antibody used: 1:1000 anti β-actin (13E5) rabbit primary antibody (Cell Signaling), 1:1000 anti myc-Tag (9B11) mouse primary antibody (Cell Signaling). After overnight incubation, the membranes were washed 3x 10 minutes in TBS-T and incubated at RT for 1 h with a secondary antibody (1:10000 anti-mouse IgG/(anti rabbit) HRP-linked antibody from Cell Signaling).

After the incubation with the secondary antibody, the membrane was washed 3x 10 minutes in TBS-T. Detection was achieved by eliciting HRP luminescence by incubating the membrane with SuperSignal WestFemto solution (Thermo Fisher) according to the manufacturer’s instructions, and imaging the membrane using a c600 Transilluminator from Azure Biosystems.

**Immunoblot of muscarinic** M_2_ **receptor transfected in COS-7 cells**

SDS-Triton protein extraction - COS-7 cells were collected 48 h after transfection and treated with lysis buffer (50 mM TrisHCl pH 7.8, 1% Triton X100, 0.1% SDS, 250 mM NaCl, 5 mM EDTA, 100 mM NaF, 2 mM NaPPi, 2 mM Na3VO4, 1 mM PMSF). Cell lysates were then centrifuged at 16000 g for 15 minutes at 4 °C and supernatants, containing solubilized receptors resolved by SDS-PAGE or stored at -80°C. Samples were mixed with 65 mM Tris, 10% glycerol, 2% SDS, 0.1 M DTT, 0.001% bromophenol blue, pH 6.80 with HCl, boiled for 5 min and applied to a 10% SDS-PAGE.

SDS-PAGE - Resolved proteins were transferred to a PVDF membrane (Bio-Rad). The membrane was blocked in blotto (5% non-fat dry milk in 1xTBS plus 0.1% Tween 20) and the expression level of the M_2_ receptor was assayed with a mouse anti-Myc-tag diluted 1:1000 in blotto and thereafter in a HRP-conjugated secondary antibody diluted 1:1000 in blotto. Membranes were then incubated in SuperSignal West Pico chemiluminescent substrate (Thermo Fisher Scientific Inc.) and the bands detected using a Bio-Rad ChemiDoc XRSplus imaging system. Optical densities of blot bands were finally determined using a computer-assisted densitometer (ImageJ U. S. National Institutes of Health, Bethesda, Maryland, USA), normalized versus the tubulin internal control.

**Detection of phosphorylated ERK1**

On day zero, HeLa cells were plated on 6-well plates (70,000 cells/well). On day 1, the cells were transiently transfected with the plasmid of interest (1 µg DNA/well). On day 3, the cells were exposed to serum-free medium until the day of the assay. On day 4, the cells were treated with 100 μM carbachol (time 0’; 1’; 5’ and 20’) at 37 °C. The cells were then lysed in a buffer containing 50 mM TrisHCl (pH 7.8), 1% Triton X100, 0.1% SDS, 250 mM NaCl, 5 mM EDTA, 100 mM NaF, 2 mM NaPPi, 2 mM Na3VO4, 1 mM PMSF. Samples were incubated on ice for 30 min and then centrifuged at 17,000 rpm for 15 min at 4 °C. The supernatants were recovered and assayed for protein concentration. Protein extracts were run on a 10% SDS-PAGE and transferred on a PVDF membrane (Bio-Rad). The membrane was then blocked in blotto (5% non-fat dry milk in 1xTBS plus 0.1% Tween 20) and the extent of phosphorylation of (ERK) mitogen-activated protein kinase was determined by immunoblotting with anti-phospho-ERK (Sigma-Aldrich) diluted 1:1000 in blotto. The blots were stripped and re-blotted with the anti-ERK (Sigma-Aldrich), diluted 1:1000 in blotto, to estimate the total amount of kinase loaded. Detection of the immunoreactive bands was carried out by the enhanced chemiluminescence method (SuperSignal west Pico, Thermo Fisher Scientific Inc.), by using a ChemiDoc XRSplus imaging system (Bio-Rad Laboratories, Milan, Italy), and their optical densities determined by using a computer-assisted densitometer.

**Cell culture of H9c2 cells**

H9c2 (ATCC, H9c2 (ATCC; CRL-1446)) were cultured in DMEM (Dulbecco’s Modified Eagle Medium (DMEM, 4.5 g/L D-glucose, 110mg/L Sodium Pyruvate) (from Thermofisher Scientific), supplemented with 10% Fetal Bovine Serum (FBS) and 1% Penicillin/Streptomycin (P/S). Cells were grown in 25 cm^2^ (check) flasks and maintained at 37C and 5% CO_2_. Cells were seeded into dimension here glass coverslips which had been coated using Poly-L-Lysine solution (PLL, 0.01%) in 6-well plates.

Cells were transfected using Lipofectamine 2000 (Thermofisher) reagent according to the manufacturer’s protocol, such that 2.5 µg plasmid DNA was transfected per coverslip at least 24 hours before imaging. Cells were stained with MitoTracker Deep Red FM (Thermofisher) through a 100x dilution of 1mM MitoTracker to each coverslip. They were stained with Hoechst 33342 Solution through a 1000x dilution of 1mg/ml Hoechst to each coverslip.

**Oxygen consumption measured with the Clark electrode**

A Clark type electrode-based polarographic method was used to evaluate oxygen consumption, in a 2 ml volume chamber kept under continuous stirring at the constant temperature of 37 °C[9] . Brieﬂy, after determination of the baseline of oxygen consumption in the presence of the sole culture medium, 6 x 106 of transfected cells were added, and the disappearance of oxygen was monitored through a Yellow Springs Instruments Model 53 Oxygen Monitor device (YSI Inc., Yellow Springs, OH). Since the electrode consumes oxygen during measurement, the rate of oxygen decrease in 2 ml of DMEM media with no cells was subtracted from the sample’s oxygen consumption rates. Results are expressed as ng-atom of oxygen/min/106cells. For the evaluation of oxygen consumption rate in the absence of oxidative phosphorylation, the ATP synthase inhibitor oligomycin was added to the measurement vessel through a fine needle.

**G_i_ protein activation FRET assays**

HEK/TSA cells were co-transfected with plasmids carrying the M_2_ receptor or its mutants (Table), together with a plasmid carrying the Gi_1_ FRET biosensor at a 1:3 ratio[10]. The co-transfection was performed using Effectene Transfection Reagent (Qiagen) following the manufacturers protocol.

| **Plasmid** | M_2_ **receptor** | **Fluorescent reporter** |
| --- | --- | --- |
| pVitro2_M_2_wt_myc_IRES_mRuby2 | M_2_wt | mRuby2 |
| pVitro2_M_2_M368A_myc_IRES_mRuby2 | M_2_M368A | mRuby2 |
| pVitro2_M_2_stop228_myc_IRES_mRuby2 | M_2_stop228 | mRuby2 |

HEK/TSA cells were seeded in a 100mm dish to a total number of 2x106 cells in 10ml DMEM (Thermo Fisher Scientific) supplemented with 10% FCS, 1% glutamine and 1% penicillin/streptomycin (PenStrep). After 24 hours, the co-transfection was performed as described above. After additional 24 hours, transfection efficiency was checked using a fluorescence microscope. After confirmation of successful transfection, the cells were plated in a 96 well black bottom plate to a total number of 5x104cells per 200µl per well. Untransfected HEK/TSA were plated as a control. The next day the cells were washed three times with 200µl 1X Hanks' Balanced Salt Solution (HBSS, Thermo Fisher Scientific) and the FRET baseline was measured using the BioTek SynergyTM Neo2 Hybrid Multi-Mode Microplate Reader. The donor fluorophore was excited with a wavelength of 430/30 and the emission was measured at 491/30. The acceptor fluorophore was excited with a wavelength of 500/18 and the emission measured at 541/20. The FRET signal was measured with an excitation wavelength of 430/20 and an emission wavelength of 541/20.

Following the baseline measurement, 200µl acetylcholine diluted in 1X HBSS were added to the cells. The concentrations were chosen to be around the published EC50 of acetylcholine for the M_2_ R of around 0,1-0,03µM. 200µl 1X HBSS were added to wells containing the untransfected control cells.

**Supplementary results related to the muscarinic M_3_ receptor**

**Ligand binding and functional properties of co-transfected M_3_trunk(1-272)/M_3_tail(M-388-589) fragments**

As previously shown by[7], none of the two muscarinic receptor fragments, generated by splitting the receptor at the level of the i3 loop, M_3_trunk(1-272) and M_3_tail(M-388-589) showed [^3^H]NMS binding activity when expressed alone in COS-7 cells. In contrast, a considerable number of specific [^3^H]NMS binding sites was observed after co-expression of M_3_trunk with M_3_tail, and the binding affinities for [^3^H]NMS and carbachol were identical to the wild type M_3_ receptors. Furthermore, co-expression of M_3_trunk with M_3_tail resulted in functional receptors able to increase phosphatidylinositol hydrolysis (**Table S2**).

**Ligand binding and functional properties of M3stop273**

In line with what as been observed with, M_2_stop228, M_3_stop273, a receptor mutant bearing a stop codon at the beginning of the i3 loop, exhibited binding activity when expressed in COS-7 cells, albeit at very low level of expression, such as 52.3 fmol/mg of protein. Remarkably, the calculated [^3^H]NMS KD value, obtained from direct saturation experiments was similar to that calculated for the wild type M_3_ receptor (**Table S2**). Furthermore, competition experiments showed that the agonist carbachol was able to inhibit [^3^H]NMS binding and the isotherm was best fitted by a one site binding model (**Table S2**).

M_3_stop273 was functionally active increasing the phosphatidylinositol hydrolysis after carbachol stimulation, even though the extent of response was reduced compared to the wild type M_3_ receptor (**Table S2**).

**Lack of [^3^H]NMS binding of M_3_stop240 and M_3_stop503**

In analogy to what has been done with the M_2_ receptor, we analyzed M_3_ receptor constructs with a stop codon upstream and downstream the i3 loop. None of the M_3_ receptor mutants bearing the stop codon in TM V, such as M3stop240, or in TM VI, such as M_3_stop503, were able to bind [^3^H]NMS (**Table S3**). Nevertheless, the binding of M_3_stop240 could be rescued by co-transfection with M_3_trunk(1-272), while the binding of M_3_stop503 was restored in the presence of M_3_tail(M-388-589) (**Table S3**). These data agree with previous ones showing the capability of receptor fragments to interact functionally with defective mutants.

**Lack of** [^3^H]**NMS binding of the double mutants M_3_stop240/stop503 and M_3_stop273/stop503**

In order to deepen our knowledge about the mechanism involved to rescue mutants bearing the stop codon in the regions TMV, (TMVI) and i3 loop, we created two additional mutants with two stop codons, such as M_3_stop240/stop503 and M_3_stop273/stop503.

None of these two receptors exhibited binding to [^3^H]NMS when they were transfected alone in COS-7 cells (**Table S3**). Nevertheless, co-transfection of M_3_stop273/stop503 with M_3_tail(M-388-589) rescued [^3^H]NMS binding activity, with Bmaxs similar to those observed with the co-transfection of M_3_trunk(1-272) with M_3_tail(M-388-589) (**Table S3**). Conversely, the co-transfection of M_3_stop240/stop503 with any of the M_3_ fragments did result in [^3^H]NMS binding (**Table S3**).

**Ligand binding properties of M_3_stop273/fr.sh**

As a stop codon could hypothetically be interpreted as a sense codon encoding for an amino acid, in order to check for stop codon read-through, a frame shift was created by inserting a four base AATT 15 nucleotides after the stop codon 273, resulting in the mutant named M_3_stop273/fr.sh. . The presence of the frame-shift after the artificial stop codon did not alter the [^3^H]NMS binding activity of the receptor that showed Bmax values of 51.5 pmol/mg of protein. (**Table S3**).

**Ligand binding properties of M_3_stop273/hairpin**

In order to check if termination re-initiation was the mechanism responsible of the functional properties of the mutant M_3_stop273, a 42 bases long palindromic structure, was inserted 15 nucleotides after the stop codon, resulting in the M_3_stop273/hairpin receptor. This palindromic sequence when transcribed into mRNA, forms a hairpin structure with a ΔG value of −64 kcal/mol, which can effectively block ribosome scanning[8] . The presence of the hairpin loop after the stop codon, which avoids the re-initiation process, slightly reduced but did not abolish [^3^H]NMS binding activity of the receptor, with Bmax values that was 29.7 pmol/mg of protein (**Table S3**).

**Supplementary methods related to the muscarinic M_3_ receptor**

**Generation of mutant muscarinic M_3_ receptors**

Rat M_3_ muscarinic receptor expressed in pcD plasmid[11] [11, 12][11, 12]was used to construct the different mutants of muscarinic M_3_ receptors. The two expression plasmids referred as M_3_trunk(1-272), containing transmembrane domains I-V and the N-terminal portion of the third cytoplasmic loop, and M_3_tail(M-388-589), containing transmembrane domains VI and VII, and the C-terminal portion of the third cytoplasmic loop, were described previously [7].

M_3_stop273 – This plasmid was created substituting codon 273 (CAA) at the N-terminal of the i3 loop of M_3_ with a stop codon 273 (TAA).

M_3_stop273/stop503 – This plasmid was created by inserting in M_3_stop273 an additional stop codon in TM region VI, at position codon 503, TGG was substituted with TAA.

M_3_stop503 – This plasmid was created by substituting codon 503 (TGG) in TM region VI of M_3_ with a stop codon (TAA).

M_3_stop240 – This plasmid was created by substituting codon 240 (TAC) in TM region V of M_3_ with a stop codon (TAA).

M_3_stop240/stop503 – This plasmid was created by inserting in M_3_stop240 an additional stop codon in TM region VI, at position codon 503, TGG was substituted with TAA.

M_3_stop273/fr.sh. – This plasmid was created by inserting in M_3_stop273 a four bases AATT fifteen nucleotides after the stop codon in order to create a shift in the correct reading frame.

M_3_stop273/hairpin – This construct was created by inserting in M_3_stop273, fifteen nucleotides after the stop codon, a 42 bases long sequence (AGGGGCGCGTGGTGGCGGCTGCAGCCGCCACCACGCGCCCCT). This palindromic sequence when transcribed into mRNA, forms a hairpin structure with a ΔG value of −64 kcal/mol, which can effectively block ribosome scanning [6].

**Generation of a bicistronic construct expressing the i3 loop of muscarinic M3 receptor between the Sirius and EGFP proteins**

Sirius-M_3_i3(558n)-EGFP – This plasmid was generated by inserting between the two fluorescent protein Sirius and EGFP the i3 loop of the muscarinic M_3_ receptor, from nucleotide 880 to nucleotide 1437. A PacI enzyme was inserted between the stop codon of Sirius and the beginning of the M_3_ i3 loop (**Fig. S5A**).
