# Supplementary material for: The C-terminus of the prototypical M2 muscarinic receptor localizes to the mitochondria and regulates cell respiration under stress conditions
Source: PLoS Biol. 2024 Apr 29;22(4):e3002582. doi: 10.1371/journal.pbio.3002582 (PMC11093360; doi:10.1371/journal.pbio.3002582)
Supplement: Attachment — Submitted filename: Reviewers reply 13.07.2023.pdf [file pbio.3002582.s018.pdf]

Reviewers' comments

Rev. 1:

This study by Fasciani & Petragnano et al, is **original and of great interest** in the field of G protein coupled receptors (GPCRs). The study reveals a cap-independent translation of the C-terminal part (Ctail) of the M2 muscarinic receptor, involving an internal ribosomal entry site (IRES) at the level of the DNA sequence corresponding to the internal loop i3 of the receptor. The authors use different strategies to reveal this IRES-dependent expression of the Ctail and show that the expression of the Ctail fragment is increased under starvation conditions of the culture medium and that it localizes to mitochondria. Under stressed conditions induced by cellular starvation, the Ctail interacts with a component of the respiratory chain in mitochondria to decrease mitochondrial respiration, decrease ROS production and decrease cell proliferation. The authors conduct their study mainly in a HEK293 cell model but also extend it to mouse tissues and human iPSC cells where they show
the existence of the M2 Ctail fragment and the impact of its expression. This study deserves to be published in Plos Biology. However, some controls need to be added, some corrections need to be made to improve the clarity of the statements, and some concerns and interpretations need to be clarified.

We would like to thank the reviewer for his supportive, insightful and detailed feedback. We have revised the manuscript addressing all these points. In some instances, we feel that we had already provided the controls required by the reviewer, and they had been clouded by inadequate formatting, and especially by a serious quality issue with the graphics caused by a PDF conversion problem. We sincerely apologise for this problem and thank the reviewer for still providing a careful and point-by-point review.

1. It is difficult to understand and analyse the figures when the descriptions and text are not exact, or sometimes impossible to read. Authors are strongly encouraged to proofread the manuscript and correct inaccuracies
- Label of fig1A is missing, is the second panel M2-STOP228-EGFP?

Yes, the reviewer is correct. We had inadvertently brought the panel on top of the label, and this mistake has now been corrected. We apologies for this error.

We do not see cells in the insert (brightfield) of fig1A of non transfected.

We have enhanced the contrast of the inset of Fig1A in order to better visualize the cells, that are present albeit they do not display any signal in the GFP-channel.

- Fig2 is difficult to understand
Annotations are of very bad quality and impossible to read
- Fig2A and 2C text is not of good quality, it is very difficult to read the text. Idem for Fig3, the text is not visible

Concerning these well founded comments by the reviewer, we overall apologise sincerely because there was clearly an issue in the PDF conversion. Albeit a source word file had been provided, including high-resolution figures, clearly the low quality of the manuscript hampered the work of the reviewer. We made sure that figure quality is now preserved in the resubmission. The minor labelling issues raised by the reviewer have been thoroughly addressed.

- Lane 240 "This is as also confirmed from ribosome profiling data from human left ventricle tissue, displaying increased p-sites as well as overall ribosomal coverage in correspondence of this region (Fig. S4B)". what are "p-sites" ? Figure S2B is just

impossible to read

Ribosome profiling is a method that uses specialized mRNA sequencing to determine which mRNAs are being actively translated in a cell or tissue. It produces a global snapshot of all the ribosomes actively translating in a cell at a particular moment, known as a translatome. Consequently, this enables the identification and the location of translation start sites (P-site), the complement of translated ORFs in a cell or tissue, the distribution of ribosomes on a messenger RNA, and the speed of translating ribosomes.
The P-site (for peptidyl) is the second binding site for tRNA in the ribosome. The other two sites are the A-site (aminoacyl), which is the first binding site in the ribosome, and the E-site (exit), the third. During protein translation, the P-site holds the tRNA which is linked to the growing polypeptide chain. An increase in P-site coverage is normally associated to translation initiation.

- Graph Fig4B: impossible to read the Y axis legend

Again, this has been fixed after PDF conversion was improved.

- FigS8 legends: S8A "below" is not the lower panel but the right panel

We thank the reviewer for pointing this out, we have corrected this.

- Fig S10B-S10D legends are too small- Impossible to read

Again, this has been fixed after PDF conversion was improved.

2. Controls are needed

2.1. Line 122-FigS2A-Table1: To fully validate the hypothesis of a reconstitution of competent M2 receptor with the Nter and Cter parts of the recepror when a stop codon is introduced at position 228 of M2, *coexpression of the Nter fragment (1-228, comprising only TM1-5 trunk, without the rest in the DNA construct) with the Cter tail (228-446, comprising only TM6-7)* would be necessary.
Indeed the residual binding observed with M2-Stop228 could be due to expression of a weak fraction of full length M2 despite the introduction of a stop at position 228 since ribosomes can have the properties to go through and pass STOP codon.
Same remark for M3 in Table S2 and figS5. Furthermore, Western Blot profiling of the different constructs should also be shown -
We thank the reviewer very much for these comments. Indeed, we have already performed the N-ter + C-ter co-expression experiment, which was not included in the current manuscript version to streamline the flow.

The table below, also included in the source data of our new submission, shows that radioligand binding is observed also for such reconstitution (bottom two lanes). Moreover, the reconstitution of the two fragments indicated by the reviewer was published by Maggio et al in 1993 for both M2 and M3 receptors (Reconstitution of functional muscarinic receptors by co-expression of amino- and carboxyl-terminal receptor fragments. Maggio R, Vogel Z, Wess J. FEBS Lett. 1993 Mar 15;319(1-2):195-200), and confirmed for several other receptors such as dopamine D2 and D3 receptors (Scarselli, M., Armogida, M., Chiacchio, S., De Montis, M.G., Colzi, A., Corsini, G.U. and Maggio, R. (2000) Reconstitution of functional dopamine D(2s) receptor by co-expression of amino- and carboxyl-terminal receptor fragments. Eur. J. Pharmacol. 397, 291–296: Scarselli, M., Novi, F., Corsini, G.U. and Maggio, R. (2003) Functional rescue of the inactive splice variant of the dopamine D3 receptor D3nf. Brain Res. 987, 244–247 CrossRef PubMed).

Binding data				Adenylyl cyclase inhibition assay	
Receptor	Bmax (fmol/mg)	[^{3}H]NMS KD (pM)	Carbachol IC50 (μM)	Carbachol EC50 (μM)	Maximum inhibition of forskolin stimulated cAMP accumulation (%)
M_2	980 ± 60	62 ± 7	2.1 ± 0.6	0.04 ± 0.01	53 ± 5
M_2trunk(1-283)	N.B.				
M_2trunk(1-228)	N.B.				
M_2stop228	45 ± 2	70 ± 10	1.8 ± 0.4	0.13 ± 0.02	27 ± 3
M_2trunk(1-283) +M_2tail(M-281-466)	480 ± 20	79 ± 10	0.7 ± 0.2	0.15 ± 0.03	37 ± 4
M_2trunk(1-228) +M_2tail(368-466)	70 ± 10	72 ± 8	0.6 ± 0.2	0.17 ± 0.03	29 ± 4

Review table 1

Stop-codon readthrough could not account for the weak observed binding in M2stop228, since in Table S1 we showed that the constructs, M2stop228/fr.sh., in which, a four nucleotides segment to shift the reading frame was inserted after the stop codon 228, was still able to bind specifically [3H]NMS. Another construct, shown in Table S1, M2stop228/Hairpin, in which an hairpin loop was inserted after the stop codon 228, excludes stop-codon readthrough. Hairpin loops are stable structures that have been shown to block ribosome scanning (Kozak M. Constraints on re-initiation of translation in mammals. Nucleic Acids Res. 2001;29: 5226–5232. doi:10.1093/nar/29.24.5226; Kozak M. Circumstances and mechanisms of inhibition of translation by secondary structure in eucaryotic mRNAs. Mol Cell Biol. 1989;9: 5134–5142. doi:10.1128/mcb.9.11.5134-5142.1989), but despite the the hairpin loop, M2stop228/Hairpin still binds [3H]NMS.

By constructing the mutants M3stop273/fr.sh. and M3stop273/Hairpin, we have also ruled-out stop-codon readthrough for the M3stop273 receptor (see Table S3).

We shall note here that, especially for GPCRs, we believe western blots are not the most suitable tool for validating these constructs, and that radioligand binding data combined with direct, single-cell microscopic imaging are more sensitive and reliable approaches. However, according to the recommendation from the reviewer and in order to prove that M2stop228 produces an N-terminal fragment that ends at amino acid 228 we conducted a western blot where we run side to N-terminally

tagged myc-M2stop228 myc-M2trunk(1-228), I.e. the construct where the DNA sequence is interrupted after coding for aa 228. The data clearly show that the N-terminal portion of M2stop228 and M2trunk have comparable molecular weights (~55 kDa, accounting for likely glycosilation).

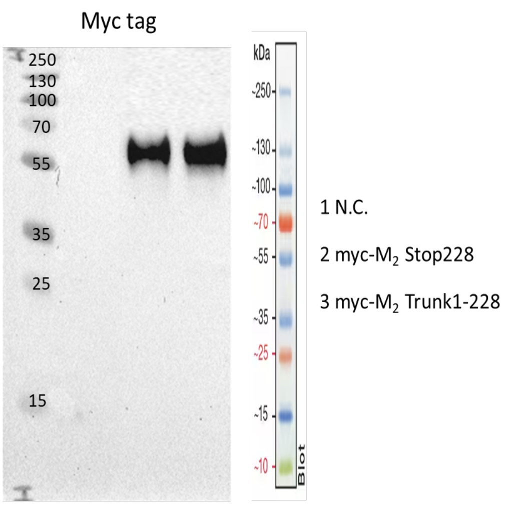

Reviewer Figure 1 Western blot of whole-cell lysates of untransfected HEK293 cells (Lane 1, N.C.= negative control), HEK293 transfected with myc-M_2Stop228 (Lane 2) and myc-M_2Trunk(1-228) (Lane 3). Detection was conducted using 1:1000 anti myc-Tag (9B11) mouse primary antibody (Cell Signaling). After overnight incubation, thes were washed 3x 10 minutes in TBS-T and incubated at RT for 1 h with a secondary antibody,1:3000 anti-mouse IgG HRP-linked antibody from Cell Signaling). On the lower part is shown a loading control, αTubulin (11H10) rabbit primary antibody (1:1000) detect with a secondary antibody (1:3000) anti-rabbit IgG HRP-linked from Cell Signaling)

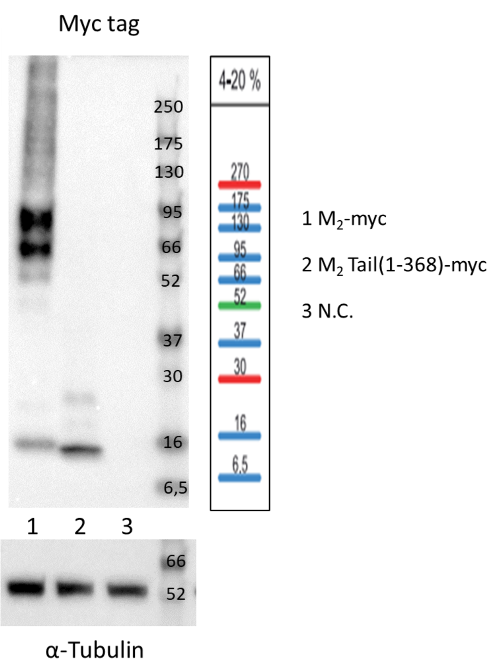

Reviewer Figure 2 Western blot of whole-cell lysates of , HEK293 transfected with M_2-myc (Lane 1) M_2Tail(1-368)-myc (Lane 2) and untransfected HEK293 cells (Lane 3, N.C.= negative control). Detection was conducted using 1:1000 anti myc-Tag (9B11) mouse primary antibody (Cell Signaling). After overnight incubation, the membranes were washed 3x 10 minutes in TBS-T and incubated at RT for 1 h with a secondary antibody (1:10000 anti-mouse IgG/(anti rabbit) HRP-linked antibody from Cell Signaling). On the lower part is shown a loading control, αTubulin (11H10) rabbit primary antibody (1:1000) detect with a secondary antibody (1:3000) anti-rabbit IgG HRP-linked from Cell Signaling)

For comparison, the full length receptor runs in several bands, the highest of which at around ~75 kDa (as also shown in Figure S6K), and prominently dispaying the C-terminal associated fragment bands running as a doublet at 15 and 21 kDa, that are eventually localized to the mitochondria, as shown by the blot in Figure 3C. As indicated in the manuscript, we ascribe the higher molecular weight band of the two arising from posttranslational modifications. The offset between the 15 kDa tail band originating from the full length receptor and that from the C-terminal fragment itself, is in agreement with the effect arising from the slightly different linker used to fuse the myc tag.

In addition to this, and to further characterise our constructs, as requested by the reviewer, we performed western blot with the M2stop228-Myc mutants expressed in COS7 cells. The blot shows that the M2stop228 runs as two bands, with the lower molecular weight band matching the M2tail(368-466), and the upper molecular weight band corresponding to the 1-228 trunk portion of the receptor.

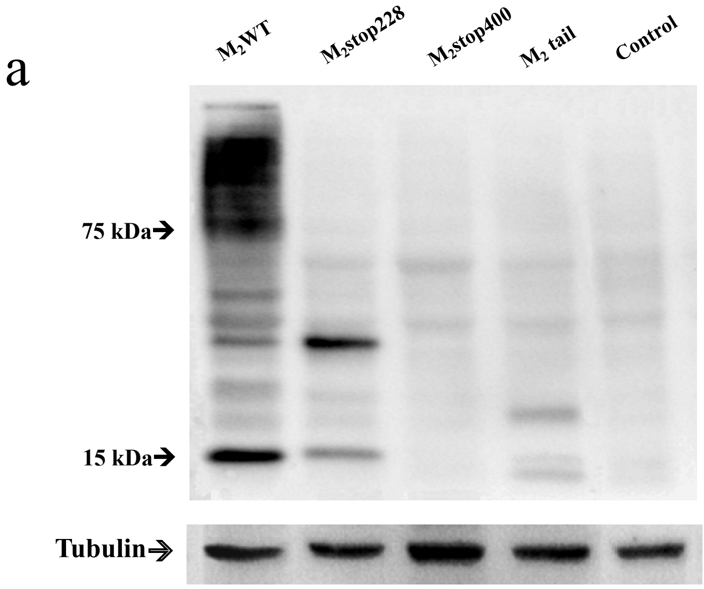

Review figure 3 WB M2/C-Myc, M2stop228/C-Myc, M2stop400/C-Myc and M2tail(368-466)/C-Myc containing the human Myc tag (EQKLISEEDL) at C-terminal were transiently transfected in COS-7 cells and immunodetected via Western Blot after SDS-Triton protein extraction. Both M2 wild type, M2stop228 and M2tail(368-466)/C-Myc showed 15 kDa bands corresponding to the segment of the M2 receptor from ATG 368 up to the C-terminal. The 75 kDa bands indicated by the arrows in M2 WT corresponds to the full lenght wild type M2 receptor. On the contrary, M2stop400 did not show any band representing an internal control.

TableS1: Explain why the coexpression of MT2-STOP196 + M2-Trunk1-283 shows some binding; properties it is rather unexpected. Furthermore, controls of expression of all the constructs must be provided; without checking level of expression of mutant constructs, conclusions can be erroneous. Same remarks for the experiments related to the M3 receptor

The M2-stop196 mutant is an additional control that we provided to demonstrate that the stop codon must be inserted within the i3 loop in order to split the receptor in a functional way. Insertion of the stop codon in transmembrane regions, generate fragments that are unable to bind ligands. In the M2-stop196 mutant, the stop codon in the middle of Transmembrane region V provides a truncated fragment that is unable to reassemble with the tail part. Nevertheless, the M2tail fragment generated by the IRES mediated mechanism, can reassemble with a cotransfected M2-Trunk1-283 and regain [3H]NMS binding. The same reasoning can be done for the mutant M3stop240 cotransfected with M3trunk, since again the stop codon is in transmembrane region V.

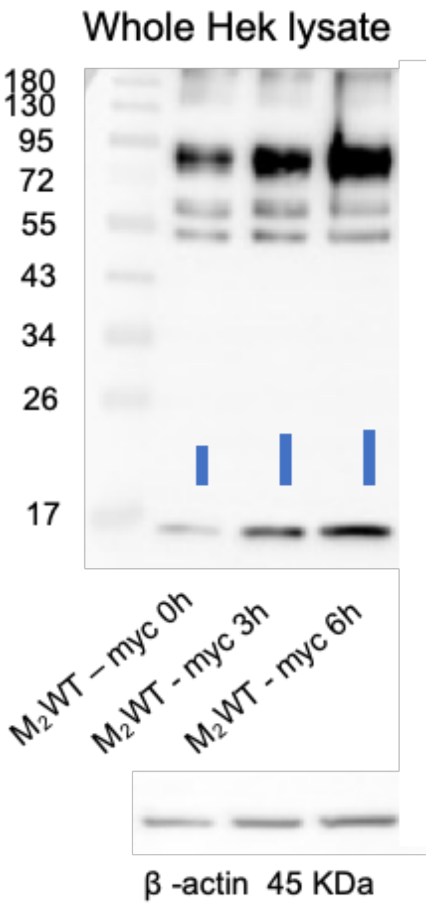

2.3 Fig3C: The authors should show the expression of each construct in the total lysate compared to the mitochondrial fraction. Evaluation of expression via IRES vs cap dependent expression would be interesting to know.

We thank the reviewer for this comment. Indeed, we had already provided this result in the supplementary findings, see Fig. S6K. We have now added a legend to guide the reader as to the origin of the different bands observed in the blot in the total lysate. Prior to our experiments reported in Figure 5, we had conducted in the past an experiment where me measured the relative increase (blue bars) of the C-terminal fragment with respect to the full length receptor in HEK293 subject to increasing starvation, a driver of increased stress response and thus of IRES activity (Reviewer Figure 4). The data qualitatively

Reviewer Figure 4 Western blot of whole-cell lysates of , HEK293 transfected with M2-myc and lysed upon increasing times of starvation (medium without serum). (3). Detection was conducted using 1:1000 anti myc-Tag (9B11) mouse primary antibody (Cell Signaling). After overnight incubation, the membranes were washed 3x 10 minutes in TBS-T and incubated at RT for 1 h with a secondary antibody (1:10000 anti-mouse IgG/(anti rabbit) HRP-linked antibody from Cell Signaling). Below, loading control using beta actin. Blue bars indicate the relative ratio of C terminal domain band intensity to full length receptor band.

show an increase of the expression of the C-terminal fragment for increasing times of starvation.

2.4. Fig 4C in vitro import into yeast mitochondria: I have the feeling that some controls are lacking: for ex, same condition of expression and import but this time in the absence of yeast mitochondria ?

We believe that the best control in this type of experiment is the full length M2 receptor protein that does not enter in the mitochondria (left portion of the blot). It is important to stress the fact that in this assay the M2 and M2tail(368-466) proteins, are synthetized in vitro in presence of a 35S radiolabelled methionine and after purification they are added to the mitochondrial preparation. The first line (5%), in both left and right blots of Fig. 4C, represents the aliquot of the M2 (left) or M2tail(368-466) (right) synthetized in vitro, and added to the mitochondrial preparation.

3. Inaccuracies and concerns:

- Fig3B: M2 is not well visible at the cell surface in 1B in contrast to what is seen in other figures

We thank the reviewer for this comment. In this specific case, the receptor is indeed expressed at the membrane. However, we focused the imaging plane on to the basal membrane, i.e. the one adhering to the coverslip. In line to previous reports, this leads to a diffuse homogeneous signal unlike the more common 'annular' intensity profile that is observed when the imaging plane crosses the cell 'halfawy' through its axial extent. We enclose here a library of images of the basolateral membrane of the M2. For comparison, we include here several confocal snapshots of the basal membrane of HEK293 cells expressing several different fluorescently labeled GPCRs.

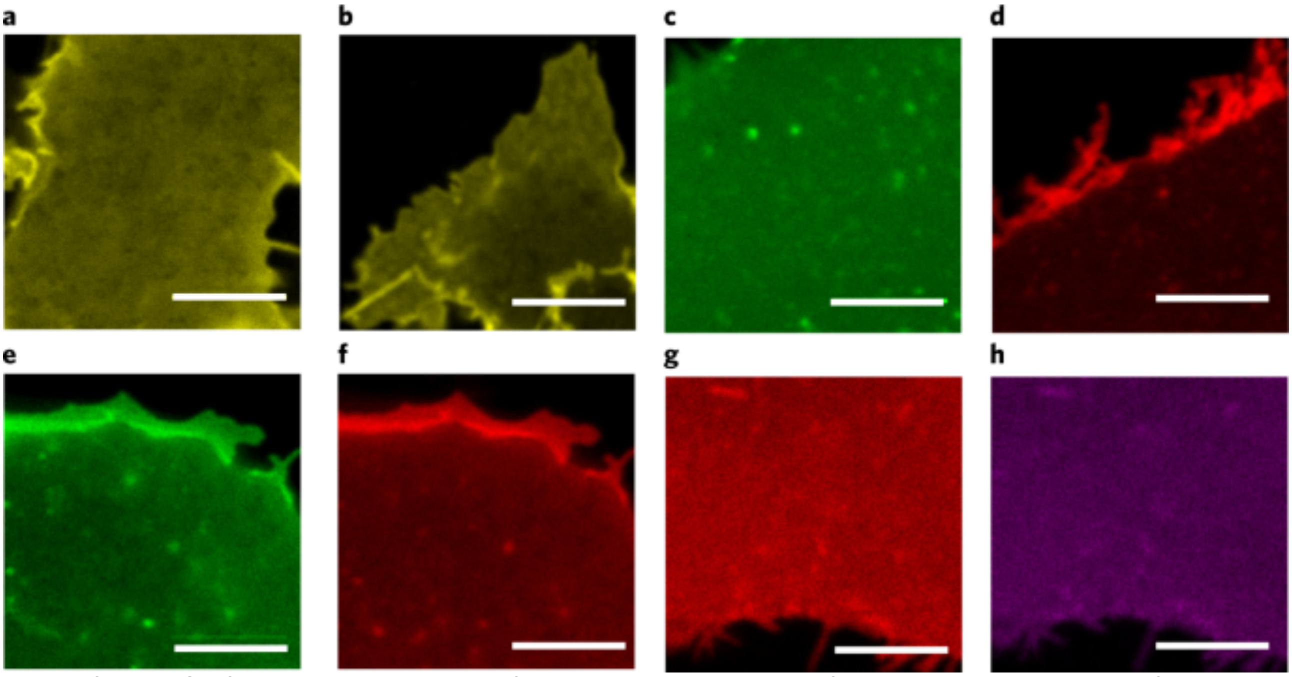

*Review figure 5 Confocal microscopy images of the basolateral membrane of HEK293AD cells expressing β1-AR-EYFP (a), EYFP-β2-AR (b), Y2R SNAP-Cell 505-Star labeled SNAP-β1-AR (c), Y2R labeled by using TAMRA-labeled Ahx(5-24)NPY at a concentration of 1 μM (d), Cy3 labeled 98TCO*β2-AR-EGFP, imaged respectively in the EGFP (e) and Cy3 channel (f) and 263TCO-CRF1R equimolarly labeled with Cy3 (g) and Cy5 (h). Scale bars, 5 μm.(Adapted from Isbilir… and Annibale, Nature Protocols 2021)*

- Fig3E: mitochondrial localization is not possible to see clearly, the images are not convincing. In addition, separate spectral green channel displayed at the bottom does not seem to correspond to the one from the merge top image.

We are sorry that the reviewer does not find the image as convincing. We would like to respectfully point out that reviewer 2 instead appears convinced by the 'large' degree of colocalization of green signal in mitochondria. We felt the folded mitochondria at the center of the cell were a good example of mitochondrial localization. We do agree, however, that there are also some green particles that do not colocalize with mitochondria. This is a common instance with labeled proteins, that may be degraded, or trafficked in vesicles. In any case, we include here for the benefit of the reviewer a few more examples of mitochondrial localization of the M2-fr.sh.-EGFP construct, both in HEK293 cells as well as in a new cell type we tested during revision, namely the cardiomyocyte-like H9c2. We have generated an additional Supplementary Figure panel (Fig S7C) containing our results from H9c2 cells.

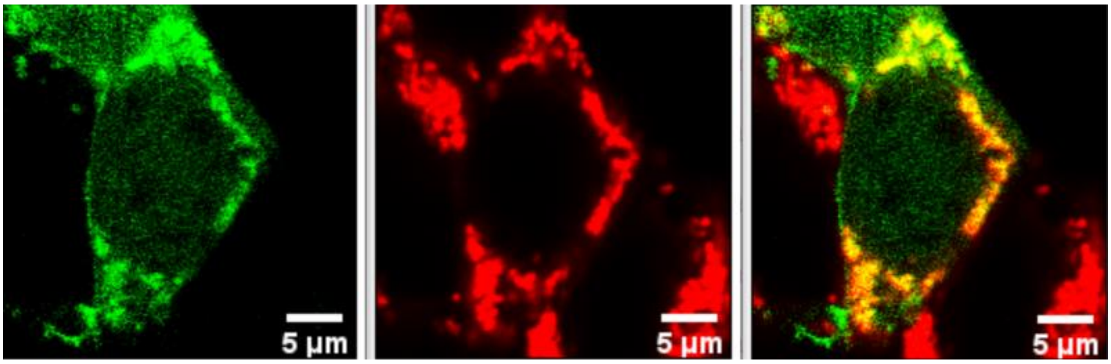

Review figure 6 M2-fr.sh.-EGFP expressed in cells stained with mitotracker red (in red) in HEK293 cells. Overlay shown in yellow (courtesy of Miss Maithri Murali, MolMed intern 2020)

We also tested a different cell line (H9c2), that displays a similar colocalization of M2tail with the mitochondrial marker Tom20-mCherry (in magenta in the figure below).

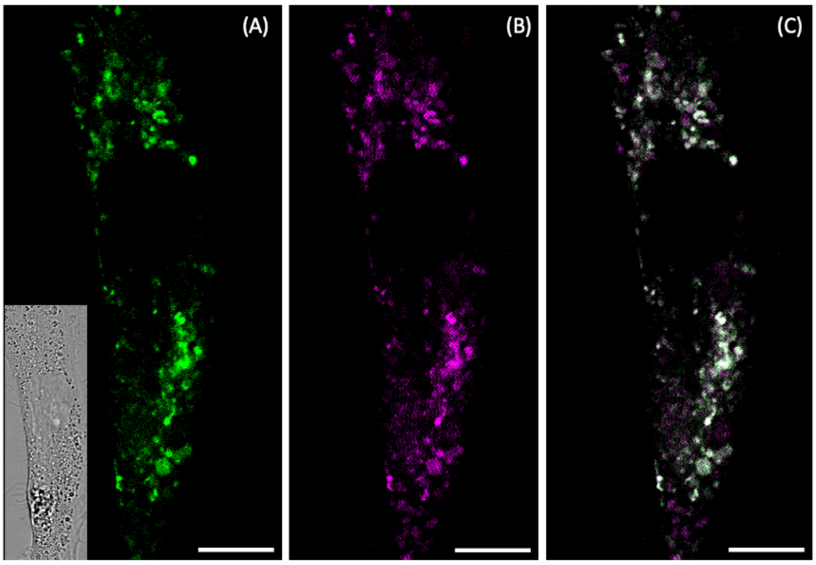

Review figure 7 Fluorescence images produced due to the co-transfection of the M2 frameshift-EGFP and the mitochondrial stain Tom20-mCherry in H9c2 cells. (A) The expression of the M2 frameshift-EGFP (B) The expression of the Tom20-mCherry receptor. (C) Composite images of both receptor expressions, where white regions indicate overlap.

- FigS6J: The WB dispalys several bands (of which a predominant one below 17kDa and several above 55kDa). What are all the bands seen in WB ? What is the size of Ful length M2 ? what is the size of C-tail fragment? This should be specified in the legend and in the text

We thank the reviewer for this insightful comment. As anticipated in response to one of the reviewer's previous comments, we have now included in what is now Figure S6K a legend, alike to what we did for Figure 7A. In general, the M2 receptor from whole cell lysates (mammalian cells) runs at approximately 75 kDa, due to glycosylation adding to the molecular weight of the receptor. This can be seen in the blots from the mitochondrial import in yeast. Here, the 'naked' receptor has a molecular weight of 55 kDa, that matches the expected molecular weight of the M2R based on the aminoacid sequence. In our blots the full-length receptor runs as a triplet of bands, spanning approximately the 90-55 kDa range. There are high molecular weight aggregates (more prominent when the lysates are boiled) running at a higher molecular weight. On the other hand, the C-termina fragment (expected MW~15kDa) runs typically as a doublet, the lower band at 15kDa and an upper band at 21 kDa. The upper band is most likely, is the result of a posttranslational modification of the lower band that, perhaps, enables the fragment to be transported into the mitochondria, albeit we felt that a further investigation of this aspect was beyond the scope of our current work, and will be thus the focus of follow-up investigations.

- Lane 340 and FigS7: The authors wrote "Similar mitochondrial localization was also observed when we transfected HEK293 cells with M2fr.sh-mRuby2 (Fig. S7B)." However in FigS7B, mitotracker staining is missing.

We thank the reviewer for this observation. We have altogether repeated the measurement, and now figure S7B displays 4 panels, clockwise for M2tail(368-466)-EGFP, M2fr.sh-mRuby2 (in red), overlay and DIC. In the top right cell the

colocalization is perfect. Please not that the lower left cell expressed the M2fr.sh-mRuby2 but no M2tail(368-466)-EGFP.

- Fig4A images are of very poor quality - impossible to judge the integration into mitochondria

We apologize deeply once again for the image conversion problem. The images at the appropriate resolution are now included.

- FigS8:

FigS8: scales are indicated to be 10um; however the scale bar size is different in all images but cell size seem to be similar….

There is no nuclear staining here, for some images, it would be necessary to include it

We thank the reviewer for these comments and suggestions. In Figure S8 the scale bars wereindeed all 10 µm, and when a larger field of view was chosen (as in the case of the former Figure S8E, this was done to show that the negative control did not show any signal across a larger population of cells. In any case, following the suggestion by the reviewer, we have incorporated several more images (there are now 40 individual micrographs) of our fluorescence complementation controls, including large field of view micrographs of the negative controls, and including DIC for all images (which we believe, provides equivalent information to that conveyed by a nuclear staining, i.e. allows to identify also non-transfected cells). We hope the information provided is now acceptable to the standards of the reviewer.

Idem nuclear staining of S6 would help. Plasma membrane is difficult to distinguish

We imagine the reviewer is referring here to Figure S6 B-F. In this case, as the focal plane is at the plasma membrane, any nuclear staining would be very faintly visible, if at all. For this reason, we believe that, by using a colorscale that emphasizes low intensities in panel C and F, we have actually done the best possible effort to highlight the plasma membrane.

In the other panels of Figure S6, we are only showing colocalization of two different proteins/stains at the level of the mitochondrial network.

Fig S8A: Indicate the MW of M2 Ctail incorporated into yeast mitochondria? Is it the one <17kDa

We thank the reviewer for this comment, since the molecular weights were indeed not indicated in this panel and have now been added. Indeed, what wasd isplayed in Supplementary Figure 8A (now Fig. 7C in the revised version) is the 15 kDa M2tail fragment.

Fig S8E and S8G: For the right interpretation, controls of expression of the constructs should be shown

We thank the reviewer for this comment. Based on their input, since we have data from at least three independent transfections for each conditions, we went back to our source images and incorporated other images. The Figure is now restructured and the complementation expanded in order to incorporate for all positive controls both a broader as well as a smaller field of view, providing more detail on the mitochondrial localization as well as on the cell morphology, as requested by the reviewer.

For the two negative controls, we have harmonized providing two equally large FOVs. We also included DIC images for completeness. We had decided not to include nuclear staining since typical stains such as DAPI or Hoechst have rather

broad spectra, that may have counfounded the appraisal of low level GFP expression. We believe that by having included the DIC images on top of mitochondrial stain, the cell morphology should now be easier to appraise.
Concerning quantification of expression in the controls, as antibodies against split GFP fragments are not available, we would be unsure about how to proceed with respect to the recommendation by the reviewer. However the constructs shown in former Figure S8E and S8G are the same leading to a signal in Figure S8C, D and F when expressed with their complementary pair. We did not expect a reduction in transfection efficiency. Moreover, we include here a figure containing large field of views from 4 independent transfections of the key negative control M2tail-GFP1-10 + SMAC-GFP11: all figures show negligible amount of signal (and thus) recombination, if at all, in the green channel (red channel is mitotracker).

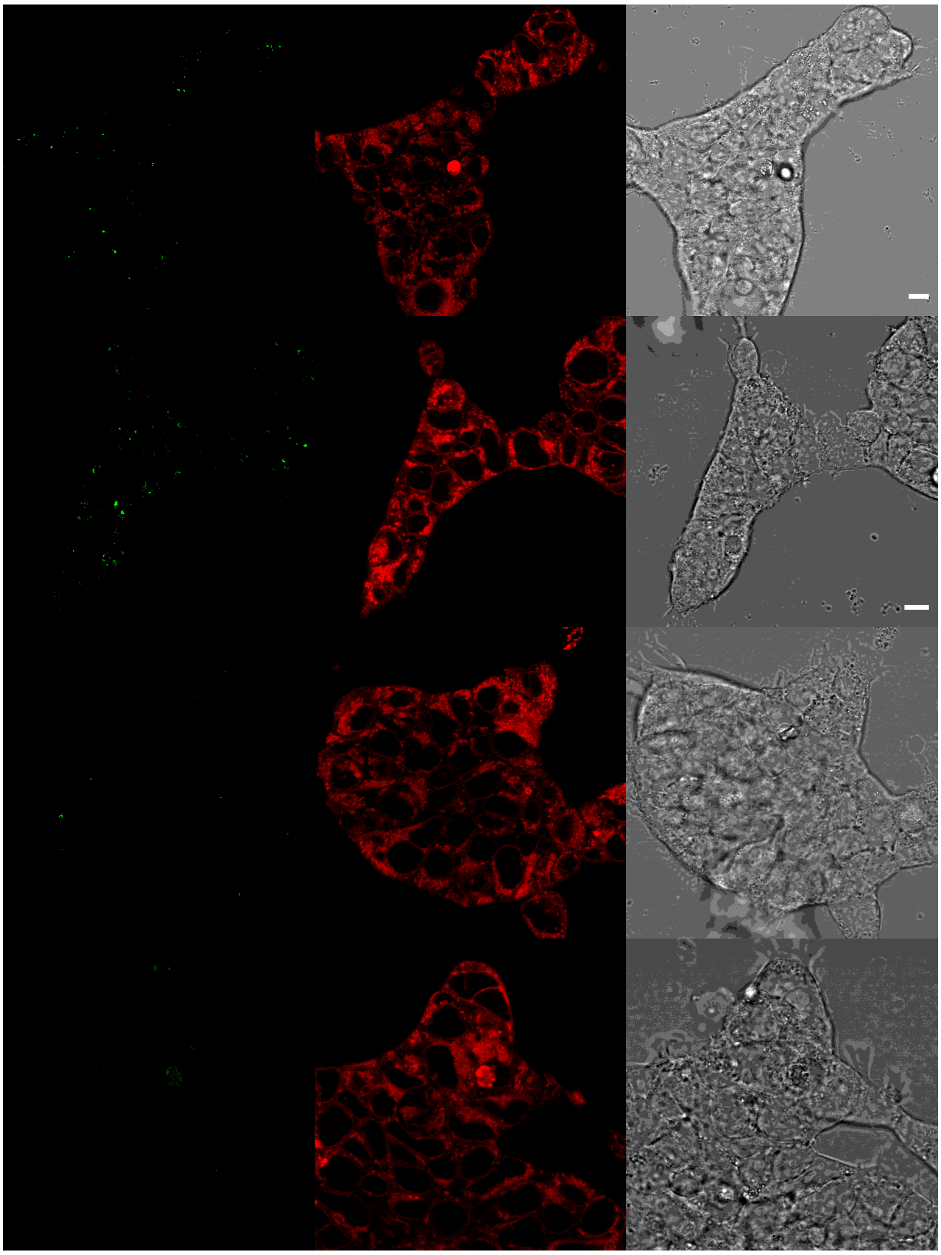

Review figure 8 independent transfections of M2tail(368-466)-GFP11 and SMAC-GFP1-10 in HEK293 cells (as also shown in Figure S8E). Scale bar is 10 µm throughout.

- Lane 442 and Fig5C-5D: "The green fluorescence detected at the plasma membrane is explained with the chaperoning effect of M2-GFP11 on Mito-GFP1-10 before its sorting to

the mitochondria". This observation calls into question the Mito-GFP1-10 sensor. Indeed Mito-GFP1-10 being observed at the plasma membrane instead of mitochondria suggests that the mitochondrial sensor is not specific; What are the comments of the authors regarding this aspect ?

We thank the reviewer for raising this valid and insightful comment. The localization of two complemented proteins when the GFP11-GFP1-10 pair is used will, in our experience, be determined by which protein takes the lead in terms of subcellular localization. In Figure 5C we believe that cap dependent receptor production is dominant, and when the receptor is expressed and targeted to the membrane it carries with it any mito-GFP1-10 that it finds around in the cytosol. On the other hand, when the C-terminal fragment is upregulated under stress and it is targeted to the mitochondria we observe a predominant mitochondrial signal. The latter can arise both from a recombination with mito-GFP1-10 prelocalized in the mitochondria (due to the 24 hours headstart in transfection of this construct) and from the chaperoning effect of the M2tail(368-466)-GFP11 on any mito-GFP1-10 being concomitantly transcribed.

- The physiological role of Ctail is not clear, its impact on cellular apoptosis or proliferation is not clear. Indeed:
FigS10D: Expression of M2stop228, M2Stop400 and M2tail increases cleaved caspase 3 compared to WT, suggesting that expression of the 3 constructs including C-tail induced some cell death (while WT does not). However, the authors wrote in the text Lane 483: "no difference in the number of apoptotic cells among the different cell groups as shown by the caspase-3 activation assays of apoptosis" Is there a discrepancy here ? Does the Ctail increase apoptosis ?

We thank the reviewer for this comment. We agree with the reviewer that we are only beginning to understand the potential physiological roles of Ctail (i.e. M2tail(368-466 or M2 C-terminal fragment). However, based on our data, we can say with confidence that in absence of its IRES-mediated, physiological expression, cell proliferation and oxygen comsumption rates are increased. This occurs both when the M2Receptor is overexpressed, but also, importantly, at endogenous expression levels, as demonstrated by our data from Crispr/Cas9 edited hiPSCs reported in Figure 7C-E.

Concerning the Caspase assay of Figure S10D: we are afraid that our choice of showing the overexposed blot in the bottom part of the figure has caused some confusion. The key result is that there is no sizable cleaved Caspase 3 for any of the lanes observed.
The amount of cleavage, when looking at the overexposed blot, is in line with that of the control construct M2 stop400. In this construct no c-terminal is made, hence any Cleaved Caspase 3 band represents the ‘background noise’. Moreover, of note, the already faint M2tail band is fainter than the negative control M2 stop400 band.
We include here, for the reviewer’s benefit, an example of HEK293 cells where Caspase cleavage instead occurs upon Taurosporine treatment of the cells. The blot shows that under untreated conditions there is a faint band of cleaved Caspase 3, but this is negligible with respect to the intensity of the band when cleavage is induced by Taurosporine.

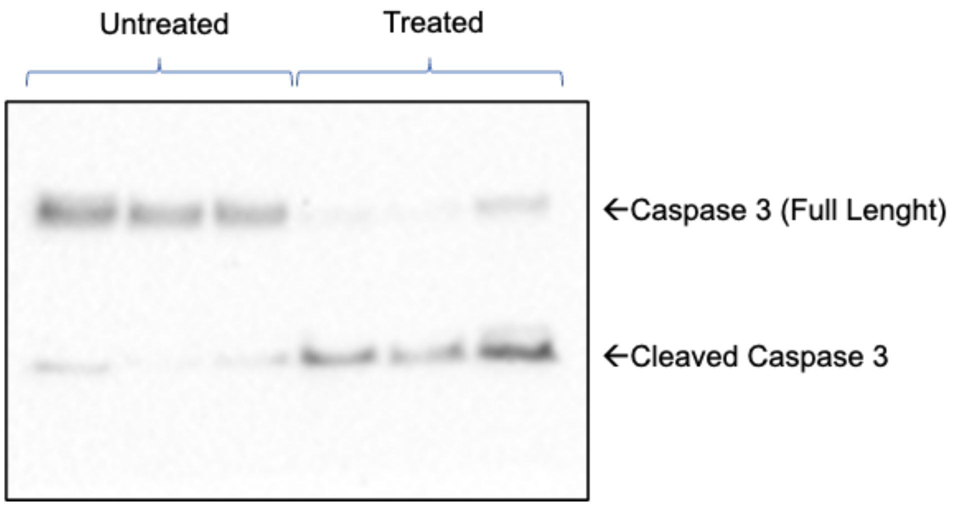

Review figure 9 Positive control for Caspase 3 cleavage assay

Ctail-induced apoptosis would also match the increased cell viability assay on HEK293 cells transfected with M2-M368A (ie in cells not expressing Ctail) (Fig6), or the cell proliferation assay performed on M368A hiPSCs (Fig7D), compared to WT receptor
Extra experiments would certainly be needed to clarify this aspect
In order to address this concern, in Figure S10E we show that the mitochondrial network of cells overexpressing (for 24-48h) M2tail(368-466)-EGFP is in excellent condition, and this does not change upon serum starvation. We hope we have clarified this concern and doubt of the reviewer.

Minor:
Lane 230: Is the word "However" appropriate here ?
We thank the reviewer for this and the subsequent careful proofreading comments. We have omitted the "however".
Lane 240, "This is as also confirmed… » remove "as"
done
Lane 328 A word is missing at the end of the sentence: " ….the mechanism of C-terminal fragment production is therefore close to physiological."
We have changed the sentence by adding "close to physiological expression".

Fig3D legends, remove « as well as strongly to the cell mitochondria »; since nothing in 3D show that the localisation is in mitochondria; replace by intracellular compartments or organelles. Idem in the text lane 332
We have amended the caption in Figure 3D. However, we believe that in the case of Figure 3E we indeed show mitochondrial localization, as testified by the counterstain. We feel have addressed this question thoroughly in response to the major point above raised by the reviewer.

-Fig2
"OF" is missing in the Title of fig2: "Localization of the IRES within the i3 loop OF the muscarinic M 209 2 receptor using a fluorescence reporter assay."
We thank the reviewer for spotting this typo, that is now amended.
The different constructs of fig2A are not easy to understand; adding colors for the different parts of the i3 loop fragments would help the reader
We thank the reviewer for this suggestion, and we have indeed coded the different sections. We have also improved the graphics of the barcharts displayed in Figure

2A and B.

- Figure 4

Fig 4C in vitro import into yeast mitochondria:

Legend does not seem correct. It is said line 428 « The kinetics pattern of M2tail is what it is expected for a protein imported in mitochondria: the appearance of the cleaved M2-tail is indicative of a post translational modifications of the fragment once imported in mitochondria.» I do not see the notion of post-translational modification here; what do the authors mean ?

What is the line at 5% correspond to ? Nothing is indicated in the legend.

We apologise for this refuse, that originated from changes to the figures from an older version of the manuscript, as all the information concerning the in-vitro import into yeast mitochondria were contained in the Supplementary Methods, now moved to the main Materials and Methods. We have removed the comment about post translational modifications from the caption, as it more appropriately belongs in the discussion, were we hope it is now in the appropriate context:

'Once inside the mitochondria, it undergoes processing leading to the appearance of a lower molecular weight band, observed both in Western blots from whole cell and mice tissue protein extracts, as well as from purified mitochondria and in the yeast in vitro assays (Fig. 4C). The fragment is found predominantly in a carbonate-insoluble fraction, i.e. a membrane, and it appears to form a high molecular weight complex of ~180 kDa with other mitochondrial proteins (Fig. S7~~C~~D).'

Fig4E. What is TEM ?

We meant Transmission Electron Microscopy, but we rephrased it as Immuno Electron Microscopy and harmonized throughout the manuscript.

Fig4F-G: specify in the legend the antibody associated with gold particles, and also needs controls with cells without M2

We thank the reviewer for pointing this out. The secondary, gold conjugated antibody used is EM Grade 10nm Goat anti-Mouse IgG Conjugate EM.GAM10/1, from BBC UK, and is now clearly spelled in the Methods section. We shall note here that the primary antibody was an anti myc-Tag (9B11) mouse primary antibody (Cell Signaling), and so we obtained Gold particle counts only in transfected cells. Furthermore, COS-7 cells were transfected with M2stop228-myc not M2-myc. The M2-myc would have produced two recognizable proteins, the full length M2-myc receptor and the M2tail-myc fragment, while the M2stop228-myc produces only one recognizable protein that is the M2tail-myc fragment that has a predominant mitochondrial localization.

The results of our counts in transfected vs non-transfected (without M2stop228-myc) cells is displayed below, together with a representative comparison of a control (immunostained, from untransfected cells, LEFT) and a transfected and immunostained (right) IEM micrographs. We shall note here that the background granularity observed in the negative control has spatially broader and less defined features than those of the sharp gold nanoparticles observed in the positive staining, as can be seen when enlarging the images.

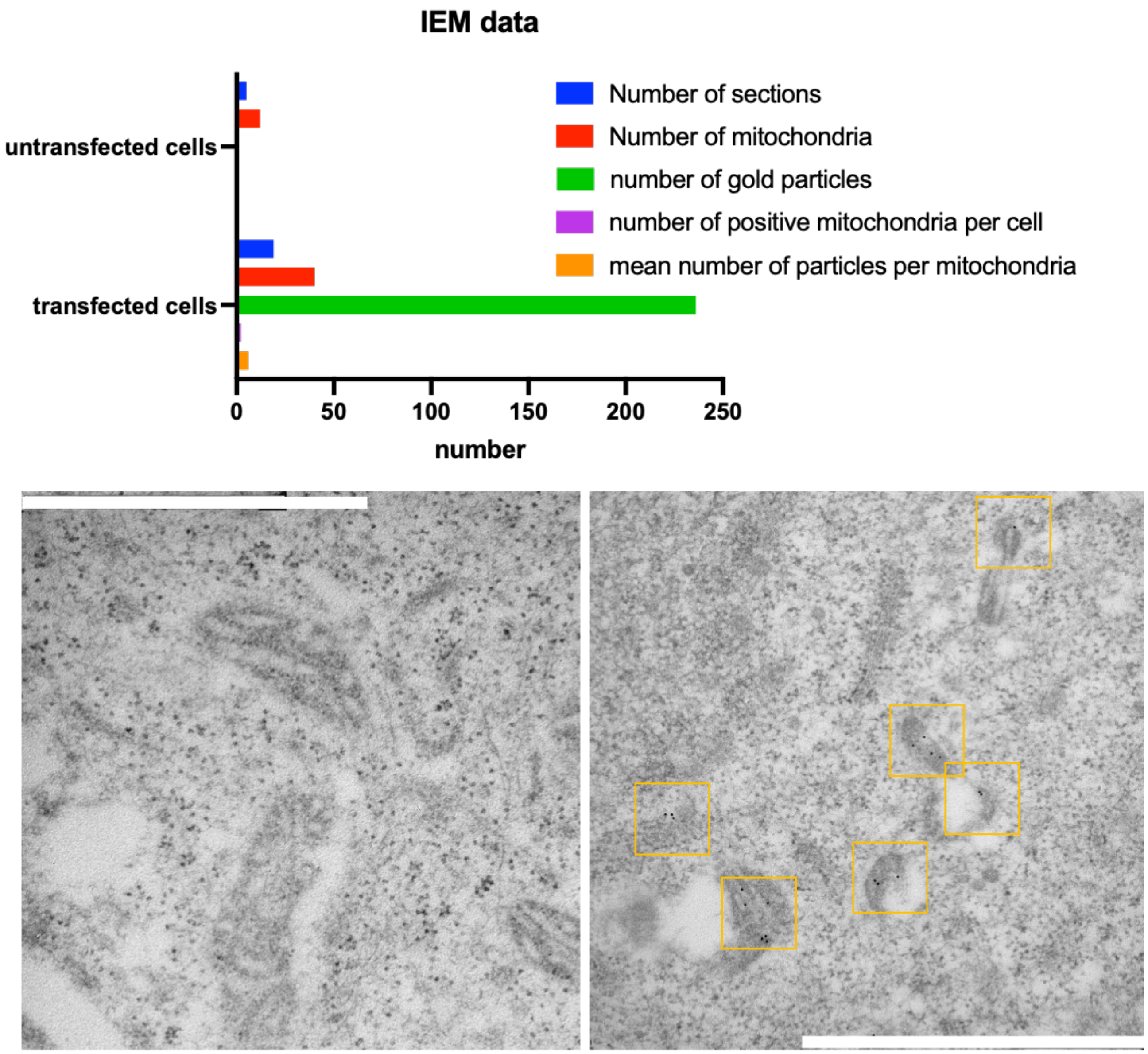

Review figure 10 Statistics of IEM staining, both in transfected as well as control cells. Representative IEM micrographs of a control (left) and transfected (right) COS-7 cells. Scale bar is 1 µm.

-Figure S9

FigS9D colocalisation should be dispalyed in white

In this specific figure we used blue instead of magenta to render mitotracker signal, as magenta would have been too close to red. We shall note that blue and red gives purple/magenta, as colocalization color.

FigS9H à mitochondria (mitotracker) are barely visible

Again scale of the images on top and bottom are not the same

We thank the reviewer for these observations. Figure S9 has been re-edited, and we introduced in panel D magnified images of mitochondrial localization of IRES generated M2tail(368-466)-mRuby2 or absence thereof in panel I. Scale bars are always 10 µm, but in this case the two original images were taken with slightly different pixel numbers (albeit same pixel size), and we refrained from cropping panels.

- Figure S10

Put a distinguishable box to differentiate the top and bottom panels of S10D
We thank very much the reviewer for this sensible suggestion, that we have duly implemented.

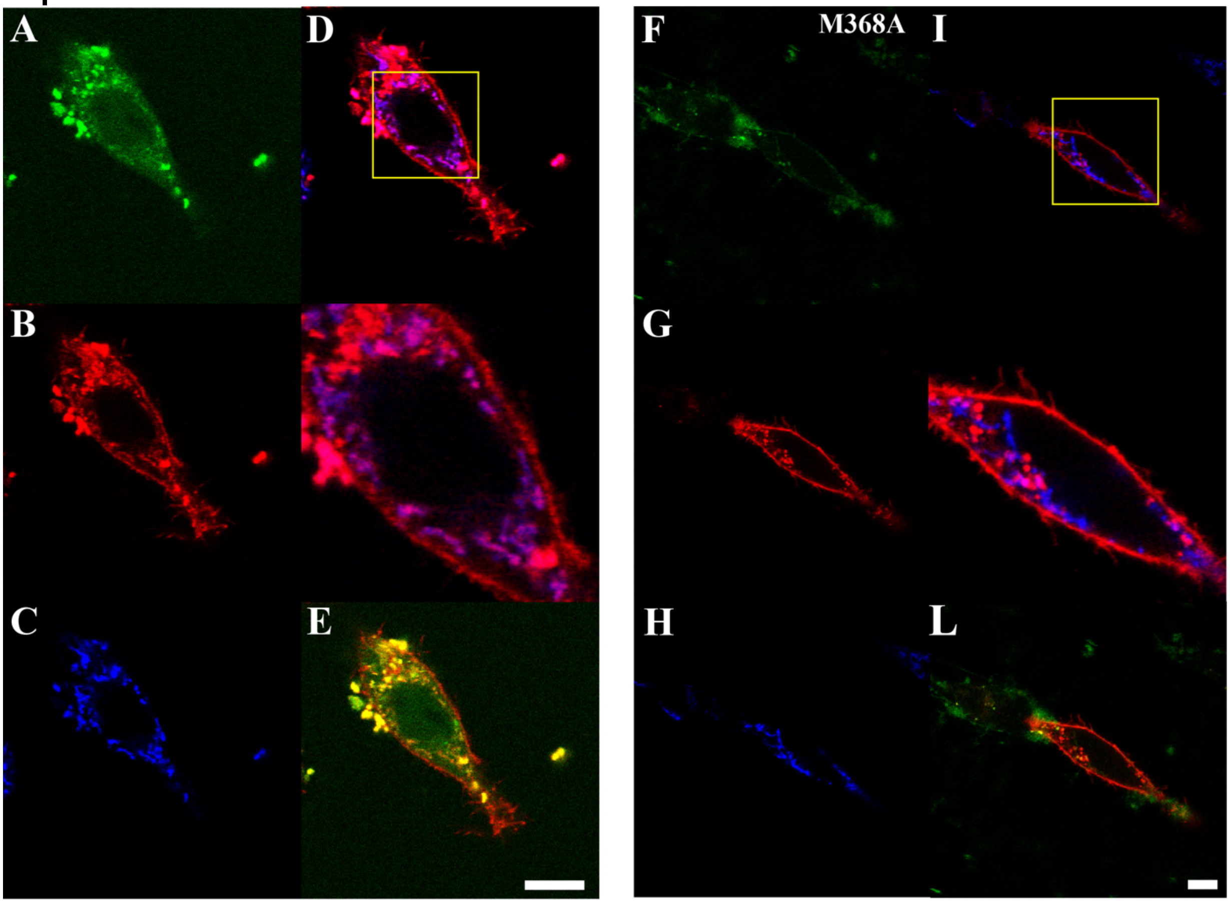

Put a box to distinguish the 3 panels of S10E
We thank the reviewer for this observation. We have spaced the panels slightly in order to distinguish them

Rev. 2:
In this paper, the authors reported that there is an internal ribosome entry site (IRES) in the third intracellular loop of M2 receptor which splits M2 receptor into two fragments. They showed that the carboxyl terminal fragment locates to the mitochondria inner membrane and regulates various functions including oxygen consumption and cell proliferation. The authors employed various techniques and a lot of different constructs to comprehensively map out the IRES site, report the subcellular localization and assess the functions.
I think this is interesting and potentially impactful, but I have a few comments as detailed below.

We thank the reviewer for this fair assessment. We shall note here however that the IRES does not actually split the receptor in two, but yields, on top of the canonical, full length M2 receptor, also a C-terminal fragment (M2tail) starting from the end of the third intracellular loop. So the full length receptor and the C-terminal fragment are both present. This concept is explained in the scheme of Fig. 8

1. How often does cap-independent translation of M2 happen and does it have significant impact in physiological conditions? Majority of M2 receptor locates at the plasma membrane as shown by the authors themselves and many other papers. If less than 5% locates to the mitochondria, would that have any significant impact?

We thank the reviwer for this frank assessment. We share his concern for the physiological relevance of the C-terminal fragment, and for this reason we strived to provide quantitative measurements of this.
Fig. 6 in its entirety is dedicated to assay the role of the fragment in transiently transfected cells. The role of the C-terminal fragment in impairing mitochondrial respiration (Fig. 6A), ROS production (Fig. 6B) and cell proliferation (Fig 6C, please note that the M368A mutant does NOT produce the C-terminal fragment) is unambiguous.

The authors tried to address this question using the M2-tdtomato knock-in mice and used western blot to look at the expression of M2-tdtomato and M2-C tail in the tissue and purified mitochondria. These bands are not quantifiable and I think it is of great importance to report the approximate ratio of M2 C tail located in mitochondria out of the total M2.
In order to assess and quantify the role of the fragment in physiological settings <u>and</u> at the endogenous level, as the reviewer pointed out, one of our strategies was to use M2-tdtomato knock-in mice. The experiments reported in Fig. 7A and B are however only meant to show the existence of the C-terminal fragment also in organ lysates from the animal, and its import in mitochondria, which is clear in Fig. 7B.

Moreover, the size of the band is questionable. The M2 receptor has a molecular weight around 52 kDa, not the 75kDa as stated in the paper (Line 558). This also directly contradicts with authors' own western blot in Figure 4C where the M2 runs a bit lower than 55 kDa. This raised the question whether the bands in Fig. 7A are really the M2 receptor bands. All the controls are without marker and the quality could be improved.

Concerning the western blots we believe there has been a misunderstanding. We shall note here that while the 'naked' M2 has an expected molecular weight of 52 kDa, it is glycosylated, and thus runs normally at a higher molecular weight. As shown by us (Fig. S6K, microsomal fraction) and others, the typical bands for the receptor are around 75kDa. A lower molecular weight band (~50-60 kDa) can be observed if the receptor is purified from Sf9 insect cells (e.g. PSH Park, JW Wells - Biochemistry, 2003), that have -however- markedly different glycosylation patterns with respect to mammalian cells.
The same applies to the in-vitro data shown in Figure 4C. Here the receptor is translated in-vitro, thus it cannot become glycosylated, and it shows the 'naked' molecular weight of ~55 kDa.
In the case of the receptor tagged with td-tomato (in itself, 55 kDa), the molecular weight becomes ~75+55=130 kDa, which is in line with what is observed in the blots of Figure 2A. On the other hand, the C-terminal fragment fused to td-tomato, now runs at approximately 75 kDa.

2. Generalization to other members of the GPCR superfamily? The authors could only identify M2 and M3 out of more than 800 different GPCRs. M2 and M3 has a very long i3 loop which are not common in GPCR family, so could the long i3 loop needed for cap-independent translation? so that it only limits to few GPCRs with long i3 loop?

We thank the reviewer for this comment and suggestion. As we explore a new mechanism (IRES mediated translation of a GPCR fragment) and concomitantly its atypical localization (mitochondria), while demonstrating the physiological impact, we decided here to focus only on 2 GPCRs. We clearly do not exclude that the

mechanism may be shared across other GPCRs, as hinted in the manuscript section 'Generalization to other members of the GPCR superfamily', based on the sequence alignments to the broader muscarinic superfamily. Moreover, we would like to confidentially share with the reviewer some preliminary data with the dopamine D2 when the C-terminal domain translation in the cap dependent construct is abolished, the receptor continues to function, analogously to the stop228 construct for the M2 receptor.

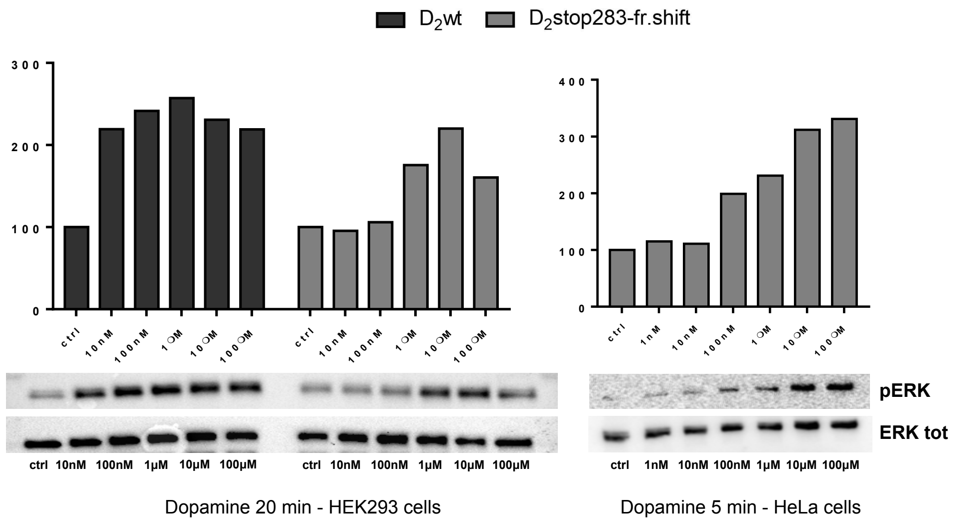

Review figure 11 ERK activation downstream of the Dopamine D2 receptor . HEK293 cells were transfected with either the WT Dopamine D2 receptor or the D2stop283-fr.shift construct. On th eright, results in HeLa cells for a shorter dopamine stimulation.

3. Fig. 3B the M2 receptor localizes in the cytosol instead of the plasma membrane, which is not canonical localization as the authors claimed. In fact, the authors contradict themselves in the very same figure. In figure 2D, the M2-mRuby2 shows a predominant cell plasma membrane localization.

We thank the reviewer for this comment. In Figure 3B we are showing a confocal (optical section) micrograph of the basal membrane of the cell (in fact, two cells at the end of their division), hence the homogeneous look of the receptor distribution. We have addressed a similar concern by Reviewer 1, and pointed to exemplary images of several labeled GPCR at the basal membrane of HEK293 cells displaying exactly this phenotype. In Figure 3D we are instead imaging 'halfway' through the axial extend of the cell, and the labeled membrane shows the more canonical annular morphology. Simply put, Fig. 3B is a slice of the feet of the cell, whereas Fig. 3D is a slice taken at waist level.

4. Fig, 3E. there are a large amount of green signal which overlaps with mitotracker, but there are also large amount of green signal which are not. Those green signals look like that they are not diffused. Where do they locate?

We thank the reviewe for pointing this out, and also for supporting the notion that indeed there is a strong colocalization with mitotracker.
The dots that do not colocalize could be C-terminal fragment being degraded into vesicles, or being trafficked. We shall remember that the C-terminal fragment retains two transmembrane domains, so will tend to 'live' in membranes, either mitochondrial or endosomal.

5. In Figure 4 E, F, G. I could see many similar particles in the images. Are these not real signals? Please clarify.
We thank the reviewer for checking carefully our electron microscopy images. While at low zoom the gold nanoparticles are hard to identify and distinguish from overall 'noise' and other sources of contrast, at higher magnifications we feel their identification (H-K)is unambiguous. We shall note furthermore that the Regions of Interest in Figure 4F and Figure 4G are arbitrary, and do not include all particles visible in the mitochondria. For Example, there are other gold nanoparticles visible in Figre 4F (to the top and left of the H square).

Minor:
Fig. 1A. top right panel add text M2stop228-EGFP.
We thank the reviewer for pointing out that the text was masked by the Figure.
Fig. 1A. Are they HEK293 or COS7 cells? "Imaging setting are the same for the five images" five or four? there are only four images in figure 1A.

In our work we have used both HEK293 and COS7 cells. COS7 cells were used for most of the radioligand binding experiments, whereas HEK cells were primarily used for microscopy. However, for consistency and verification, most of the microscopic imaging and biochemistry was conducted on both cell types as for example reported in Figure S6J (current version) (formerly Figure S7C). We shall further note here that we tested IRES activity in three further cell types, as displayed in Figure S4A, and more recently also in H9c2 cells (as shown in the reply to Reviewer 1 above).
In Figure 1A, the top right panel illustrates a COS7 cell, the other panels are HEK293. The confusion about 4 vs five panels arises because the sentence was supposed to be at the end of the caption and refer to all imaging panels. We have now corrected this.

Page 15, line 418, over a 4h interval? Do you mean 1h interval?

What we mean here is that after transfection, we waited 5h and then started imaging for an additional 4h (over a 4h interval). Images were on the other hand taken every 30 minutes, as shown in Figure 4B.
