# Supplementary material for: The C-terminus of the prototypical M2 muscarinic receptor localizes to the mitochondria and regulates cell respiration under stress conditions
Source: PLoS Biol. 2024 Apr 29;22(4):e3002582. doi: 10.1371/journal.pbio.3002582 (PMC11093360; doi:10.1371/journal.pbio.3002582)
Supplement: Attachment — Submitted filename: PBIOLOGY-D-22-02557_R4 annotated.pdf [file pbio.3002582.s020.pdf]

PLOS Biology

# One gene - two proteins: The C-terminus of the prototypical M2 muscarinic receptor localizes to the mitochondria and regulates cell respiration.

--Manuscript Draft--

| | |
|---|---|
| Manuscript Number: | PBIOLOGY-D-22-02557R4 |
| Full Title: | One gene - two proteins: The C-terminus of the prototypical M2 muscarinic receptor localizes to the mitochondria and regulates cell respiration. |
| Short Title: | The C-terminus of the M2 muscarinic receptor localizes to the mitochondria |
| Article Type: | Research Article |
| Keywords: | GPCR; Internal Ribosome Entry Site; mitochondria; Fluorescence Microscopy; fluorescence complementation; cellular respiration; hiPSC |
| Corresponding Author: | Paolo Annibale<br>Max Delbruck Centre for Molecular Medicine: Max-Delbruck-Centrum fur Molekulare Medizin in der Helmholtz-Gemeinschaft<br>Berlin, GERMANY |
| First Author: | Irene Fasciani |
| Order of Authors: | Irene Fasciani |
| | Francesco Petragnano |
| | Ziming Wang |
| | Ruairidh Edwards |
| | Narasimha Telugu |
| | Ilaria Pietrantoni |
| | Ulrike Zabel |
| | Henrik Zauber |
| | Marlies Grieben |
| | Maria E Terzenidou |
| | Jacopo Di Gregorio |
| | Cristina Pellegrini |
| | Silvano Santini Jr |
| | Anna R Taddei |
| | Barbel Pöhl |
| | Stefano Aringhieri |
| | Marco Carli |
| | Gabriella Aloisi |
| | Francesco Marampon |
| | Eve Charlesworth |
| | Alexandra Roman |
| | Sebastian Diecke |
| | Vincenzo Flati |
| | Franco Giorgi |
| | Fernanda Amicarelli |
| | |

Powered by Editorial Manager® and ProduXion Manager® from Aries Systems Corporation

| | |
|---|---|
| | Andrew B Tobin |
| | Marco Scarselli |
| | Kostas Tokatlidis |
| | Mario Rossi |
| | Martin J Lohse |
| | Paolo Annibale |
| | Roberto Maggio |

| | |
|---|---|
| **Abstract:** | Muscarinic acetylcholine receptors are prototypical G protein-coupled receptors (GPCRs), members of a large family of seven transmembrane receptors mediating a wide variety of extracellular signals. We show here, for the first time in a GPCR, that the carboxyl terminal fragment of the muscarinic M2 receptor, comprising the transmembrane regions 6 and 7 (M2tail), is expressed by virtue of an internal ribosome entry site located in the third intracellular loop. Single cell imaging reveals that M2tail, whose expression is upregulated in cells undergoing integrated stress response, does not follow the normal route to the plasma membrane, but is almost exclusively sorted to the mitochondria inner membrane: here it controls oxygen consumption, cell proliferation and the formation of reactive oxygen species by reducing oxidative phosphorylation. Crispr/Cas9 editing of the key methionine where cap-independent translation begins in human induced pluripotent stem cells, reveals the physiological role of this process in influencing cell proliferation and oxygen consumption at the endogenous level. The expression of the C-terminal domain of a GPCR, capable of regulating mitochondrial function, constitutes a hitherto unknown mechanism notably unrelated to its canonical signalling function as a GPCR at the plasma membrane. This work thus highlights a potential novel mechanism thar cells may use for controlling their metabolism under variable environmental conditions, notably as a negative regulator of cell respiration. |

| | |
|---|---|
| **Suggested Reviewers:** | Peter McCormick<br>Professor, Queen Mary University of London<br>p.mccormick@qmul.ac.uk<br>with Expertise in GPCR trafficking and structural biology |
| | David R Sibley<br>section chief, National Institutes of Health<br>sibleyd@ninds.nih.gov<br>with expertise in cell and molecular biology as well as GPCR signaling |
| | Julie Dam<br>Cochin Institute: Institut Cochin<br>julie.dam@inserm.fr<br>with expertise on GPCR and GPCR mitochondrial localization |
| | Eran Segal<br>Professor, Weizmann Institute of Science<br>eran.segal@weizmann.ac.il<br>with expertise on cap-independent translation sequences) |
| | Martine Smit<br>Vrije Universiteit Amsterdam<br>mj.smit@vu.nl<br>with expertise on GPCR biology and pharmacology |
| | Abdussalam Azem<br>Tel Aviv University<br>azema@tauex.tau.ac.il<br>with expertise in Biochemistry, molecular biology and mitochondrial import |
| **Opposed Reviewers:** | Krzysztof Palczewski<br>UC Irvine: University of California Irvine<br><br>competing research |
| | Luca Scorrano |

Powered by Editorial Manager® and ProduXion Manager® from Aries Systems Corporation

| | |
|---|---|
| | University of Pavia: Universita degli Studi di Pavia<br><br>competing research |
| | Marc Von Zastrow<br>UCSF: University of California San Francisco<br><br>Competing research |
| | Dmitry B Veprinstev<br>University of Nottingham<br><br>Competing research |
| **Additional Information:** | |
| **Question** | **Response** |
| **Competing Interests**<br><br>You are responsible for recognizing and disclosing on behalf of all authors any competing interest that could be perceived to bias their work, acknowledging all financial support and any other relevant financial or non-financial competing interests.<br><br>Do any authors of this manuscript have competing interests (as described in the PLOS Policy on Declaration and Evaluation of Competing Interests)?<br><br>**If yes**, please provide details about any and all competing interests in the box below. Your response should begin with this statement: *I have read the journal's policy and the authors of this manuscript have the following competing interests:*<br><br>**If no** authors have any competing interests to declare, please enter this statement in the box: "*The authors have declared that no competing interests exist.*"<br><br>* typeset | The authors have declared that no competing interests exist |

Powered by Editorial Manager® and ProduXion Manager® from Aries Systems Corporation

| | |
|---|---|
| This statement is required for submission and **will appear in the published article** if the submission is accepted. Please make sure it is accurate and that any funding sources listed in your Funding Information later in the submission form are also declared in your Financial Disclosure statement. | |
| **Ethics Statement**<br><br>You must include an Ethics Statement in the Methods section of your manuscript if your study involved:<br>• Human participants<br>• Human specimens or tissue<br>• Vertebrate animals or cephalopods<br>• Vertebrate embryos or tissues<br>• Field research<br><br>**Not including a statement when required will delay the review process.**<br><br>General guidance is provided below. Please consult the guidelines on human subjects research and animal research for detailed instructions. | I confirm that I have read and understood PLOS' requirements for an Ethics Statement. |

*Powered by Editorial Manager® and ProduXion Manager® from Aries Systems Corporation*

| | |
|---|---|
| **Format for specific study types**<br><br>**Human Subject Research (involving human participants and/or tissue)**<br>• Give the name of the institutional review board or ethics committee that approved the study<br>• Include the approval number and/or a statement indicating approval of this research<br>• Indicate the form of consent obtained (written/oral) or the reason that consent was not obtained (e.g. the data were analyzed anonymously)<br><br>**Animal Research (involving vertebrate animals, embryos or tissues)**<br>• Provide the name of the Institutional Animal Care and Use Committee (IACUC) or other relevant ethics board that reviewed the study protocol, and indicate whether they approved this research or granted a formal waiver of ethical approval<br>• Include an approval number if one was obtained<br>• If the study involved *non-human primates*, add *additional details* about animal welfare and steps taken to ameliorate suffering<br><br>**Field Research**<br><br>Include the following details if this study involves the collection of plant, animal, or other materials from a natural setting:<br>• Field permit number<br>• Name of the institution or relevant body that granted permission<br><br>Please check the box to confirm your understanding of this policy, then read and agree to one of the statements. | |
| Please respond:<br>as follow-up to "**Ethics Statement**<br><br>You must include an Ethics Statement in the Methods section of your manuscript if your study involved:<br>• Human participants | My study does not require an ethics statement. |

Powered by Editorial Manager® and ProduXion Manager® from Aries Systems Corporation

| | |
|---|---|
| • Human specimens or tissue<br>• Vertebrate animals or cephalopods<br>• Vertebrate embryos or tissues<br>• Field research<br><br>**Not including a statement when required will delay the review process.**<br><br>General guidance is provided below. Please consult the guidelines on human subjects research and animal research for detailed instructions. | |

Powered by Editorial Manager® and ProduXion Manager® from Aries Systems Corporation

| | |
|---|---|
| **Format for specific study types**<br><br>**Human Subject Research (involving human participants and/or tissue)**<br>• Give the name of the institutional review board or ethics committee that approved the study<br>• Include the approval number and/or a statement indicating approval of this research<br>• Indicate the form of consent obtained (written/oral) or the reason that consent was not obtained (e.g. the data were analyzed anonymously)<br><br>**Animal Research (involving vertebrate animals, embryos or tissues)**<br>• Provide the name of the Institutional Animal Care and Use Committee (IACUC) or other relevant ethics board that reviewed the study protocol, and indicate whether they approved this research or granted a formal waiver of ethical approval<br>• Include an approval number if one was obtained<br>• If the study involved *non-human primates*, add *additional details* about animal welfare and steps taken to ameliorate suffering<br><br>**Field Research**<br><br>Include the following details if this study involves the collection of plant, animal, or other materials from a natural setting:<br>• Field permit number<br>• Name of the institution or relevant body that granted permission<br><br>Please check the box to confirm your understanding of this policy, then read and agree to one of the statements. " | |
| **Data Availability**<br><br>PLOS journals require authors to make all data underlying the findings described in their manuscript fully available, without restriction and from the time of | Yes - all data are fully available without restriction |

| | |
|---|---|
| publication, with only rare exceptions to address legal and ethical concerns (see the PLOS Data Policy and FAQ for further details). When submitting a manuscript, authors must provide a Data Availability Statement that describes where the data underlying their manuscript can be found.<br><br>Your answers to the following constitute your statement about data availability and will be included with the article in the event of publication. **Please note that simply stating 'data available on request from the author' is not acceptable.** ***If, however, your data are only available upon request from the author(s), you must answer "No" to the first question below, and explain your exceptional situation in the text box provided.***<br><br>Do the authors confirm that all data underlying the findings described in their manuscript are fully available without restriction? | |
| Please describe where your data may be found, writing in full sentences. **Your answers should be entered into the box below and will be published in the form you provide them, if your manuscript is accepted.** If you are copying our sample text below, please ensure you replace any instances of XXX with the appropriate details.<br><br>• If your data are all contained within the paper and/or Supporting Information files, please state this in your answer below. For example, "All relevant data are within the paper and its Supporting Information files."<br>• If your data are held or will be held in a public repository, include URLs, accession numbers or DOIs. For example, "All XXX files are available from the XXX database (accession | All source data used to generate charts and tables in the main and supplementary Figures has been provided within a ZIP file to the Journal. The data are archived on the server of the Max-Delbrück-Institute of Molecular Medicine in Berlin and of the University of L'Aquila. All fluorescence microscopy datasets will be made available in high resolution on the IDR: Image Data Resource @ https://idr.openmicroscopy.org upon Publication of the manuscript |

| | |
|---|---|
| number(s) XXX, XXX)." If this information will only be available after acceptance, please indicate this by ticking the box below.<br>• If neither of these applies but you are able to provide details of access elsewhere, with or without limitations, please do so in the box below. For example:<br><br>"Data are available from the XXX Institutional Data Access / Ethics Committee for researchers who meet the criteria for access to confidential data."<br><br>"Data are from the XXX study whose authors may be contacted at XXX."<br><br>* typeset | |
| Additional data availability information: | Tick here if the URLs/accession numbers/DOIs will be available only after acceptance of the manuscript for publication so that we can ensure their inclusion before publication. |

*Powered by Editorial Manager® and ProduXion Manager® from Aries Systems Corporation*

St Andrews, 09.11.2023

Dear Ines,

We have addressed below, and in the revised manuscript, the concerns raised by the reviewer (#2) and the academic editor, as well as the follow up indications you provided. Without further instructions concerning controls mentioned by the academic editor, we have decided against conducting more animal experiments, and worked in providing meaningful controls to the experiments that had already been shown. Most of the material shown in the rebuttal letter to the referees in July is now included in the manuscript, of which we enclose a version in track changes to display our edits. We hope that this addresses any outstanding questions, and we look forward to hearing back from you.

Sincerely,

Roberto and Paolo

---------------------------------------------------------------

Reviewers' comments

Rev. #2 comments:
The authors address the comments reasonably well. I appreciate that the authors inserted the figures into the text which makes the resolution so much better. That alone makes the paper easier to understand.

One comment:

M2R molecular weight, I wouldn't call the insect cell expressed M2R "naked" because they are also glycosylated, but the pattern could be different that from the HEK cell. It could be better that the authors provide the references for that the M2R run at 72kDa. A quick literature search reveals mixed results, some papers show 52 kDa and some show ~72 kDa. I am not 100% convinced that glycosylation can cause a difference as big as 20 kDa in mobility shift.

**We very much thank Reviewer 2 for their encouraging comments following our revision. The M2R expressed using the Promega TNT Coupled Reticulocyte Lysate Systems allows indeed generating a receptor which will have a different set of post-translational modifications than those translated in mammalian cells. Moreover, the running weight for the M2R may depend on whether samples have been boiled or not (as in our case) before running. In their 1995 study published in the Journal of Biological Chemistry, McKinnon and Nathanson demonstrated that Y1 epithelial cells, when heterologously transfected, exhibit a distinct M2R band ranging from 66 to 97 kDa.**

Academic Editor's comments

I still find the manuscript difficult to follow. Both reviewers raised legitimate concerns and while the authors made a real effort to address these, only minor changes were made in the manuscript. Furthermore, many concerns raised by the reviewers regarding controls are not clearly addressed in the rebuttal, making it very difficult to follow the logic. As presented, most of

the westerns do not have appropriate controls, or the controls are scattered in different blots and figure panels. Therefore, to meet the standards of PLOS Biology, the authors need to provide a significantly revised version of their manuscript that not only incorporates appropriate controls for each experiment, but also includes the data they provided to the reviewers in a clear and logical manner.

IA-G: ...I have obtained now more specific comments from the Academic Editor that may help you with the revision. We generally felt that many of the comments present in the rebuttal were not incorporated in the manuscript.

For example, for the answer of Reviewer 1 Point 2 you include four different western blots (Fig. 1-4 for reviewers), but these, as far as we can see, are not present in the figures and should be added.

**We thank the academic editor both for reading the manuscript and providing their comments. We also acknowledge that, despite addressing in full reviewers concerns in the rebuttal letter, we did not incorporate most of these additional control data in the manuscript. We have now corrected this. Specifically:**

1. **By incorporating Western blots from lysates of whole HEK293 expressing the key constructs M2wt-myc, M2tail-myc, as well as myc-M2trunk and myc-M2stop228 (with appropriate loading controls) in Figure S2C and D. Moreover the WB of these constructs was also conducted in COS-7 cells (Figure S2E).**

**We have also added controls to the blot where they were not included:**

2. **In Figure 3C the blot from the mitochondrial fraction now shows also a control using β-actin (no bands) alongside COXIV, to demonstrate the purity of the mitochondrial preparations.**
3. **A β-actin control was also added to the corresponding supernatant/microsomal blot reported in Figure S6K.**

**Concerning Westerns from the transgenic Achm2r-td-tomato mice**

4. **The Western blot of mitochondrial preparation from these mice in Figure 7B includes now -more appropriately- the control with tubulin, to demonstrate the purity of the preparations.**
5. **Finally, positive control of Caspase cleavage requested by the reviewers and provided in our rebuttal, are now included in Figure S10D.**

**Original micrographs from all Western blots are provided separately according to Journal guidelines.**

Similarly, both Reviewers 1 and 2 raised concerns about the distribution of the M2 receptor on Fig. 3B and this needs to be addressed in the manuscript, not only in the rebuttal.

6. **We further expanded the text describing Figure 3B, clarifying that the homogeneous distribution of the 'red' $M_2$receptor arises from the fact that we are imaging the basal membrane of the cell. We feel, and we hope the Academic editor will agree, that including the collection of published examples displayed in Reviewer Figure 5 is at this point not necessary, and we just cited the relevant previous manuscript from our group.**

It is the same case for EM controls, which are shown in Fig. 10 for reviewers, but they are not present in the manuscript – we also worry about the strong EM-gold pattern in the control cells, which is not clear to us if this is background granularity as you mention.

7. **We have added the EM control data as a new panel Figure S7E, and provide now representative zoom-ins to clarify that the apparent 'dark spots' that are visible in the control, likely due to a different exposure settings at the time of imaging, are significantly larger and with less defined shapes as opposed to the circular, sharp and intense gold nanoparticles visible in the transfected samples. We enclose the expanded image (now Figure S7E) below for your appraisal. We have specifically selected in the control one of the most prominent 'dark spots' to emphasize how it does not, nor any of its parts, reflect the sharp circular shape observed when gold nanoparticles are present.**

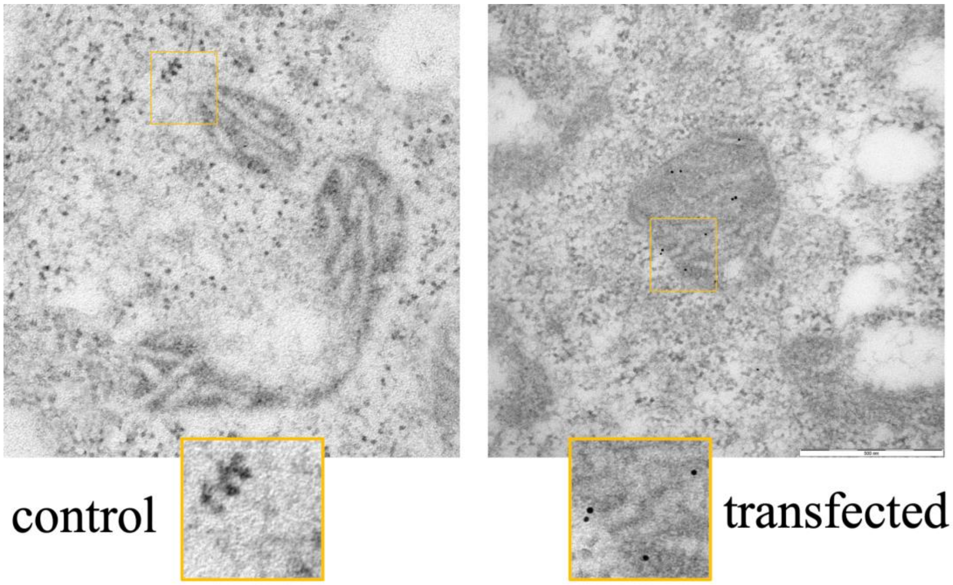


In addition, you also need to include labels for all the panels of the IF images shown in the figures. We might have missed some instances, so please check carefully the rebuttal and make sure that all the information is incorporated to the manuscript.

8. **We apologise for this oversight, and have added labels to the fluorescence microscopy images where they were missing, namely in Figure 5, Figure S6, S7, S8 and S10. This should now better guide the reader to appraise the content of each subpanel.**
9. **the content of each subpanel.**

St Andrews, 09.11.2023

Dear Ines,

We have addressed below, and in the revised manuscript, the concerns raised by the reviewer (#2) and the academic editor, as well as the follow up indications you provided. Without further instructions concerning controls mentioned by the academic editor, we have decided against conducting more animal experiments, and worked in providing meaningful controls to the experiments that had already been shown. Most of the material shown in the rebuttal letter to the referees in July is now included in the manuscript, of which we enclose a version in track changes to display our edits. We hope that this addresses any outstanding questions, and we look forward to hearing back from you.

Sincerely,

Roberto and Paolo

---------------------------------------------------------------

Reviewers' comments

Rev. #2 comments:
The authors address the comments reasonably well. I appreciate that the authors inserted the figures into the text which makes the resolution so much better. That alone makes the paper easier to understand.

One comment:

M2R molecular weight, I wouldn't call the insect cell expressed M2R "naked" because they are also glycosylated, but the pattern could be different that from the HEK cell. It could be better that the authors provide the references for that the M2R run at 72kDa. A quick literature search reveals mixed results, some papers show 52 kDa and some show ~72 kDa. I am not 100% convinced that glycosylation can cause a difference as big as 20 kDa in mobility shift.

**We very much thank Reviewer 2 for their encouraging comments following our revision. The M2R expressed using the Promega TNT Coupled Reticulocyte Lysate Systems allows indeed generating a receptor which will have a different set of post-translational modifications than those translated in mammalian cells. Moreover, the running weight for the M2R may depend on whether samples have been boiled or not (as in our case) before running. In their 1995 study published in the Journal of Biological Chemistry, McKinnon and Nathanson demonstrated that Y1 epithelial cells, when heterologously transfected, exhibit a distinct M2R band ranging from 66 to 97 kDa.**

Academic Editor's comments

I still find the manuscript difficult to follow. Both reviewers raised legitimate concerns and while the authors made a real effort to address these, only minor changes were made in the manuscript. Furthermore, many concerns raised by the reviewers regarding controls are not clearly addressed in the rebuttal, making it very difficult to follow the logic. As presented, most of

the westerns do not have appropriate controls, or the controls are scattered in different blots and figure panels. Therefore, to meet the standards of PLOS Biology, the authors need to provide a significantly revised version of their manuscript that not only incorporates appropriate controls for each experiment, but also includes the data they provided to the reviewers in a clear and logical manner.

IA-G: ...I have obtained now more specific comments from the Academic Editor that may help you with the revision. We generally felt that many of the comments present in the rebuttal were not incorporated in the manuscript.

For example, for the answer of Reviewer 1 Point 2 you include four different western blots (Fig. 1-4 for reviewers), but these, as far as we can see, are not present in the figures and should be added.

**We thank the academic editor both for reading the manuscript and providing their comments. We also acknowledge that, despite addressing in full reviewers concerns in the rebuttal letter, we did not incorporate most of these additional control data in the manuscript. We have now corrected this. Specifically:**

1. **By incorporating Western blots from lysates of whole HEK293 expressing the key constructs M2wt-myc, M2tail-myc, as well as myc-M2trunk and myc-M2stop228 (with appropriate loading controls) in Figure S2C and D. Moreover the WB of these constructs was also conducted in COS-7 cells (Figure S2E).**

**We have also added controls to the blot where they were not included:**

2. **In Figure 3C the blot from the mitochondrial fraction now shows also a control using β-actin (no bands) alongside COXIV, to demonstrate the purity of the mitochondrial preparations.**
3. **A β-actin control was also added to the corresponding supernatant/microsomal blot reported in Figure S6K.**

**Concerning Westerns from the transgenic Achm2r-td-tomato mice**

4. **The Western blot of mitochondrial preparation from these mice in Figure 7B includes now -more appropriately- the control with tubulin, to demonstrate the purity of the preparations.**
5. **Finally, positive control of Caspase cleavage requested by the reviewers and provided in our rebuttal, are now included in Figure S10D.**

**Original micrographs from all Western blots are provided separately according to Journal guidelines.**

Similarly, both Reviewers 1 and 2 raised concerns about the distribution of the M2 receptor on Fig. 3B and this needs to be addressed in the manuscript, not only in the rebuttal.

6. **We further expanded the text describing Figure 3B, clarifying that the homogeneous distribution of the ‘red’ $M_2$receptor arises from the fact that we are imaging the basal membrane of the cell. We feel, and we hope the Academic editor will agree, that including the collection of published examples displayed in Reviewer Figure 5 is at this point not necessary, and we just cited the relevant previous manuscript from our group.**

It is the same case for EM controls, which are shown in Fig. 10 for reviewers, but they are not present in the manuscript – we also worry about the strong EM-gold pattern in the control cells, which is not clear to us if this is background granularity as you mention.

7. **We have added the EM control data as a new panel Figure S7E, and provide now representative zoom-ins to clarify that the apparent 'dark spots' that are visible in the control, likely due to a different exposure settings at the time of imaging, are significantly larger and with less defined shapes as opposed to the circular, sharp and intense gold nanoparticles visible in the transfected samples. We enclose the expanded image (now Figure S7E) below for your appraisal. We have specifically selected in the control one of the most prominent 'dark spots' to emphasize how it does not, nor any of its parts, reflect the sharp circular shape observed when gold nanoparticles are present.**

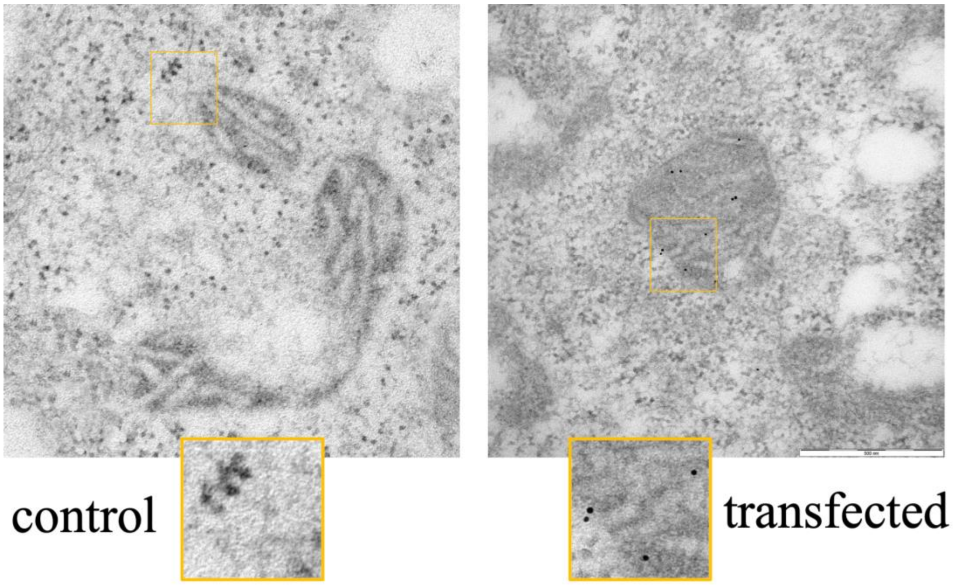


In addition, you also need to include labels for all the panels of the IF images shown in the figures. We might have missed some instances, so please check carefully the rebuttal and make sure that all the information is incorporated to the manuscript.

8. **We apologise for this oversight, and have added labels to the fluorescence microscopy images where they were missing, namely in Figure 5, Figure S6, S7, S8 and S10. This should now better guide the reader to appraise the content of each subpanel.**

Manuscript

Click here to access/download;Manuscript;IRES_manuscript_09112023_clean

# One gene - two proteins: The C-terminus of the prototypical M2 muscarinic receptor localizes to the mitochondria and regulates cell respiration.

Irene Fasciani[1†], Francesco Petragnano[1†], Ziming Wang[2], Ruairidh Edwards[3], Narasimha Telegu[2], Ilaria Pietrantoni[1], Ulrike Zabel[4], Henrik Zauber[2], Marlies Grieben[2], Maria E. Terzenidou[3], Jacopo Di Gregorio[1], Cristina Pellegrini[1], Silvano Santini Jr[5], Anna R. Taddei[6], Barbel Pöhl[2], Stefano Aringhieri[7], Marco Carli[7], Gabriella Aloisi[1], Francesco Marampon[8], Eve Charlesworth[9], Alexandra Roman[2], Sebastian Diecke[2], Vincenzo Flati[1], Franco Giorgi[7], Fernanda Amicarelli[5], Andrew B. Tobin[3], Marco Scarselli[7], Kostas Tokatlidis[3], Mario Rossi[1], Martin J. Lohse[2,4,10]*, Paolo Annibale[2,9]*, Roberto Maggio[1]*

[1] Department of Biotechnological and Applied Clinical Sciences, University of L'Aquila, 67100 L'Aquila, Italy.

[2]Max Delbrück Center for Molecular Medicine, 13125 Berlin, Germany.

[3]Centre for Translational Pharmacology, Institute of Molecular, Cell and Systems Biology, College of Medical, Veterinary and Life Sciences, University of Glasgow, G128QQ Glasgow, UK.

[4]Institute of Pharmacology and Toxicology, University of Würzburg, 97078 Würzburg, Germany.

[5]Department of Life, Health and Environmental Sciences, University of L'Aquila, 67100 L'Aquila, Italy.

[6]Section of Electron Microscopy, Great Equipment Center, University of Tuscia, Viterbo, Italy.

[7]Department of Translational Research and New Technology in Medicine, University of Pisa, 56126 Pisa, Italy.

[8]Department of Radiotherapy, University of Roma La Sapienza, 00151 Roma, Italy.

[9]School of Physics and Astronomy, University of St Andrews, KY16 9SS St Andrews.

[10]ISAR Bioscience Institute, 82152 Munich, Germany.

† Equal contribution

*email: martin.lohse@isarbioscience.de;

*email: roberto.maggio@univaq.it;

*email: paolo.annibale@mdc-berlin.de, pa53@st-andrews.ac.uk

## Abstract

**Muscarinic acetylcholine receptors are prototypical G protein-coupled receptors (GPCRs), members of a large family of seven transmembrane receptors mediating a wide variety of extracellular signals. We show here, for the first time in a GPCR, that the carboxyl terminal fragment of the muscarinic $M_2$ receptor, comprising the transmembrane regions 6 and 7 ($M_2$tail), is expressed by virtue of an internal ribosome entry site located in the third intracellular loop. Single cell imaging reveals that $M_2$tail, whose expression is upregulated in cells undergoing integrated stress response, does not follow the normal route to the plasma membrane, but is almost exclusively sorted to the mitochondria inner membrane: here it controls oxygen consumption, cell proliferation and the formation of reactive oxygen species by reducing oxidative phosphorylation. Crispr/Cas9 editing of the key methionine where cap-independent translation begins in human induced pluripotent stem cells, reveals the physiological role of this process in influencing cell proliferation and oxygen consumption at the endogenous level. The expression of the C-terminal domain of a GPCR, capable of regulating mitochondrial function, constitutes a hitherto unknown mechanism notably unrelated to its canonical signalling function as a GPCR at the plasma membrane. This work thus highlights a potential novel mechanism thar cells may use for controlling their metabolism under variable environmental conditions, notably as a negative regulator of cell respiration.**

## Introduction

Muscarinic receptors include five structurally related receptors, belonging to class A of the G protein-coupled receptor (GPCR) family [1]. They are comprised of seven transmembrane domains (TM domains 1-7) connected by intra and extracellular loops. Of these structural elements, the third intracellular (i3) domain is the one conferring a coupling specificity for G proteins, β-arrestin recruitment and endocytic internalization [2]. However, only a small portion of the i3 loop is required for receptor function, and for this reason large deletions of this segment have no effect on receptor activation and internalization [3].

GPCRs are normally distributed at the plasma membrane, where, after activation by an agonist and coupling to either G proteins or β-arrestin, regulate a number of intracellular effectors. Nevertheless, non-canonical localizations of GPCRs have also been described, such as in

endosomes [4],  cell division compartments (centrosomes, spindle midzone and midbodies) [5] and mitochondria [6], underpinning non-traditional roles for this protein family.
Several studies have shown that the GPCR structurally related transmembrane protein bacteriorhodopsin consists of autonomous folding domains that translocate to the membrane and interact with each other to function as a proton pump.[illegible]nterestingly, it was also shown that fragments of muscarinic receptors can interact to form functionally active chimeric receptors, highlighting the conserved evolutionary ability of domains from these receptor family to interact among each other, as well as with other proteins.[illegible]ntriguingly, in 2016, we observed that the insertion of a stop codon in the third intracellular loop of the muscarinic $M_2$ receptor was not able to abolish its function [7], presenting the suggestive possibility that a cap-independent translation of the $M_2$ C-terminal domain may occur in this mutant, i.e. that the $M_2$ mRNA may lead to the translation not only of the full length receptor, but also of its C-terminal domain, that we name here $M_2$tail.

In this work, we therefore set out to explore whether the expression of the $M_2$tail, a region containing the TM regions 6 and 7, occurs naturally by a cap-independent translation mechanism. Combining radio-ligand binding, molecular biology approaches, advanced fluorescence imaging and Crispr/Cas9 genetic editing of human induced pluripotent stem cells (hiPSCs) we have gathered compelling data indicating a novel translation mechanisms for C-terminal domain of the $M_2$ muscarinic receptor. This translation mechanism appears to be regulated by an internal ribosome entry site (IRES) sequence within the M2 mRNA allowing the independent expression of the receptor's C-terminal domain as a distinct polypeptide.

Moreover, by using bimolecular fluorescence complementation, in-vitro mitochondrial import and cell metabolism assays we observe that $M_2$tail is strongly targeted to the cell mitochondria. Importantly, this mitochondrial targeting has functional ramifications since both cap-dependent mediated overexpression of $M_2$tail, as well as cap-independent mediated translation starting from the mRNA of the full length $M_2$ receptor, resulted in a sizable reduction of oxidative phosphorylation. In fact, we show that cellular stress favours the activation of this newly discovered IRES-dependent translation of $M_2$tail and that the fragment interacts with the Complex V of the respiratory chain on the mitochondria inner membrane. The implication of this newly discovered regulatory mechanism of the IRES mediated translation of the $M_2$ C-terminal domain might have profound physiological and pathophysiological implications.

In summary our study reports the new observation that alternative reading of a GPCR receptor gene can translate into a fragment of the receptor that serves a cellular function entirely different from canonical GPCR signalling**.** Taken together these data strongly suggest that the $M_2$ mRNA yields not only a plasma membrane receptor able to transduce many of the acetylcholine functions but $M_2$tail, a protein fragment that reduces the activity of mitochondria and ROS accumulation and it might represent a hitherto unappreciated step for in-vivo anti-oxidative stress, during for instance reperfusion, tissue damage, and disease-promoted metabolic cellular stress.

## Results

### Cap independent production of the C-terminus of the $M_2$ receptor and evidence for an IRES

We began our work by testing our earlier hypothesis that the last two TM regions (TM6-7) of $M_2$stop228, a $M_2$ muscarinic receptor mutant bearing a stop codon at the beginning of the i3 loop in position 228 (**Fig. S1**), may still be translated [10]. We thus generated EGFP C-terminal fusion of the $M_2$ wt receptor and $M_2$stop228: **Fig. 1A** shows that cells expressing $M_2$stop228-EGFP display a green fluorescence signal. Moreover, the signal displays a predominant intracellular localization with respect to the wt $M_2$R, which instead localizes -as expected- largely at the plasma membrane. A key observation is that the specific location of the stop codon upstream of the i3 loop appears to have particular relevance. For istance, if a stop is instead inserted downstream of position 228, e.g. at positions 400, right before the first methionine in TM6, no fluorescence can be observed in cells expressing the mutant $M_2$stop400-EGFP **Fig. 1A.**

To further appreciate the origin of this unexpected process -the 228 stop codon should in principle abolish any EGFP signal- we performed radioligand binding experiments in cells transfected with this mutant. $M_2$stop228 exhibited $K_D$ value for the antagonist [$^3$H]NMS (N-methylscopolamine) calculated from direct saturation experiments, as well as $IC_{50}$ values, obtained in competition experiments for the agonist carbachol, similar to those calculated for the wild type $M_2$ receptor (**Table 1**), albeit displaying a very low level of binding sites at the plasma membrane, specifically 45 fmol/mg of protein. Furthermore, $M_2$stop228 was found functionally active as it was capable to inhibit adenylyl cyclase [10] and increase ERK phosphorylation (**Fig. S2A**), even though the extent of response was reduced if compared to the wild type $M_2$ receptor (**Table 1)**. As a control,

receptor fragments, translated from mRNAs comprising only the sequence for TM1-5 (i.e. whose sequences run only until position 228 or 283 of the corresponding full length receptor), called $M_2$trunk(1-228) or $M_2$trunk(1-283), did not show any radio-ligand binding activity, despite coding for the same first five trans-membrane regions (TM1-5) that the canonical translation of $M_2$stop228 should also produce (**Fig. 1B** and **Table 1**) [11].

Notably, our previous research has demonstrated the ability of distinct fragments of muscarinic receptors, cotransfected together, to reassemble into a fully functionally receptors. Specifically, we showed that the last two TMs (TM6-7) of the $M_2$ receptor can combine with TM1-5 to recreate a functional receptor [11] (**Table 1**), supporting the notion that the explanation of the apparent paradox of $M_2$stop228 lies in the independent translation of $M_2$tail as a distinct polypeptide, as graphically summarized in **Fig. 1B**.

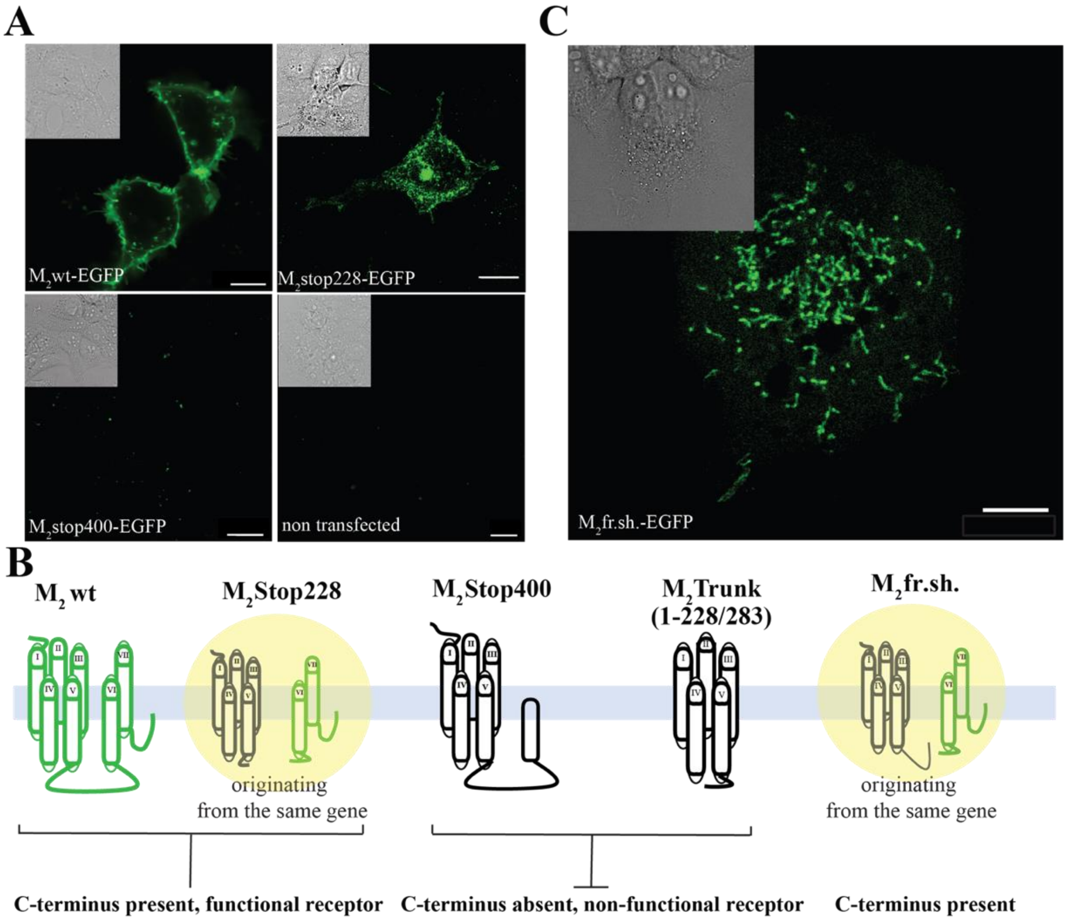

**Fig. 1. The c-terminus of the $M_2$ receptor is expressed by a cap independent translation mechanism, originating in the i3loop.**

**A**, Fluorescence micrographs of HEK293 and COS-7 cells respectively expressing $M_2$-EGFP, $M_2$stop228-EGFP, $M_2$stop400-EGFP, together with an untransfected control. **B**, Schematic representation of the gene products that reach the plasma membrane of the wild type $M_2$, $M_2$stop228, $M_2$trunk(1-283) and $M_2$fr.sh. (**Fig. S3**). **C**, Fluorescence micrograph of a representative HEK293 cell expressing $M_2$fr.sh.-EGFP. Insets display DIC image of the same field of view (scale bar 10 µm). Imaging settings are the same for the f images, whereas contrast was adjusted to improve visualization. In the untransfected cells and stop400 panels, the contrast was set to 0. Insets display DIC image of the same field of view (scale bars 10 µm).

| Binding data | | | | Adenylyl cyclase inhibition assay | |
|---|---|---|---|---|---|
| Receptor | Bmax (fmol/mg) | [$^3$H]NMS KD (pM) | Carbachol IC50 (µM) | Carbachol EC50 (µM) | Maximum inhibition of forskolin stimulated cAMP accumulation (%) |
| $M_2$ | 980 ± 60 | 62 ± 7 | 2.1 ± 0.6 | 0.04 ± 0.01 | 53 ± 5 |
| $M_2$trunk(1-283) | N.B. | | | | |
| $M_2$trunk(1-228) | N.B. | | | | |
| $M_2$stop228 | 45 ± 2 | 70 ± 10 | 1.8 ± 0.4 | 0.13 ± 0.02 | 27 ± 3 |
| M2trunk(1-283) +M2tail(M-281-466) | 480 ± 20 | 79 ± 10 | 0.7 ± 0.2 | 0.15 ± 0.03 | 37 ± 4 |
| M2trunk(1-228) +M2tail(368-466) | 70 ± 10 | 72 ± 8 | 0.6 ± 0.2 | 0.17 ± 0.03 | 29 ± 4 |

**Table 1. Radioligand binding and activation properties of $M_2$ receptor mutants.**

Binding properties and inhibition of forskolin stimulated adenylyl cyclase by $M_2$, $M_2$stop228, $M_2$trunk(1-228), $M_2$trunk(1-283), and cotransfected M2trunk(1-283) + M2tail(M-281-466) and. M2trunk(1-228) + M2tail(368-466). Untransfected COS-7 cells did not show any [$^3$H]NMS specific binding. N.B. = no specific [$^3$H]NMS binding. Data are averages with s.e.m.

## Evidence of an IRES-mediated $M_2$ C-terminus translation

$M_2$stop228 mRNA appears thus able to give rise -despite the presence of a stop codon in the i3 loop- to two distinct protein products (a *trunk* TM1-5 and a *tail* TM6-7), in turn able to reconstitute a functional receptor at the plasma level.

To challenge this hypothesis and gain further insight into the mechanism at play, we generated a frame shift mutant of the $M_2$ receptor: we inserted a single guanine nucleotide in the sequence of the $M_2$-EGFP, changing the methionine in position 368 into an aspartate, which in turn generates an in-frame stop codon at position 370 (**Fig. S1 and Fig. S3)**. The observation that cells transfected with $M_2$fr.sh.-EGFP retain green fluorescence is most remarkable (**Fig. 1C**), since the EGFP is now out of the original reading frame and it can only be properly translated by a *cap-independent translation mechanism*, starting from a methionine in the new reading frame.

Furthermore, the observation that both $M_2$stop228-EGFP and $M_2$fr.sh.-EGFP retain green fluorescence, whereas $M_2$stop400-EGFP does not, indicates not only that a cap independent translation mechanism occurs, but also that the starting methionine for the process must be downstream of stop 228 and upstream of stop 400, leaving as a leading candidate one of the in-frame methionines of the i3 loop.

To narrow this down, we employed radio-ligand binding data from expression of receptor mutants carrying a double stop codon, the first in position 228 and the second in positions ranging from 248 to 400, including mutations of the first (M248) and second (M296) in-frame methionines in the third i3 loop (**Table S1**, **Fig. S3)**. The observation that mutation of the first two methionines does not affect $B_{max}$ points to M368 as the most likely site for the cap-independent initiation of translation of the $M_2$tail, that we now term more specifically $M_2$tail(368-466). These binding results can furthermore rule out read-through and termination re-initiation: if these mechanisms were responsible for translation of the C-terminal of the $M_2$stop228, suppression of the first methionine would have abolished the binding. We shall further note that cells expressing $M_2$stop228 showed only one single $M_2$ specific cDNA band supporting the concept that the $M_2$tail(368-466) was produced from the same $M_2$-mRNA molecule by a cap independent mechanism instead of, for instance, by RNA splicing variants (**Supplementary Materials and Methods** and **Fig. S2B)**. However, upon immunoblotting the protein products it is immediate to observe that the full length $M_2$ receptor produces a set of low molecular weight band matching those observed for $M_2$tail(368-466), when the constructs posses a C-terminal tag (myc) (**Fig. S2C**[illegible]n contrast N-terminally tagged $M_2$stop228 produces a band in line with that observed for cells transfected with the

$M_2$trunk(1-228) fragment, further ruling out stop-cordon readthrough for the our findings (**Fig. S2D)**. Comparable results are observed in COS-7 cells (**Fig. S2E)**.

**IRES position within the $M_2$ coding sequence**

To further validate the results obtained working with full length $M_2$ receptor mutants and to investigate the critical nucleotides for IRES activity, we generated a fluorescence reporter construct (Sirius-$M_2$i3(417n)-EGFP) in which the i3 loop of $M_2$ was inserted between the two fluorescent proteins, Sirius and EGFP (**Fig. 2A)**. The sequence for Sirius is followed by a stop codon, which means that Sirius-$M_2$i3(417n)-EGFP translates Sirius according to the classical cap-dependent mechanism, serving as an indicator of transfection efficiency, and, at the same time, drives EGFP translation only if the $M_2$i3 loop contains an IRES sequence, essentially functioning as a bicistronic expression cassette. We transfected several cell lines with this construct, and all of them were positive for the blue and green fluorescence **(Fig. S4A**[illegible]EK293 cells, used throughout our study, appeared positive for both the blue and green fluorescence, as shown in the inset of **Fig. 2A** and in line with the results reported in **Fig. 1A**. To further rule out the possibility that EGFP translation from the Sirius-$M_2$i3(417n)-EGFP construct could be attributable to read-through and termination-re-initiation mechanisms, a mRNA hairpin loop was inserted in the sequence after the stop codon of the Sirius protein and before the $M_2$i3(417n), resulting in Sirius-H-$M_2$i3(417n)-EGFP. This modification did not eliminate the EGFP signal, showing only a slight reduction of the EGFP/Sirius with respect to the value measured for the analogous reporter without hairpin (~90%) (**Fig. 2A**), underscoring that read-through and termination-re-initiation mechanisms do not appear to be at play. In contrast, when the $M_2$i3(417n) segment was removed from Sirius-H-$M_2$i3(417n)-EGFP, yielding Sirius-H-EGFP, it entirely abolished EGFP expression (**Fig. 2A**). This observation indicates that the hairpin loop effectively blocks read-through and termination-re-initiation mechanisms

To narrow down the position of the IRES sequence we divided the $M_2$ i3 loop in three parts and the resulting nucleotide sequences, 685-699, 685-1071 and 685-699/1072-1101, were inserted downstream of the hairpin loop in three different constructs (**Fig. 2A** and **Fig. S3)**.

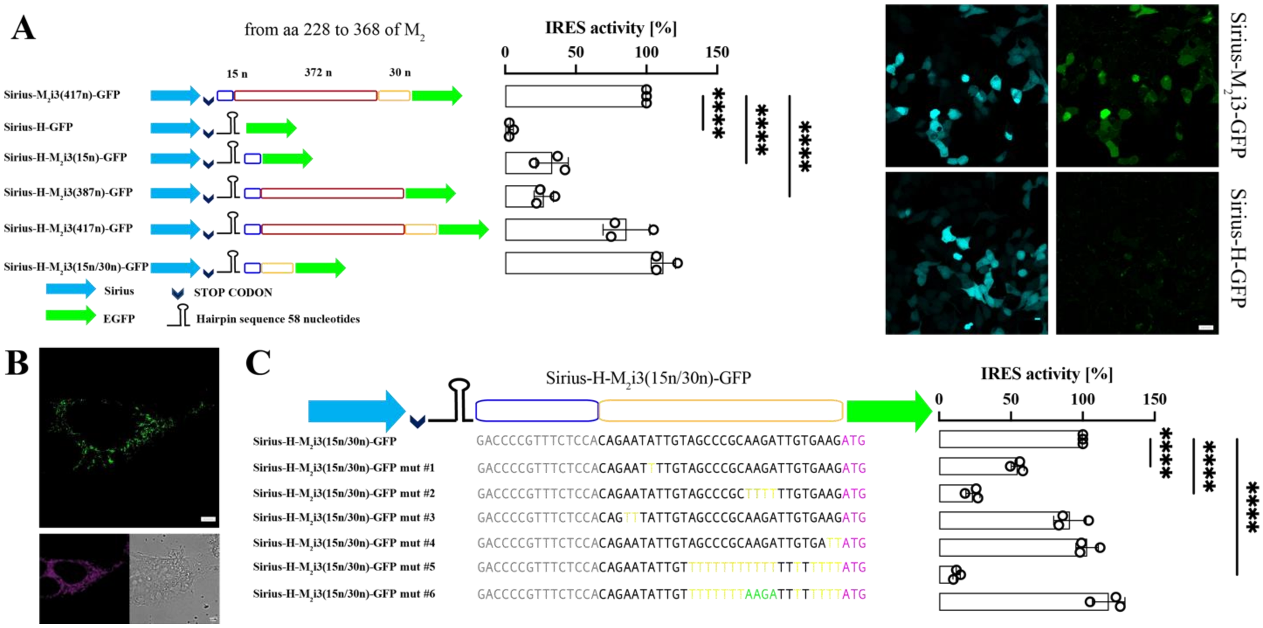


**Fig. 2. Localization of the IRES within the i3 loop the muscarinic $M_2$ receptor using a fluorescence reporter assay.**

**A**, **left** Schematic representation of bicistronic plasmids bearing the $M_2$ i3 loop between the coding regions of the Sirius and EGFP fluorescent proteins. A guanine nucleotide was originally inserted before the EGFP in the plasmid Sirius-H-EGFP in order to maintain in frame the sequences of the two fluorescent proteins. In all the other plasmids the sequence of the two fluorescent proteins is not in frame. Fifteen nucleotides derived from the $M_2$ i3 loop were inserted between the end of Sirius and the hairpin loop, in order to allow the easy scanning of the ribosome across the stop codon. **right**, IRES activity, highlighting the efficiency of cap-independent translation for the control Sirius-$M_2$i3(417n)-EGFP and the six mutants. The insert shows the difference in the green fluorescence between two of the mutants. Significance values reported in the graphs were determined by a one-tailed Student t-test, p-values: ** $0.001<p<0.01$. **B,** Expression of the construct $M_2$-Hairpin-EGFP in HEK293 cells: (top) Confocal images of $M_2$-Hairpin-EGFP, Mitotracker Deep red staining and DIC (bottom). Scale bar 5 µm. **C**, **left** Mutations of the bicistronic Sirius-H-$M_2$i3(15n/30n)-EGFP plasmids in the region of the $M_2$ i3 loop from nucleotides 1972 to 2001, in yellow are the modified nucleotides. In Sirius-H-$M_2$i3(15n/30n)mut#6-EGFP the four nucleotides in green (AAGA) were reinserted in the stretch of 20 T of Sirius-H-$M_2$i3(15n/30n)mut#5-EGFP. **right** IRES activity, highlighting the efficiency of cap-independent translation for the control Sirius-H-$M_2$i3(15n/30n)-EGFP and the six mutants illustrated. Significance ($p < 0.01$) was calculated in **A** against Sirius-$M_2$i3(417n)-EGFP and in **B** against Sirius-H-$M_2$i3(15n/30n)-EGFP. Error bars are s.e.m. Significance values reported in the graphs were determined by a one-tailed Student t-test, p-values: ** $0.001<p<0.01$.

Only, when the last portion of the $M_2$ i3 loop was inserted in the construct after the hairpin, leading to Sirius-H-$M_2$i3(15n/30n)-EGFP, ~110% of the value measured for the Sirius-$M_2$i3(417n)-EGFP plasmid was recovered (**Fig. 2A**). Notably, when the hairpin was inserted within the i3 loop of the full length $M_2$-EGFP, upstream of the M368, it was still possible to observe green fluorescence in transfected cells (**Fig. 2B)**.

These results confirm that the IRES is located in a region of the i3 loop sequence immediately upstream of the third methionine (M368). This is also confirmed from ribosome profiling data from human left ventricle tissue, displaying increased p-sites as well as overall ribosomal coverage in correspondence of this region (**Fig. S4B**). Based on this evidence, we mutated the conserved residues highlighted in yellow in **Fig. S4C,** in order to check for their relevance in recruiting the ribosome on the $M_2$ i3 loop sequence, as shown in **Fig. 2C**. As discussed in detail in the **Supplementary Materials**, we found that the four nucleotides AAGA1090-1093 next to the methionine 368 play a pivotal role in recruiting the ribosome on the $M_2$ mRNA and in translating the $M_2$ C-terminal fragment.

**Generalization to other members of the GPCR superfamily**

In order to determine if the observed behavior is specific to the $M_2$ receptor or could highlight a feature common to other members of the GPCR superfamily, we repeated several of our experiments in another GPCR, the related muscarinic $M_3$ receptor. The $M_3$ displays an in-frame methionine at the end of the i3loop, in position 481. We then inserted a stop codon in position 273, generating the construct $M_3$stop273 that still displayed radio-ligand binding and functional activity (**Table S2**). Several other mutants, mimicking the single and double stop mutations made for the $M_2$ displayed a behavior consistent with M481 being the start point of a cap-independent translation of the C-terminal also for the $M_3$ receptor (**Table S2)**, thus indicating that IRES-mediated cap-independent translation of the C-terminal domain may not be an exclusive phenomenon confined solely to the $M_2$ receptor, but may be a shared characteristic among some other members of the GPCR superfamily. The presence of an IRES sequence upstream of M481 further appears to be supported by the observation of GFP fluorescence when 558 nucleotides from the i3 loop of the $M_3$ receptor (from nucleotide 880 to 1437) were inserted into the identical Sirius-EGFP reporter construct used for the $M_2$ receptor (**Fig. S5B**[illegible]urthermore, the sequence alignment of the last portion of the $M_2$ i3 loop (from nucleotide 1072 to 1104) with regions adjacent to the second ($M_1$), fourth ($M_3$ and $M_4$) and first ($M_5$) in-frame Methionines, clearly shows that

some nucleotide residues are conserved (**Fig. S4C)**. Overall, these findings underline that cap-independent translation could be a more general feature of GPCRs mRNAs.

**Mitochondrial localization of the $M_2$ C-terminal fragment generated by cap-dependent expression under the control of a viral promoter**

Having established that the expression of the $M_2$ C-terminal domain is regulated by an IRES and that its translation starts with M368, we proceeded to investigate the role it might play in the cell. For this purpose, we fused $M_2$tail(368-466) with EGFP in pcDNA3, that is under the control of a strong CMV promoter, and we expressed it in HEK293 cells in order to study its subcellular localization. Confocal microscopy revealed a remarkable mitochondrial localization of the $M_2$tail(368-466)-EGFP construct (**Fig. 3A**).

We confirmed this observation by co-staining HEK293 cells with a conventional mitochondrial stain and by co-expressing $M_2$tail(368-466)-EGFP with Tom20-mCherry, a marker for the outer mitochondrial membrane (**Fig. S6A**). The observed mitochondrial localization of $M_2$tail(368-466)-EGFP appeared in stark contrast to the canonical cellular localization of the $M_2$ receptor, as shown in **Fig. 3B**, a confocal section at the level of the basal membrane of a cell co-transfected with the $M_2$ muscarinic receptor C-terminally labeled with the red fluorescent protein mRuby2. As expected, and in agreement with what is observed for several other GPCRs[12], the $M_2$ muscarinic receptor expression is homogeneous at the basal membrane, as opposed to the localized signal of the subplasmalemmal mitochondria.

In line with the radioligand binding data shown in **Table 1**, very little expression of $M_2$tail(368-466)-EGFP was observed on the plasma membrane, as we confirmed by fluorescence correlation spectroscopy (FCS) [13] (**Figs S6B-D**). In contrast, when the $M_2$tail(368-466)-EGFP was co-expressed with $M_2$stop228an increased localization could be seen on the cell membrane, suggesting a chaperon activity of the $M_2$ N-terminal domain ($M_2$trunk) (**Figs S6E-G**) and providing an explanation for the data obtained in radioligand binding studies and reported in **Table 1**. Likewise, constructs such as $M_2$tail(368-466)-mRuby2 (a red monomeric fluorescent protein) and $M_2$tail(368-466)-tdTomato (a tandem-dimer red fluorescent protein) also displayed clear mitochondrial localization (**Figs S6H-I**), ruling out bias due to the fluorophore used. We obtained comparable findings also in COS-7 cells (**Fig. S6J**).

Mitochondrial import of the C-terminal fragment of the $M_2$ receptor was further verified by immunoblotting of protein extracts from purified mitochondria of HEK293 cells transfected with myc-tagged $M_2$ receptor, $M_2$tail(368-466) and $M_2$M368A, a mutant where the key methionine for the cap-independent translation of the C-terminus is changed to an Alanine. **Fig. 3C** shows that

in purified mitochondria (unlike in the cytosolic and microsomal supernatants originating from the purification process, **Fig. S6K)**, only the protein bands associated to the $M_2$ C-terminal fragment were visible. Notably, no mitochondrial import was visible for the $M_2$M368A, confirming that the third in-frame methionine (M368) in the i3 loop of the $M_2$ receptor is necessary for the C-terminal fragment production and its localization to the mitochondria. The slight offset between the lower bands in the $M_2$ wt and $M_2$tail(368-466) lanes matches exactly the different length of the linker used to fuse the myc-tag in the two constructs.

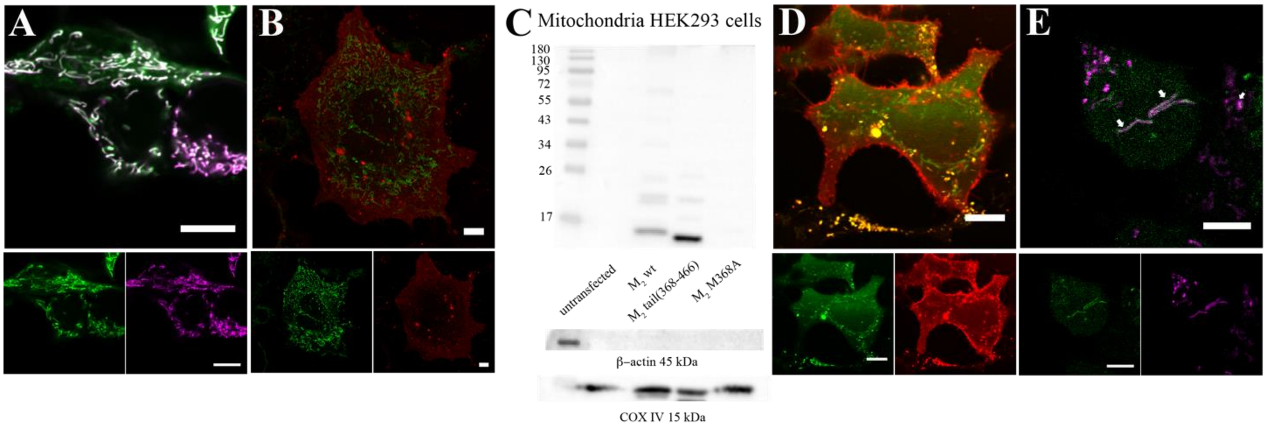


**Fig. 3. $M_2$tail(368-466) predominantly localizes to the cell mitochondria**

**A**, Colocalization of $M_2$tail(368-466)-EGFP (green) together with the mitochondrial targeted dye Mitotracker (magenta) in HEK293 cells. Separate spectral channels are displayed below. **B**, Co-expression of $M_2$tail(368-466)-EGFP (green) and wild type $M_2$-mRuby2 (red) in HEK293 cells. Separate spectral channels are displayed below. **C**, Western blot (10% TRIS-Glycine PAA gel) of mitochondria purified from Hek293 cells transfected with the $M_2$-myc, $M_2$tail(368-466)-myc, $M_2$M368A-myc, together with an untransfected control are blotted using an anti-myc antibody. Loading control was verified by immunoblotting COXIV and β-actin. **D**, Cellular localization of the construct $M_2$-mRuby2-STOP-$M_2$-i3-tail-EGFP in HEK293 cells. Expression of the $M_2$tail(368-466)-EGFP (green) after the STOP codon is an indicator of the endogenous IRES activity. While $M_2$-mRuby2 localizes correctly and predominantly to the cell plasma membrane, $M_2$tail(368-466)-EGFP localizes intracellularly. Separate spectral channels are displayed below. **E**, $M_2$-fr.sh.-EGFP (green) expressed in conjunction with Mitotracker (magenta). The EGFP is expressed despite the creation of an in-frame stop after M368, confirming endogenous cap-independent translation of the $M_2$tail(368-466). Arrows indicate areas of colocalization with the mitochondria. Separate spectral channels are displayed below.

## Localization of the $M_2$ C-terminal fragment generated by cap-independent expression under the control of the i3loop IRES

We then sought to express the $M_2$tail under the control of the i3loop IRES rather than a viral promoter, to further exclude the possibility that the observed mitochondrial localization of the $M_2$ carboxyl terminal fragment were due to overexpression of the construct. HEK293 cells were transfected with the construct $M_2$-mRuby2-STOP-$M_2$-i3-tail-EGFP which contains the full wild type $M_2$ receptor, labeled at the C-terminal with mRuby2, additionally fused to the C-terminal part of the $M_2$ receptor starting at nucleotide 685 of the i3 loop, following the stop codon of mRuby2, and ending with a C-terminal EGFP sequence (**Fig. S3**). Through this experimental strategy, the expression of $M_2$tail(368-466)-EGFP is regulated by the IRES, and C-terminal fragment production is therefore close to physiological expression. The expression of EGFP confirmed that the *in vivo* production of the $M_2$tail(368-466) is regulated by the IRES within the i3 loop. In addition, the processed receptor fragment was eventually observed to be predominantly confined to the cytosol and the mitochondrial network (**Fig. 3D**).

Notably, when the $M_2$-EGFP fusion protein was expressed and the cells were co-stained with Mitotracker, a faint EGFP signal was also observed in the mitochondrial regions (**Fig. S7A**), although the expression of the full-length receptor masks this effect and makes quantification difficult. On the other hand, **Fig. 3E** shows that, when the $M_2$fr.sh-EGFP mutant was expressed in HEK293 cells, the IRES-generated C-terminal fragment of the $M_2$ receptor localized to the mitochondria. As mentioned above, in this construct the masking effect of the EGFP fused to the full-length receptor is eliminated (see also **Fig. S3**). Similar mitochondrial localization was also observed when we transfected HEK293 cells with $M_2$fr.sh-mRuby2 (**Fig. S7B**), ruling out possible effects due to the specific fluorophore used as a tag. Again, comparable localization patternswere observed when the constructs were expressed in a different cell type, such as the H9c2 rat cardiomyoblast cell lines (**Fig. S7C**).

**Mechanism of $M_2$tail import into the mitochondria and localization within the organelle**

To gain further insight into the dynamics and mechanisms of the $M_2$ C-terminal fragment import within the mitochondria, we first monitored the kinetics of colocalization between mitochondria and EGFP in cells transfected with $M_2$tail(368-466)-EGFP. In cells observed for several hours, starting at 5h after transfection (**Fig. 4A)**, the construct was found initially expressed in the perinuclear region, likely the endoplasmic reticulum, indicating that at least the initial step of subcellular trafficking of the $M_2$tail(368-466)-EGFP is the same as for the full length receptor [14]. At this time (5h post-transfection), almost no colocalization with the Mitotracker-stained mitochondria was visible. However, at 6h post-transfection, the colocalization level increased significantly, and $M_2$tail(368-466)-EGFP was either found in the cytosol or in the mitochondria. At

9h post-transfection the colocalization at the mitochondria was well visible (see **Materials and Methods**) and we could observe that almost half of the mitochondrial import occurred within 6h post-transfection (**Fig. 4B)**.

This value is qualitatively in agreement with what we observed using an in-vitro assay to determine the import of the $M_2$ carboxyl terminal into isolated mitochondria (see **Materials and Methods**). To this end, we used mitochondria form *S. cerevisiae*, which is a robust and well-studied system for protein import assays. **Fig. 4C** shows the autoradiography experiment of isolated, intact, mitochondria incubated with 35S-labelled full length muscarinic $M_2$ receptor or $M_2$tail(368-466), made using a reticulocyte in vitro translation system. The data show that the full-length receptor was not imported into mitochondria. On the other hand, the $M_2$tail(368-466) displays a clear mitochondrial import already after 5 minutes and increasing over time, up to 30 minutes. Interestingly, this import appears to be independent upon the transporters of the outer membrane Tom5, Tom6, Tom7 and Tom70 (**Fig. S7D)**, whereas the import of an inner membrane protein, the ADP/ATP carrier (AAC), used here as a control, depends on Tom70. Upon extraction with carbonate most of $M_2$tail(368-466) was found in the pellet, and therefore presumably integrated in mitochondrial membranes. Furthermore *Blue native* analysis suggests that the mitochondrially imported $M_2$tail(368-466) is not a monomer but rather it is part of a larger complex of approximately 180 kDa (**Fig. S7D).** All together these data indicate that the import of the $M_2$tail(368-466) leads to its localization in one of the mitochondrial membranes, to form a complex with other native mitochondrial proteins.

To further discriminate if the construct is on the outer or inner mitochondrial membrane, we set up a fluorescence assay based upon GFP fluorescence complementation [15] . HEK293 cells were first transfected with two constructs: $M_2$tail(368-466)-GFP11, in which a short 16 amino acids peptide of GFP was fused at the C-terminus of $M_2$tail(368-466), and mito-GFP1-10, in which the COX8A was N-terminally fused to the other GFP fragment containing the remaining 10 beta strands (See **Materials and Methods**). **Figure S8A** shows the successful recombination of the two proteins within the mitochondrial matrix. This observation is supported by a set of controls that include a mitochondrial intermembrane space-targeted protein achieved using the targeting sequence of SMAC [16]:(**Figure S8B-D** show a set of positive recombination controls using respectively SMAC-GFP11+SMAC-GFP1-10, mito-GFP11+mito-GFP1-10 and $M_2$tail(368-466)-GFP11+ $M_2$tail(368-466)-GFP1-10, all displaying mitochondrial recombination comparable to what is observed in **Figure S8A** for $M_2$tail(368-466)-GFP11+mito-GFP1-10. As negative controls

we observed the lack of complementation of $M_2$tail-GFP11 with SMAC-GFP1-10 (**Figure S8E**), and the absence of any significant signal when mito1-10 was expressed alone (**Figure S8F**).

We then applied this approach to the full length $M_2$ receptor, by generating the $M_2$-GFP11 construct and by transfecting it in HEK293 cells. **Fig. 4D** shows a green signal from the recombined GFP visible in the mitochondria. All together these data suggest that the $M_2$ C-terminal fragment is localized in the inner mitochondrial membrane, with its own C-terminal domain facing the matrix.This interpretation is also finally confirmed by Immuno Electron Microscopy (IEM) imaging of thin sections from cells expressing the $M_2$stop228 C-terminally labeled using a myc-tag after immune-gold labelling (**Fig. 4E**). As shown in **Figs 4F-G**, gold particles were observed only in transfected cells (**Figure S7E**), and exclusively in mitochondria, and at this stage they appeared localized either to the mitochondrial matrix or to the lumen of the cristae (**Figs 4H-K)**.

**Cell stress upregulates IRES-mediated $M_2$tail expression and drives mitochondrial localization**

Having established that $M_2$tail localizes in the inner mitochondrial membrane we then moved to look at the effect of stress on the level of expression of the C-terminal fragment. Stressed cells are known to attenuate their translational activity via phosphorylation of the initiation factor eIF2α. However, certain mRNAs may be insensitive to eIF2α phosphorylation level if their translation is regulated by IRES, and under these conditions their expression is bound to increase rather than to attenuate [17, 18].

Therefore, we transfected COS-7 cells with the Sirius-$M_2$i3(417n)-EGFP reporter construct and stressed by starvation for three hours by replacing the culture medium with HBSS or phosphate saline buffer (PBS). Notably, PBS has a poorer nutrient content than the HBSS media, thus causing a more pronounced starvation. During this time interval, the green fluorescence was seen to gradually increase up to 1.4 times the basal level observed in HBSS-starved cells and 3.5 times for PBS-starved cells (**Fig. 5A**), confirming that the C-terminal fragment expression is upregulated in response to stress conditions such as cell starvation. The same experiment was reproduced in single cells, displaying an increase of the average EGFP/Sirius ratio (slope), and thus of IRES-mediated translation, upon HBSS starvation in HEK293 cells (**Fig. 5B)**. We further used the GFP fluorescence complementation assay to emphasize the importance of stress in the pattern of expression of the $M_2$ gene. HEK293 cells were co-transfected with $M_2$-GFP11 and Mito-GFP1-10. HEK293 cells grown in culture medium predominantly expressed the full length $M_2$ receptor protein (**Fig. 5C**).

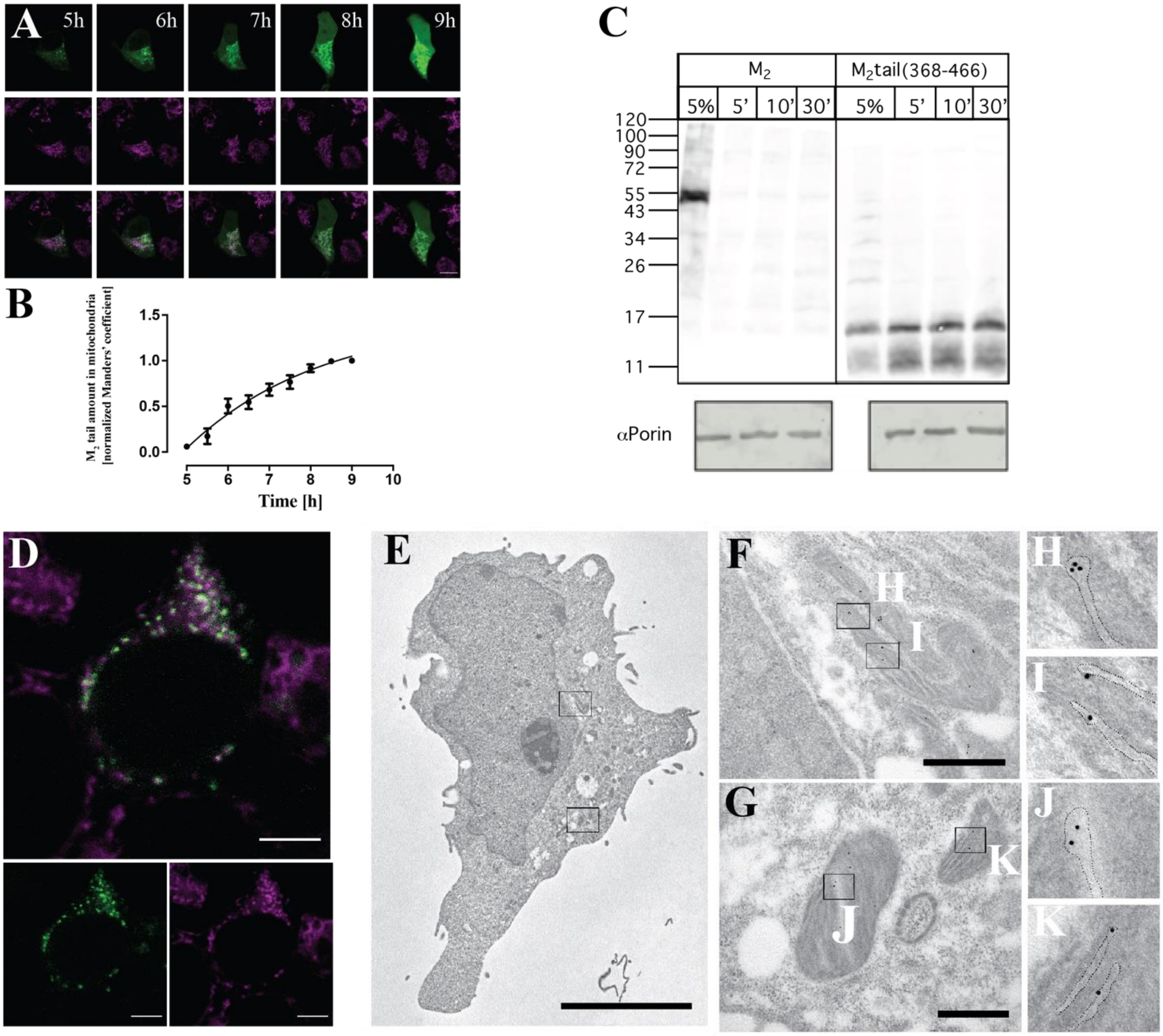


**Fig. 4. Mitochondrial import and organelle localization of $M_2$tail**

**A,** Time course of $M_2$tail(368-466)-EGFP import in the mitochondria of a HEK293 cell over a 4h interval, starting 5h after cell transfection, as indicated. Panels, from top to bottom show $M_2$tail(368-466)-EGFP (green), Mitotracker (magenta) and overlay displaying increasing colocalization over time. **B**, Quantification of the colocalization of the $M_2$tail(368-466)-EGFP in the mitochondria by calculating the Manders Coefficients (n=7 cells, from 3 independent transfections, error bars are s.e.m.). **C**, Import of protein $M_2$tail(368-466) in isolated yeast mitochondria: $^{35}$S-labelled and translated in-vitro full length muscarinic $M_2$ receptor or $M_2$tail(368-466) expressing 5 C-terminal Methionines were with isolated yeast mitochondria for 5, 10 and 30 minutes in the presence of the inner membrane potential The 5% lane represents a control with the pure protein, with no mitochondria. $\alpha$Porin is used as a loading control.. **D,** Detail of a HEK293 cell expressing the $M_2$-GFP11 construct together with mito-GFP1-10. The $M_2$-GFP11 was sequentially

transfected 24 h after the mito-GFP1-10  Magenta indicates mitotracker. White signal in the overlay indicates $M_2$tail(368-466) trafficking to the organelle. All confocal micrographs were obtained by sequential imaging using 488 nm excitation and detection in the 520-600 nm (EGFP), and at 633 nm and detected in the 650-700 nm range (Mitotracker) using HyD detectors in Photon Counting Mode. Scale bars are 10 µm throughout. **E,** Representative Immuno Electron Microscopy (IEM) micrograph of a COS-7 cell showing a large nucleus, several mitochondria and vacuoles nearby. Bar: 5 µm. **F-G**, High magnification of portion of the cell cytoplasm showing a few gold particles confined to the mitochondria. Bar: 500 nm. **H-K,** Gold particles appear associated with the mitochondrial cristae, highlighted by the dotted line. A total number of 236 gold particles in 40 mitochondria from 19 sections were detected. Controls reported in **Fig. S7E**.

The green fluorescence detected at the plasma membrane is explained with the chaperoning effect of $M_2$-GFP11 on Mito-GFP1-10 before its sorting to the mitochondria. In sharp contrast, when HEK293 cells were first transfected with Mito-GFP1-10 and, 24h later, transfected with $M_2$-GFP11 followed by a 6 h starvation in HBSS, the pattern of expression changed drastically: IRES mediated translation of the $M_2$tail(368-466) fragment led to a predominantly mitochondrial green fluorescence signal of the complemented GFP (**Fig. 5D**). These results are in line with our observation that the IRES mediated C-terminal fragment localization to the mitochondria in cells expressing the construct $M_2$-mRuby2-STOP-$M_2$-i3-tail-EGFP was enhanced upon cell starvation, and drastically reduced upon mutation of the M368 (**Figure S9** and **Supplementary Materials**).

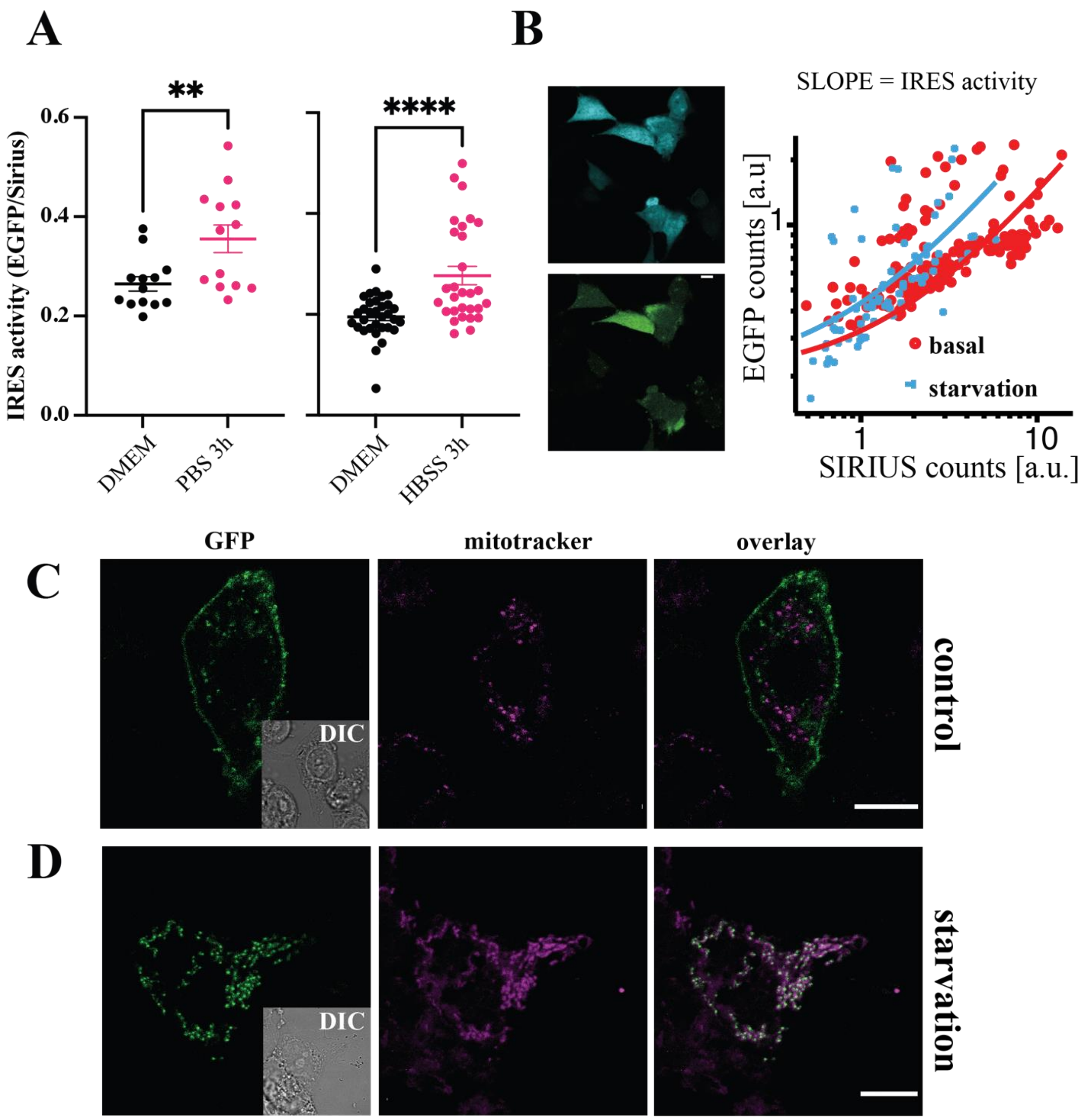


**Fig. 5 Effect of integrated stress response on IRES activity and $M_2$tail(368-466) localization**

**A**, 96-well plate reader measurement of the increase of EGFP fluorescence relative to Sirius in COS-7 cells transfected with the bicistronic Sirius-$M_2$i3(417n)-EGFP plasmid and starved for three hours respectively in PBS (left panel) (n= 3 transfections, 16 wells) and HBSS (right panel) (n=1 transfection, 16 wells). Significance values reported in the graphs were determined by a one-tailed Student t-test, p-values: **** p<0.0001; ** 0.001<p<0.01. **B,** Single cell measurement of EGFP vs SIRIUS expression in HEK293 cells, in basal conditions and upon starvation. Lines are linear fits (note log-log scale). **C**, HEK293 cells were sequentially transfected with pMito-GFP1-10 (24h) and $M_2$-GFP11 (8h, of which 6h under starvation

conditions) and then grown in full culture medium or **D,** starved for 6 h after transfection of $M_2$-GFP11.Shown are EGFP fluorescence (left, with DIC inset), Mitotracker (center) and overlay (right). Scale bars are 10 µm.

### Functional role of the C-terminal fragment in the mitochondria

The next step of our investigation concerned the obvious question about the functional effects that the observed localization of the $M_2$ C-terminal fragment might have on the mitochondrial activity, and -more generally- on the energy metabolism of the cell. To investigate this aspect, we measured the extent of oxygen consumption in COS-7 cells expressing $M_2$-EGFP, $M_2$tail(368-466)-EGFP or the sole EGFP as a control. Initially, we normalized the expression of the three proteins to the amount of EGFP expressed in the cells, then the metabolic functions were assayed on a *Seahorse* instrument using cells transfected with normalized amounts of plasmids (**Fig. 6A**). Our data did not show a significant difference in total oxygen consumption capacity between cells transfected with cytosolic EGFP and the untransfected control. However, a significant decrease of oxygen consumption was found in cells transfected with $M_2$-EGFP and even more so with $M_2$tail(368-466)-EGFP (**Fig. 6A**). Notably, the basal oxygen consumption rate (OCR) in starvation-induced stress conditions was profoundly decreased in $M_2$tail(368-466) cells. This occurred to a smaller extent also in $M_2$ cells compared to EGFP-transfected and empty control cells (see also the time-course in **Fig. S10A)**. These results are in line with what was observed using a Clarke-type oxygen electrode (**Supplementary Materials and Fig S10B-C**).

Notably, there was no difference in the number of apoptotic cells among the different cell groups as shown by the caspase-3 activation assays of apoptosis [19](**Fig. S10D**) and morphological investigation of the mitochondrial network (**Fig. S10E**). To test whether the $M_2$tail effects on mitochondrial oxygen consumption could alter the production of Reactive Oxygen Species (ROS), the measurements were combined in a single cell assay. Remarkably, ROS production (assayed using a fluorescence readout kit, where the fluorochrome becomes activated in contact with oxidized species) was clearly reduced within and in proximity of mitochondria of cells expressing a high level of the $M_2$tail(368-466) (**Fig. 6Binset**). By regrouping individual mitochondria in classes according to the level of the ROS marker expression, an inverse correlation between the amount of receptor fragment (Green Fluorescence) and the production of ROS (Red Fluorescence) was revealed (F**ig. 6B)**.

Cell growth was also found to be affected depending on which of the constructs was transfected. Notably, transfection of HEK293 cells with full length $M_2$ receptor reduced significantly cell growth compared to the control, whereas cells transfected with the mutant $M_2$M368A, that do not express the $M_2$ C-terminal fragment (**Fig. 6C**), displayed a significantly higher growth. All cells were

incubated with saturating concentration of the $M_2$ antagonist atropine throughout, in order to exclude effects on cell growth and proliferation due to the canonical receptor signaling.

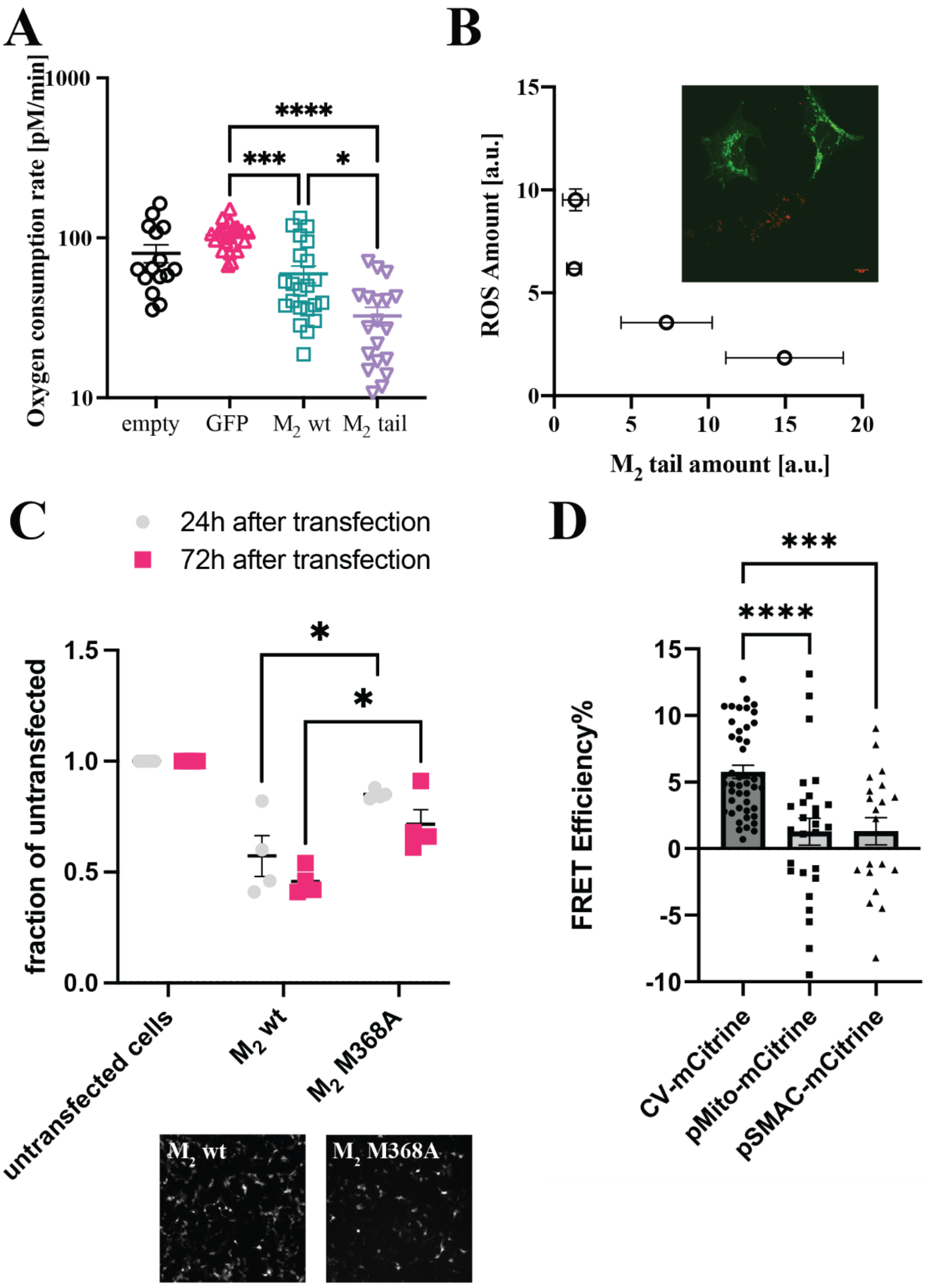

**Fig. 6. Effects of $M_2$tail expression, or absence thereof, on mitochondrial function and cell metabolism in transfected HEK293 cells.**

**A**, Basal oxygen consumption upon HEK293 cell stress -following starvation- measured by a Seahorse assay at a constant temperature of 37 °C. Significance values reported in the graphs were determined by a one-tailed Student t-test, p-values: **** $p<0.0001$; *** $0.0001<p<0.001$;* $0.01<p<0.05$. **B**, Localization of $M_2$tail(368-466)-EGFP in HEK293 (green) cells stained with a marker for reactive oxygen species (Cell Rox Deep Red). Relationship between green ($M_2$tail abundance) vs red (amount of ROS produced) fluorescence intensities measured from regions of the mitochondrial network. The data were binned in four groups as a function of the amplitude of Cell Rox Deep Red signal (0-2.5, 2.5-5. 5-7.5 and 7.5-10 intensity counts). **C**, Cell viability assay performed on HEK293 cells transfected with $M_2$ and $M_2$M368A, compared to an untransfected control. Cells were counted 24h and 72h after transfection in T-25 flasks, exposed throughout to 100 nM atropine. The experiments were performed in two biological replicates, each with two technical replicates. Transfection efficiency (to the right of the chart) was monitored thanks to the mRuby2 fluorescent reporter within a bicistronic cassette within each vector (see **Materials and Methods**). Significance values reported in the graphs were determined by a one-tailed Student t-test, p-values: ** $0.001<p<0.01$; * $0.01<p<0.05$. **D**, FRET efficiency plot of the interaction between $M_2$tail(368-466)-mTurquoise2 and $F_0F_1$ATP Synthetase (CV-mCitrine), COX8a(pMito)-mCitrine and SMAC-mCitrine, as determined by acceptor photobleaching measurement in a confocal microscope. Each dot represents a ROI within a cell. At least 3 cells from two separate transfections were imaged for each pair.

These functional data are further supported by Fluorescence Resonance Energy Transfer (FRET) data aimed at investigating the molecular interactions of the mTurquoise2 labeled $M_2$tail(368-466) with mitochondrial proteins. Based on the oxygen consumption data (**Fig. 6A** and **Fig. S10A**) we chose as primary target the $F_0F_1$ ATP Synthetase complex, also indicated as complex V (CV), and we labeled the γ subunit with mCitrine, a fluorescent acceptor. The γ subunit is located towards the mitochondrial matrix. We further monitored interactions with an intermembrane space protein (SMAC) and a protein localized to the mitochondrial matrix (COX8a), both fused to the mCitrine fluorescent acceptor. As clearly illustrated in **Fig. 6D,** FRET efficiency, an indicator of molecular interaction, is significantly larger for $M_2$tail(368-466)-CV pair than both the intermembrane space SMAC and the matrix COX8a. The latter result is particularly relevant, as it indicates a specific interaction of $M_2$tail(368-466) with CV.

## Relevance of C-terminal expression and mitochondrial translocation in a physiological, endogenous setting

The results provided in the previous sections convincingly demonstrate the cap-independent translation of the $M_2$ C-terminal fragment, its upregulation and mitochondrial localization upon stress response, and the subsequent effect in regulating oxidative phosphorylation by a direct interaction with complex CV of the respiratory chain. To test the physiological relevance of these findings we first prepared protein extracts from heart, brain and spleen tissues from a line of knock-in mice (see **Materials and Methods**), designed to have expression of a Chrm2/tdtomato fusion protein driven by the endogenous $M_2$ muscarinic acetylcholine receptor promoter/enhancer sequences. The heart and brain are known to express sizable levels of the $M_2$ receptor, whereas the spleen, where no expression of the $M_2$ receptor is reported in the proteomic databases, was used as a negative control.

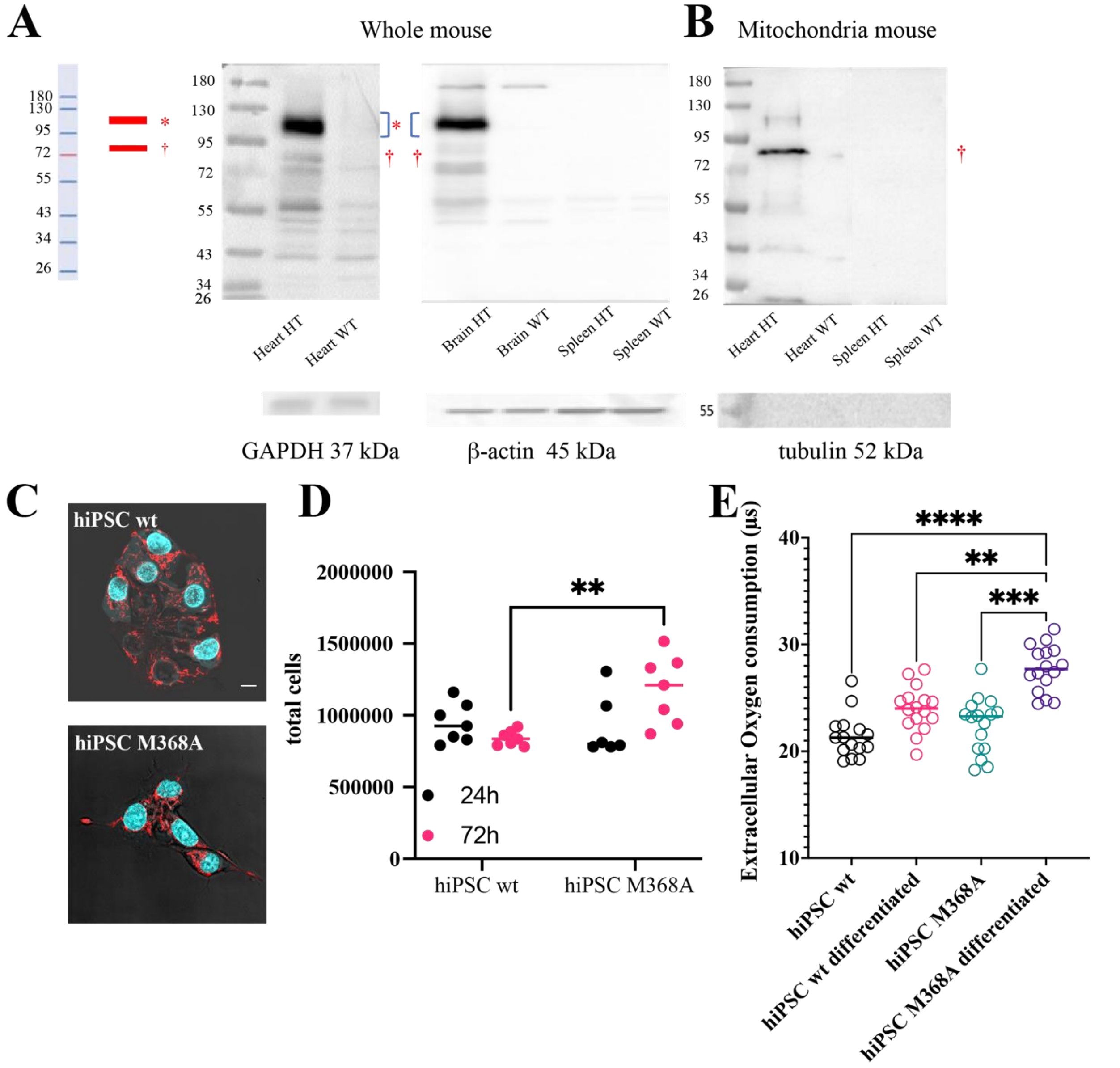

**Fig. 7. IRES-mediated $M_2$tail(368-466) expression occurs in endogenous, physiological settings and its suppression affects cell metabolism**

**A**, Western blot from tissue preparations originating from the indicated organs of $M_2$-tdtomato knock-in mice (HT) and wild-type animals (WT). Immunodetection was performed using an anti-tdtomato primary antibody. Loading control normalization was performed using GAPDH in the heart, and $\beta$-actin in the brain and spleen. **B**, Western blot (8% TRIS-Glycine PAA gel) of mitochondria purified from tissue preparations originating from the indicated organs of $M_2$-tdtomato knock-in mice (HT) and wild-type animals (WT), detected using an anti-tdtomato antibody. Mitochondrial preparation purity was assessed by blotting tubulin. **C,** Confocal micrographs of wt hiPSCs (left) and Crispr/Cas9 clone harboring the M368A mutation within the $M_2$ gene (right), stained with DAPI (cyan) and Mitotracker (red). **D**, Cell proliferation assay performed for wt hiPSCs and M368A hiPSCs grown in T25 flasks, exposed to Atropine. Cells were counted 24h and 72h after seeding. Significance values reported in the graphs were determined by a one-tailed Student t-test, p-values: ** $0.001<p<0.01$. **E**, Extracellular oxygen consumption assay, performed on wt and M368A hiPSCs, prior to and after 7 days undirected differentiation under Atropine exposure. The lifetime of a commercial dye increases as oxygen in the medium is progressively consumed by the cells. The data displayed wascollected after 60 minutes incubation. Significance values reported in the graphs were determined by a one-tailed Student t-test, p-values: *** $0.0001<p<0.001$; ** $0.001<p<0.01$.

The use of a transgene tag was necessary, since antibody against the $M_2$ receptor are notoriously ineffective when targeted at the C-terminal portion of the receptor. Instead, the tdtomato fluorescent protein can be targeted by a specific polyclonal antibody, as shown by the Western blot displayed in **Fig. 7A**. As expected, the full-length receptor-tdtomato fusion was observed at ~130 kDa (~75 kDa contributed by the receptor and ~55 kDa contributed by tdtomato). Notably we observed again (both in the heart and brain, but not in the spleen) a specific band doublet, this time at ~75 kDa, which corresponds to the sum of the molecular weights of the tail fragment added to the molecular weight of tdtomato. When mitochondria were purified from the heart and spleen of the Chrm2/tdtomato mice **Fig. 7B** the band at ~75 kDa was substantially enriched, matching the expected molecular weight of the $M_2$tail. These data strongly suggest that the $M_2$tail is expressed endogenously, and it is sorted to the mitochondria.

We finally posed the question of what would happen abolishing the translocation of the $M_2$ C-terminal fragment to the mitochondria, in a physiological and entirely label-free setting. Notably, human induced pluripotent stem cells (hiPSC) combined with genome editing approach are state-of-the art tools to investigate the effect of gene mutations in physiological and label-free setting. Therefore, we incorporated by Crispr/Cas9 the homozygous M368A mutation in hiPSCs

(**Materials and Methods**). Based on our data of (**Fig. 3C**), this mutation should be sufficient to significantly impair the $M_2$tail production and mitochondrial translocation. Wild type (wt) and M368A hiPSCs were cultured in pluripotency maintaining medium, and subsequently undirectedly differentiated upon exposure for seven days to DMEM-F12 medium, as described in the **Materials and Methods**. **Fig. 7C** shows confocal micrographs for wild type and M368A hiPSCs cultured in E8 medium respectively, with the M368A hiPSCs displaying a mildly more spread-out phenotype. We shall note here that the $M_2$ receptor has been found to be expressed in PSCs lines, and it has been shown to play a role in stem cell differentiation [20, 21]. In this context, however, we were not interested in the canonical signaling of the receptor, and all experiments were performed with a saturating concentration of atropine, to block any possible effect of the canonical signaling of the $M_2$ receptor. We then evaluated cell proliferation, observing a significant increase in the proliferation of the M368A clone 72h after seeding **Fig. 7D**. The observation that cells harboring the mutation that suppresses $M_2$tail display an increased proliferation suggests that the same effect is at play here, as observed in the overexpression system (**Fig. 6C**). These results are supported by extracellular oxygen consumption assays performed, for both hiPSCs and differentiated hiPSCs (**Fig. 7E)**: the M368A cells indeed display an accelerated oxygen consumption rate, and this effect increases as the cells progressively differentiate.
For comparison, the assay was also carried on HEK293 overexpressing the wild type $M_2$ receptor and the mutant $M_2$M368A (**Fig. S10F)**. We shall further note here that signaling properties of $M_2$ M368A with respect to the wild type $M_2$ receptor are comparable, as displayed by pERK immunoblotting as well as FRET $G_i$ activation assays performed in cells overexpressing either the wild type $M_2$ or the $M_2$ M368A (**Fig. S10 G,H**).

## Discussion

Over the last 10 years, growing evidence has accumulated indicating that GPCRs can be functional and signal also at cellular locations other than the plasma membrane. These locations can be associated to their canonical trafficking pathways (such as sorting vesicles and endosomes) [22], but also to non-canonical locations, such as the nucleus, the nuclear membrane, the Golgi apparatus and the mitochondria (reviewed in Jong et al. BPJ 2018 [23]). Some of these locations are surprising, since many ligands are non-membrane permeable, and it is still debated how they can reach their target receptors at such intracellular locations.

Interestingly, the specific localization of these receptors appears to go hand in hand with a specific function. In these cases, activation/inhibition of mitochondrially localized GPCRs have been

shown to specifically affect the organelle function, ranging from oxygen consumption rates to $Ca^{2+}$ handling and even to apoptosis. Notably Benárd et al. [24] showed that the $CB_1R$ on the outer mitochondrial membranes affects neuronal energy metabolism; Suofu et al. [25] demonstrated that activation of the $MT_1R$ by a mitochondrial melatonin pool can affect cytochrome C release. This is particularly interesting, since cytosolic cAMP does not appear to be able to activate mitochondrial PKA, and hence a mitochondrially localized GPCR signalosome appears to be required to enable such downstream signaling cascade [26].

However, other non-canonical mechanisms are also possible for the regulation of mitochondrial function. It is well known that GPCRs are subject to interindividual variability, arising, for example, from mRNA alternative splicing (i.e. the process which allows the recombination of exons) which leads to different isoforms which may present distinct signaling and localization properties[27]. Receptor variants with different functional and localization properties may also occur due to other processes. For example, our comprehension of eukaryotic translational regulation is facing a rapid expansion, as exemplified by the observation that small open reading frames (smORFs), also within long non-coding RNAs (lncRNAs), are an important class of genes, oftentimes with mitochondrial localization [28].

In this study, for the first time, we provide evidence that an IRES sequence drives expression of a membrane receptor fragment and acts as a regulator of its expression levels and function. IRES sequences are distinct regions of the mRNAs that are capable of recruiting ribosomes in the proximity of an internal initiation codon and translate transcripts in a cap-independent manner[8, 9, 28-30]. While in eukaryotes the most common mechanism of translation initiation is the cap-dependent process, which requires the interaction of several key proteins at the 5'-end (5′ cap) of mRNA molecules, recent years have seen growing evidence that IRES sequences, which are common features of virus replication [8], can mediate cap independent translation of eukaryotic mRNA(s) [9].

The IRES sequence is located within the third intracellular loop of the $M_2$ muscarinic receptor, where it plays a crucial role in regulating the expression of its carboxy-terminal domain, which we identified as $M_2$tail(368-466).

We assessed the IRES-driven expression of the C-terminal fragment through a broad set of constructs, meant to exclude alternative synthesis mechanisms such as stop-codon read-through, and a diversified palette of techniques. Radioligand binding data (**Table 1**) pointed to the necessity

of the C-terminal fragment synthesis, to explain the observed downstream signaling in mutants carrying a stop codon in the initial portion of the i3loop of the $M_2$ receptor, upstream of M368. Fluorescence microscopy allowed us visualizing the production of the EGFP-tagged C-terminal fragment in these receptor mutants (**Fig. 1**).

Mutagenesis of key residues within the i3 loop of the $M_2$ receptor allowed us to pinpoint the 3$^{rd}$ in-frame methionine as the most likely candidate for the beginning of the cap-independent translation (**Fig. 2**). This observation appears to be supported by the observation of a peak in ribosome coverage around the M368 seen in ribosome profiling databases (as reviewed in Ingolia et al. [31]), and particularly prominent in those from left ventricle cardiomyocytes, where $M_2$ receptor expression is known to be abundant [32].

To our surprise, in contrast to the plasma membrane localization of the wild type, full length $M_2$ receptor, the C-terminal fragment was localized mostly in mitochondria in HEK293 cells. This localization occurred markedly when overexpressing the C-terminal fragment $M_2$tail(368-466), but could be also clearly detected when the C-terminal fragment was expressed under the control of the IRES sequence of the full-length receptor (**Fig. 3**). These results were further validated in a rat cardiomyoblast cell line such as H9c2 (**Fig. S7C**).

We devised several strategies to detect the localization of the IRES-driven fragment in the mitochondria by avoiding the cell-wide background signal from the full-length receptor. **Fig. 3D,E** show two prominent examples, namely relying on spectrally unmixing the two components, or by sending out of frame the EGFP at the C-terminus of the full-length receptor. By studying mitochondria purified from HEK293 cells we could confirm that the mutation of the M368, which we identified as the starting point of the IRES-mediated translation, is sufficient to suppress any mitochondrial targeting of the $M_2$ C-terminal fragment, and most likely, its synthesis altogether (**Fig. 3C**).

We then addressed the mechanism of mitochondrial import of the C-terminal fragment and its localization within the organelle (**Fig.4**). Our data, originating both from live cell import kinetics as well as from in-vitro import into isolated mitochondria (**Fig.4A,B**), support the notion that the fragment becomes rapidly imported into mitochondria following translation in the cytosol. Once inside the mitochondria, it undergoes processing leading to the appearance of a lower molecular weight band, observed both in Western blots from whole cell and mice tissue protein extracts, as

well as from purified mitochondria and in the yeast in vitro assays (**Fig. 4C**). The fragment is found predominantly in a carbonate-insoluble fraction, i.e. a membrane, and it appears to form a high molecular weight complex of ~180 kDa with other mitochondrial proteins (**Fig. S7D)**.
Fluorescence complementation assays (**Fig. 4D**) and electron microscopy (**Fig. 4E-H**) suggest that the $M_2$ C-terminal fragment predominantly resides in the inner mitochondrial membrane, with its own C-terminal domain exposed towards the matrix. Future work should clarify the precise import pathway.

Under integrated stress response, namely aminoacid starvation, IRES-mediated production of the C-terminal fragment was increased (**Fig. 5A-B**), with a marked increase in mitochondrial localization (**Fig. 5C)**. Most importantly, our data show that mitochondrial oxygen consumption was reduced by the $M_2$tail(368-466) (**Fig. 6A,B**), and cell proliferation was affected (**Fig. 6C**). On these bases, the $M_2$tail might be needed in mitochondria as a means to reduce mitochondrial basal oxygen consumption and ATP-linked respiration. An important consequence of reduced respiration is the lower level of ROS produced in mitochondria, as we observed in agreement with this hypothesis **Fig. 6B**.

This observation was confirmed in physiological conditions by Western blots in a knock-in mice carrying a tag at the C-terminus of the endogenous $M_2$ receptor. These could show mitochondrial enrichment of a band corresponding to the molecular weight of the C-terminal domain (**Fig. 7A).** Moreover**,** the physiological relevance of our observations was tested in an entirely label-free assay, by Crispr/Cas9 editing of pluripotent stem cells in order to suppress the synthesis of the $M_2$ C-terminal fragment. By mutating the IRES starting methionine M368, we observed an increase of cell proliferation of this clone compared to its wild type counterpart, together with an increase of oxygen consumption rates (**Fig. 7B-D**). Overall this corroborates our hypothesis that the $M_2$ C-terminal fragment reduces oxygen consumption upon reaching the mitochondria. The effect was further amplified when the cells were undirectedly differentiated, and it was consistent with the shift of the cell phenotype towards oxidative phosphorylation [33].

The mechanism by which the $M_2$tail(368-466) may reduce oxygen consumption in mitochondria and its meaning needs to be further explored. Our FRET data (**Fig. 6D**) indicate clearly that the $M_2$ C-terminal fragment has a specific interaction with the $F_0F_1$ ATP-synthase complex, supporting the concept that it inhibits the activity of this enzyme, thus leading to the observed reduction in oxygen consumption rates. Future work should clarify the precise import pathway and the

inhibitory effect of $M_2$tail(368-466) on $F_0F_1$ ATP-synthase. Nonetheless, since ROS production is implicated in the pathogenesis of several diseases [33], we would propose the hypothesis that increased localization of the $M_2$ C-terminal fragment in mitochondria, as it occurs under integrated stress response, might have protective effects (**Fig. 8**).

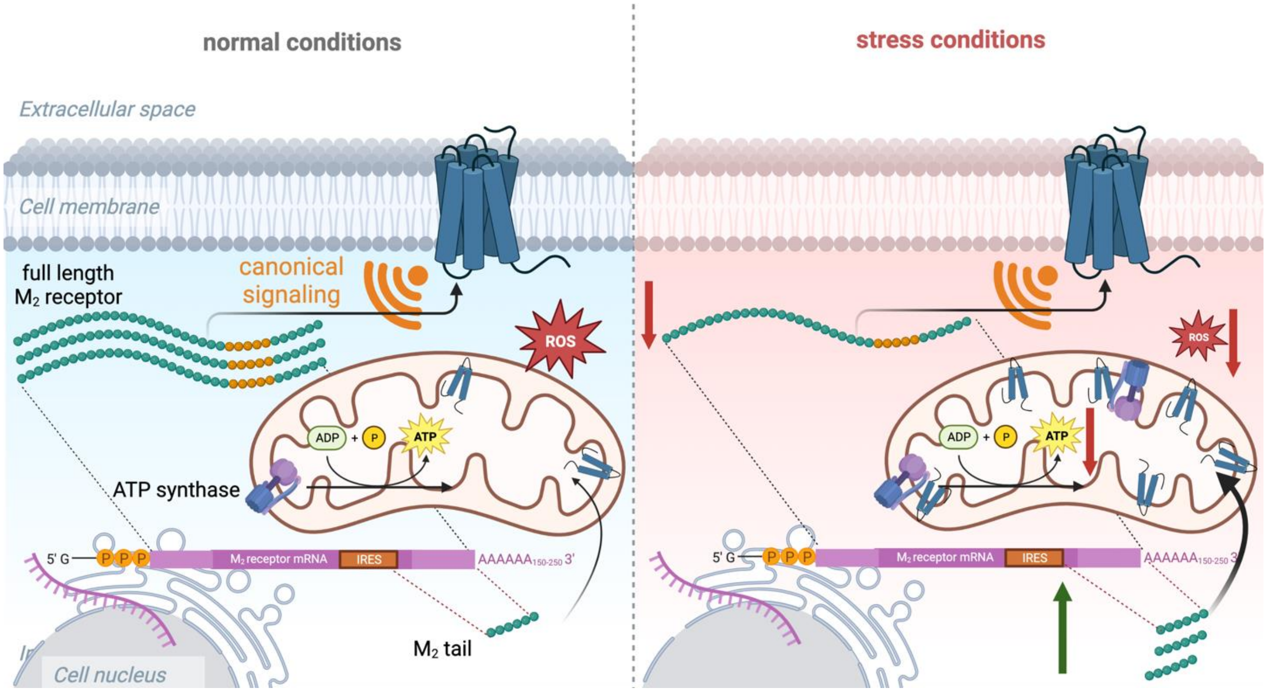


**Fig. 8. Schematic representation of the wild type $M_2$ receptor gene products, their destination in the cell and their function.**

The $M_2$ receptor gene gives rise to one transcript but two different translation outputs: when translation starts from the canonical cap site the wild type M2 receptor is generated (full length $M_2$ receptor). When translation is driven by the internal ribosome entry site (IRES) the C-terminal fragment is generated ($M_2$ tail). The two proteins are then sorted in two different cell compartments where they play completely different roles: the $M_2$ receptor signals canonically at the plasma membrane as a G protein-coupled receptor. The $M_2$tail is instead sorted to the mitochondria.

In normal conditions the activity of the IRES is low, and only a small amount of $M_2$tail is produced in the cell (left). However, upon cell stress conditions (right), $M_2$tail IRES-mediated translation is upregulated and $M_2$tail in the mitochondria interacts with the ATP synthase and reduces oxidative phosphorylation and ROS formation. Created with BioRender.

In broader terms, protection of mitochondria by unconventional import mechanisms to allow localization within the organelle of specific proteins has been previously observed for cells that undergo stress conditions[34] . The mechanism of IRES-dependent modulation of translation and consequent mitochondrial targeting of the translation product of a GPCR may provide another

means, completely unrelated to the canonical signaling function of this class of membrane receptors, of a generic cellular response to overcome different types of stress by modulating the mitochondrial proteome and adjusting the mitochondrial bioenergetic state.

## Materials and Methods

### Generation of mutant muscarinic $M_2$ receptors for radioligand binding studies, Western blotting, immunohistochemistry and fluorescence microscopy

Multiple receptor mutants were constructed starting from the human $M_2$ muscarinic receptor expressed in the pcD vector [35]. The constructs are sketched in **Fig. S3** and their cloning is described in detail in the **Supplementary Methods**.

### Reverse transcription

Reverse transcription was performed using a 24-nucleotide antisense oligo (TTACCTTGTAGCGCCTATGTTCTT) directed against the last 24 nucleotides at the 3' end of the $M_2$ open reading frame. cDNA amplification was carried out with the same antisense oligo used for reverse transcription, together with a 24 nucleotides sense oligo (ATGAATAACTCAACAAACTCCTCT) directed against the first 24 nucleotides at the 5' end of the $M_2$ open reading frame.

### Cell culture and transfection

COS-7 (African green monkey kidney fibroblast-like SV40 transformed cell line) and CHO (Chinese Hamster Ovary) cells (from American Type Culture Collection) were grown at 5% $CO_2$ and 37 °C, in high glucose Dulbecco's modified Eagle's medium (DMEM) supplemented with 10% fetal bovine serum (FBS), 2 mM L-glutamine, 50 IU/ml penicillin, and 50 mg/ml streptomycin, and non-essential amino acids. HeLa cells (from American Type Culture Collection) were cultured in the same medium, but with the double amount of L-glutamine. SHSY-5Y cells were cultured in Roswell Park Memorial Institute (RPMI) medium supplemented with 10% FBS, 2 mM L-glutamine, 50 IU/ml penicillin, and 50 mg/ml streptomycin. Media and reagents used for cell cultures were from Euroclone, PAN Biotech, Biochrome and Sigma-Aldrich. Cell transfection was performed using FuGene® transfection reagents (Promega) as recommended by the manufacturer. Eighty percent confluent cells were washed twice with free-serum medium and the mix of FuGene and plasmid DNA was added. In every experimental condition 6-8 µg of plasmid DNA per Petri (10 $cm^2$) were used. Cells were grown for 48 h post-transfection before use. In co-transfection experiments, half amount of each plasmid DNA was used.

HEK293 (Human Embryonic Kidney, ECACC 96121229 from Sigma-Aldrich Chemie GmbH) were cultured in DMEM (Dulbecco's modified Eagle's medium PAN biotech, Aidenbach, Germany), supplemented with 4.5 g/L Glucose, 2 mM L-Glutamine, 10% FCS, 100 units/mL penicillin and 0.1 mg/mL streptomycin and maintained at 37 °C and 5% $CO_2$. Cells cultured in 15-cm dishes were split at a 1:36 ratio into 6-well plates containing poly-D-lysine (PDL)-coated 24 mm glass coverslips. Cells were transfected using Effectene transfection reagent (QIAGEN) according to the manufacturer's instructions. Cells seeded on PDL-coated coverslips in 6-well plates were transfected 16 hours after seeding with 0.6 µg plasmid/well.

**Fluorescence microscopy**

Fluorescence microscopy experiments were performed either on a Leica SP5 (or SP8) Confocal Microscopes, using HyD photon counting detectors and an Argon/Ion laser (or white light laser) source for 488 nm/633 nm excitation and a solid-state diode laser (or white light laser) for 561 nm excitation. A solid-state diode laser was further used for 405 nm excitation. A 40x 1.3 NA objective was used, and the electronic zoom was set to achieve a pixel size of 50 nm. For visualization, mitochondria of transfected cells were labelled for 30 minutes at 37 C with 50nM MitoTracker® Deep Red FM (Invitrogen™, Thermo Scientific).

**Ligand binding studies**

Cells were lysed 48 h post-transfection with ice-cold 2 mM EDTA, 1 nM Na-HEPES (pH 7.4), scraped with a rubber policeman in the presence of ~4 ml rinsing medium, and homogenized with a Polytron homogenizer (ULTRA-TURRAX) in binding assay buffer (50 mM Tris-HCl, pH 7.4, 155 mM NaCl, 0.01 mg/ml bovine serum albumin). Radioligand bindings were carried out at 37 °C for 3 h in a final volume of 1 ml. Non-specific binding was determined in presence of 1 µM atropine. For competition binding assays, increasing concentrations of carbachol (from 0.01 µM up to 10 µM) were used in presence of 200 pM [$^3$H]N-Methylscopolamine ([$^3$H]NMS; 78.9 Ci/mmol; PerkinElmer).

The bound ligand was separated from the unbound ligand using glass-fiber filters (Whatmann, GF/B) with a Brandel Cell Harvester, and filters were counted with a scintillation β-counter. $K_D$ values of [$^3$H]NMS were calculated in direct saturation experiments, whereas carbachol Inibitory Concentration $(IC)_{50}$ values were calculated in competition curves, against 400 pM [$^3$H]NMS, fitted to one binding models using the iterative, non-linear least-squares regression analysis of OriginPro 7.5 software (OriginLab Corporation).

**Phosphatidylinositol Breakdown Assay**

Transfected COS-7 cells were incubated with myo-[$^3$H]inositol (23 Ci/mmol; PerkinElmer) for 48 h. Immediately before the experiment the cells were washed twice with phosphate-buffered saline and incubated for 15 min in Eagle's medium containing 10 mM LiCl and 20 mM HEPES. The medium was then replaced by 0.25 ml of the same medium containing the experimental agents. After a 1-hour incubation at 25 °C the reaction was arrested by the addition of 0.75 ml of 7.5% (w/v) ice-cold trichloroacetic acid followed by a 30-min incubation on ice. The trichloroacetic acid was extracted with water-saturated diethyl ether (3 × 4 ml), and levels of $IP_1$ determined by anion exchange chromatography.

**Adenyl Cyclase Assay**

COS-7 cells were transfected with plasmid(s) containing the receptor of interest plus adenyl cyclase V. 24 h after transfection the cells were detached with trypsin and re-cultured in 24-well plates. Following an additional period of 24 h culture, they were assayed for adenyl cyclase activity. The assay was performed in triplicate**.** In brief, cells were incubated in 24-well plates for 2 h with 0.25 ml/well of fresh growth medium containing 5 μCi/ml [$^3$H]adenine (PerkinElmer). The medium was then replaced with 0.5 ml/well of DMEM containing 20 mM HEPES, pH 7.4, 0.1 mg of bovine serum albumin, and the phosphodiesterase inhibitors 1-methyl-3-isobutylxanthine (0.5 mM) and Ro-20–1724 (0.5 mM). AC activity was stimulated by the addition of 1 μM forskolin in the presence or absence of carbachol. After 10 min of incubation at +30 °C the medium was removed, and the reaction terminated by addition of perchloric acid containing 0.1 mM unlabeled cAMP followed by neutralization with KOH. The amount of [$^3$H]cAMP formed under these conditions was determined by a two-step column separation procedure[3].

**Immunoelectron Microscopy (IEM)**

Samples were fixed for 3 hours at 4 °C with a mixture of 4% PFA and 0.05% GA in 0.1M PB, pH 7.4. After rinsing in the same buffer, samples were dehydrated in a graded series of ethanol and embedded in medium grade LR White resin, following addition of the LR White accelerator (London Resin Company, Berkshire, England). The resin was then polymerized for 20 min at room temperature in tightly capped gelatine capsules. Ultrathin sections were obtained using a Reichert Ultracut ultramicrotome equipped with a diamond knife and collected on nickel grids. For immunogold labelling, 60-80 nm thick sections were processed following the incubation protocol originally developed by Aurion (Wageningen, the

Netherlands). Unspecific binding was prevented by treating sections for 20 min with 0.05 M glycine in PBS, at pH 7.4. A subsequent block step was made by washing sections for 30 min in 5% BSA, 5% normal goat serum and 0.1% cold water fish skin gelatine. Sections were soaked overnight 4 °C in incubation buffer (PBS and 0.1% BSA-cTM, pH 7.4) (3x5 min) in a moist chamber containing the Myc-Tag (9B11) mouse antibody (Cell Signaling Technology) diluted 1:50 with incubation buffer. Sections were then washed in incubation buffer (6x5 min) and incubated for 1 hour with a secondary goat anti-mouse antibody conjugated to 10 nm gold particles (EM Grade 10nm Goat anti-Mouse IgG Conjugate EM.GAM10/1, British BioCell International, UK), diluted 1:10 in incubation buffer. After rinsing in incubation buffer (6x5 min), grids were washed in PBS (6x5 min). Sections were subsequently stained with uranyl acetate and observed with a Jeol JEM EXII Transmission Electron Microscope at 100kV. Micrographs were acquired by the Olympus SIS VELETA CCD camera equipped the iTEM software. Control sections were obtained by omitting the primary antibody from the incubation mixture.

**Isolation of Yeast Mitochondria**

Mitochondria were isolated as in Kritsiligkou et al.[34],  , 2017. In short, D273-10B yeast were grown to OD600 1 in yeast peptone lactate. Cells were pelleted by centrifugation (3,000g) and washed in ddH2O and the cell pellet weight was calculated. The yeast cells were then washed in 100mM Tris-SO4 pH 9.4, 10mM DTT for 30 minutes in the presence of constant agitation (120rpm). The cells were washed in sorbitol buffer (1.2M Sorbitol, 10mM KPi pH 7.4) followed by resuspension in sorbitol buffer (5ml/g of cells) supplemented with zymolyase 20T (3.5mg/g of cells) and incubation for 45 minutes under constant agitation (120rpm) to create spheroplasts. The spheroplasts created in this process were washed in sorbitol buffer and resuspended in breaking buffer pH6.0(BB pH6.0)  (600mM Sorbitol buffer, 20mM MES-KOH pH 6.0, 1mM PMSF). The spheroplasts were the broken in BB pH6.0 using a glass type B pestle homogeniser for 15 strokes on ice. The cell debris was removed via differential centrifugation; first low speed centrifugation (1,480g  4ºC) then high speed (12,000g 4 ºC). Crude mitochondria were then purified further via gradient centrifugation on a 14.5-20% Nycodenz® gradient and spun at 202,496g at 4 ºC. The pure mitochondria were collected with a 19-gauge needle, washed in BB pH 7.4 (600mM sorbitol buffer, 20mM HEPES-KOH pH7.4) and protein concentration estimated. Finally, the mitochondria are frozen at -80ºC at a concentration of 20mg/ml in BB pH 7.4 supplemented

with 10mg/ml fatty-acid free BSA.

**Import of** $M_2$ **receptor and** $M_2$**tail(368-466) in isolated yeast mitochondria**

The $M_2$ receptor and $M_2$tail(368-466) were cloned into the pSP64 in vitro translation vector and translated with a 35S radiolabelled methionine label using the TNT® coupled reticulocyte lysate system (Promega) following manufacturers guidelines. Each import reaction comprised of 50µg of isolated yeast mitochondria resuspended in 100µl import buffer 1X (600mM Sorbitol, 2mM KH2PO4, 50mM KCl, 50mM HEPES-KOH pH 7.4, 10mM MgCl2, 2.5mM Na2EDTA pH 7, 5mM L-methionine, 1mg/ml fatty acid free BSA) supplemented with 2mM ATP and 2.5mM NADH. 5µl of TNT translated protein was added to each import reaction and the samples were incubated at 30ºC for 5,10 or 30 minutes. Import was ceased by the transfer of samples on ice. Where indicated, samples were treated with 0.1mg/ml Trypsin in breaking buffer (600mM Sorbitol, 20mM HEPES-KOH pH 7.4) for 30 minutes on ice followed by protease inactivation with the addition of soybean trypsin inhibitor (SBTI)(1mg/ml) for 10 minutes. Mitochondria were reisolated by centrifugation (16,000g, 4ºC) and resuspended in either 2X Laemmli sample buffer (65mM Tris-HCl pH 6.8, 25% [v/v] glycerol, 2% SDS, 0.01% bromophenol blue) for SDS-PAGE analysis or solubilization buffer (0.1mM EDTA, 50mM NaCl, 10% [v/v] glycerol, 20mM Tris-HCl pH 7.4, 1% [w/v] digitonin) for Blue Native-PAGE analysis. For NaCO3 extraction 100µg of mitochondria were pelleted after SBTI at 16,000g, 4ºC and resuspended in 200ul of 0.1M NaCO3 for 30 minutes on ice followed by ultracentrifugation (100,000g 30 minutes 4ºC). Supernatants were TCA-precipitated (10% [v/v] final) for 20 minutes on ice followed by centrifugation (16,000g, 4ºC, 20 minutes). The TCA precipitated supernatant and the pellet after ultracentrifugation were resuspended in 2X Laemmli sample buffer and proteins were separated by SDS-PAGE.

**Animal Experiments**

All animal experiments were carried out according to the German animal welfare act, considering the guidelines of the National Institute of Health and the 2010/63/EU Directive of the European Parliament on the protection of animals used for scientific purposes. The animals had free access to food and water and were kept in individually ventilated cages under a 12h:12h light/dark regime (light from 6:30 am to 6:30 pm), a constant 22±2°C temperature and 55±10% humidity.

The transgenic line B6.Cg-Chrm2tm1.1Hze/J, also known as Chrm2-tdT-D knock-in, was obtained from 'The Jackson Laboratory' (Stock No: 030330). They are heterozygous animals, designed to have expression of a Chrm2/tdTomato fusion protein directed by the endogenous M2 muscarinic acetylcholine receptor promoter/enhancer sequences.

**Tissue preparation and immunoblot of endogenous $M_2$ receptor in Chrm2-tdT-D knock-in mice**

Mouse tissues were weighed out and ice-cold lysis buffer was added to approx. 0.2-0.5mg tissue/ml buffer (10mM K2HPO4, 150mM NaCl, 5mM EDTA, 5mM EGTA, 1% Triton X-100, 0.2% Na-deoxycholate. Fresh protease inhibitors PMSF 0.5mM, Na3VO4 0.1mM, NaF 50mM, Jenny's mix (Soybean trypsin inhibitor 3.2µg/ml, Aprotinin 2µg/ml, Benzamidin 0.5mM) were added to the buffer.

Samples were first homogenized, and then pellet debris were centrifuged at 4°C for 30 minutes at max speed (tabletop) and the supernatant (soluble proteins) was transferred into fresh tube (pellets = non-soluble proteins)

Protein concentrations were determined with BCA assay. Cell lysates were loaded using Laemmli Buffer 2x (Sigma Aldrich) in equal mass (see above for the immunoblot procedure) into 8% polyacrylamide gels.

The membrane was then incubated overnight at 4 °C with primary antibody solution (1:1000 anti β-actin (13E5) rabbit primary antibody #4970 from Cell Signaling) (1:1000 anti GAPDH) (1:1000 anti-TdTomato rabbit primary antibody #632496 from Takara).

**Isolation of mitochondrial fractions and immunodetection**

HEK293 cells were seeded in 15 cm plates and transfected after 24h according to manufacturer protocols (Effectene, Qiagen) using 5 µg of plasmid DNA. 48h after transfection cells were harvested and centrifuged at 1000 rpm for 10 minutes, resuspended in NaCl 0.9% (w/v) and centrifuged at 1000 rpm for 10 minutes.

Mouse tissues were freshly excised and placed in ice cold PBS (1x). The samples were washed with NaCl 0.9% (w/v). The homogenization steps were performed with Precellys Evolution Homogenizer, at 4500 rpm for 10 seconds.

The samples were processed according to manufacturer protocols (Qproteome Mitochondria Isolation Kit, Qiagen). From the different steps were obtained three different fractions: the cytosolic, the microsomal and the mitochondrial fraction.

The fresh mitochondrial pellet was resuspended in 150µL of ice-cold lysis buffer and the protein concentration of the three fractions from each sample was quantified with a BCA assay (see above).

Cell lysates were loaded into 12% polyacrylamide gels using an SDS loading buffer (6x solution: 2.28g Tris-HCl, 2.313 DTT, 6g SDS, 30% Glycerol and 0.03% bromophenol blue in a final volume of 50 mL) in equal mass (see above for the immunoblot procedure).

The membrane was then incubated overnight at 4 °C with primary antibody solution using the following concentrations: 1:1000 anti β-actin (13E5) rabbit primary antibody (#4970 from Cell Signaling), 1:1000 anti-tdtomato rabbit primary antibody (#632496 from Takara), 1:1000 anti β-tubulin mouse primary antibody, 1:1000 anti GAPDH mouse primary antibody, 1:1000 anti COXIV mouse primary antibody (#ab33985 from abcam).

**Quantification of $M_2$tail(368-466) mitochondrial import kinetics from confocal time sequences** Before imaging, $2.5\times10^4$ HEK-TSA cells were seeded in each PDL-coated well of the µ-Slide 8 well (Ibidi) with DMEM (ScienCell™) supplemented with 10% FBS and 1% P/S overnight, and transfected with a total of 2 µg C-terminal EGFP tagged $M_2$ tail using Lipofectamine 2000 (Thermo Fisher Scientific) according to the manufacturer protocol after washing with 200 µL PBS and refreshing with 100 µL DMEM (ScienCell™) supplemented with 10% FBS and 1% P/S. After transfection for five hours, mitochondria of transfected cells were rapidly labelled with 50nM MitoTracker® Deep Red FM (Invitrogen™, Thermo Scientific) in FluoroBrite DMEM medium (Thermo Scientific) supplemented with 1% P/S for 20 minutes after the removal of old culture medium. After mitochondria labeling, the µ-Slide were washed twice with FluoroBrite DMEM medium (Thermo Scientific) and subsequently mounted onto the TCS SP8 confocal microscope (Leica) (See imaging). Firstly, the stage of the microscope was equilibrated in temperature, and the acquisition mode was set to the tile scanning. After storing the position of each desired transfected cells, the imaging mode was set to XYT and a HC PL APO CS2 40×/NA1.3 oil immersion objective was selected. Both DIC channel and HyD were utilized to obtain 512×512 pixels images. Laser wavelengths were set as described in the section Fluorescence Microscopy above. All settings remained the same throughout the whole measurement. Images were taken at every 30 minutes over a 5h interval. The images were afterwards analyzed by ImageJ software, using the Plugin 'JACoP'. Briefly, the degree of colocalization between EGFP-tagged $M_2$tail(368-466) (CH1) and MitoTracker stained mitochondria (CH2) was quantified by Mander's colocalization coefficients (40) [38]. The two colocalization coefficients M1

($M1 = \frac{CHB}{CH1}$) and M2 ($M2 = \frac{CHB}{CH2}$), CHB indicates overlapping pixels in channel 1 and channel 2. A threshold was determined for individual channel in order to distinguish specific signals from nonspecific ones. Due to their sensitivities to thresholds, the Pearson correlation coefficient (PCC)[36, 37] , as well as the faster decaying attribute of nonspecific signals, were also taken into account while estimating thresholds for each channel. For the analysis of each cell, the threshold for Mitotracker channel was kept constant. Whereas the threshold for EGFP channel was evaluated based on the PCC value.

**Transfection and expression of split-GFP constructs in HEK293 cells**

To perform the sequential split GFP complementation assay illustrated in **Figs 4D and 5C-D**, $3\times10^5$ HEK-TSA cells were seeded on a 10% poly-D-lysine (PDL)-coated 25mm coverslip in each well of a Falcon® 6-well flat-bottom plate (StemCell™) and cultured overnight. Cells were transfected using Effectene (see above) with a 0.4 µg Mito-GFP1-10 plasmid. The cells were then washed once with fresh 2 mL phosphate-buffered saline (PBS) first and supplemented with 1.5 mL fresh DMEM medium (ScienCell™) supplemented with 10% FBS and 1% P/S. After adding the transfection mixture, the cells were cultured overnight. After the first transfection, a second transfection of a 0.6 µg plasmid encoding either the C-terminal GFP11 tagged $M_2$tail(368-466) or $M_2$ receptor was performed using the Effectene Transfection Reagent (Qiagen). After washing each well with 2 mL PBS, the old medium was exchanged with 1.5 mL low glucose Minimum Essential Medium (MEM) (Gibco™, Thermo Scientific) supplemented with 1% P/S, in order to create a stress-like cell starvation condition. After a four hours starvation, the cells were fixed using 4% polyformaldehyde (PFA)/PBS at 37℃ for 30 minutes and washed twice with PBS before imaging.

**Oxygen consumption measured with the Seahorse assay**

$5x10^4$ COS-7 cells were seeded in seahorse 24MW plates with Complete media (DMEM supplemented with 10%FBS and 1X PS) and then incubated for about 4h at 37C in a cell incubator. After this incubation, cells were washed twice with Seahorse media supplemented with glutamate (1 mM), pyruvate (1 mM) and glucose (20 mM) and then incubated for 1h at 37 °C in an incubator without CO2 before being analyzed in the Seahorse XF Analyzers. In particular, the baseline cell oxygen consumption rate (OCR) was measured at the beginning of the assay, followed by measurements of ATP linked respiration and maximal respiratory capacity after the addition of respectively oligomycine

(1 µM) and the FCCP protonophore (1 µM). Finally, rotenone (1 µM), an inhibitor of complex I, and antimycin (1 µM), inhibitor of complex III, were added to shut down completely the electron transport chain (ETC) function, revealing the non-mitochondrial respiration.

**Extracellular oxygen consumption**

HEK293 cells 24h after transfection and hPSCs were harvested and seeded respectively in 96 multiwell black plate and pre-coated (Geltrex A1413302, Thermo Fisher) 96 multiwell black plate, $5x10^4$ cells for well; after incubation O/N at 37 °C and in 5% CO2, the cell culture medium was replaced by HBSS and the assay were performed according to the manufacturer protocols (abcam Extracellular Oxygen Consumption Assay).

**Generation of M368A hiPSC clone**

The human induced pluripotent stem cell (hiPSC) line BIHi005-A (https://hpscreg.eu/cell-line/BIHi005-A) was used to generate an isogenic iPSC clone (BIHi005-A-39) with M368A mutation using a protocol previously described (42) [40]. In brief, small guide RNA (sgRNA) targeting the $M_2$ gene close to the mutation site to be corrected were designed using the http://crispor.tefor.net/ webpage and the sgRNA (5`- AGTCATCTTCACAATCTTGC-3`) were synthesized by Integrated DNA technologies (https://eu.idtdna.com). The donor ssODN template was designed to convert the ATG to GCT and was synthesized as an Ultramar DNA oligo by IDT. (5´-AAAAGTGACTCATGTACCCCAACTAATACCACCGTGGAGGTAGTGGGGTCTTCAGGTCAGAATGGAGATGAAAAGCAGAATATTGTAGCCCGCAAGATTGTGAAGGCTACTAAGCAGCCTGCAAAA-3´). The Ribonucleoprotein (RNP) complexes were prepared by incubating of 1.5µg Cas-9 protein and 360 ng gRNA for 10 minutes at room temperature. For delivery of RNPs and ssODN template, 10µl cell suspension containing Cas9 RNPs and ssODN and 1x105 cells were electroporated using the Neon transfection System (ThermoFisher Scientific). The electroporated cells were plated in one well of 6 well plate with StemFlex media (ThermoFisher Scientific) supplemented CloneR™ (Stemcell technologies). Three days after the transfection we analyzed the bulk population using the Sanger sequencing to estimate the editing efficiency. Thereafter, the automated single cell cloning of the genome edited cell pool was performed as described in protocol [38] . The clones were screened by SANGER sequencing. The positive confirmed clones were banked and characterized for pluripotency and karyotype stability as described in [39] .

Briefly, for pluripotency assay, single cells were labelled with conjugated antibodies. Surface markers (SSEA1, SSEA4 and TRA1-60) staining was performed in unfixed cells by incubation with antibodies diluted in 0.5% BSA/PBS (10 min, 4 °C, dark). Intracellular markers (OCT3/4, NANOG) were stained by fixation/permeabilization solution (Miltenyi Biotec, 130-093-142) (30 min, 4 °C, dark) followed by antibody incubation in permeabilization buffer (Miltenyi Biotec) (30 min, 4 °C, dark). Analysis was done using the MACSQuant® AnalyzerVYB and FlowJo v10.4.

Briefly, for karyotype stability, genomic DNA was isolated using the DNeasy blood and tissue kit (Qiagen, Valencia, CA, United States) and samples were analyzed using the human Illumina OMNI-EXPRESS-8v1.6 BeadChip. First, the genotyping was analyzed using GenomeStudio 1 genotyping module (Illumina). Thereafter, KaryoStudio 1.3 (Illumina) was used to perform automatic normalization and to identify genomic aberrations utilizing settings to generate B-allele frequency and smoothened Log R ratio plots for detected regions. The stringency parameters used to detect copy number variations (CNVs) were set to 75 kb (loss), 100 kb (gain) and CN-LOH (loss of heterozygosity) regions larger than 3 MB.

**Culture and handling of hiPSCs cells**

hiPSCs were cultured on Geltrex (Thermo Fisher) coated plates using: Essential 8 medium (Thermo Fisher). The culture was routinely monitored for mycoplasma contamination using a PCR assay. 10 μM ROCK Inhibitor Y-27632 2HCl (Selleck Chemicals, #SEL-S1049) was added after cell splitting to promote survival. hiPSC cultures were kept in a humidified atmosphere of 5% CO2 at 37°C and 5% oxygen. hiPSCs were undirectedly differentiated to a mixture of three lineages (ectodermal, endodermal and mesodermal) by replacing Essential 8 medium with DMEM-F12 medium supplemented with 10% FBS for seven days. 10 μM ROCK Inhibitor Y-27632 2HCl (Selleck Chemicals) was added after cell splitting to promote survival.

**Cell proliferation assay with HEK293 cells**

$4x10^5$ HEK293 cells 24h after transfection and $4x10^5$ hPSCs were seeded respectively in T25 flask and pre-coated T25 flask, in culture medium with Atropine at final concentration of 100nM; the medium with Atropine was replaced every day. After 72h from transfection of the HEK293 and from seeding of the hiPSCs, the medium and the cells were collected and centrifuged, the pellet was resuspended in the same amount of medium for each pellet; an

aliquot of the resuspension was mixed with the same amount of Trypan blue (ratio 1:1), 10 microliters were used for counting in a Neubauer chamber.

**Fluorescence plate reader assays**

HEK293 cells were grown on 6 well plates without coverslips, and, upon reaching 60-70% confluence, the cells were transfected using Effectene (Quiagen, Venlo Netherlands) according to the manufacturer’s instructions. After 24 h the cells were split and transferred to 96 well (black plate) at a seeding density of 50,000 cells/well. The 96 well plate was pre-coated with Poly-D-Lysine for 30 min at RT. The medium was removed and each well was washed 3x with 100µl HBSS. The cells were finally analyzed by a fluorescence plate reader (Neo2, BioTek, Bad Friedrichshall) in HBSS.

**Statistical analysis**

Significance values reported in the graphs, in comparing tabulated values, were determined by a one-tailed Student t-test using Prism 9 (GraphPad Software), and significance values (p-values) are indicated according to the following legend: **** $p<0.0001$; *** $0.0001<p<0.001$; ** $0.001<p<0.01$; * $0.01<p<0.05$. p-values larger than 0.05 are deemed non-significant. Unless otherwise indicated, bar charts and scatter plots indicate average value with s.e.m.

## Acknowledgments

We are grateful to Dr. Marco Marcia (EMBL) for insightful discussion. I.P acknowledges a short term exchange grant from the Deutscher Akademischer Austauschdienst (DAAD). F.P. Acknowledges support from the University of L'Aquila towards international mobility.

## Funding:

European Union's Horizon2020 Marie Skłodowska-Curie Actions (MSCA)
Program under Grant Agreement 641833 (ONCORNET). This project was funded by the Deutsche Forschungsgemeinschaft (DFG, German Research Foundation) through Project 421152132 SFB1423 subproject C03 (P.A. and M.J.L.)

## Author contributions:

Conduced primary experiments: FP, IF, PA, ZW, MR, IP, RE, HZ, UZ, MET, JDG, SS, ART,SA, MC
Contributed to the experimental data: MG, CP, GA, FM, BP, AR, EC
Contributed tools and reagents: NT and SD
Contributed to experimental design and assisted in writing paper: PA, IP, IF, MR, UZ, AT, KT, VF, FA, FG, MS
Conceived and lead the study, and wrote the paper: PA, MJL and RM

## Competing interests:

The authors declare that they have no conflicts of interest with the contents of this article.

## Data and materials availability:

Source data will be provided with this manuscript.

## References

1. Kruse AC, Kobilka BK, Gautam D, Sexton PM, Christopoulos A, Wess J. Muscarinic acetylcholine receptors: novel opportunities for drug development. Nature Reviews Drug Discovery. 2014;13(7):549-60. doi: 10.1038/nrd4295.
2. Ad F, A C, Nm N. Muscarinic Receptors. 2012 ed: Springer Berlin Heidelberg; 2012.
3. Novi F, Scarselli M, Corsini GU, Maggio R. The Paired Activation of the Two Components of the Muscarinic M3 Receptor Dimer Is Required for Induction of ERK1/2 Phosphorylation. Journal of Biological Chemistry. 2004;279(9):7476-86. doi: 10.1074/jbc.M306818200.
4. Calebiro D, Nikolaev VO, Persani L, Lohse MJ. Signaling by internalized G-protein-coupled receptors. Trends in Pharmacological Sciences. 2010;31(5):221-8. doi: 10.1016/j.tips.2010.02.002.
5. Zhang X, Bedigian AV, Wang W, Eggert US. G protein-coupled receptors participate in cytokinesis. Cytoskeleton. 2012;69(10):810-8. doi: 10.1002/cm.21055.
6. !!! INVALID CITATION !!! [6, 7].
7. Maggio R, Fasciani I, Rossi M, Gregorio JD, Pietrantoni I, Puca V, et al. Variants of G protein-coupled receptors: a reappraisal of their role in receptor regulation. Biochemical Society Transactions. 2016;44(2):589-94. doi: 10.1042/BST20150239.
8. Jackson RJ, Kaminski A. Internal initiation of translation in eukaryotes: the picornavirus paradigm and beyond. RNA (New York, NY). 1995;1(10):985-1000.
9. Hellen CUT, Sarnow P. Internal ribosome entry sites in eukaryotic mRNA molecules. Genes & Development. 2001;15(13):1593-612. doi: 10.1101/gad.891101.
10. Popot JL, Engelman DM. Membrane protein folding and oligomerization: the two-stage model. Biochemistry. 1990;29(17):4031-7. doi: 10.1021/bi00469a001. PubMed PMID: 1694455.
11. Maggio R, Vogel Z, Wess J. Reconstitution of functional muscarinic receptors by co-expression of amino- and carboxyl-terminal receptor fragments. FEBS Letters. 1993;319(1-2):195-200. doi: 10.1016/0014-5793(93)80066-4.
12. Isbilir A, Serfling R, Moller J, Thomas R, De Faveri C, Zabel U, et al. Determination of G-protein-coupled receptor oligomerization by molecular brightness analyses in single cells. Nat Protoc. 2021;16(3):1419-51. Epub 20210129. doi: 10.1038/s41596-020-00458-1. PubMed PMID: 33514946.
13. Bathe-Peters M, Gmach P, Annibale P, Lohse MJ. Linescan microscopy data to extract diffusion coefficient of a fluorescent species using a commercial confocal microscope. Data in Brief. 2020;29:105063-. doi: 10.1016/j.dib.2019.105063.
14. Dong C, Filipeanu CM, Duvernay MT, Wu G. Regulation of G protein-coupled receptor export trafficking. Biochimica et Biophysica Acta (BBA) - Biomembranes. 2007;1768(4):853-70. doi: 10.1016/j.bbamem.2006.09.008.
15. Kamiyama D, Sekine S, Barsi-Rhyne B, Hu J, Chen B, Gilbert LA, et al. Versatile protein tagging in cells with split fluorescent protein. Nature Communications. 2016;7(1):11046-. doi: 10.1038/ncomms11046.
16. Newman LE, Schiavon C, Kahn RA. Plasmids for variable expression of proteins targeted to the mitochondrial matrix or intermembrane space. Cellular Logistics. 2016;6(4):e1247939-e. doi: 10.1080/21592799.2016.1247939.
17. Thakor N, Holcik M. IRES-mediated translation of cellular messenger RNA operates in eIF2α-independent manner during stress. Nucleic Acids Research. 2012;40(2):541-52. doi: 10.1093/nar/gkr701.
18. Holcik M, Sonenberg N. Translational control in stress and apoptosis. Nature Reviews Molecular Cell Biology. 2005;6(4):318-27. doi: 10.1038/nrm1618.
19. Bonfili L, Cecarini V, Cuccioloni M, Angeletti M, Flati V, Corsetti G, et al. Essential amino acid mixtures drive cancer cells to apoptosis through proteasome inhibition and autophagy activation. The FEBS Journal. 2017;284(11):1726-37. doi: 10.1111/febs.14081.
20. Ishizuka T, Ozawa A, Katsuura M, Nomura S, Satoh Y. Effects of muscarinic acetylcholine receptor stimulation on the differentiation of mouse induced pluripotent stem cells into neural progenitor cells. Clinical and Experimental Pharmacology and Physiology. 2018;45(11):1198-205. doi: 10.1111/1440-1681.12993.
21. Hoogduijn MJ, Cheng A, Genever PG. Functional Nicotinic and Muscarinic Receptors on Mesenchymal Stem Cells. Stem Cells and Development. 2009;18(1):103-12. doi: 10.1089/scd.2008.0032.
22. Weinberg ZY, Puthenveedu MA. Regulation of G protein-coupled receptor signaling by plasma membrane organization and endocytosis. Traffic. 2019;20(2):121-9. doi: 10.1111/tra.12628.
23. Jong Y-JI, Harmon SK, O'Malley KL. GPCR signalling from within the cell. British Journal of Pharmacology. 2018;175(21):4026-35. doi: 10.1111/bph.14023.

24. Bénard G, Massa F, Puente N, Lourenço J, Bellocchio L, Soria-Gómez E, et al. Mitochondrial CB1 receptors regulate neuronal energy metabolism. Nature Neuroscience. 2012;15(4):558-64. doi: 10.1038/nn.3053.
25. Suofu Y, Li W, Jean-Alphonse FG, Jia J, Khattar NK, Li J, et al. Dual role of mitochondria in producing melatonin and driving GPCR signaling to block cytochrome c release. Proceedings of the National Academy of Sciences. 2017;114(38). doi: 10.1073/pnas.1705768114.
26. Lefkimmiatis K, Leronni D, Hofer AM. The inner and outer compartments of mitochondria are sites of distinct cAMP/PKA signaling dynamics. Journal of Cell Biology. 2013;202(3):453-62. doi: 10.1083/jcb.201303159.
27. Marti-Solano M, Crilly SE, Malinverni D, Munk C, Harris M, Pearce A, et al. Combinatorial expression of GPCR isoforms affects signalling and drug responses. Nature. 2020;587(7835):650-6. doi: 10.1038/s41586-020-2888-2.
28. Pestova TV, Shatsky IN, Hellen CU. Functional dissection of eukaryotic initiation factor 4F: the 4A subunit and the central domain of the 4G subunit are sufficient to mediate internal entry of 43S preinitiation complexes. Molecular and Cellular Biology. 1996;16(12):6870-8. doi: 10.1128/MCB.16.12.6870.
29. Fitzgerald KD, Semler BL. Bridging IRES elements in mRNAs to the eukaryotic translation apparatus. Biochimica et Biophysica Acta (BBA) - Gene Regulatory Mechanisms. 2009;1789(9-10):518-28. doi: 10.1016/j.bbagrm.2009.07.004.
30. Jan E, Sarnow P. Factorless Ribosome Assembly on the Internal Ribosome Entry Site of Cricket Paralysis Virus. Journal of Molecular Biology. 2002;324(5):889-902. doi: 10.1016/S0022-2836(02)01099-9.
31. Ingolia NT, Hussmann JA, Weissman JS. Ribosome Profiling: Global Views of Translation. Cold Spring Harbor Perspectives in Biology. 2019;11(5):a032698-a. doi: 10.1101/cshperspect.a032698.
32. van Heesch S, Witte F, Schneider-Lunitz V, Schulz JF, Adami E, Faber AB, et al. The Translational Landscape of the Human Heart. Cell. 2019;178(1):242-60.e29. doi: 10.1016/j.cell.2019.05.010.
33. Guo C, Sun L, Chen X, Zhang D. Oxidative stress, mitochondrial damage and neurodegenerative diseases. Neural regeneration research. 2013;8(21):2003-14. doi: 10.3969/j.issn.1673-5374.2013.21.009.
34. Kritsiligkou P, Chatzi A, Charalampous G, Mironov A, Grant CM, Tokatlidis K. Unconventional Targeting of a Thiol Peroxidase to the Mitochondrial Intermembrane Space Facilitates Oxidative Protein Folding. Cell Reports. 2017;18(11):2729-41. doi: 10.1016/j.celrep.2017.02.053.
35. Bonner TI, Buckley NJ, Young AC, Brann MR. Identification of a Family of Muscarinic Acetylcholine Receptor Genes. Science. 1987;237(4814):527-32. doi: 10.1126/science.3037705.
36. Manders EM, Stap J, Brakenhoff GJ, van Driel R, Aten JA. Dynamics of three-dimensional replication patterns during the S-phase, analysed by double labelling of DNA and confocal microscopy. Journal of Cell Science. 1992;103(3):857-62. doi: 10.1242/jcs.103.3.857.
37. Manders EMM, Verbeek FJ, Aten JA. Measurement of co-localization of objects in dual-colour confocal images. Journal of Microscopy. 1993;169(3):375-82. doi: 10.1111/j.1365-2818.1993.tb03313.x.
38. Vallone VF, Telugu NS, Fischer I, Miller D, Schommer S, Diecke S, et al. Methods for Automated Single Cell Isolation and Sub-Cloning of Human Pluripotent Stem Cells. Current Protocols in Stem Cell Biology. 2020;55(1). doi: 10.1002/cpsc.123.
39. Metzler E, Telugu N, Diecke S, Spuler S, Escobar H. Generation of two human induced pluripotent stem cell lines derived from myoblasts (MDCi014-A) and from peripheral blood mononuclear cells (MDCi014-B) from the same donor. Stem Cell Research. 2020;48:101998-. doi: 10.1016/j.scr.2020.101998.

Figure 1

Click here to access/download;Figure;Figure 1_PlosBio.eps

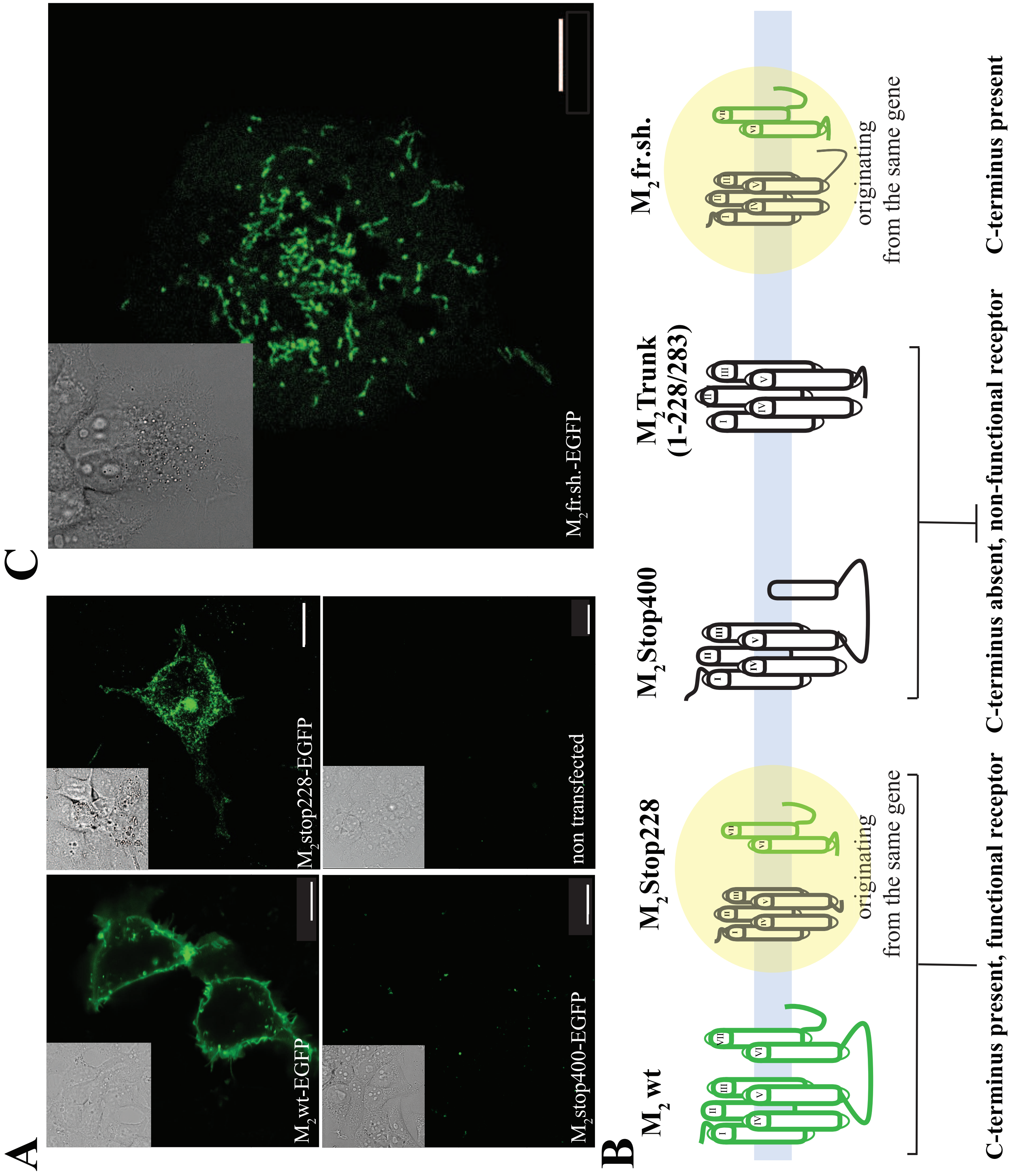

Figure 2

Click here to access/download;Figure;Figure 2_PlosBio.tif

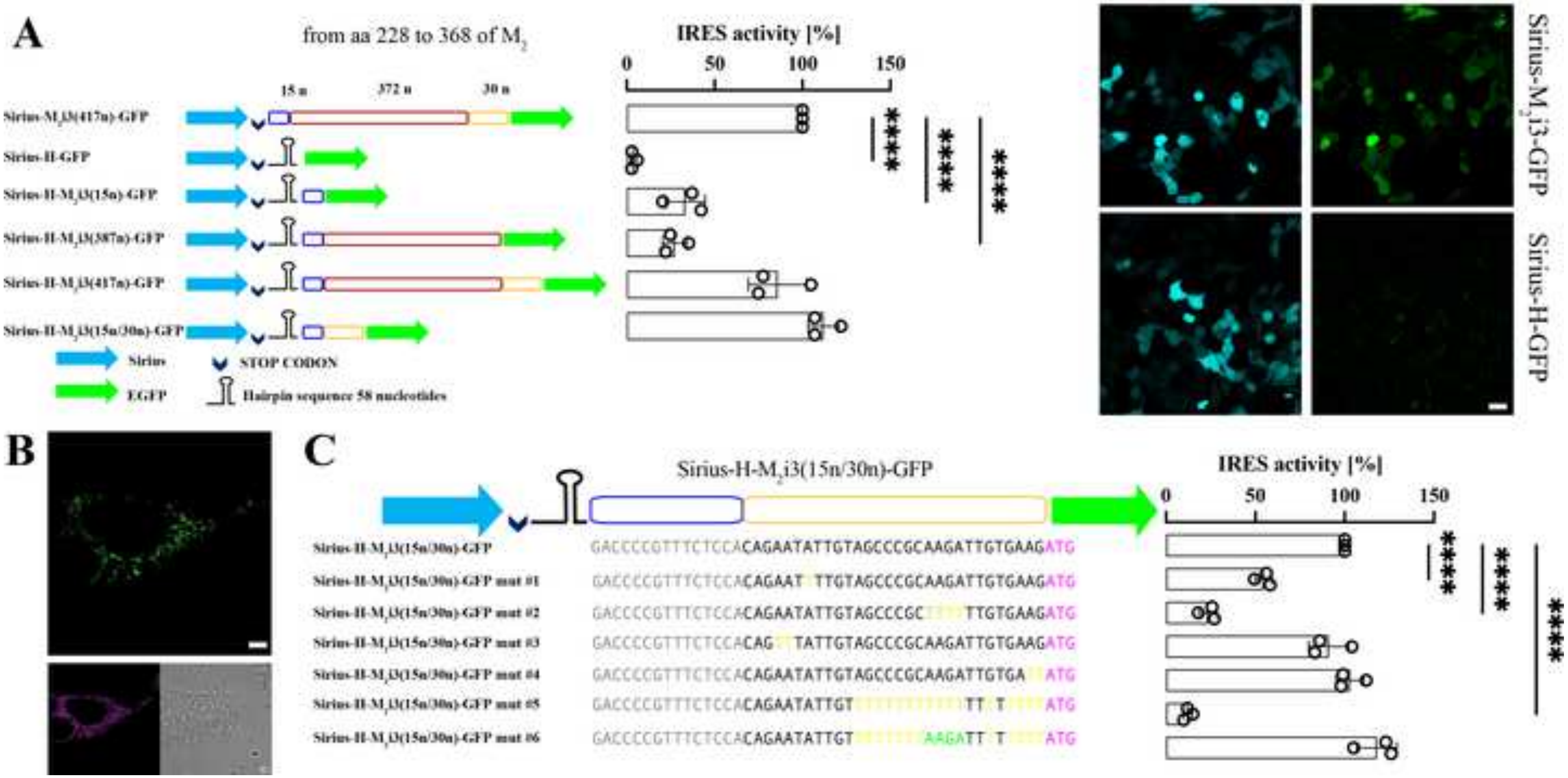

Figure 3

Click here to access/download;Figure;Figure 3_PlosBio.eps

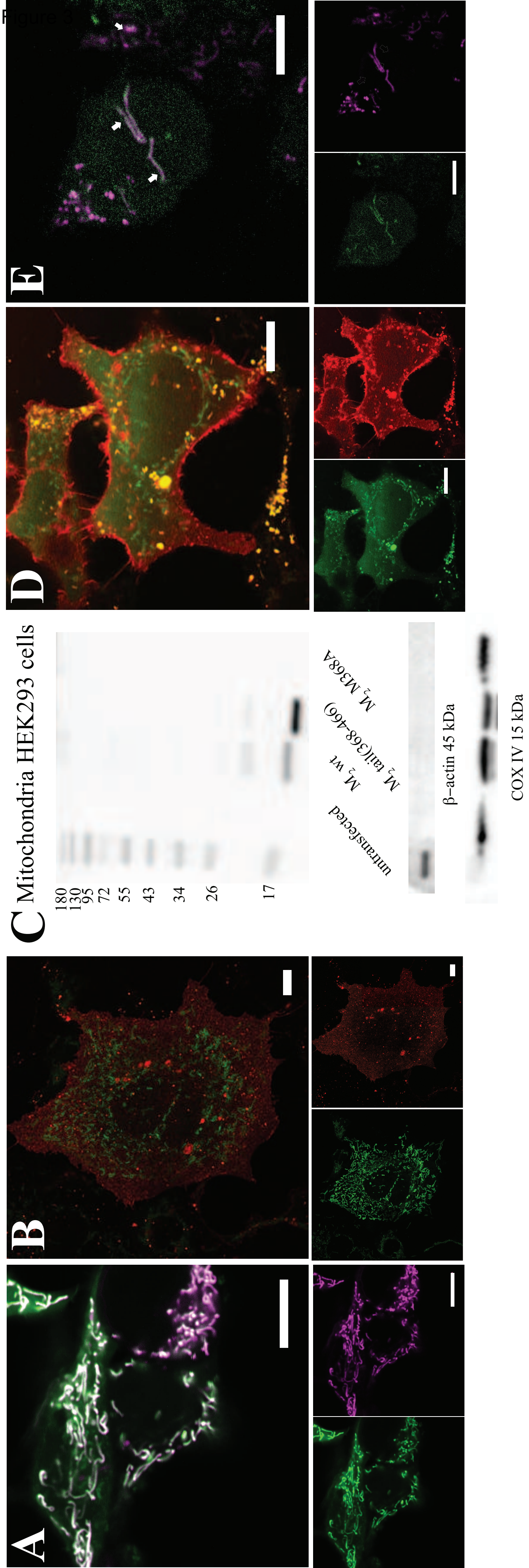

Figure 4

Click here to access/download;Figure;Figure 4_PlosBio.eps

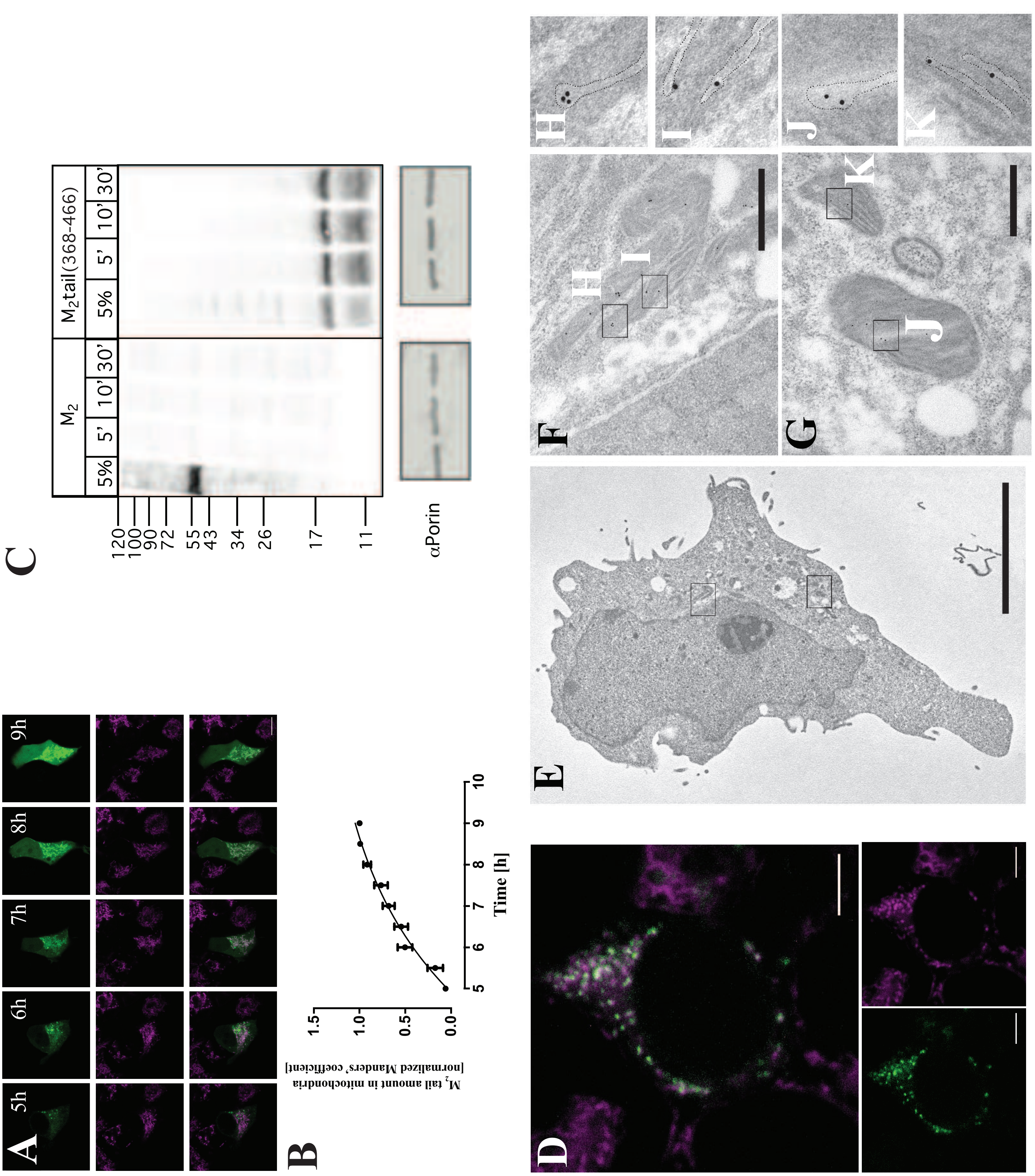

Figure 5 Click here to access/download;Figure;Figure 5_PlosBio.eps

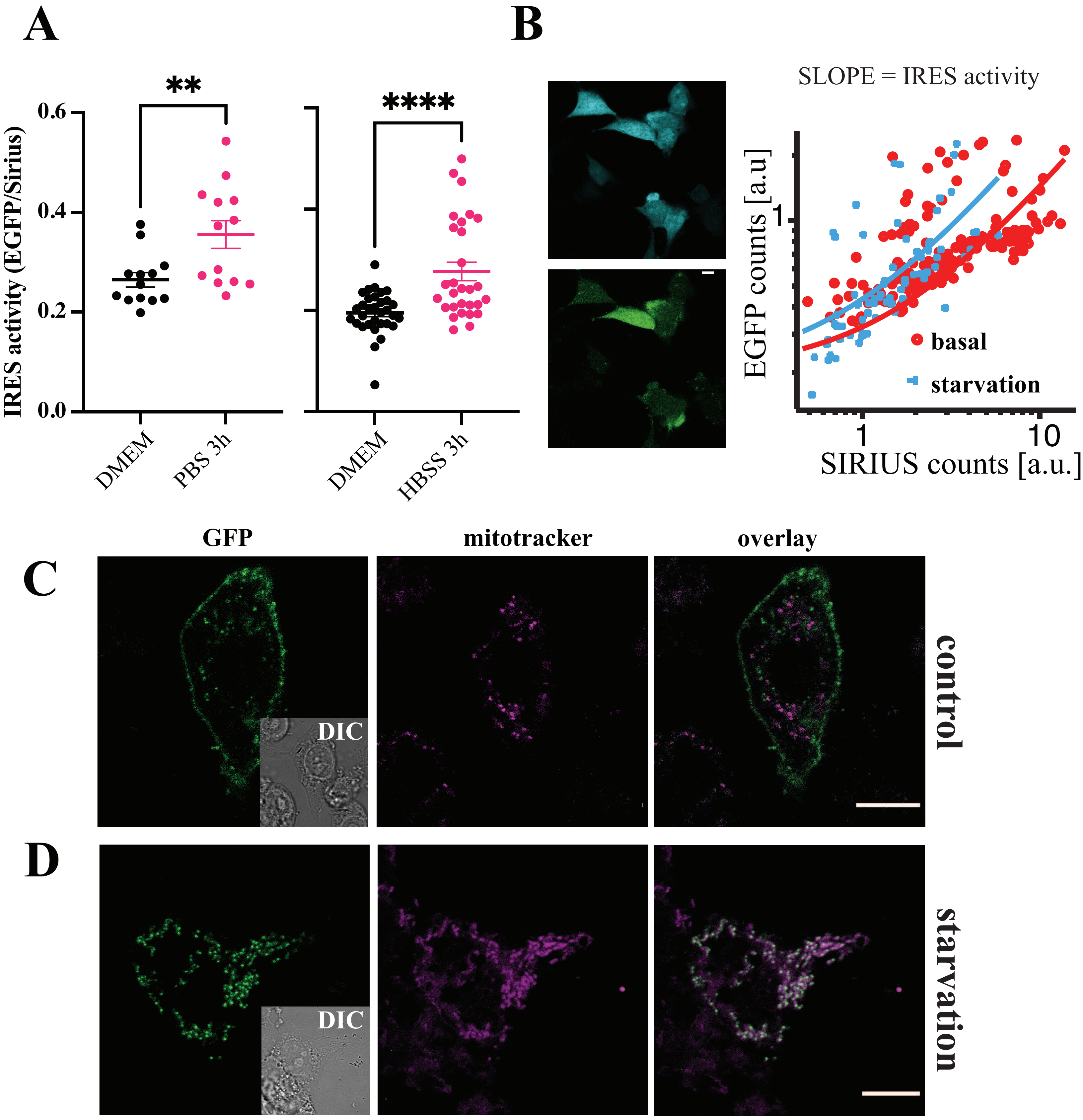

Figure 6

Click here to access/download;Figure;Figure 6_PlosBio.eps

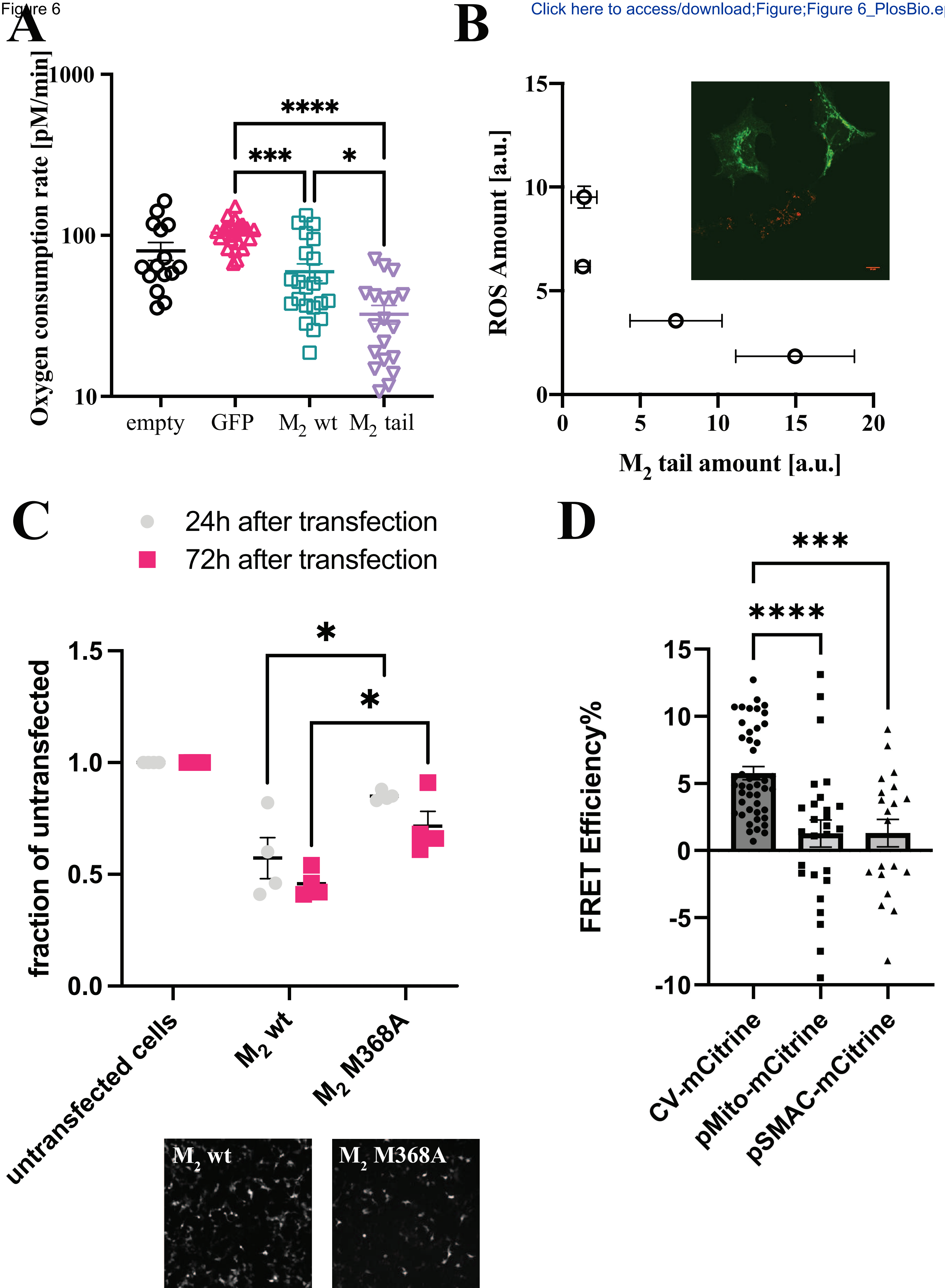

Figure 7

Click here to access/download;Figure;Figure 7_PlosBio.eps

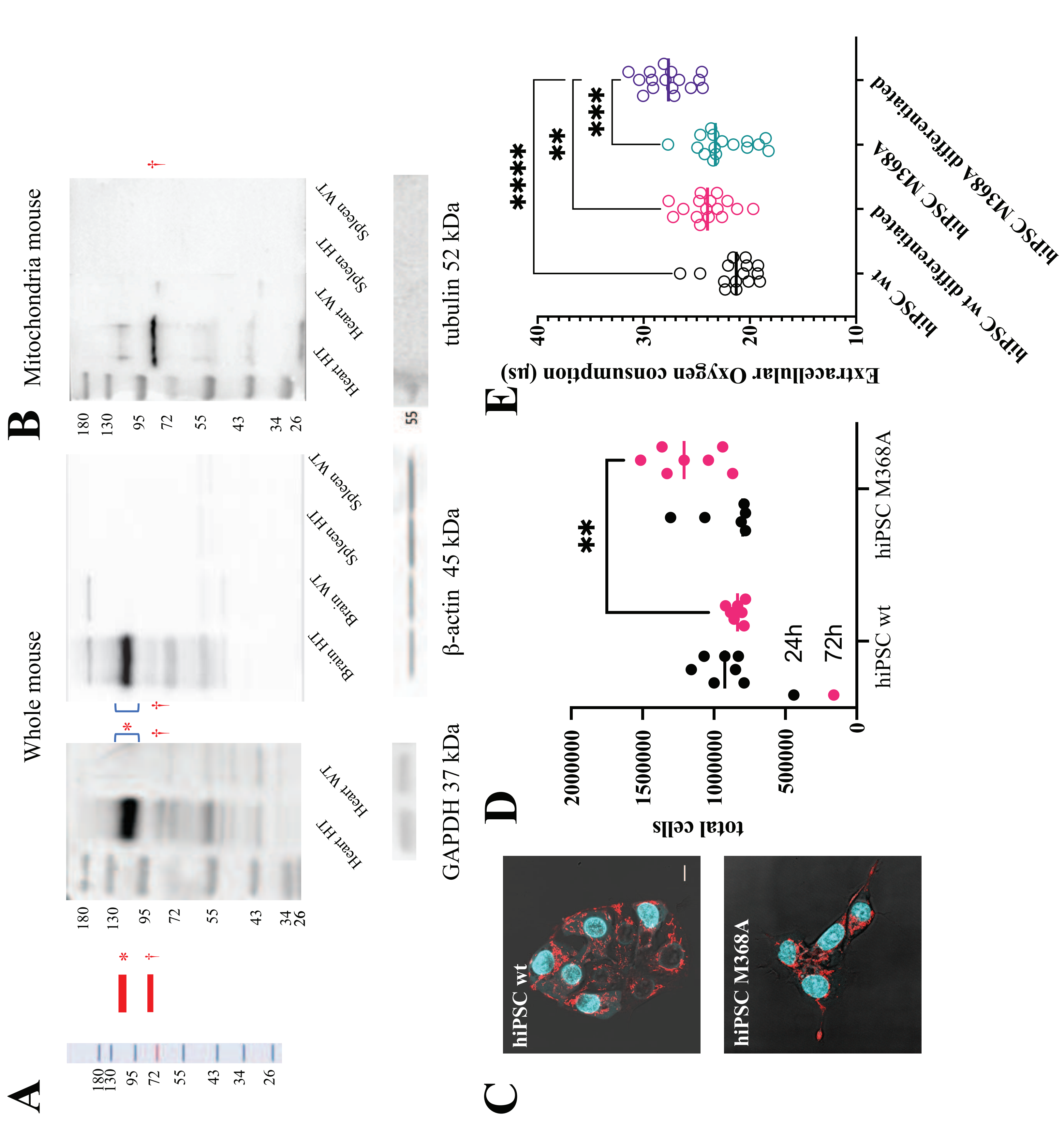

Figure 8

Click here to access/download;Figure;Figure 8_PlosBio_highRes.tif

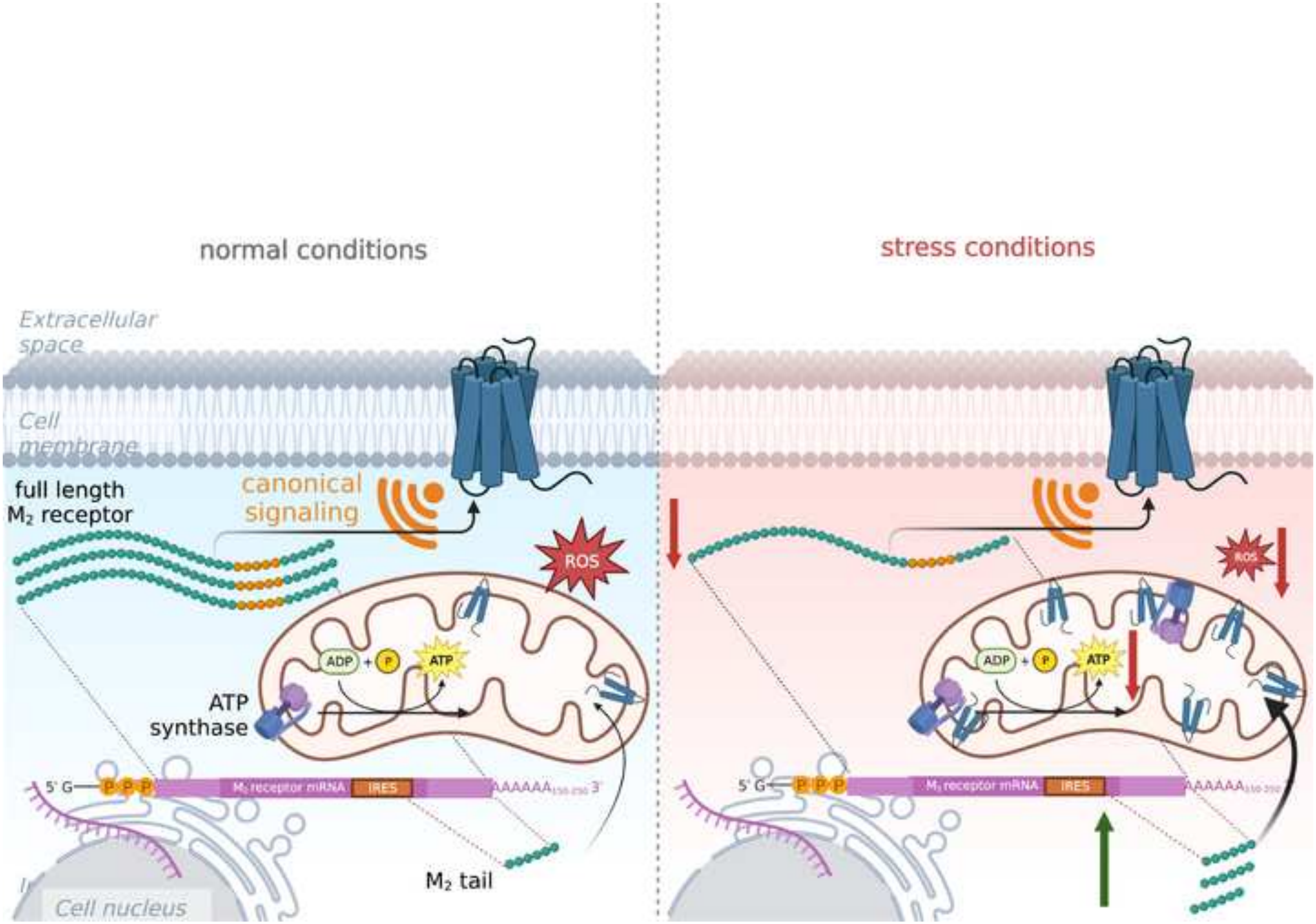

Click here to access/download
**Supporting Information**
IRES_manuscript_SI_09112023_clean.docx

Revised SI with Changes Highlighted

Click here to access/download
**Other**
IRES_manuscript_SI_09112023_annotated.docx

Western Blot Source Data

Click here to access/download
**Other**
Blots_supporting_data.pdf

Revised Article with Changes Highlighted

# One gene - two proteins: The C-terminus of the prototypical M2 muscarinic receptor localizes to the mitochondria and regulates cell respiration.

Irene Fasciani[1†], Francesco Petragnano[1†], Ziming Wang[2], Ruairidh Edwards[3], Narasimha Telegu[2], Ilaria Pietrantoni[1], Ulrike Zabel[4], Henrik Zauber[2], Marlies Grieben[2], Maria E. Terzenidou[3], Jacopo Di Gregorio[1], Cristina Pellegrini[1], Silvano Santini Jr[5], Anna R. Taddei[6], Barbel Pöhl[2], Stefano Aringhieri[7], Marco Carli[7], Gabriella Aloisi[1], Francesco Marampon[8], Eve Charlesworth[9], Alexandra Roman[2], Sebastian Diecke[2], Vincenzo Flati[1], Franco Giorgi[7], Fernanda Amicarelli[5], Andrew B. Tobin[3], Marco Scarselli[7], Kostas Tokatlidis[3], Mario Rossi[1], Martin J. Lohse[2,4,10]*, Paolo Annibale[2,9]*, Roberto Maggio[1]*

[1] Department of Biotechnological and Applied Clinical Sciences, University of L'Aquila, 67100 L'Aquila, Italy.
[2] Max Delbrück Center for Molecular Medicine, 13125 Berlin, Germany.
[3] Centre for Translational Pharmacology, Institute of Molecular, Cell and Systems Biology, College of Medical, Veterinary and Life Sciences, University of Glasgow, G128QQ Glasgow, UK.
[4] Institute of Pharmacology and Toxicology, University of Würzburg, 97078 Würzburg, Germany.
[5] Department of Life, Health and Environmental Sciences, University of L'Aquila, 67100 L'Aquila, Italy.
[6] Section of Electron Microscopy, Great Equipment Center, University of Tuscia, Viterbo, Italy.
[7] Department of Translational Research and New Technology in Medicine, University of Pisa, 56126 Pisa, Italy.
[8] Department of Radiotherapy, University of Roma La Sapienza, 00151 Roma, Italy.
[9] School of Physics and Astronomy, University of St Andrews, KY16 9SS St Andrews.
[10] ISAR Bioscience Institute, 82152 Munich, Germany.
[†] Equal contribution
*email: martin.lohse@isarbioscience.de;
*email: roberto.maggio@univaq.it;
*email: paolo.annibale@mdc-berlin.de, pa53@st-andrews.ac.uk

## Abstract

**Muscarinic acetylcholine receptors are prototypical G protein-coupled receptors (GPCRs), members of a large family of seven transmembrane receptors mediating a wide variety of extracellular signals. We show here, for the first time in a GPCR, that the carboxyl terminal fragment of the muscarinic $M_2$ receptor, comprising the transmembrane regions 6 and 7 ($M_2$tail), is expressed by virtue of an internal ribosome entry site located in the third intracellular loop. Single cell imaging reveals that $M_2$tail, whose expression is upregulated in cells undergoing integrated stress response, does not follow the normal route to the plasma membrane, but is almost exclusively sorted to the mitochondria inner membrane: here it controls oxygen consumption, cell proliferation and the formation of reactive oxygen species by reducing oxidative phosphorylation. Crispr/Cas9 editing of the key methionine where cap-independent translation begins in human induced pluripotent stem cells, reveals the physiological role of this process in influencing cell proliferation and oxygen consumption at the endogenous level. The expression of the C-terminal domain of a GPCR, capable of regulating mitochondrial function, constitutes a hitherto unknown mechanism notably unrelated to its canonical signalling function as a GPCR at the plasma membrane. This work thus highlights a potential novel mechanism thar cells may use for controlling their metabolism under variable environmental conditions, notably as a negative regulator of cell respiration.**

## Introduction

Muscarinic receptors include five structurally related receptors, belonging to class A of the G protein-coupled receptor (GPCR) family [1]. They are comprised of seven transmembrane domains (TM domains 1-7) connected by intra and extracellular loops. Of these structural elements, the third intracellular (i3) domain is the one conferring a coupling specificity for G proteins, β-arrestin recruitment and endocytic internalization [2]. However, only a small portion of the i3 loop is required for receptor function, and for this reason large deletions of this segment have no effect on receptor activation and internalization [3].

GPCRs are normally distributed at the plasma membrane, where, after activation by an agonist and coupling to either G proteins or β-arrestin, regulate a number of intracellular effectors. Nevertheless, non-canonical localizations of GPCRs have also been described, such as in

endosomes [4], cell division compartments (centrosomes, spindle midzone and midbodies) [5] and mitochondria [6], underpinning non-traditional roles for this protein family.
Several studies have shown that the GPCR structurally related transmembrane protein bacteriorhodopsin consists of autonomous folding domains that translocate to the membrane and interact with each other to function as a proton pump. Interestingly, it was also shown that fragments of muscarinic receptors can interact to form functionally active chimeric receptors, highlighting the conserved evolutionary ability of domains from these receptor family to interact among each other, as well as with other proteins. Intriguingly, in 2016, we observed that the insertion of a stop codon in the third intracellular loop of the muscarinic $M_2$ receptor was not able to abolish its function [7], presenting the suggestive possibility that a cap-independent translation of the $M_2$ C-terminal domain may occur in this mutant, i.e. that the $M_2$ mRNA may lead to the translation not only of the full length receptor, but also of its C-terminal domain, that we name here $M_2$tail.

In this work, we therefore set out to explore whether the expression of the $M_2$tail, a region containing the TM regions 6 and 7, occurs naturally by a cap-independent translation mechanism. Combining radio-ligand binding, molecular biology approaches, advanced fluorescence imaging and Crispr/Cas9 genetic editing of human induced pluripotent stem cells (hiPSCs) we have gathered compelling data indicating a novel translation mechanisms for C-terminal domain of the $M_2$ muscarinic receptor. This translation mechanism appears to be regulated by an internal ribosome entry site (IRES) sequence within the M2 mRNA allowing the independent expression of the receptor's C-terminal domain as a distinct polypeptide.under control of a IRES sequence within the $M_2$ mRNA and that the C-terminal domain of this receptor can indeed be expressed as an independent polypeptide.

While in eukaryotes the most common mechanism of translation initiation is the cap-dependent process, which requires the interaction of several key proteins at the 5'-end (5′ cap) of mRNA molecules, recent years have seen growing evidence that internal ribosome entry site (IRES) sequences, which are common features of virus replication [8], can mediate cap independent translation of eukaryotic RNA(s) [9].

Moreover, by using bimolecular fluorescence complementation, in-vitro mitochondrial import and cell metabolism assays we observe that $M_2$tail is strongly targeted to the cell mitochondria. Importantly, this mitochondrial targeting has functional ramifications since both cap-dependent

mediated overexpression of $M_2$tail, as well as cap-independent mediated translation starting from the mRNA of the full length $M_2$ receptor, resulted in a sizable reduction of oxidative phosphorylation. In fact, we show that cellular stress favours the activation of this newly discovered IRES-dependent translation of $M_2$tail and that the fragment interacts with the Complex V of the respiratory chain on the mitochondria inner membrane. The implication of this newly discovered regulatory mechanism of the IRES mediated translation of the $M_2$ C-terminal domain might have profound physiological and pathophysiological implications.

In summary our study reports the new observation that alternative reading of a GPCR receptor gene can translate into a fragment of the receptor that serves a cellular function entirely different from canonical GPCR signalling**.** Taken together these data strongly suggest that the $M_2$ mRNA yields not only a plasma membrane receptor able to transduce many of the acetylcholine functions but $M_2$tail, a protein fragment that reduces the activity of mitochondria and ROS accumulation and it might represent a hitherto unappreciated step for in-vivo anti-oxidative stress, during for instance reperfusion, tissue damage, and disease-promoted metabolic cellular stress.

## Results

**Cap independent production of the C-terminus of the $M_2$ receptor and evidence for an IRES**

We began our work by testing our earlier hypothesis that the last two TM regions (TM6-7) of $M_2$stop228, a $M_2$ muscarinic receptor mutant bearing a stop codon at the beginning of the i3 loop in position 228 (**Fig. S1**), may still be translated [10]. We thus generated EGFP C-terminal fusion of the $M_2$ wt receptor and $M_2$stop228: **Fig. 1A** shows that cells expressing $M_2$stop228-EGFP display a green fluorescence signal. Moreover, the signal displays a predominant intracellular localization with respect to the wt $M_2$R, which instead localizes -as expected- largely at the plasma membrane. A key observation is that the specific location position of the stop codon upstream of the i3 loop appears to havebear a particular relevance., For istance, since if a stop is instead inserted downstream of position 228, e.g. at positions 400, right before the first methionine in TM6, no fluorescence can be observed in cells expressing the mutant $M_2$stop400-EGFP **Fig. 1A.**

To further appreciate the origin of this unexpected process -the 228 stop codon should in principle abolish any EGFP signal- we performed radioligand binding experiments in cells transfected with

this mutant. $M_2$stop228 exhibited ~~showed both~~ $K_D$ value for~~of~~ the antagonist [$^3$H]NMS (N-methylscopolamine~~, an antagonist~~) calculated from direct saturation experiments, as well as ~~and the~~ $IC_{50}$ values, ~~calculated~~ obtained in competition experiments for the agonist carbachol, similar to those calculated for the wild type $M_2$ receptor (**Table 1**), albeit displaying a very low level of binding sites ~~expression~~ at the plasma membrane, specifically~~i.e.~~ 45 fmol/mg of protein. Furthermore, $M_2$stop228 was found functionally active as it was capable to inhibit adenylyl cyclase [10] and increase ERK phosphorylation (**Fig. S2A**), even though the extent of response was reduced if compared to the wild type $M_2$ receptor (**Table 1**). As a control, receptor fragments, translated from mRNAs comprising only the sequence for TM1-5 (i.e. whose sequences run only until position 228 or 283 of the corresponding full length receptor), called $M_2$trunk(1-228) or ~~/~~$M_2$trunk(1-283), did not show any radio-ligand binding activity, despite coding for the same first five trans-membrane regions (TM1-5) that the canonical translation of $M_2$stop228 should also produce (**Fig. 1B** and **Table 1**) [11].

Notably, our previous research has demonstrated the ability of distinct fragments of muscarinic receptors, cotransfected together, to reassemble into a fully functionally receptors. Specifically, w~~W~~e showed ~~in the past~~ that the last two TMs (TM6-7) of the $M_2$ receptor can combine with ~~reconstitute a functional receptor with~~ TM1-5 to recreate a functional receptor [11] (**Table 1**), supporting the notion that the explanation of the apparent paradox of $M_2$stop228 lies in the independent translation of ~~must be that~~ $M_2$tail ~~is translated~~ as a distinct ~~an independent~~ polypeptide, as graphically summarized in **Fig. 1B**.

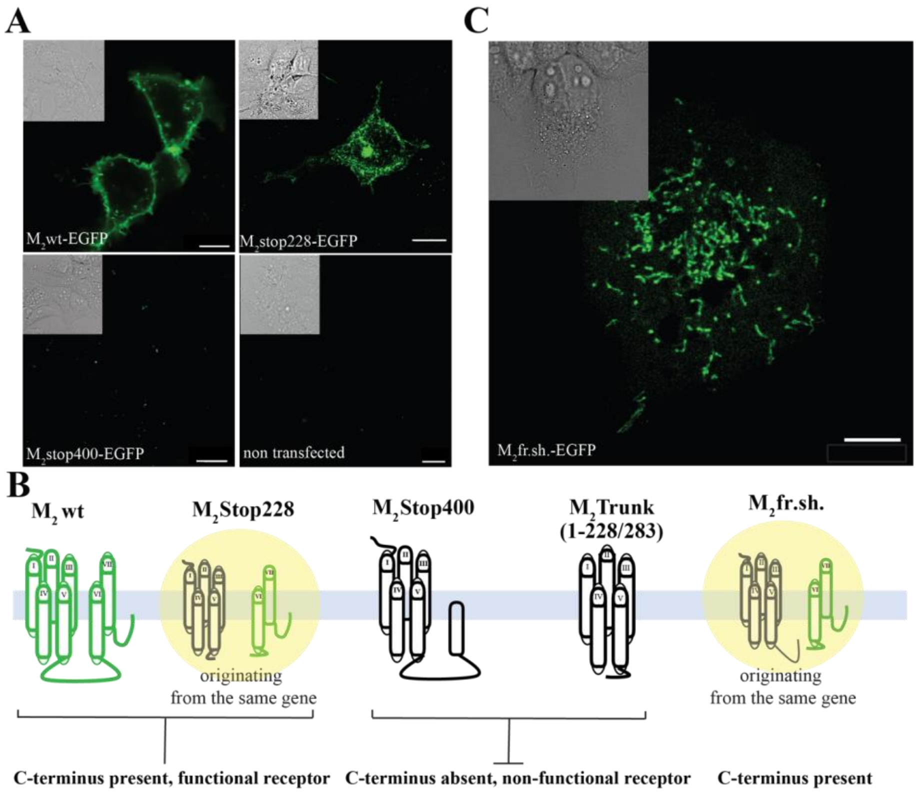


**Fig. 1. The c-terminus of the $M_2$ receptor is expressed by a cap independent translation mechanism, originating in the i3loop.**

**A**, Fluorescence micrographs of HEK293 and COS7 cells respectively expressing $M_2$-EGFP, $M_2$stop228-EGFP, $M_2$stop400-EGFP, together with an untransfected control. **B**, Schematic representation of the gene products that reach the plasma membrane of the wild type $M_2$, $M_2$stop228, $M_2$trunk(1-283) and $M_2$fr.sh. (**Fig. S3**). **C**, Fluorescence micrograph of a representative HEK293 cell expressing $M_2$fr.sh.-EGFP. Insets display DIC image of the same field of view (scale bar 10 µm). Imaging settings are the same for the f images, whereas contrast was adjusted to improve visualization. In the untransfected cells and stop400 panels, the contrast was set to 0. Insets display DIC image of the same field of view (scale bars 10 µm).

| Binding data | | | | Adenylyl cyclase inhibition assay | |
|---|---|---|---|---|---|
| Receptor | Bmax (fmol/mg) | [$^3$H]NMS KD (pM) | Carbachol IC50 (μM) | Carbachol EC50 (μM) | Maximum inhibition of forskolin stimulated cAMP accumulation (%) |
| $M_2$ | 980 ± 60 | 62 ± 7 | 2.1 ± 0.6 | 0.04 ± 0.01 | 53 ± 5 |
| $M_2$trunk(1-283) | N.B. | | | | |
| $M_2$trunk(1-228) | N.B. | | | | |
| $M_2$stop228 | 45 ± 2 | 70 ± 10 | 1.8 ± 0.4 | 0.13 ± 0.02 | 27 ± 3 |
| M2trunk(1-283) +M2tail(M-281-466) | 480 ± 20 | 79 ± 10 | 0.7 ± 0.2 | 0.15 ± 0.03 | 37 ± 4 |
| M2trunk(1-228) +M2tail(368-466) | 70 ± 10 | 72 ± 8 | 0.6 ± 0.2 | 0.17 ± 0.03 | 29 ± 4 |

**Table 1. Radioligand binding and activation properties of $M_2$ receptor mutants.**

Binding properties and inhibition of forskolin stimulated adenylyl cyclase by $M_2$, $M_2$stop228, $M_2$trunk(1-228), $M_2$trunk(1-283), and cotransfected M2trunk(1-283) + M2tail(M-281-466) and. M2trunk(1-228) + M2tail(368-466). Untransfected COS-7 cells did not show any [$^3$H]NMS specific binding. N.B. = no specific [$^3$H]NMS binding. Data are averages with s.e.m.

### Evidence of an IRES-mediated $M_2$ C-terminus translation

$M_2$stop228 mRNA appears thus able to give rise -despite the presence of a stop codon in the i3 loop- to two distinct protein products (a *trunk* TM1-5 and a *tail* TM6-7), in turn able to reconstitute a functional receptor at the plasma level.

To challenge this hypothesis and gain further insight into the mechanism at play, we generated a frame shift mutant of the $M_2$ receptor: we inserted a single guanine nucleotide in the sequence of the $M_2$-EGFP, changing the methionine in position 368 into an aspartate, which in turn generates an in-frame stop codon at position 370 (**Fig. S1 and Fig. S3**). The observation that- cells transfected with $M_2$fr.sh.-EGFP retain green fluorescence is most remarkable (**Fig. 1C**), since the

EGFP is now out of the original reading frame and it can only be properly translated by a *cap-independent translation mechanism*, starting from a methionine in the new reading frame. Furthermore, the observation that both $M_2$stop228-EGFP and $M_2$fr.sh.-EGFP retain green fluorescence, whereas $M_2$stop400-EGFP does not, indicates not only that a cap independent translation mechanism occurs, but also that the starting methionine for the process must be downstream of stop 228 and upstream of stop 400, leaving as a leading candidate one of the in-frame methionines of the i3 loop.

To narrow this down, we employed radio-ligand binding data from expression of receptor mutants carrying a double stop codon, the first in position 228 and the second in positions ranging from 248 to 400, including mutations of the first (M248) and second (M296) in-frame methionines in the third i3 loop (**Table S1**, **Fig. S3**). The observation that mutation of the first two methionines does not affect $B_{max}$ points to M368 as the most likely site for the cap-independent initiation of translation of the $M_2$tail, that we now term more specifically $M_2$tail(368-466). These binding results can furthermore rule out read-through and termination re-initiation: if these mechanisms were responsible for translation of the C-terminal of the $M_2$stop228, suppression of the first methionine would have abolished the binding. We shall further note that cells expressing $M_2$stop228 showed only one single $M_2$ specific cDNA band supporting the concept that the $M_2$tail(368-466) was produced from the same $M_2$-mRNA molecule by a cap independent mechanism instead of, for instance, by RNA splicing variants (**Supplementary Materials and Methods** and **Fig. S2B**). However, upon immunoblotting the protein products it is immediate to observe that the full length $M_2$ receptor produces a set of low molecular weight band matching those observed for $M_2$tail(368-466), when the constructs posses a C-terminal tag (myc) (**Fig. S2C**). In contrast N-terminally tagged $M_2$stop228 produces a band in line with that observed for cells transfected with the $M_2$trunk(1-228) fragment, further ruling out stop-cordon readthrough for the our findings (**Fig. S2D**). Comparable results are observed in COS-7 cells (**Fig. S2E**).

## IRES position within the $M_2$ coding sequence

To further validate the results obtained working with full length $M_2$ receptor mutants and ~~screen for what~~to investigate the critical nucleotides ~~are important~~ for ~~the~~ IRES activity, we generated ~~additionally prepared~~ a fluorescence reporter construct (Sirius-$M_2$i3(417n)-EGFP) in which the i3 loop of $M_2$ was inserted between the two fluorescent proteins, Sirius and EGFP (**Fig. 2A**). The sequence for Sirius is followed by a stop codon, which means that ~~thus making~~ Sirius-$M_2$i3(417n)-EGFP translates Sirius according to the classical cap-dependent mechanism, serving as an indicator of ~~reporting on~~ transfection efficiency, and, at the same time, drives EGFP translation

**Formatted:** Font: Not Bold
**Formatted:** Font: Not Bold, Subscript
**Formatted:** Font: Not Bold
**Formatted:** Font: Not Bold, Subscript
**Formatted:** Font: Not Bold
**Formatted:** Highlight
**Formatted:** Font: Not Bold
**Formatted:** Subscript
**Formatted:** Subscript

only if the $M_2$i3 loop contains an IRES sequence, ~~effectively~~ essentially functioning ~~acting~~ as a bicistronic expression cassette. We transfected several cell lines with this construct, and all of them were positive for the blue and green fluorescence (**Fig. S4A**). HEK293 cells, used throughout our study, appeared positive for both the blue and green fluorescence, as shown in the inset of **Fig. 2A** and in line with the results reported in **Fig. 1A**. To further rule out the possibility that EGFP translation from the Sirius-$M_2$i3(417n)-EGFP construct could be ~~due~~ attributable to read-through and termination-re-initiation mechanisms, a mRNA hairpin loop was inserted in the sequence after the stop codon of the Sirius protein and before the $M_2$i3(417n), resulting in ~~(~~Sirius-H-$M_2$i3(417n)-EGFP~~Sirius-H-EGFP),~~. This modification did not eliminate~~d~~ ~~abolishing completely~~ the EGFP signal, showing only a slight reduction of the EGFP/Sirius with respect to the value measured for the analogous reporter without hairpin (~90%) (**Fig. 2A**), underscoring that read-through and termination-re-initiation mechanisms do not appear to be at play. In contrast, when the $M_2$i3(417n) segment was removed from Sirius-H-$M_2$i3(417n)-EGFP, yielding Sirius-H-EGFP, it entirely abolished EGFP expression (**Fig. 2A**). This observation indicates that the hairpin loop effectively blocks read-through and termination-re-initiation mechanisms

To narrow down the position of the IRES sequence we divided the $M_2$ i3 loop in three parts and the resulting nucleotide sequences, 685-699, 685-1071 and 685-699/1072-1101, were inserted downstream of the hairpin loop in three different constructs (**Fig. 2A** and **Fig. S3**).

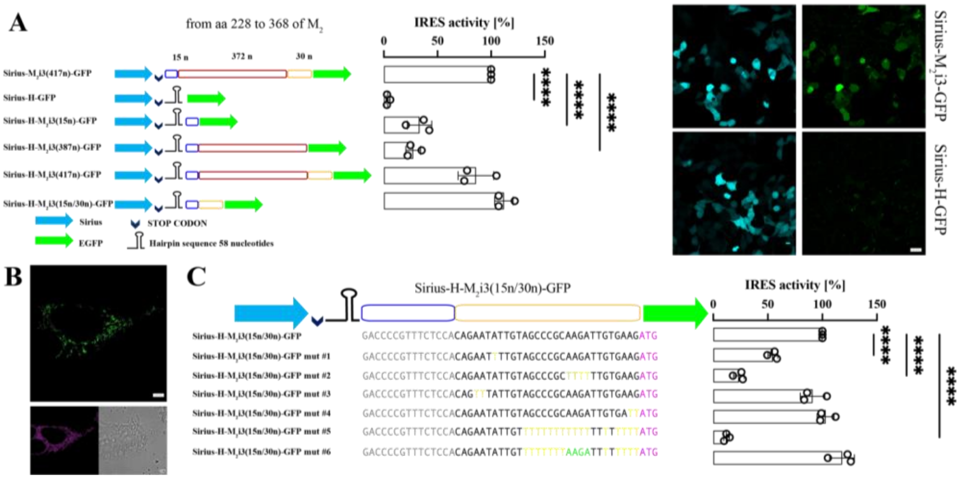


**Fig. 2. Localization of the IRES within the i3 loop the muscarinic $M_2$ receptor using a fluorescence reporter assay.**

**A**, **left** Schematic representation of bicistronic plasmids bearing the M$_2$ i3 loop between the coding regions of the Sirius and EGFP fluorescent proteins. A guanine nucleotide was originally inserted before the EGFP in the plasmid Sirius-H-EGFP in order to maintain in frame the sequences of the two fluorescent proteins. In all the other plasmids the sequence of the two fluorescent proteins is not in frame. Fifteen nucleotides derived from the M$_2$ i3 loop were inserted between the end of Sirius and the hairpin loop, in order to allow the easy scanning of the ribosome across the stop codon. **right**, IRES activity, highlighting the efficiency of cap-independent translation for the control Sirius-M$_2$i3(417n)-EGFP and the six mutants. The insert shows the difference in the green fluorescence between two of the mutants. Significance values reported in the graphs were determined by a one-tailed Student t-test, p-values: ** $0.001<p<0.01$. **B,** Expression of the construct M$_2$-Hairpin-EGFP in HEK293 cells: (top) Confocal images of M$_2$-Hairpin-EGFP, Mitotracker Deep red staining and DIC (bottom). Scale bar 5 µm. **C**, **left** Mutations of the bicistronic Sirius-H-M$_2$i3(15n/30n)-EGFP plasmids in the region of the M$_2$ i3 loop from nucleotides 1972 to 2001, in yellow are the modified nucleotides. In Sirius-H-M$_2$i3(15n/30n)mut#6-EGFP the four nucleotides in green (AAGA) were reinserted in the stretch of 20 T of Sirius-H-M$_2$i3(15n/30n)mut#5-EGFP. **right** IRES activity, highlighting the efficiency of cap-independent translation for the control Sirius-H-M$_2$i3(15n/30n)-EGFP and the six mutants illustrated. Significance ($p < 0.01$) was calculated in **A** against Sirius-M$_2$i3(417n)-EGFP and in **B** against Sirius-H-M$_2$i3(15n/30n)-EGFP. Error bars are s.e.m. Significance values reported in the graphs were determined by a one-tailed Student t-test, p-values: ** $0.001<p<0.01$.

~~When the full i3loop was placed after the hairpin, green fluorescence was fully recovered and the resulting construct (Sirius-H-M$_2$i3(417n)-EGFP) showed only a slight reduction of the EGFP/Sirius with respect to the value measured for the analogous reporter without hairpin (~90%) (**Fig. 2A**).~~ Only~~Finally~~, when the last portion of the M$_2$ i3 loop was inserted in the construct after the h~~H~~airpin, leading to Sirius-H-M$_2$i3(15n/30n)-EGFP, ~110% of the value measured for the Sirius-M$_2$i3(417n)-EGFP plasmid was recovered (**Fig. 2A**). Notably, when the hairpin was inserted within the i3 loop of the full length M$_2$-EGFP, upstream of the M368, it was still possible to observe green fluorescence in transfected cells (**Fig. 2B**).

These results confirm that the IRES is located in a region of the i3 loop sequence immediately upstream of the third methionine (M368). This is also confirmed from ribosome profiling data from human left ventricle tissue, displaying increased p-sites as well as overall ribosomal coverage in correspondence of this region (**Fig. S4B**). Based on this evidence, we mutated the conserved residues highlighted in yellow in **Fig. S4C,** in order to check for their relevance in recruiting the ribosome on the M$_2$ i3 loop sequence, as shown in **Fig. 2C**. As discussed in detail in the **Supplementary Materials**, we found that the four nucleotides AAGA1090-1093 next to the

methionine 368 play a pivotal role in recruiting the ribosome on the $M_2$ mRNA and in translating the $M_2$ C-terminal fragment.

**Generalization to other members of the GPCR superfamily**

In order to determine if the observed behavior is specific to the $M_2$ receptor or could highlight a feature common to other members of the GPCR superfamily, we repeated several of our experiments in another GPCR, the related muscarinic $M_3$ receptor. The $M_3$ displays an in-frame methionine at the end of the i3loop, in position 481. We then inserted a stop codon in position 273, generating the construct $M_3$stop273 that still displayed radio-ligand binding and functional activity (**Table S2**). Several other mutants, mimicking the single and double stop mutations ~~performed~~made for the $M_2$ displayed a behavior consistent with M481 being the start point of a cap-independent translation of the C-terminal also for the $M_3$ receptor (**Table S2**), thus indicating that IRES-mediated cap-independent translation of the C-terminal domain may not ~~could~~ be an exclusive phenomenon ~~not restricted~~ confined solely to the $M_2$ receptor, but may be a shared characteristic among ~~at least by~~ some other members of the GPCR superfamily. The presence of an IRES sequence upstream of M481 further appears to be supported by the observation of GFP fluorescence when 558 nucleotides from the i3 loop of the $M_3$ receptor (from nucleotide 880 to 1437) were inserted into the identical ~~same~~ Sirius-EGFP reporter construct ~~as~~ used for the $M_2$ receptor (**Fig. S5B**). ~~This appears to be indeed supported by the sequence-alignment of the five muscarinic receptors.~~Furthermore, ~~The~~the sequence alignment of the last portion of the $M_2$ i3 loop (from nucleotide 1072 to 1104) with regions adjacent to the second ($M_1$), fourth ($M_3$ and $M_4$) and first ($M_5$) in-frame Methionines, clearly shows that some nucleotide residues are conserved (**Fig. S4C**). Overall, these findings~~data~~ underline that cap-independent translation could be a more general feature of GPCRs mRNAs.

**Mitochondrial localization of the $M_2$ C-terminal fragment generated by cap-dependent expression under the control of a viral promoter**

~~After h~~Having established that the expression of the $M_2$ C-terminal domain is regulated by an IRES and that its translation starts with M368, we proceeded to investigate ~~went on to study~~ the role it might play in the cell. For this purpose, we fused $M_2$tail(368-466) with~~to~~ EGFP in pcDNA3, that is under the control of a strong CMV promoter, and we expressed it in HEK293 cells in order to study its subcellular localization. Confocal microscopy ~~demonstrated~~ revealed a remarkable ~~striking~~ mitochondrial localization of the $M_2$tail(368-466)-EGFP construct (**Fig. 3A**).

We confirmed this observation by co-staining HEK293 cells with a conventional mitochondrial stain and by co-expressing $M_2$tail(368-466)-EGFP with Tom20-mCherry, a marker for the outer mitochondrial membrane (**Fig. S6A**). The observed mitochondrial localization of $M_2$tail(368-466)-EGFP appeared in stark contrast to the canonical cellular localization of the $M_2$ receptor, as shown in ~~-~~**Fig. 3**~~b~~**B**, a confocal section at the level of the basal membrane of ~~transfected cel~~a cell co-transfected with ~~I with asa where~~ the $M_2$ muscarinic receptor ~~was~~ C-terminally labeled with the red fluorescent protein mRuby2. As expected, and in agreement with what is observed for several other GPCRs[12], the $M_2$ muscarinic receptor expression is homogeneous at the basal membrane, as opposed to the localized signal of the subplasmalemmal mitochondria.

In line with the radioligand binding data shown in **Table 1**, very little expression of $M_2$tail(368-466)-EGFP was observed on the plasma membrane, as we confirmed by fluorescence correlation spectroscopy (FCS) [13] (**Figs S6B-D**). In~~By~~ contrast, when the $M_2$tail(368-466)-EGFP was co-expressed with $M_2$stop228~~,~~ an increased localization could be seen on the cell membrane, suggesting a chaperon activity of the $M_2$ N-terminal domain ($M_2$trunk) (**Figs S6E-G**) and providing an explanation for the data obtained ~~above~~ in radioligand binding studies and reported in **Table 1**. Likewise, constructs such as $M_2$tail(368-466)-mRuby2 (a red monomeric fluorescent protein) and $M_2$tail(368-466)-tdTomato (a tandem-dimer red fluorescent protein) also displayed clear mitochondrial localization (**Figs S6H-I**), ruling out bias due to the fluorophore used. We obtained comparable findings also in COS-7 cells (**Fig. S6J**).

Mitochondrial import of the C-terminal fragment of the $M_2$ receptor was further verified by immunoblotting of protein extracts from purified mitochondria of HEK293 cells transfected with myc-tagged $M_2$ receptor, $M_2$tail(368-466) and $M_2$M368A, a mutant where the key methionine for the cap-independent translation of the C-terminus is changed to an Alanine. **Fig. 3C** shows that in purified mitochondria (unlike in the cytosolic and microsomal supernatants originating from the purification process, **Fig. S6K**), only the protein bands associated to the $M_2$ C-terminal fragment were visible. Notably, no mitochondrial import was visible for the $M_2$M368A, confirming that the third in-frame methionine (M368) in the i3 loop of the $M_2$ receptor is necessary for the C-terminal fragment production and its localization to the mitochondria. The slight offset between the lower bands in the $M_2$ wt and $M_2$tail(368-466) lanes matches exactly the different length of the linker used to fuse the myc-tag in the two constructs.

**Formatted:** Font: Bold, Highlight

**Formatted:** Not Highlight

**Formatted:** Font: Not Bold, Not Highlight

**Formatted:** Font: Bold, Highlight

**Formatted:** Highlight

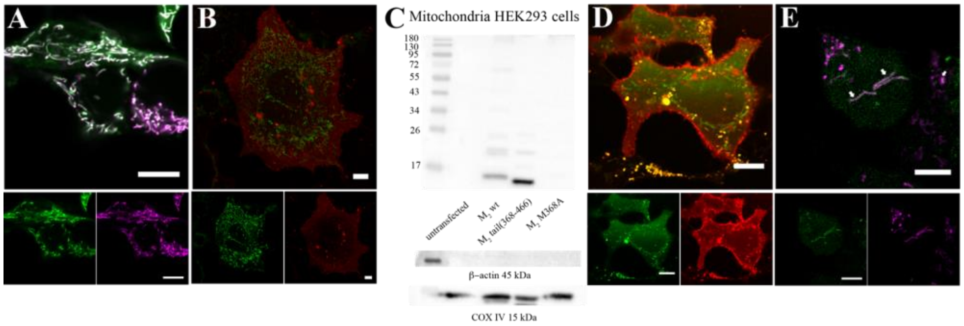


**Fig. 3. $M_2$tail(368-466) predominantly localizes to the cell mitochondria**

**A**, Colocalization of $M_2$tail(368-466)-EGFP (green) together with the mitochondrial targeted dye Mitotracker (magenta) in HEK293 cells. Separate spectral channels are displayed below. **B**, Co-expression of $M_2$tail(368-466)-EGFP (green) and wild type $M_2$-mRuby2 (red) in HEK293 cells. Separate spectral channels are displayed below. **C**, Western blot (10% TRIS-Glycine PAA gel) of mitochondria purified from Hek293 cells transfected with the $M_2$-myc, $M_2$tail(368-466)-myc, $M_2$M368A-myc, together with an untransfected control are blotted using an anti-myc antibody. Loading control was verified by immunoblotting COXIV and β-actin. **D**, Cellular localization of the construct $M_2$-mRuby2-STOP-$M_2$-i3-tail-EGFP in HEK293 cells. Expression of the $M_2$tail(368-466)-EGFP (green) after the STOP codon is an indicator of the endogenous IRES activity. While $M_2$-mRuby2 localizes correctly and predominantly to the cell plasma membrane, $M_2$tail(368-466)-EGFP localizes intracellularly. Separate spectral channels are displayed below. **E**, $M_2$-fr.sh.-EGFP (green) expressed in conjunction with Mitotracker (magenta). The EGFP is expressed despite the creation of an in-frame stop after M368, confirming endogenous cap-independent translation of the $M_2$tail(368-466). Arrows indicate areas of colocalization with the mitochondria. Separate spectral channels are displayed below.

## Localization of the $M_2$ C-terminal fragment generated by cap-independent expression under the control of the i3loop IRES

We then sought to express the $M_2$tail under the control of the i3loop IRES rather than a viral promoter, to further exclude the possibility that the observed mitochondrial localization of the $M_2$ carboxyl terminal fragment were due to overexpression of the construct. HEK293 cells were transfected with the construct $M_2$-mRuby2-STOP-$M_2$-i3-tail-EGFP which contains the full wild type $M_2$ receptor, labeled at the C-terminal with mRuby2, additionally fused to the C-terminal part of the $M_2$ receptor starting at nucleotide 685 of the i3 loop, following the stop codon of mRuby2, and ending with a C-terminal EGFP sequence (Fig. S3). Through this experimental strategy, the expression of $M_2$tail(368-466)-EGFP is regulated by the IRES, and C-terminal fragment

production is therefore close to physiological expression. The expression of EGFP confirmed that the *in vivo* production of the $M_2$tail(368-466) is regulated by the IRES within the i3 loop. In addition, the processed receptor fragment was eventually observed to be predominantly confined to the cytosol and the mitochondrial network (**Fig. 3D**).

Notably, when the $M_2$-EGFP fusion protein was expressed and the cells were co-stained with Mitotracker, a faint EGFP signal was also observed in the mitochondrial regions (**Fig. S7A**), although the expression of the full-length receptor masks this effect and makes quantification difficult. On the other hand, **Fig. 3E** shows that, when the $M_2$fr.sh-EGFP mutant was expressed in HEK293 cells, the IRES-generated C-terminal fragment of the $M_2$ receptor localized to the mitochondria. As mentioned above, in this construct the masking effect of the EGFP fused to the full-length receptor is ~~removed~~eliminated (see also **Fig. S3**). Similar mitochondrial localization was also observed when we transfected HEK293 cells with $M_2$fr.sh-mRuby2 (**Fig. S7B**), ruling out possible effects due to the specific fluorophore used as a tag. Again, ~~Similar~~comparable localization patterns were observed when the constructs were expressed in a different cell type, such as the H9c2 rat cardiomyoblast cell lines (**Fig. S7C**).

**Mechanism of $M_2$tail import into the mitochondria and localization within the organelle**

To gain further insight into the dynamics and mechanisms of the $M_2$ C-terminal fragment import within the mitochondria, we first monitored the kinetics of colocalization between mitochondria and EGFP in cells transfected with $M_2$tail(368-466)-EGFP. In cells observed for several hours, starting at 5h after transfection (**Fig. 4A**), the construct was found initially expressed in the perinuclear region, likely the endoplasmic reticulum, indicating that at least the initial step of subcellular trafficking of the $M_2$tail(368-466)-EGFP is the same as for the full length receptor [14]. At this time (5h post-transfection), almost no colocalization with the Mitotracker-stained mitochondria was visible. However, at 6h post-transfection, the colocalization level increased significantly, and $M_2$tail(368-466)-EGFP was either found in the cytosol or in the mitochondria. At 9h post-transfection the colocalization at the mitochondria was well visible (see **Materials and Methods**) and we could observe that almost half of the mitochondrial import occurred within 6h post-transfection (**Fig. 4B**).

This value is qualitatively in agreement with what we observed using an in-vitro assay to determine the import of the $M_2$ carboxyl terminal into isolated mitochondria (see **Materials and Methods**). To this end, we used mitochondria form *S. cerevisiae*, which is a robust and well-studied system for protein import assays. **Fig. 4C** shows the autoradiography experiment of isolated, intact, mitochondria incubated with 35S-labelled full length muscarinic $M_2$ receptor or $M_2$tail(368-466),

made using a reticulocyte in vitro translation system. The data show that the full-length receptor was not imported into mitochondria. On the other hand, the $M_2$tail(368-466) displays a clear mitochondrial import already after 5 minutes and increasing over time, up to 30 minutes. Interestingly, this import appears to be independent upon the transporters of the outer membrane Tom5, Tom6, Tom7 and Tom70 (**Fig. S7D**), whereas the import of an inner membrane protein, the ADP/ATP carrier (AAC), used here as a control, depends on Tom70. Upon extraction with carbonate most of $M_2$tail(368-466) was found in the pellet, and therefore presumably integrated in mitochondrial membranes. Furthermore *Blue native* analysis suggests that the mitochondrially imported $M_2$tail(368-466) is not a monomer but rather it is part of a larger complex of approximately 180 kDa (**Fig. S7D**).

All together these data indicate that the import of the $M_2$tail(368-466) leads to its localization in one of the mitochondrial membranes, to form a complex with other native mitochondrial proteins.

To further discriminate if the construct is on the outer or inner mitochondrial membrane, we set up a fluorescence assay based upon GFP fluorescence complementation [15] . HEK293 cells were first transfected with two constructs: $M_2$tail(368-466)-GFP11, in which a short 16 amino acids peptide of GFP was fused at the C-terminus of $M_2$tail(368-466), and mito-GFP1-10, in which the COX8A was N-terminally fused to the other GFP fragment containing the remaining 10 beta strands (See **Materials and Methods**). **Figure S8A** shows the successful recombination of the two proteins within the mitochondrial matrix. This observation is supported by a set of controls that include a mitochondrial intermembrane space-targeted protein achieved using the targeting sequence of SMAC [16]:(**Figure S8B-D** show a set of positive recombination controls using respectively SMAC-GFP11+SMAC-GFP1-10, mito-GFP11+mito-GFP1-10 and $M_2$tail(368-466)-GFP11+ $M_2$tail(368-466)-GFP1-10, all displaying mitochondrial recombination comparable to what is o~~v~~bserved in **Figure S8A** for $M_2$tail(368-466)-GFP11+mito-GFP1-10. As negative controls we observed the lack of complementation of $M_2$tail-GFP11 with SMAC-GFP1-10 (**Figure S8E**), and the absence of any significant signal when mito1-10 was expressed alone (**Figure S8F**).

We then applied this approach to the full length $M_2$ receptor, by generating the $M_2$-GFP11 construct and by transfecting it in HEK293 cells. **Fig. 4D** shows a green signal from the recombined GFP visible in the mitochondria. All together these data suggest that the $M_2$ C-terminal fragment is localized in the inner mitochondrial membrane, with its own C-terminal domain facing the matrix. This interpretation is also finally confirmed by Immuno Electron Microscopy (IEM) imaging of thin sections from cells expressing the $M_2$stop228 C-terminally labeled using a myc-tag after immune-gold labelling (**Fig. 4E**). As shown in **Figs 4F-G**, gold particles were observed

**Formatted:** Font: Bold, Highlight

only in transfected cells (Figure S7E), and exclusively in mitochondria, and at this stage they appeared localized either to the mitochondrial matrix or to the lumen of the cristae (**Figs 4H-K**).

Formatted: Font: Bold, Highlight

### Cell stress upregulates IRES-mediated $M_2$tail expression and drives mitochondrial localization

Having established that $M_2$tail localizes in the inner mitochondrial membrane we then moved to look at the effect of stress on the level of expression of the C-terminal fragment. Stressed cells are known to attenuate their translational activity via phosphorylation of the initiation factor eIF2α. However, certain mRNAs may be insensitive to eIF2α phosphorylation level if their translation is regulated by IRES, and under these conditions their expression is bound to increase rather than to attenuate [17, 18].

Therefore, we transfected COS-7 cells with the Sirius-$M_2$i3(417n)-EGFP reporter construct and stressed by starvation for three hours by replacing the culture medium with HBSS or phosphate saline buffer (PBS). Notably, PBS has a poorer nutrient content than the HBSS media, thus causing a more pronounced starvation. During this time interval, the green fluorescence was seen to gradually increase up to 1.4 times the basal level observed in HBSS-starved cells and 3.5 times for PBS-starved cells (**Fig. 5A**), confirming that the C-terminal fragment expression is upregulated in response to stress conditions such as cell starvation. The same experiment was reproduced in single cells, displaying an increase of the average EGFP/Sirius ratio (slope), and thus of IRES-mediated translation, upon HBSS starvation in HEK293 cells (**Fig. 5B**). We further used the GFP fluorescence complementation assay to emphasize the importance of stress in the pattern of expression of the $M_2$ gene. HEK293 cells were co-transfected with $M_2$-GFP11 and Mito-GFP1-10. HEK293 cells grown in culture medium predominantly expressed the full length $M_2$ receptor protein (**Fig. 5C**).

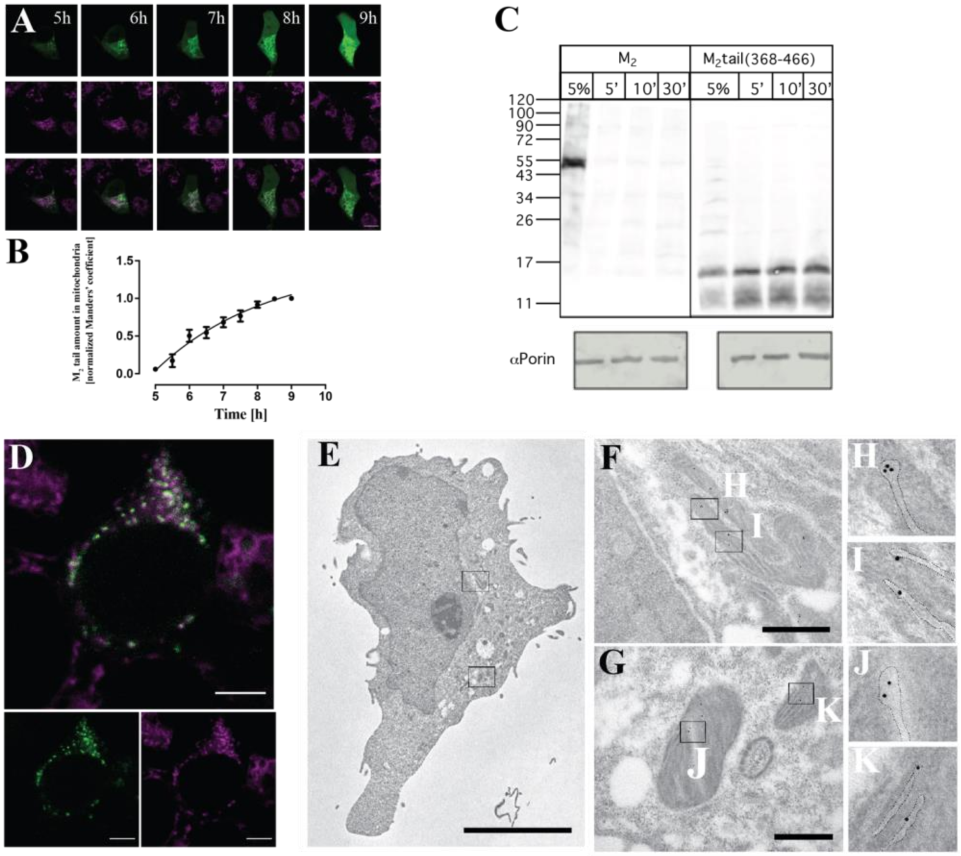


**Fig. 4. Mitochondrial import and organelle localization of $M_2$tail**

**A,** Time course of $M_2$tail(368-466)-EGFP import in the mitochondria of a HEK293 cell over a 4h interval, starting 5h after cell transfection, as indicated. Panels, from top to bottom show $M_2$tail(368-466)-EGFP (green), Mitotracker (magenta) and overlay displaying increasing colocalization over time. **B**, Quantification of the colocalization of the $M_2$tail(368-466)-EGFP in the mitochondria by calculating the Manders Coefficients (n=7 cells, from 3 independent transfections, error bars are s.e.m.). **C**, Import of protein $M_2$tail(368-466) in isolated yeast mitochondria: $^{35}$S-labelled and translated in-vitro full length muscarinic $M_2$ receptor or $M_2$tail(368-466) expressing 5 C-terminal Methionines were with isolated yeast mitochondria for 5, 10 and 30 minutes in the presence of the inner membrane potential The 5% lane represents a control with the pure protein, with no mitochondria. $\alpha$Porin is used as a loading control.. **D,** Detail of a HEK293 cell expressing the $M_2$-GFP11 construct together with mito-GFP1-10. The $M_2$-GFP11 was sequentially

transfected 24 h after the mito-GFP1-10 Magenta indicates mitotracker. White signal in the overlay indicates $M_2$tail(368-466) trafficking to the organelle. All confocal micrographs were obtained by sequential imaging using 488 nm excitation and detection in the 520-600 nm (EGFP), and at 633 nm and detected in the 650-700 nm range (Mitotracker) using HyD detectors in Photon Counting Mode. Scale bars are 10 µm throughout. **E,** Representative Immuno Electron Microscopy (IEM) micrograph of a COS-7 cell showing a large nucleus, several mitochondria and vacuoles nearby. Bar: 5 µm. **F-G**, High magnification of portion of the cell cytoplasm showing a few gold particles confined to the mitochondria. Bar: 500 nm. **H-K,** Gold particles appear associated with the mitochondrial cristae, highlighted by the dotted line. A total number of 236 gold particles in 40 mitochondria from 19 sections were detected. Controls reported in **Fig. S7E**.

**Formatted:** Font: Bold, Highlight

The green fluorescence detected at the plasma membrane is explained with the chaperoning effect of $M_2$-GFP11 on Mito-GFP1-10 before its sorting to the mitochondria. In sharp contrast, when HEK293 cells were first transfected with Mito-GFP1-10 and, 24h later, transfected with $M_2$-GFP11 followed by a 6 h starvation in HBSS, the pattern of expression changed drastically: IRES mediated translation of the $M_2$tail(368-466) fragment led to a predominantly mitochondrial green fluorescence signal of the complemented GFP (**Fig. 5D**). These results are in line with our observation that the IRES mediated C-terminal fragment localization to the mitochondria in cells expressing the construct $M_2$-mRuby2-STOP-$M_2$-i3-tail-EGFP was enhanced upon cell starvation, and drastically reduced upon mutation of the M368 (**Figure S9** and **Supplementary Materials**).

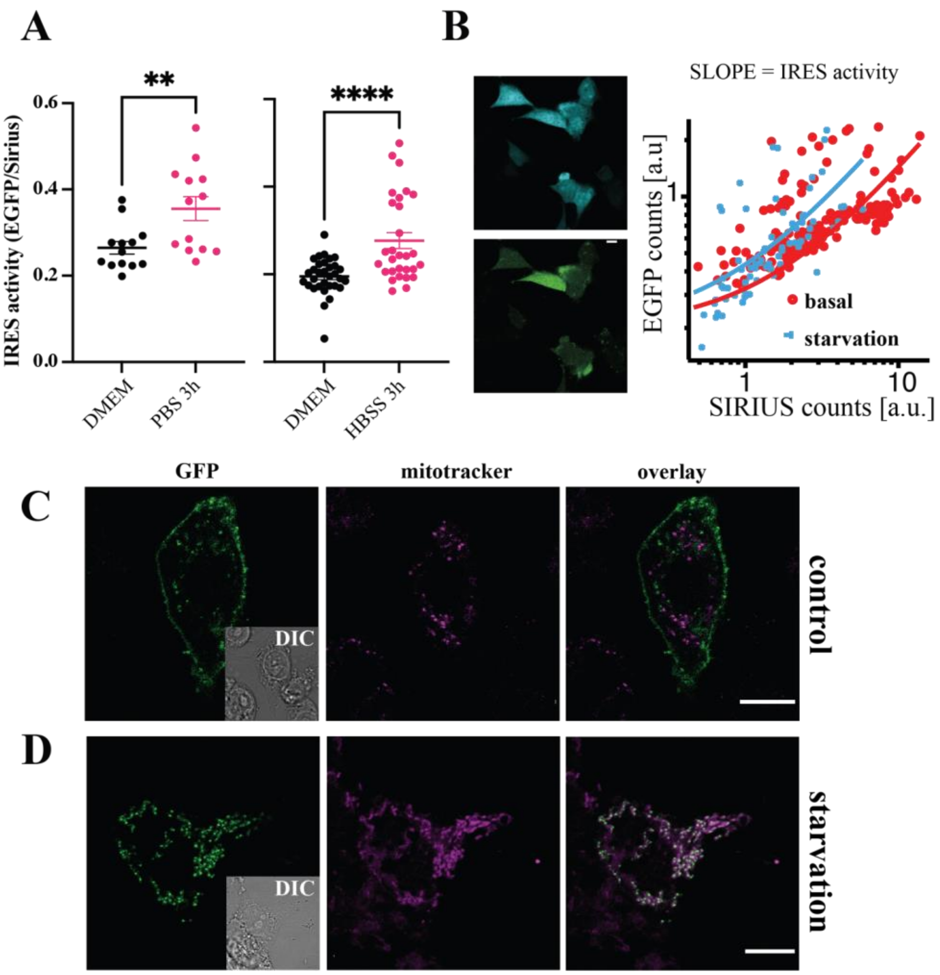


**Fig. 5 Effect of integrated stress response on IRES activity and $M_2$tail(368-466) localization**

**A**, 96-well plate reader measurement of the increase of EGFP fluorescence relative to Sirius in COS-7 cells transfected with the bicistronic Sirius-$M_2$i3(417n)-EGFP plasmid and starved for three hours respectively in PBS (left panel) (n= 3 transfections, 16 wells) and HBSS (right panel) (n=1 transfection, 16 wells). Significance values reported in the graphs were determined by a one-tailed Student t-test, p-values: **** $p<0.0001$; ** $0.001<p<0.01$. **B,** Single cell measurement of EGFP vs SIRIUS expression in HEK293 cells, in basal conditions and upon starvation. Lines are linear fits (note log-log scale). **C**, HEK293 cells were sequentially transfected with pMito-GFP1-10 (24h) and $M_2$-GFP11 (8h, of which 6h under starvation

conditions) and then grown in full culture medium or **D,** starved for 6 h after transfection of $M_2$-GFP11.Shown are EGFP fluorescence (left, with DIC inset), Mitotracker (center) and overlay (right). Scale bars are 10 µm.

**Functional role of the C-terminal fragment in the mitochondria**

The next step of our investigation concerned the obvious question about the functional effects that the observed localization of the $M_2$ C-terminal fragment might have on the mitochondrial activity, and -more generally- on the energy metabolism of the cell. To investigate this aspect, we measured the extent of oxygen consumption in COS-7 cells expressing $M_2$-EGFP, $M_2$tail(368-466)-EGFP or the sole EGFP as a control. Initially, we normalized the expression of the three proteins to the amount of EGFP expressed in the cells, then the metabolic functions were assayed on a *Seahorse* instrument using cells transfected with normalized amounts of plasmids (**Fig. 6A**). Our data did not show a significant difference in total oxygen consumption capacity between cells transfected with cytosolic EGFP and the untransfected control. However, a significant decrease of oxygen consumption was found in cells transfected with $M_2$-EGFP and even more so with $M_2$tail(368-466)-EGFP (**Fig. 6A**). Notably, the basal oxygen consumption rate (OCR) in starvation-induced stress conditions was profoundly decreased in $M_2$tail(368-466) cells. This occurred to a smaller extent also in $M_2$ cells compared to EGFP-transfected and empty control cells (see also the time-course in **Fig. S10A)**. These results are in line with what was observed using a Clarke-type oxygen electrode (**Supplementary Materials and Fig S10B-C**).

Notably, there was no difference in the number of apoptotic cells among the different cell groups as shown by the caspase-3 activation assays of apoptosis [19](**Fig. S10D**) and morphological investigation of the mitochondrial network (**Fig. S10E**). To test whether the $M_2$tail effects on mitochondrial oxygen consumption could alter the production of Reactive Oxygen Species (ROS), the measurements were combined in a single cell assay. Remarkably, ROS production (assayed using a fluorescence readout kit, where the fluorochrome becomes activated in contact with oxidized species) was clearly reduced within and in proximity of mitochondria of cells expressing a high level of the $M_2$tail(368-466) (**Fig. 6Binset**). By regrouping individual mitochondria in classes according to the level of the ROS marker expression, an inverse correlation between the amount of receptor fragment (Green Fluorescence) and the production of ROS (Red Fluorescence) was revealed (**Fig. 6B)**.

Cell growth was also found to be affected depending on which of the constructs was transfected. Notably, transfection of HEK293 cells with full length $M_2$ receptor reduced significantly cell growth compared to the control, whereas cells transfected with the mutant $M_2$M368A, that do not express the $M_2$ C-terminal fragment (**Fig. 6C**), displayed a significantly higher growth. All cells were

incubated with saturating concentration of the $M_2$ antagonist atropine throughout, in order to exclude effects on cell growth and proliferation due to the canonical receptor signaling.

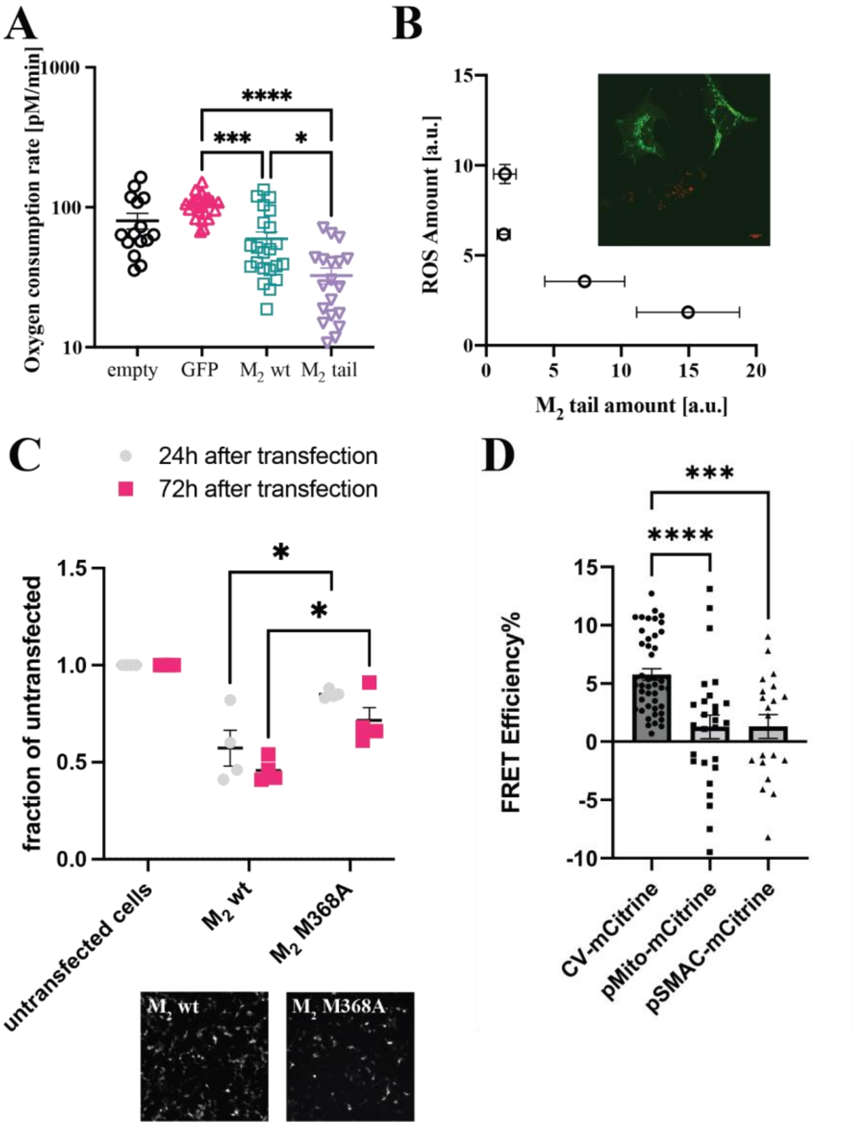

**Fig. 6. Effects of $M_2$tail expression, or absence thereof, on mitochondrial function and cell metabolism in transfected HEK293 cells.**

**A**, Basal oxygen consumption upon HEK293 cell stress -following starvation- measured by a Seahorse assay at a constant temperature of 37 °C. Significance values reported in the graphs were determined by a one-tailed Student t-test, p-values: **** $p<0.0001$; *** $0.0001<p<0.001$;* $0.01<p<0.05$. **B**, Localization of $M_2$tail(368-466)-EGFP in HEK293 (green) cells stained with a marker for reactive oxygen species (Cell Rox Deep Red). Relationship between green ($M_2$tail abundance) vs red (amount of ROS produced) fluorescence intensities measured from regions of the mitochondrial network. The data were binned in four groups as a function of the amplitude of Cell Rox Deep Red signal (0-2.5, 2.5-5. 5-7.5 and 7.5-10 intensity counts). **C**, Cell viability assay performed on HEK293 cells transfected with $M_2$ and $M_2$M368A, compared to an untransfected control. Cells were counted 24h and 72h after transfection in T-25 flasks, exposed throughout to 100 nM atropine. The experiments were performed in two biological replicates, each with two technical replicates. Transfection efficiency (to the right of the chart) was monitored thanks to the mRuby2 fluorescent reporter within a bicistronic cassette within each vector (see **Materials and Methods**). Significance values reported in the graphs were determined by a one-tailed Student t-test, p-values: ** $0.001<p<0.01$; * $0.01<p<0.05$. **D**, FRET efficiency plot of the interaction between $M_2$tail(368-466)-mTurquoise2 and $F_0F_1$ATP Synthetase (CV-mCitrine), COX8a(pMito)-mCitrine and SMAC-mCitrine, as determined by acceptor photobleaching measurement in a confocal microscope. Each dot represents a ROI within a cell. At least 3 cells from two separate transfections were imaged for each pair.

These functional data are further supported by Fluorescence Resonance Energy Transfer (FRET) data aimed at investigating the molecular interactions of the mTurquoise2 labeled $M_2$tail(368-466) with mitochondrial proteins. Based on the oxygen consumption data (**Fig. 6A** and **Fig. S10A**) we chose as primary target the $F_0F_1$ ATP Synthetase complex, also indicated as complex V (CV), and we labeled the γ subunit with mCitrine, a fluorescent acceptor. The γ subunit is located towards the mitochondrial matrix. We further monitored interactions with an intermembrane space protein (SMAC) and a protein localized to the mitochondrial matrix (COX8a), both fused to the mCitrine fluorescent acceptor. As clearly illustrated in **Fig. 6D,** FRET efficiency, an indicator of molecular interaction, is significantly larger for $M_2$tail(368-466)-CV pair than both the intermembrane space SMAC and the matrix COX8a. The latter result is particularly relevant, as it indicates a specific interaction of $M_2$tail(368-466) with CV.

## Relevance of C-terminal expression and mitochondrial translocation in a physiological, endogenous setting

The results provided in the previous sections convincingly demonstrate the cap-independent translation of the $M_2$ C-terminal fragment, its upregulation and mitochondrial localization upon stress response, and the subsequent effect in regulating oxidative phosphorylation by a direct interaction with complex CV of the respiratory chain. To test the physiological relevance of these findings we first prepared protein extracts from heart, brain and spleen tissues from a line of knock-in mice (see **Materials and Methods**), designed to have expression of a Chrm2/tdtomato fusion protein driven by the endogenous $M_2$ muscarinic acetylcholine receptor promoter/enhancer sequences. The heart and brain are known to express sizable levels of the $M_2$ receptor, whereas the spleen, where no expression of the $M_2$ receptor is reported in the proteomic databases, was used as a negative control.

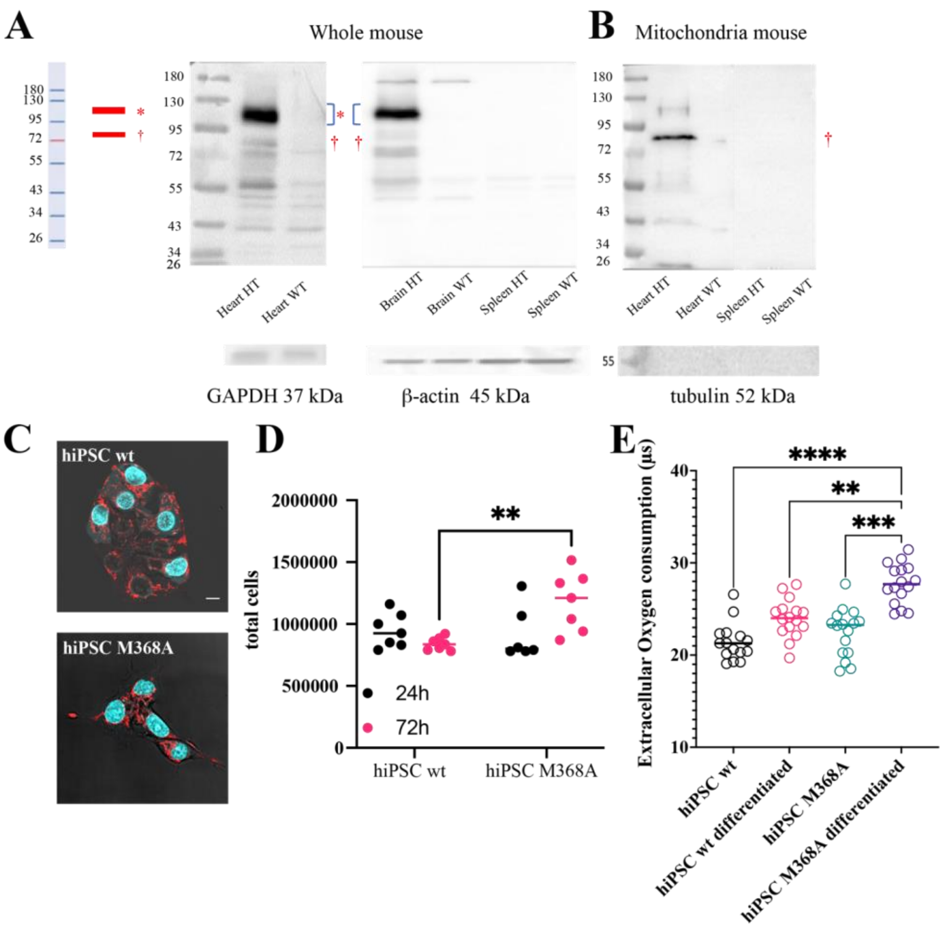


**Fig. 7. IRES-mediated $M_2$tail(368-466) expression occurs in endogenous, physiological settings and its suppression affects cell metabolism**

**A**, Western blot from tissue preparations originating from the indicated organs of $M_2$-tdtomato knock-in mice (HT) and wild-type animals (WT). Immunodetection was performed using an anti-tdtomato primary antibody. Loading control normalization was performed using GAPDH in the heart, and $\beta$-actin in the brain and spleen. **B**, Western blot (8% TRIS-Glycine PAA gel) of mitochondria purified from tissue preparations originating from the indicated organs of $M_2$-tdtomato knock-in mice (HT) and wild-type animals (WT), detected using an anti-tdtomato antibody. Mitochondrial preparation purity was assessed by blotting tubulin. **C,** Confocal micrographs of wt hiPSCs (left) and Crispr/Cas9 clone harboring the M368A mutation within the $M_2$ gene (right), stained with DAPI (cyan) and Mitotracker (red). **D**, Cell proliferation assay performed

for wt hiPSCs and M368A hiPSCs grown in T25 flasks., exposed to Atropine. Cells were counted 24h and 72h after seeding. Significance values reported in the graphs were determined by a one-tailed Student t-test, p-values: ** $0.001<p<0.01$. **E**, Extracellular oxygen consumption assay, performed on wt and M368A hiPSCs, prior to and after 7 days undirected differentiation under Atropine exposure. The lifetime of a commercial dye increases as oxygen in the medium is progressively consumed by the cells. The data displayed wascollected after 60 minutes incubation. Significance values reported in the graphs were determined by a one-tailed Student t-test, p-values: *** $0.0001<p<0.001$; ** $0.001<p<0.01$.

The use of a transgene tag was necessary, since antibody against the $M_2$ receptor are notoriously ineffective when targeted at the C-terminal portion of the receptor. Instead, the tdtomato fluorescent protein can be targeted by a specific polyclonal antibody, as shown by the Western blot displayed in **Fig. 7A**. As expected, the full-length receptor-tdtomato fusion was observed at ~130 kDa (~75 kDa contributed by the receptor and ~55 kDa contributed by tdtomato). Notably we observed again (both in the heart and brain, but not in the spleen) a specific band doublet, this time at ~75 kDa, which corresponds to the sum of the molecular weights of the tail fragment added to the molecular weight of tdtomato. When mitochondria were purified from the heart and spleen of the Chrm2/tdtomato mice **Fig. 7B** the band at ~75 kDa was substantially enriched, matching the expected molecular weight of the $M_2$tail. These data strongly suggest that the $M_2$tail is expressed endogenously, and it is sorted to the mitochondria.

We finally posedasked the question of what the effect of would happen be abolishing the translocation of the $M_2$ C-terminal fragment to the mitochondria, in a physiological and entirely label-free setting. Notably, human induced pluripotent stem cells (hiPSC) combined with genome editing approach are state-of-the art tools to investigate the effect of gene mutations in physiological and label-free setting. Therefore, we incorporated by Crispr/Cas9 the homozygous M368A mutation in hiPSCs (**Materials and Methods**). Based on our data of (**Fig. 3C**), this mutation should be sufficient to significantly impair the $M_2$tail production and mitochondrial translocation. Wild type (wt) and M368A hiPSCs were cultured in pluripotency maintaining medium, and subsequently undirectedly differentiated upon exposure for seven days to DMEM-F12 medium, as described in the **Materials and Methods**. **Fig. 7C** shows confocal micrographs for wild type and M368A hiPSCs cultured in E8 medium respectively, with the M368A hiPSCs displaying a mildly more spread-out phenotype. We shall note here that the $M_2$ receptor has been found to be expressed in PSCs lines, and it has been shown to play a role in stem cell differentiation [20, 21]. In this context, however, we were not interested in the canonical signaling of the receptor, and all experiments were performed with a saturating concentration of atropine,

to block any possible effect of the canonical signaling of the $M_2$ receptor. We then evaluated cell proliferation, observing a significant increase in the proliferation of the M368A clone 72h after seeding **Fig. 7D**. The observation that cells harboring the mutation that suppresses $M_2$tail display an increased proliferation suggests that the same effect is at play here, as observed in the overexpression system (**Fig. 6C**). These results are supported by extracellular oxygen consumption assays performed, for both hiPSCs and differentiated hiPSCs (**Fig. 7E**): the M368A cells indeed display an accelerated oxygen consumption rate, and this effect increases as the cells progressively differentiate.

For comparison, the assay was also carried on HEK293 overexpressing the wild type $M_2$ receptor and the mutant $M_2$M368A (**Fig. S10F**). We shall further note here that signaling properties of $M_2$ M368A with respect to the wild type $M_2$ receptor are comparable, as displayed by pERK immunoblotting as well as FRET $G_i$ activation assays performed in cells overexpressing either the wild type $M_2$ or the $M_2$ M368A (**Fig. S10 G,H**).

## Discussion

Over the last 10 years, growing evidence has accumulated indicating that GPCRs can be functional and signal also at cellular locations other than the plasma membrane. These locations can be associated to their canonical trafficking pathways (such as sorting vesicles and endosomes) [22], but also to non-canonical locations, such as the nucleus, the nuclear membrane, the Golgi apparatus and the mitochondria (reviewed in Jong et al. BPJ 2018 [23]). Some of these locations are surprising, since many ligands are non-membrane permeable, and it is still debated how they can reach their target receptors at such intracellular locations.

Interestingly, the specific localization of these receptors appears to go hand in hand with a specific function. In these cases, activation/inhibition of mitochondrially localized GPCRs have been shown to specifically affect the organelle function, ranging from oxygen consumption rates to $Ca^{2+}$ handling and even to apoptosis. Notably Benárd et al. [24] showed that the $CB_1R$ on the outer mitochondrial membranes affects neuronal energy metabolism; Suofu et al. [25] demonstrated that activation of the $MT_1R$ by a mitochondrial melatonin pool can affect cytochrome C release. This is particularly interesting, since cytosolic cAMP does not appear to be able to activate mitochondrial PKA, and hence a mitochondrially localized GPCR signalosome appears to be required to enable such downstream signaling cascade [26].

However, other non-canonical mechanisms are also possible for the regulation of mitochondrial function. It is well known that GPCRs are subject to interindividual variability, arising, for example, from mRNA alternative splicing (i.e. the process which allows the recombination of exons) which leads to different isoforms which may present distinct signaling and localization properties[27]. Receptor variants with different functional and localization properties may also occur due to other processes. For example, our comprehension of eukaryotic translational regulation is facing a rapid expansion, as exemplified by the observation that small open reading frames (smORFs), also within long non-coding RNAs (lncRNAs), are an important class of genes, oftentimes with mitochondrial localization [28].

In this study, for the first time, we provide evidence that an IRES sequence drives expression of a membrane receptor fragment and acts as a regulator of its expression levels and function. IRES sequences are distinct regions of the mRNAs that are capable of recruiting ribosomes in the proximity of an internal initiation codon and translate transcripts in a cap-independent manner[8, 9, 28-30]. While in eukaryotes the most common mechanism of translation initiation is the cap-dependent process, which requires the interaction of several key proteins at the 5'-end (5′ cap) of mRNA molecules, recent years have seen growing evidence that IRES sequences, which are common features of virus replication [8], can mediate cap independent translation of eukaryotic mRNA(s) [9].
~~Here, we show that an~~The IRES sequence is located within the third intracellular loop of the $M_2$ muscarinic receptor, where it plays a crucial role in regulating the expression of its carboxy-terminal domain, which we identified as $M_2$tail(368-466).

We assessed the IRES-driven expression of the C-terminal fragment through a broad set of constructs, meant to exclude alternative synthesis mechanisms such as stop-codon read-through, and a diversified palette of techniques. Radioligand binding data (**Table 1**) pointed to the necessity of the C-terminal fragment synthesis, to explain the observed downstream signaling in mutants carrying a stop codon in the initial portion of the i3loop of the $M_2$ receptor, upstream of M368. Fluorescence microscopy allowed us visualizing the production of the EGFP-tagged C-terminal fragment in these receptor mutants (**Fig. 1**).

Mutagenesis of key residues within the i3 loop of the $M_2$ receptor allowed us to pinpoint the 3$^{rd}$ in-frame methionine as the most likely candidate for the beginning of the cap-independent translation (**Fig. 2**). This observation appears to be supported by the observation of a peak in ribosome

coverage around the M368 seen in ribosome profiling databases (as reviewed in Ingolia et al. [31]), and particularly prominent in those from left ventricle cardiomyocytes, where $M_2$ receptor expression is known to be abundant [32].

To our surprise, in contrast to the plasma membrane localization of the wild type, full length $M_2$ receptor, the C-terminal fragment was localized mostly in mitochondria in HEK293 cells. This localization occurred markedly when overexpressing the C-terminal fragment $M_2$tail(368-466), but could be also clearly detected when the C-terminal fragment was expressed under the control of the IRES sequence of the full-length receptor (**Fig. 3**). These results were further validated in a rat cardiomyoblast cell line such as H9c2 (**Fig. S7C**).

We devised several strategies to detect the localization of the IRES-driven fragment in the mitochondria by avoiding the cell-wide background signal from the full-length receptor. **Fig. 3D,E** show two prominent examples, namely relying on spectrally unmixing the two components, or by sending out of frame the EGFP at the C-terminus of the full-length receptor. By studying mitochondria purified from HEK293 cells we could confirm that the mutation of the M368, which we identified as the starting point of the IRES-mediated translation, is sufficient to suppress any mitochondrial targeting of the $M_2$ C-terminal fragment, and most likely, its synthesis altogether (**Fig. 3C**).

We then addressed the mechanism of mitochondrial import of the C-terminal fragment and its localization within the organelle (**Fig.4**). Our data, originating both from live cell import kinetics as well as from in-vitro import into isolated mitochondria (**Fig.4A,B**), support the notion that the fragment becomes rapidly imported into mitochondria following translation in the cytosol. Once inside the mitochondria, it undergoes processing leading to the appearance of a lower molecular weight band, observed both in Western blots from whole cell and mice tissue protein extracts, as well as from purified mitochondria and in the yeast in vitro assays (**Fig. 4C**). The fragment is found predominantly in a carbonate-insoluble fraction, i.e. a membrane, and it appears to form a high molecular weight complex of ~180 kDa with other mitochondrial proteins (**Fig. S7D)**.
Fluorescence complementation assays (**Fig. 4D**) and electron microscopy (**Fig. 4E-H**) suggest that the $M_2$ C-terminal fragment predominantly resides in the inner mitochondrial membrane, with its own C-terminal domain exposed towards the matrix. Future work should clarify the precise import pathway.

Under integrated stress response, namely aminoacid starvation, IRES-mediated production of the C-terminal fragment was increased (**Fig. 5A-B**), with a marked increase in mitochondrial localization (**Fig. 5C)**. Most importantly, our data show that mitochondrial oxygen consumption was reduced by the $M_2$tail(368-466) (**Fig. 6A,B**), and cell proliferation was affected (**Fig. 6C**). On these bases, the $M_2$tail might be needed in mitochondria as a means to reduce mitochondrial basal oxygen consumption and ATP-linked respiration. An important consequence of reduced respiration is the lower level of ROS produced in mitochondria, as we observed in agreement with this hypothesis **Fig. 6B**.

This observation was confirmed in physiological conditions by Western blots in a knock-in mice carrying a tag at the C-terminus of the endogenous $M_2$ receptor. These could show mitochondrial enrichment of a band corresponding to the molecular weight of the C-terminal domain (**Fig. 7A).** Moreover**,** the physiological relevance of our observations was tested in an entirely label-free assay, by Crispr/Cas9 editing of pluripotent stem cells in order to suppress the synthesis of the $M_2$ C-terminal fragment. By mutating the IRES starting methionine M368, we observed an increase of cell proliferation of this clone compared to its wild type counterpart, together with an increase of oxygen consumption rates (**Fig. 7B-D**). Overall this corroborates our hypothesis that the $M_2$ C-terminal fragment reduces oxygen consumption upon reaching the mitochondria. The effect was further amplified when the cells were undirectedly differentiated, and it was consistent with the shift of the cell phenotype towards oxidative phosphorylation [33].

The mechanism by which the $M_2$tail(368-466) may reduce oxygen consumption in mitochondria and its meaning needs to be further explored. Our FRET data (**Fig. 6D**) indicate clearly that the $M_2$ C-terminal fragment has a specific interaction with the $F_0F_1$ ATP-synthase complex, supporting the concept that it inhibits the activity of this enzyme, thus leading to the observed reduction in oxygen consumption rates. Future work should clarify the precise import pathway and the inhibitory effect of $M_2$tail(368-466) on $F_0F_1$ ATP-synthase. Nonetheless, since ROS production is implicated in the pathogenesis of several diseases [33], we would propose the hypothesis that increased localization of the $M_2$ C-terminal fragment in mitochondria, as it occurs under integrated stress response, might have protective effects (**Fig. 8**).

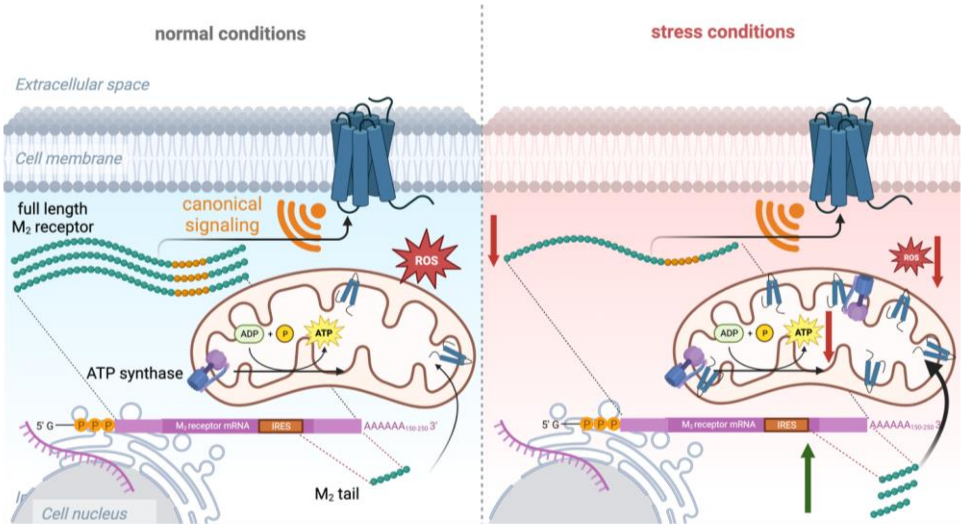


**Fig. 8. Schematic representation of the wild type $M_2$ receptor gene products, their destination in the cell and their function.**

The $M_2$ receptor gene gives rise to one transcript but two different translation outputs: when translation starts from the canonical cap site the wild type M2 receptor is generated (full length $M_2$ receptor). When translation is driven by the internal ribosome entry site (IRES) the C-terminal fragment is generated ($M_2$ tail). The two proteins are then sorted in two different cell compartments where they play completely different roles: the $M_2$ receptor signals canonically at the plasma membrane as a G protein-coupled receptor. The $M_2$tail is instead sorted to the mitochondria.

In normal conditions the activity of the IRES is low, and only a small amount of $M_2$tail is produced in the cell (left). However, upon cell stress conditions (right), $M_2$tail IRES-mediated translation is upregulated and $M_2$tail in the mitochondria interacts with the ATP synthase and reduces oxidative phosphorylation and ROS formation. Created with BioRender.

In broader terms, protection of mitochondria by unconventional import mechanisms to allow localization within the organelle of specific proteins has been previously observed for cells that undergo stress conditions[34] . The mechanism of IRES-dependent modulation of translation and consequent mitochondrial targeting of the translation product of a GPCR may provide another means, completely unrelated to the canonical signaling function of this class of membrane receptors, of a generic cellular response to overcome different types of stress by modulating the mitochondrial proteome and adjusting the mitochondrial bioenergetic state.

## Materials and Methods

### Generation of mutant muscarinic $M_2$ receptors for radioligand binding studies, Western blotting, immunohistochemistry and fluorescence microscopy

Multiple receptor mutants were constructed starting from the human $M_2$ muscarinic receptor expressed in the pcD vector [35]. The constructs are sketched in **Fig. S3** and their cloning is described in detail in the **Supplementary Methods**.

### Reverse transcription

Reverse transcription was performed using a 24-nucleotide antisense oligo (TTACCTTGTAGCGCCTATGTTCTT) directed against the last 24 nucleotides at the 3' end of the $M_2$ open reading frame. cDNA amplification was carried out with the same antisense oligo used for reverse transcription, together with a 24 nucleotides sense oligo (ATGAATAACTCAACAAACTCCTCT) directed against the first 24 nucleotides at the 5' end of the $M_2$ open reading frame.

### Cell culture and transfection

COS-7 (African green monkey kidney fibroblast-like SV40 transformed cell line) and CHO (Chinese Hamster Ovary) cells (from American Type Culture Collection) were grown at 5% $CO_2$ and 37 °C, in high glucose Dulbecco's modified Eagle's medium (DMEM) supplemented with 10% fetal bovine serum (FBS), 2 mM L-glutamine, 50 IU/ml penicillin, and 50 mg/ml streptomycin, and non-essential amino acids. HeLa cells (from American Type Culture Collection) were cultured in the same medium, but with the double amount of L-glutamine. SHSY-5Y cells were cultured in Roswell Park Memorial Institute (RPMI) medium supplemented with 10% FBS, 2 mM L-glutamine, 50 IU/ml penicillin, and 50 mg/ml streptomycin. Media and reagents used for cell cultures were from Euroclone, PAN Biotech, Biochrome and Sigma-Aldrich. Cell transfection was performed using FuGene® transfection reagents (Promega) as recommended by the manufacturer. Eighty percent confluent cells were washed twice with free-serum medium and the mix of FuGene and plasmid DNA was added. In every experimental condition 6-8 µg of plasmid DNA per Petri (10 $cm^2$) were used. Cells were grown for 48 h post-transfection before use. In co-transfection experiments, half amount of each plasmid DNA was used.

HEK293 (Human Embryonic Kidney, ECACC 96121229 from Sigma-Aldrich Chemie GmbH)  were cultured in DMEM (Dulbecco's modified Eagle's medium PAN biotech, Aidenbach, Germany), supplemented with 4.5 g/L Glucose, 2 mM L-Glutamine, 10% FCS, 100 units/mL penicillin and 0.1 mg/mL streptomycin and maintained at 37 °C and 5% $CO_2$. Cells cultured in 15-cm dishes were split at a 1:36 ratio into 6-well plates containing poly-D-lysine (PDL)-coated 24 mm glass coverslips. Cells were transfected using Effectene transfection reagent (QIAGEN) according to the manufacturer's instructions. Cells seeded on PDL-coated coverslips in 6-well plates were transfected 16 hours after seeding with 0.6 µg plasmid/well.

**Fluorescence microscopy**

Fluorescence microscopy experiments were performed either on a Leica SP5 (or SP8) Confocal Microscopes, using HyD photon counting detectors and an Argon/Ion laser (or white light laser) source for 488 nm/633 nm excitation and a solid-state diode laser (or white light laser) for 561 nm excitation. A solid-state diode laser was further used for 405 nm excitation. A 40x 1.3 NA objective was used, and the electronic zoom was set to achieve a pixel size of 50 nm. For visualization, mitochondria of transfected cells were labelled for 30 minutes at 37 C with 50nM MitoTracker® Deep Red FM (Invitrogen™, Thermo Scientific).

**Ligand binding studies**

Cells were lysed 48 h post-transfection with ice-cold 2 mM EDTA, 1 nM Na-HEPES (pH 7.4), scraped with a rubber policeman in the presence of ~4 ml rinsing medium, and homogenized with a Polytron homogenizer (ULTRA-TURRAX) in binding assay buffer (50 mM Tris-HCl, pH 7.4, 155 mM NaCl, 0.01 mg/ml bovine serum albumin). Radioligand bindings were carried out at 37 °C for 3 h in a final volume of 1 ml. Non-specific binding was determined in presence of 1 µM atropine. For competition binding assays, increasing concentrations of carbachol (from 0.01 µM up to 10 µM) were used in presence of 200 pM $[^3H]$N-Methylscopolamine ($[^3H]$NMS; 78.9 Ci/mmol; PerkinElmer).

The bound ligand was separated from the unbound ligand using glass-fiber filters (Whatmann, GF/B) with a Brandel Cell Harvester, and filters were counted with a scintillation β-counter. $K_D$ values of $[^3H]$NMS were calculated in direct saturation experiments, whereas carbachol Inibitory Concentration $(IC)_{50}$ values were calculated in competition curves, against 400 pM $[^3H]$NMS, fitted to one binding models using the iterative, non-linear least-squares regression analysis of OriginPro 7.5 software (OriginLab Corporation).

**Phosphatidylinositol Breakdown Assay**

Transfected COS-7 cells were incubated with myo-[$^3$H]inositol (23 Ci/mmol; PerkinElmer) for 48 h. Immediately before the experiment the cells were washed twice with phosphate-buffered saline and incubated for 15 min in Eagle's medium containing 10 mM LiCl and 20 mM HEPES. The medium was then replaced by 0.25 ml of the same medium containing the experimental agents. After a 1-hour incubation at 25 °C the reaction was arrested by the addition of 0.75 ml of 7.5% (w/v) ice-cold trichloroacetic acid followed by a 30-min incubation on ice. The trichloroacetic acid was extracted with water-saturated diethyl ether (3 × 4 ml), and levels of $IP_1$ determined by anion exchange chromatography.

**Adenyl Cyclase Assay**

COS-7 cells were transfected with plasmid(s) containing the receptor of interest plus adenyl cyclase V. 24 h after transfection the cells were detached with trypsin and re-cultured in 24-well plates. Following an additional period of 24 h culture, they were assayed for adenyl cyclase activity. The assay was performed in triplicate**.** In brief, cells were incubated in 24-well plates for 2 h with 0.25 ml/well of fresh growth medium containing 5 μCi/ml [$^3$H]adenine (PerkinElmer). The medium was then replaced with 0.5 ml/well of DMEM containing 20 mM HEPES, pH 7.4, 0.1 mg of bovine serum albumin, and the phosphodiesterase inhibitors 1-methyl-3-isobutylxanthine (0.5 mM) and Ro-20–1724 (0.5 mM). AC activity was stimulated by the addition of 1 μM forskolin in the presence or absence of carbachol. After 10 min of incubation at +30 °C the medium was removed, and the reaction terminated by addition of perchloric acid containing 0.1 mM unlabeled cAMP followed by neutralization with KOH. The amount of [$^3$H]cAMP formed under these conditions was determined by a two-step column separation procedure[3].

**Immunoelectron Microscopy (IEM)**

Samples were fixed for 3 hours at 4 °C with a mixture of 4% PFA and 0.05% GA in 0.1M PB, pH 7.4. After rinsing in the same buffer, samples were dehydrated in a graded series of ethanol and embedded in medium grade LR White resin, following addition of the LR White accelerator (London Resin Company, Berkshire, England). The resin was then polymerized for 20 min at room temperature in tightly capped gelatine capsules. Ultrathin sections were obtained using a Reichert Ultracut ultramicrotome equipped with a diamond knife and

collected on nickel grids. For immunogold labelling, 60-80 nm thick sections were processed following the incubation protocol originally developed by Aurion (Wageningen, the Netherlands). Unspecific binding was prevented by treating sections for 20 min with 0.05 M glycine in PBS, at pH 7.4. A subsequent block step was made by washing sections for 30 min in 5% BSA, 5% normal goat serum and 0.1% cold water fish skin gelatine. Sections were soaked overnight 4 °C in incubation buffer (PBS and 0.1% BSA-cTM, pH 7.4) (3x5 min) in a moist chamber containing the Myc-Tag (9B11) mouse antibody (Cell Signaling Technology) diluted 1:50 with incubation buffer. Sections were then washed in incubation buffer (6x5 min) and incubated for 1 hour with a secondary goat anti-mouse antibody conjugated to 10 nm gold particles (EM Grade 10nm Goat anti-Mouse IgG Conjugate EM.GAM10/1, British BioCell International, UK), diluted 1:10 in incubation buffer. After rinsing in incubation buffer (6x5 min), grids were washed in PBS (6x5 min). Sections were subsequently stained with uranyl acetate and observed with a Jeol JEM EXII Transmission Electron Microscope at 100kV. Micrographs were acquired by the Olympus SIS VELETA CCD camera equipped the iTEM software. Control sections were obtained by omitting the primary antibody from the incubation mixture.

**Isolation of Yeast Mitochondria**

Mitochondria were isolated as in Kritsiligkou et al.[34], , 2017. In short, D273-10B yeast were grown to OD600 1 in yeast peptone lactate. Cells were pelleted by centrifugation (3,000g) and washed in ddH2O and the cell pellet weight was calculated. The yeast cells were then washed in 100mM Tris-SO4 pH 9.4, 10mM DTT for 30 minutes in the presence of constant agitation (120rpm). The cells were washed in sorbitol buffer (1.2M Sorbitol, 10mM KPi pH 7.4) followed by resuspension in sorbitol buffer (5ml/g of cells) supplemented with zymolyase 20T (3.5mg/g of cells) and incubation for 45 minutes under constant agitation (120rpm) to create spheroplasts. The spheroplasts created in this process were

washed in sorbitol buffer and resuspended in breaking buffer pH6.0(BB pH6.0) (600mM Sorbitol buffer, 20mM MES-KOH pH 6.0, 1mM PMSF). The spheroplasts were the broken in BB pH6.0 using a glass type B pestle homogeniser for 15 strokes on ice. The cell debris was removed via differential centrifugation; first low speed centrifugation (1,480g 4ºC) then high speed (12,000g 4 ºC). Crude mitochondria were then purified further via gradient centrifugation on a 14.5-20% Nycodenz® gradient and spun at 202,496g at 4 ºC. The pure mitochondria were collected with a 19-gauge needle, washed in BB pH 7.4 (600mM sorbitol buffer, 20mM HEPES-KOH pH7.4) and protein concentration estimated. Finally, the mitochondria are frozen at -80ºC at a concentration of 20mg/ml in BB pH 7.4 supplemented with 10mg/ml fatty-acid free BSA.

**Import of $M_2$ receptor and $M_2$tail(368-466) in isolated yeast mitochondria**

The $M_2$ receptor and $M_2$tail(368-466) were cloned into the pSP64 in vitro translation vector and translated with a 35S radiolabelled methionine label using the TNT® coupled reticulocyte lysate system (Promega) following manufacturers guidelines. Each import reaction comprised of 50µg of isolated yeast mitochondria resuspended in 100µl import buffer 1X (600mM Sorbitol, 2mM KH2PO4, 50mM KCl, 50mM HEPES-KOH pH 7.4, 10mM MgCl2, 2.5mM Na2EDTA pH 7, 5mM L-methionine, 1mg/ml fatty acid free BSA) supplemented with 2mM ATP and 2.5mM NADH. 5µl of TNT translated protein was added to each import reaction and the samples were incubated at 30ºC for 5,10 or 30 minutes. Import was ceased by the transfer of samples on ice. Where indicated, samples were treated with 0.1mg/ml Trypsin in breaking buffer (600mM Sorbitol, 20mM HEPES-KOH pH 7.4) for 30 minutes on ice followed by protease inactivation with the addition of soybean trypsin inhibitor (SBTI)(1mg/ml) for 10 minutes. Mitochondria were reisolated by centrifugation (16,000g, 4ºC) and resuspended in either 2X Laemmli sample buffer (65mM Tris-HCl pH 6.8, 25% [v/v] glycerol, 2% SDS, 0.01% bromophenol blue) for SDS-PAGE

analysis or solubilization buffer (0.1mM EDTA, 50mM NaCl, 10% [v/v] glycerol, 20mM Tris-HCl pH 7.4, 1% [w/v] digitonin) for Blue Native-PAGE analysis. For NaCO3 extraction 100µg of mitochondria were pelleted after SBTI at 16,000g, 4ºC and resuspended in 200ul of 0.1M NaCO3 for 30 minutes on ice followed by ultracentrifugation (100,000g 30 minutes 4ºC). Supernatants were TCA-precipitated (10% [v/v] final) for 20 minutes on ice followed by centrifugation (16,000g, 4ºC, 20 minutes). The TCA precipitated supernatant and the pellet after ultracentrifugation were resuspended in 2X Laemmli sample buffer and proteins were separated by SDS-PAGE.

**Animal Experiments**

All animal experiments were carried out according to the German animal welfare act, considering the guidelines of the National Institute of Health and the 2010/63/EU Directive of the European Parliament on the protection of animals used for scientific purposes. The animals had free access to food and water and were kept in individually ventilated cages under a 12h:12h light/dark regime (light from 6:30 am to 6:30 pm), a constant 22±2°C temperature and 55±10% humidity.

The transgenic line B6.Cg-Chrm2tm1.1Hze/J, also known as Chrm2-tdT-D knock-in, was obtained from ‘The Jackson Laboratory’ (Stock No: 030330). They are heterozygous animals, designed to have expression of a Chrm2/tdTomato fusion protein directed by the endogenous M2 muscarinic acetylcholine receptor promoter/enhancer sequences.

**Tissue preparation and immunoblot of endogenous $M_2$ receptor in Chrm2-tdT-D knock-in mice**

Mouse tissues were weighed out and ice-cold lysis buffer was added to approx. 0.2-0.5mg tissue/ml buffer (10mM K2HPO4, 150mM NaCl, 5mM EDTA, 5mM EGTA, 1% Triton X-100, 0.2% Na-deoxycholate. Fresh protease inhibitors PMSF 0.5mM, Na3VO4 0.1mM, NaF 50mM, Jenny’s mix (Soybean trypsin inhibitor 3.2µg/ml, Aprotinin 2µg/ml, Benzamidin 0.5mM) were added to the buffer.

Samples were first homogenized, and then pellet debris were centrifuged at 4°C for 30 minutes at max speed (tabletop) and the supernatant (soluble proteins) was transferred into fresh tube (pellets = non-soluble proteins)

Protein concentrations were determined with BCA assay. Cell lysates were loaded using Laemmli Buffer 2x (Sigma Aldrich) in equal mass (see above for the immunoblot procedure) into 8% polyacrylamide gels.

The membrane was then incubated overnight at 4 °C with primary antibody solution (1:1000 anti β-actin (13E5) rabbit primary antibody #4970 from Cell Signaling) (1:1000 anti GAPDH) (1:1000 anti-TdTomato rabbit primary antibody #632496 from Takara).

**Isolation of mitochondrial fractions and immunodetection**

HEK293 cells were seeded in 15 cm plates and transfected after 24h according to manufacturer protocols (Effectene, Qiagen) using 5 µg of plasmid DNA. 48h after transfection cells were harvested and centrifuged at 1000 rpm for 10 minutes, resuspended in NaCl 0.9% (w/v) and centrifuged at 1000 rpm for 10 minutes.

Mouse tissues were freshly excised and placed in ice cold PBS (1x). The samples were washed with NaCl 0.9% (w/v). The homogenization steps were performed with Precellys Evolution Homogenizer, at 4500 rpm for 10 seconds.

The samples were processed according to manufacturer protocols (Qproteome Mitochondria Isolation Kit, Qiagen). From the different steps were obtained three different fractions: the cytosolic, the microsomal and the mitochondrial fraction.

The fresh mitochondrial pellet was resuspended in 150µL of ice-cold lysis buffer and the protein concentration of the three fractions from each sample was quantified with a BCA assay (see above).

Cell lysates were loaded into 12% polyacrylamide gels using an SDS loading buffer (6x solution: 2.28g Tris-HCl, 2.313 DTT, 6g SDS, 30% Glycerol and 0.03% bromophenol blue in a final volume of 50 mL) in equal mass (see above for the immunoblot procedure).

The membrane was then incubated overnight at 4 °C with primary antibody solution using the following concentrations: 1:1000 anti β-actin (13E5) rabbit primary antibody (#4970 from Cell Signaling), 1:1000 anti-tdtomato rabbit primary antibody (#632496 from Takara), 1:1000 anti β-tubulin mouse primary antibody, 1:1000 anti GAPDH mouse primary antibody, 1:1000 anti COXIV mouse primary antibody (#ab33985 from abcam).

**Quantification of $M_2$tail(368-466) mitochondrial import kinetics from confocal time sequences** Before imaging, $2.5 \times 10^4$ HEK-TSA cells were seeded in each PDL-coated well of the µ-Slide 8 well (Ibidi) with DMEM (ScienCell™) supplemented with 10% FBS and 1%

P/S overnight, and transfected with a total of 2 µg C-terminal EGFP tagged $M_2$ tail using Lipofectamine 2000 (Thermo Fisher Scientific) according to the manufacturer protocol after washing with 200 µL PBS and refreshing with 100 µL DMEM (ScienCell™) supplemented with 10% FBS and 1% P/S. After transfection for five hours, mitochondria of transfected cells were rapidly labelled with 50nM MitoTracker® Deep Red FM (Invitrogen™, Thermo Scientific) in FluoroBrite DMEM medium (Thermo Scientific) supplemented with 1% P/S for 20 minutes after the removal of old culture medium. After mitochondria labeling, the µ-Slide were washed twice with FluoroBrite DMEM medium (Thermo Scientific) and subsequently mounted onto the TCS SP8 confocal microscope (Leica) (See imaging). Firstly, the stage of the microscope was equilibrated in temperature, and the acquisition mode was set to the tile scanning. After storing the position of each desired transfected cells, the imaging mode was set to XYT and a HC PL APO CS2 40×/NA1.3 oil immersion objective was selected. Both DIC channel and HyD were utilized to obtain 512×512 pixels images. Laser wavelengths were set as described in the section Fluorescence Microscopy above. All settings remained the same throughout the whole measurement. Images were taken at every 30 minutes over a 5h interval. The images were afterwards analyzed by ImageJ software, using the Plugin 'JACoP'. Briefly, the degree of colocalization between EGFP-tagged $M_2$tail(368-466) (CH1) and MitoTracker stained mitochondria (CH2) was quantified by Mander's colocalization coefficients (40) [38]. The two colocalization coefficients M1 ($M1 = \frac{CHB}{CH1}$) and M2 ($M2 = \frac{CHB}{CH2}$), CHB indicates overlapping pixels in channel 1 and channel 2. A threshold was determined for individual channel in order to distinguish specific signals from nonspecific ones. Due to their sensitivities to thresholds, the Pearson correlation coefficient (PCC)[36, 37] , as well as the faster decaying attribute of nonspecific signals, were also taken into account while estimating thresholds for each channel. For the analysis of each cell, the threshold for Mitotracker channel was kept constant. Whereas the threshold for EGFP channel was evaluated based on the PCC value.

**Transfection and expression of split-GFP constructs in HEK293 cells**

To perform the sequential split GFP complementation assay illustrated in **Figs 4D and 5C-D**, $3{\times}10^5$ HEK-TSA cells were seeded on a 10% poly-D-lysine (PDL)-coated 25mm coverslip in each well of a Falcon® 6-well flat-bottom plate (StemCell™) and cultured overnight. Cells were transfected using Effectene (see above) with a 0.4 µg Mito-GFP1-10 plasmid. The cells were then washed once with fresh 2 mL phosphate-buffered saline (PBS) first and supplemented with 1.5 mL fresh DMEM medium (ScienCell™) supplemented with

10% FBS and 1% P/S. After adding the transfection mixture, the cells were cultured overnight. After the first transfection, a second transfection of a 0.6 µg plasmid encoding either the C-terminal GFP11 tagged $M_2$tail(368-466) or $M_2$ receptor was performed using the Effectene Transfection Reagent (Qiagen). After washing each well with 2 mL PBS, the old medium was exchanged with 1.5 mL low glucose Minimum Essential Medium (MEM) (Gibco™, Thermo Scientific) supplemented with 1% P/S, in order to create a stress-like cell starvation condition. After a four hours starvation, the cells were fixed using 4% polyformaldehyde (PFA)/PBS at 37℃ for 30 minutes and washed twice with PBS before imaging.

**Oxygen consumption measured with the Seahorse assay**

$5x10^4$ COS7 cells were seeded in seahorse 24MW plates with Complete media (DMEM supplemented with 10%FBS and 1X PS) and then incubated for about 4h at 37C in a cell incubator. After this incubation, cells were washed twice with Seahorse media supplemented with glutamate (1 mM), pyruvate (1 mM) and glucose (20 mM) and then incubated for 1h at 37 °C in an incubator without CO2 before being analyzed in the Seahorse XF Analyzers. In particular, the baseline cell oxygen consumption rate (OCR) was measured at the beginning of the assay, followed by measurements of ATP linked respiration and maximal respiratory capacity after the addition of respectively oligomycine (1 µM) and the FCCP protonophore (1 µM). Finally, rotenone (1 µM), an inhibitor of complex I, and antimycin (1 µM), inhibitor of complex III, were added to shut down completely the electron transport chain (ETC) function, revealing the non-mitochondrial respiration.

**Extracellular oxygen consumption**

HEK293 cells 24h after transfection and hPSCs were harvested and seeded respectively in 96 multiwell black plate and pre-coated (Geltrex A1413302, Thermo Fisher) 96 multiwell black plate, $5x10^4$ cells for well; after incubation O/N at 37 °C and in 5% CO2, the cell culture medium was replaced by HBSS and the assay were performed according to the manufacturer protocols (abcam Extracellular Oxygen Consumption Assay).

**Generation of M368A hiPSC clone**

The human induced pluripotent stem cell (hiPSC) line BIHi005-A (https://hpscreg.eu/cell-line/BIHi005-A) was used to generate an isogenic iPSC clone (BIHi005-A-39) with M368A mutation using a protocol previously described (42) [40]. In brief, small guide RNA (sgRNA)

targeting the $M_2$ gene close to the mutation site to be corrected were designed using the http://crispor.tefor.net/ webpage and the sgRNA (5`- AGTCATCTTCACAATCTTGC-3`) were synthesized by Integrated DNA technologies (https://eu.idtdna.com). The donor ssODN template was designed to convert the ATG to GCT and was synthesized as an Ultramar DNA oligo by IDT. (5´-AAAAGTGACTCATGTACCCCAACTAATACCACCGTGGAGGTAGTGGGGTCTTCAGGTCAGAATGGAGATGAAAAGCAGAATATTGTAGCCCGCAAGATTGTGAAGGCTACTAAGCAGCCTGCAAAA-3´). The Ribonucleoprotein (RNP) complexes were prepared by incubating of 1.5µg Cas-9 protein and 360 ng gRNA for 10 minutes at room temperature. For delivery of RNPs and ssODN template, 10µl cell suspension containing Cas9 RNPs and ssODN and 1x105 cells were electroporated using the Neon transfection System (ThermoFisher Scientific). The electroporated cells were plated in one well of 6 well plate with StemFlex media (ThermoFisher Scientific) supplemented CloneR™ (Stemcell technologies). Three days after the transfection we analyzed the bulk population using the Sanger sequencing to estimate the editing efficiency. Thereafter, the automated single cell cloning of the genome edited cell pool was performed as described in protocol [38] . The clones were screened by SANGER sequencing. The positive confirmed clones were banked and characterized for pluripotency and karyotype stability as described in [39] .

Briefly, for pluripotency assay, single cells were labelled with conjugated antibodies. Surface markers (SSEA1, SSEA4 and TRA1-60) staining was performed in unfixed cells by incubation with antibodies diluted in 0.5% BSA/PBS (10 min, 4 °C, dark). Intracellular markers (OCT3/4, NANOG) were stained by fixation/permeabilization solution (Miltenyi Biotec, 130-093-142) (30 min, 4 °C, dark) followed by antibody incubation in permeabilization buffer (Miltenyi Biotec) (30 min, 4 °C, dark). Analysis was done using the MACSQuant® AnalyzerVYB and FlowJo v10.4.

Briefly, for karyotype stability, genomic DNA was isolated using the DNeasy blood and tissue kit (Qiagen, Valencia, CA, United States) and samples were analyzed using the human Illumina OMNI-EXPRESS-8v1.6 BeadChip. First, the genotyping was analyzed using GenomeStudio 1 genotyping module (Illumina). Thereafter, KaryoStudio 1.3 (Illumina) was used to perform automatic normalization and to identify genomic aberrations utilizing settings to generate B-allele frequency and smoothened Log R ratio plots for detected regions. The stringency parameters used to detect copy number variations (CNVs) were set to 75 kb (loss), 100 kb (gain) and CN-LOH (loss of heterozygosity) regions larger than 3 MB.

**Culture and handling of hiPSCs cells**

hiPSCs were cultured on Geltrex (Thermo Fisher) coated plates using: Essential 8 medium (Thermo Fisher). The culture was routinely monitored for mycoplasma contamination using a PCR assay. 10 μM ROCK Inhibitor Y-27632 2HCl (Selleck Chemicals, #SEL-S1049) was added after cell splitting to promote survival. hiPSC cultures were kept in a humidified atmosphere of 5% CO2 at 37°C and 5% oxygen. hiPSCs were undirectedly differentiated to a mixture of three lineages (ectodermal, endodermal and mesodermal) by replacing Essential 8 medium with DMEM-F12 medium supplemented with 10% FBS for seven days. 10 μM ROCK Inhibitor Y-27632 2HCl (Selleck Chemicals) was added after cell splitting to promote survival.

**Cell proliferation assay with HEK293 cells**

$4x10^5$ HEK293 cells 24h after transfection and $4x10^5$ hPSCs were seeded respectively in T25 flask and pre-coated T25 flask, in culture medium with Atropine at final concentration of 100nM; the medium with Atropine was replaced every day. After 72h from transfection of the HEK293 and from seeding of the hiPSCs, the medium and the cells were collected and centrifuged, the pellet was resuspended in the same amount of medium for each pellet; an aliquot of the resuspension was mixed with the same amount of Trypan blue (ratio 1:1), 10 microliters were used for counting in a Neubauer chamber.

**Fluorescence plate reader assays**

HEK293 cells were grown on 6 well plates without coverslips, and, upon reaching 60-70% confluence, the cells were transfected using Effectene (Quiagen, Venlo Netherlands) according to the manufacturer's instructions. After 24 h the cells were split and transferred to 96 well (black plate) at a seeding density of 50,000 cells/well. The 96 well plate was pre-coated with Poly-D-Lysine for 30 min at RT. The medium was removed and each well was washed 3x with 100µl HBSS. The cells were finally analyzed by a fluorescence plate reader (Neo2, BioTek, Bad Friedrichshall) in HBSS.

**Statistical analysis**

Significance values reported in the graphs, in comparing tabulated values, were determined by a one-tailed Student t-test using Prism 9 (GraphPad Software), and significance values

(p-values) are indicated according to the following legend: **** p<0.0001; *** 0.0001<p<0.001; ** 0.001<p<0.01; * 0.01<p<0.05. p-values larger than 0.05 are deemed non-significant. Unless otherwise indicated, bar charts and scatter plots indicate average value with s.e.m.

## Acknowledgments

We are grateful to Dr. Marco Marcia (EMBL) for insightful discussion. I.P acknowledges a short term exchange grant from the Deutscher Akademischer Austauschdienst (DAAD). F.P. Acknowledges support from the University of L'Aquila towards international mobility.

## Funding:

European Union's Horizon2020 Marie Skłodowska-Curie Actions (MSCA) Program under Grant Agreement 641833 (ONCORNET). This project was funded by the Deutsche Forschungsgemeinschaft (DFG, German Research Foundation) through Project 421152132 SFB1423 subproject C03 (P.A. and M.J.L.)

## Author contributions:

Conduced primary experiments: FP, IF, PA, ZW, MR, IP, RE, HZ, UZ, MET, JDG, SS, ART,SA, MC

Contributed to the experimental data: MG, CP, GA, FM, BP, AR, EC

Contributed tools and reagents: NT and SD

Contributed to experimental design and assisted in writing paper: PA, IP, IF, MR, UZ, AT, KT, VF, FA, FG, MS

Conceived and lead the study, and wrote the paper: PA, MJL and RM

## Competing interests:

The authors declare that they have no conflicts of interest with the contents of this article.

## Data and materials availability:

Source data will be provided with this manuscript.

## References


1. Kruse AC, Kobilka BK, Gautam D, Sexton PM, Christopoulos A, Wess J. Muscarinic acetylcholine receptors: novel opportunities for drug development. Nature Reviews Drug Discovery. 2014;13(7):549-60. doi: 10.1038/nrd4295.
2. Ad F, A C, Nm N. Muscarinic Receptors. 2012 ed: Springer Berlin Heidelberg; 2012.
3. Novi F, Scarselli M, Corsini GU, Maggio R. The Paired Activation of the Two Components of the Muscarinic M3 Receptor Dimer Is Required for Induction of ERK1/2 Phosphorylation. Journal of Biological Chemistry. 2004;279(9):7476-86. doi: 10.1074/jbc.M306818200.
4. Calebiro D, Nikolaev VO, Persani L, Lohse MJ. Signaling by internalized G-protein-coupled receptors. Trends in Pharmacological Sciences. 2010;31(5):221-8. doi: 10.1016/j.tips.2010.02.002.
5. Zhang X, Bedigian AV, Wang W, Eggert US. G protein-coupled receptors participate in cytokinesis. Cytoskeleton. 2012;69(10):810-8. doi: 10.1002/cm.21055.
6. !!! INVALID CITATION !!! [6, 7].
7. Maggio R, Fasciani I, Rossi M, Gregorio JD, Pietrantoni I, Puca V, et al. Variants of G protein-coupled receptors: a reappraisal of their role in receptor regulation. Biochemical Society Transactions. 2016;44(2):589-94. doi: 10.1042/BST20150239.
8. Jackson RJ, Kaminski A. Internal initiation of translation in eukaryotes: the picornavirus paradigm and beyond. RNA (New York, NY). 1995;1(10):985-1000.
9. Hellen CUT, Sarnow P. Internal ribosome entry sites in eukaryotic mRNA molecules. Genes & Development. 2001;15(13):1593-612. doi: 10.1101/gad.891101.
10. Popot JL, Engelman DM. Membrane protein folding and oligomerization: the two-stage model. Biochemistry. 1990;29(17):4031-7. doi: 10.1021/bi00469a001. PubMed PMID: 1694455.
11. Maggio R, Vogel Z, Wess J. Reconstitution of functional muscarinic receptors by co-expression of amino- and carboxyl-terminal receptor fragments. FEBS Letters. 1993;319(1-2):195-200. doi: 10.1016/0014-5793(93)80066-4.
12. Isbilir A, Serfling R, Moller J, Thomas R, De Faveri C, Zabel U, et al. Determination of G-protein-coupled receptor oligomerization by molecular brightness analyses in single cells. Nat Protoc. 2021;16(3):1419-51. Epub 20210129. doi: 10.1038/s41596-020-00458-1. PubMed PMID: 33514946.
13. Bathe-Peters M, Gmach P, Annibale P, Lohse MJ. Linescan microscopy data to extract diffusion coefficient of a fluorescent species using a commercial confocal microscope. Data in Brief. 2020;29:105063-. doi: 10.1016/j.dib.2019.105063.
14. Dong C, Filipeanu CM, Duvernay MT, Wu G. Regulation of G protein-coupled receptor export trafficking. Biochimica et Biophysica Acta (BBA) - Biomembranes. 2007;1768(4):853-70. doi: 10.1016/j.bbamem.2006.09.008.
15. Kamiyama D, Sekine S, Barsi-Rhyne B, Hu J, Chen B, Gilbert LA, et al. Versatile protein tagging in cells with split fluorescent protein. Nature Communications. 2016;7(1):11046-. doi: 10.1038/ncomms11046.
16. Newman LE, Schiavon C, Kahn RA. Plasmids for variable expression of proteins targeted to the mitochondrial matrix or intermembrane space. Cellular Logistics. 2016;6(4):e1247939-e. doi: 10.1080/21592799.2016.1247939.
17. Thakor N, Holcik M. IRES-mediated translation of cellular messenger RNA operates in eIF2α-independent manner during stress. Nucleic Acids Research. 2012;40(2):541-52. doi: 10.1093/nar/gkr701.
18. Holcik M, Sonenberg N. Translational control in stress and apoptosis. Nature Reviews Molecular Cell Biology. 2005;6(4):318-27. doi: 10.1038/nrm1618.
19. Bonfili L, Cecarini V, Cuccioloni M, Angeletti M, Flati V, Corsetti G, et al. Essential amino acid mixtures drive cancer cells to apoptosis through proteasome inhibition and autophagy activation. The FEBS Journal. 2017;284(11):1726-37. doi: 10.1111/febs.14081.
20. Ishizuka T, Ozawa A, Katsuura M, Nomura S, Satoh Y. Effects of muscarinic acetylcholine receptor stimulation on the differentiation of mouse induced pluripotent stem cells into neural progenitor cells. Clinical and Experimental Pharmacology and Physiology. 2018;45(11):1198-205. doi: 10.1111/1440-1681.12993.
21. Hoogduijn MJ, Cheng A, Genever PG. Functional Nicotinic and Muscarinic Receptors on Mesenchymal Stem Cells. Stem Cells and Development. 2009;18(1):103-12. doi: 10.1089/scd.2008.0032.
22. Weinberg ZY, Puthenveedu MA. Regulation of G protein-coupled receptor signaling by plasma membrane organization and endocytosis. Traffic. 2019;20(2):121-9. doi: 10.1111/tra.12628.
23. Jong Y-JI, Harmon SK, O'Malley KL. GPCR signalling from within the cell. British Journal of Pharmacology. 2018;175(21):4026-35. doi: 10.1111/bph.14023.


**Formatted:** Font: (Default) Arial

**Formatted:** Font: (Default) Cambria Math

**Formatted:** Font: (Default) Arial

24. Bénard G, Massa F, Puente N, Lourenço J, Bellocchio L, Soria-Gómez E, et al. Mitochondrial CB1 receptors regulate neuronal energy metabolism. Nature Neuroscience. 2012;15(4):558-64. doi: 10.1038/nn.3053.

25. Suofu Y, Li W, Jean-Alphonse FG, Jia J, Khattar NK, Li J, et al. Dual role of mitochondria in producing melatonin and driving GPCR signaling to block cytochrome c release. Proceedings of the National Academy of Sciences. 2017;114(38). doi: 10.1073/pnas.1705768114.

26. Lefkimmiatis K, Leronni D, Hofer AM. The inner and outer compartments of mitochondria are sites of distinct cAMP/PKA signaling dynamics. Journal of Cell Biology. 2013;202(3):453-62. doi: 10.1083/jcb.201303159.

27. Marti-Solano M, Crilly SE, Malinverni D, Munk C, Harris M, Pearce A, et al. Combinatorial expression of GPCR isoforms affects signalling and drug responses. Nature. 2020;587(7835):650-6. doi: 10.1038/s41586-020-2888-2.

28. Pestova TV, Shatsky IN, Hellen CU. Functional dissection of eukaryotic initiation factor 4F: the 4A subunit and the central domain of the 4G subunit are sufficient to mediate internal entry of 43S preinitiation complexes. Molecular and Cellular Biology. 1996;16(12):6870-8. doi: 10.1128/MCB.16.12.6870.

29. Fitzgerald KD, Semler BL. Bridging IRES elements in mRNAs to the eukaryotic translation apparatus. Biochimica et Biophysica Acta (BBA) - Gene Regulatory Mechanisms. 2009;1789(9-10):518-28. doi: 10.1016/j.bbagrm.2009.07.004.

30. Jan E, Sarnow P. Factorless Ribosome Assembly on the Internal Ribosome Entry Site of Cricket Paralysis Virus. Journal of Molecular Biology. 2002;324(5):889-902. doi: 10.1016/S0022-2836(02)01099-9.

31. Ingolia NT, Hussmann JA, Weissman JS. Ribosome Profiling: Global Views of Translation. Cold Spring Harbor Perspectives in Biology. 2019;11(5):a032698-a. doi: 10.1101/cshperspect.a032698.

32. van Heesch S, Witte F, Schneider-Lunitz V, Schulz JF, Adami E, Faber AB, et al. The Translational Landscape of the Human Heart. Cell. 2019;178(1):242-60.e29. doi: 10.1016/j.cell.2019.05.010.

33. Guo C, Sun L, Chen X, Zhang D. Oxidative stress, mitochondrial damage and neurodegenerative diseases. Neural regeneration research. 2013;8(21):2003-14. doi: 10.3969/j.issn.1673-5374.2013.21.009.

34. Kritsiligkou P, Chatzi A, Charalampous G, Mironov A, Grant CM, Tokatlidis K. Unconventional Targeting of a Thiol Peroxidase to the Mitochondrial Intermembrane Space Facilitates Oxidative Protein Folding. Cell Reports. 2017;18(11):2729-41. doi: 10.1016/j.celrep.2017.02.053.

35. Bonner TI, Buckley NJ, Young AC, Brann MR. Identification of a Family of Muscarinic Acetylcholine Receptor Genes. Science. 1987;237(4814):527-32. doi: 10.1126/science.3037705.

36. Manders EM, Stap J, Brakenhoff GJ, van Driel R, Aten JA. Dynamics of three-dimensional replication patterns during the S-phase, analysed by double labelling of DNA and confocal microscopy. Journal of Cell Science. 1992;103(3):857-62. doi: 10.1242/jcs.103.3.857.

37. Manders EMM, Verbeek FJ, Aten JA. Measurement of co-localization of objects in dual-colour confocal images. Journal of Microscopy. 1993;169(3):375-82. doi: 10.1111/j.1365-2818.1993.tb03313.x.

38. Vallone VF, Telugu NS, Fischer I, Miller D, Schommer S, Diecke S, et al. Methods for Automated Single Cell Isolation and Sub-Cloning of Human Pluripotent Stem Cells. Current Protocols in Stem Cell Biology. 2020;55(1). doi: 10.1002/cpsc.123.

39. Metzler E, Telugu N, Diecke S, Spuler S, Escobar H. Generation of two human induced pluripotent stem cell lines derived from myoblasts (MDCi014-A) and from peripheral blood mononuclear cells (MDCi014-B) from the same donor. Stem Cell Research. 2020;48:101998-. doi: 10.1016/j.scr.2020.101998.

**Formatted:** Font: (Default) Arial, German (Germany)

**Formatted:** Font: (Default) Arial

**Formatted:** Font: (Default) Cambria Math

**Formatted:** Font: (Default) Arial
