# Supplementary material for: The C-terminus of the prototypical M2 muscarinic receptor localizes to the mitochondria and regulates cell respiration under stress conditions
Source: PLoS Biol. 2024 Apr 29;22(4):e3002582. doi: 10.1371/journal.pbio.3002582 (PMC11093360; doi:10.1371/journal.pbio.3002582)
Supplement: Attachment — Submitted filename: Response to Reviewers.docx [file pbio.3002582.s019.docx]

St Andrews, 09.11.2023

Dear Ines,

We have addressed below, and in the revised manuscript, the concerns raised by the reviewer (#2) and the academic editor, as well as the follow up indications you provided. Without further instructions concerning controls mentioned by the academic editor, we have decided against conducting more animal experiments, and worked in providing meaningful controls to the experiments that had already been shown. Most of the material shown in the rebuttal letter to the referees in July is now included in the manuscript, of which we enclose a version in track changes to display our edits. We hope that this addresses any outstanding questions, and we look forward to hearing back from you.

Sincerely,

Roberto and Paolo

----------------------------------------------------------------

Reviewers' comments

Rev. #2 comments:
The authors address the comments reasonably well. I appreciate that the authors inserted the figures into the text which makes the resolution so much better. That alone makes the paper easier to understand.

One comment:

M2R molecular weight, I wouldn't call the insect cell expressed M2R "naked" because they are also glycosylated, but the pattern could be different that from the HEK cell. It could be better that the authors provide the references for that the M2R run at 72kDa. A quick literature search reveals mixed results, some papers show 52 kDa and some show ~72 kDa. I am not 100% convinced that glycosylation can cause a difference as big as 20 kDa in mobility shift.

**We very much thank Reviewer 2 for their encouraging comments following our revision. The M2R expressed using the Promega TNT Coupled Reticulocyte Lysate Systems allows indeed generating a receptor which will have a different set of post-translational modifications than those translated in mammalian cells. Moreover, the running weight for the M2R may depend on whether samples have been boiled or not (as in our case) before running. In their 1995 study published in the Journal of Biological Chemistry, McKinnon and Nathanson demonstrated that Y1 epithelial cells, when heterologously transfected, exhibit a distinct M2R band ranging from 66 to 97 kDa.**


Academic Editor's comments

I still find the manuscript difficult to follow. Both reviewers raised legitimate concerns and while the authors made a real effort to address these, only minor changes were made in the manuscript. Furthermore, many concerns raised by the reviewers regarding controls are not clearly addressed in the rebuttal, making it very difficult to follow the logic. As presented, most of the westerns do not have appropriate controls, or the controls are scattered in different blots and figure panels. Therefore, to meet the standards of PLOS Biology, the authors need to provide a significantly revised version of their manuscript that not only incorporates appropriate controls for each experiment, but also includes the data they provided to the reviewers in a clear and logical manner.

IA-G: ...I have obtained now more specific comments from the Academic Editor that may help you with the revision. We generally felt that many of the comments present in the rebuttal were not incorporated in the manuscript.

For example, for the answer of Reviewer 1 Point 2 you include four different western blots (Fig. 1-4 for reviewers), but these, as far as we can see, are not present in the figures and should be added.

**We thank the academic editor both for reading the manuscript and providing their comments. We also acknowledge that, despite addressing in full reviewers concerns in the rebuttal letter, we did not incorporate most of these additional control data in the manuscript. We have now corrected this. Specifically:**

1. **By incorporating Western blots from lysates of whole HEK293 expressing the key constructs M2wt-myc, M2tail-myc, as well as myc-M2trunk and myc-M2stop228 (with appropriate loading controls) in Figure S2C and D. Moreover the WB of these constructs was also conducted in COS-7 cells (Figure S2E).**

**We have also added controls to the blot where they were not included:**

2. **In Figure 3C the blot from the mitochondrial fraction now shows also a control using β-actin (no bands) alongside COXIV, to demonstrate the purity of the mitochondrial preparations.**
3. **A β-actin control was also added to the corresponding supernatant/microsomal blot reported in Figure S6K.**

**Concerning Westerns from the transgenic Achm2r-td-tomato mice**

4. **The Western blot of mitochondrial preparation from these mice in Figure 7B includes now -more appropriately- the control with tubulin, to demonstrate the purity of the preparations.**
5. **Finally, positive control of Caspase cleavage requested by the reviewers and provided in our rebuttal, are now included in Figure S10D.**

**Original micrographs from all Western blots are provided separately according to Journal guidelines.**

Similarly, both Reviewers 1 and 2 raised concerns about the distribution of the M2 receptor on Fig. 3B and this needs to be addressed in the manuscript, not only in the rebuttal.

6. **We further expanded the text describing Figure 3B, clarifying that the homogeneous distribution of the ‘red’ M_2_receptor arises from the fact that we are imaging the basal membrane of the cell. We feel, and we hope the Academic editor will agree, that including the collection of published examples displayed in Reviewer Figure 5 is at this point not necessary, and we just cited the relevant previous manuscript from our group.**

It is the same case for EM controls, which are shown in Fig. 10 for reviewers, but they are not present in the manuscript – we also worry about the strong EM-gold pattern in the control cells, which is not clear to us if this is background granularity as you mention.

7. **We have added the EM control data as a new panel Figure S7E, and provide now representative zoom-ins to clarify that the apparent ‘dark spots’ that are visible in the control, likely due to a different exposure settings at the time of imaging, are significantly larger and with less defined shapes as opposed to the circular, sharp and intense gold nanoparticles visible in the transfected samples. We enclose the expanded image (now Figure S7E) below for your appraisal. We have specifically selected in the control one of the most prominent ‘dark spots’ to emphasize how it does not, nor any of its parts, reflect the sharp circular shape observed when gold nanoparticles are present.**

In addition, you also need to include labels for all the panels of the IF images shown in the figures. We might have missed some instances, so please check carefully the rebuttal and make sure that all the information is incorporated to the manuscript.

8. **We apologise for this oversight, and have added labels to the fluorescence microscopy images where they were missing, namely in Figure 5, Figure S6, S7, S8 and S10. This should now better guide the reader to appraise the content of each subpanel.**
